# Supplementary material for: Migrant-friendly maternity care in Montreal, Canada: A cross-sectional study on migrant women’s care perspectives
Source: PLoS One. 2025 Aug 21;20(8):e0330830. doi: 10.1371/journal.pone.0330830 (PMC12370051; doi:10.1371/journal.pone.0330830)
Supplement: S2 Appendix — (PDF) [file pone.0330830.s002.pdf]

استبيان العناية الطبيّة الأمويّة المخصّصة للنساء المهاجرات

MFMCQ-MTL – Arabic – February 2014

تعليمات للمقابلون

1. أثناء المقابلة، يرجى قراءة كل سؤال للمرأة ببطء وبوضوح. اتبع التعليمات التالية لخيارات الاجوبة على النحو التالي:

- بالنسبة للأسئلة التي تقول **اقرأ جميع الخيارات بصوت عالي وضع علامة على كل ما ينطبق**، أقرأ كل إجابة واسمح لها للرد **بنعم** أو **لا** لكل من خيارات الجواب.

- بالنسبة للأسئلة التي تقول **اسمح الأم للرد وضع علامة على كل ما ينطبق**، اطرح هذا السؤال دون أن تعطيها أي من الخيارات ومن ثم قرر أي جواب يطابق جوابها. إذا لم تستطع بالتفكير في إجابة أقرأ 2-3 من خيارات الجواب لمساعدتها.

2. اتبع التعليمات حول خيارات التخطي.

على سبيل المثال:
Q10 يسأل عن الخدمات التي كانت تود الأم أن تستخدمها لكن لم تفعل ذلك. اسألQ11 " إذا كنت لم تحصلي على الرعاية التي تريدي لهذا الحمل، ما كانت العوائق ؟" فقط إذا أشارت الأم في Q10 أن هناك خدمة أرادت أن تستخدمها .

3. لأي سؤال يتعلق بإطار زمني (مثلاً Q2,6) شجع المرأة للحصول على أفضل إجابة بامكنها أن تعطي.

4. إذا كنت غير متأكد من الجواب (أي مضاعفات طبية أو عملية)، أضع إشارة عند "أخرى" واكتب التفسير.

5. للكلمات التي قد لا تفهمها المرأة، استخدام هذه التعاريف/التفسيرات:
   - **تنظيم الأسرة:** التنظيم لإنجاب الأطفال، واستخدام وسائل منع الحمل
   - **الأمراض المنقولة جنسيا:** مرض أو عدوى المشتركة بالجماع الجنسي
   - **التبنيج:** الادوية التي تعطى لجعل الشخص يفقد الشعور أو يشعر بالخدر في منطقة معينة (المحلي) أو في كل مكان (عام)
   - **وحدة خاصة لرعاية الأطفال:** الأم والطفل انفصلوا لأن الطفل في حاجة إلى أن يراقب من قبل اخصائيي الرعاية الصحية لقلق طبي.
   - **الترويح:** فقدان الحمل قبل 20 أسبوعا. إسقاط الطفل.
   - **الإجهاض:** إنهاء الحمل. إزالة الطفل قبل أن ينمو.
   - **وضع الهجرة** (Q93) : إبتداءً من اليوم الذي وصلوا في هذا البلد، وليس في اليوم الذي تلقو أوراقهم
   - **مركز احتجاز المهاجرين:** اسئلة محتملة: هل سبق أنك سجنتي من قبل سلطات الهجرة؟ هل سبق أنك سجنتي لأسباب الهجرة؟
   - **الدخل:** دخل كل فرد يعيش في المنزل (على سبيل المثال، أخت، زوج، الأم) قبل الضرائب
   - **كم شخص يدعم:** ويشمل شقيقة، أطفالها، الخ
   - **المخاض:** بداية التقلصات (الألام) حتى ولادة الطفل

NOTES: (1) Questions marked with * (n=86) were identified (during a Delphi consensus process with international perinatal health research experts) as a minimum set of questions for use in international comparisons; (2) Questions marked with M are those only relevant for migrant women or identified as recommended migration indicators to capture in analyses of perinatal health (see Gagnon AJ, Zimbeck M, Zeitlin J. Migration and Perinatal Health Surveillance: An International Delphi Survey. *European Journal of Obstetrics & Gynecology and Reproductive Biology*. 2010;149(1), 37-43).

بداية المقابلة: ملخص الدراسة للأمهات

أنا أعمل مع فريق من الباحثين المهتمين في تجارب الأمومة للنساء المهاجرات في بلدهم الجديد. سأسألك أسئلة حول تجاربك أثناء الحمل والمخاض(بداية التقلصات والالام) والولادة، وفترة بعد ولادة طفلك، و تجارب أمومة بشكل عام. ثم سيكون هناك سلسلة من الأسئلة حول تاريخ توليدك وهجرتك. خلال المقابلة يمكنك أن تطلبي مني إعادة قراءة السؤال، أو توضيح أو شرح مسألة ما. وأود أيضا أن أكرر أن جميع المعلومات التي تعطيها سوف تبقى خاصة. يمكنك سحب مشاركتك من الدراسة في أي وقت، و يمكنك عدم الرد على أي أسئلة كنت لا تشعري بالراحة بالرد عليها.

واسمحي لي أن أعرف في أي لحظة إذا كان لديك أي أسئلة. هل لديك أي أسئلة قبل أن نبدأ؟

حسناء، لنبدأ

**MFMCQ Arabic Version**		**رقم الهوية**	
**ساعة البدئ**		**إسم المقابل**	
**ساعة الإنتهاء**		**تاريخ المقابلة**	

1. *M **في أي بلد ولدتي؟**

____

2. M* **منذ متى وانت تعيشي في هذا البلد؟**
(الوقت **الشامل** التي عاشت فيه الأم في البلد. خلال الهجرة الكثير من النساء تأتي وتذهب قبل الإنتقال كلياً إلى بلد.)

_____ (أيام) _____ (اسابيع) _____ (اشهر) _____ (سنين)

هذه المجموعة من الأسئلة حول <u>حملك الأخير</u> في هذا البلد، وهناك 14 سؤالا في هذا القسم.

3. M **هل أتيتي إلى هذه البلاد حامل بالمولود الجديد؟**

- ☐ نعم، كم أسبوع حامل كنت؟_____
- ☐ لا
- ☐ لا أعلم

4. * **هل حصلتي على رعاية صحية خلال هذا الحمل (مثل طبيب، ممرضة، أو قابلة مولدة)؟**

- ☐ نعم ________________________ (في أي بلاد)
- ☐ لا *(تخطى إلى Q8)*

5. **من الذي قدم الرعاية لهذا الحمل في هذا البلد؟**
(اسمح الأم للرد، وضع علامة على كل ما ينطبق؛ شجع إذا لزم الأمر)

- ☐ طبيب عائلي، طبيب صحة عامة
- ☐ طبيب نسائي
- ☐ قابلة
- ☐ ممرضة
- ☐ أخرى، *(يرجى التحديد)* ______________
- ☐ N/A

---

6. ***كم أسبوعا حامل كنت عندما تلقيتي أول رعاية لهذا الحمل؟ ___________(اسابيع) ;في هذا البلد؟ ___________ (اسابيع)**
(لا تعتبر الزيارة إذا كانت فقط لفحص حمل)

☐ N/A (لا رعاية خلال هذا الحمل)

---

7. ***كم مرة قمتي بزيارة طبيب أو ممرضة أو قابلة مولدة (sage-femme) أثناء الحمل؟ _________**

☐ N/A لا رعاية

---

8. *** هل إختبرتي مضاعفات طبية خلال الحمل؟**

- ☐ نعم***(اسمح الأم للرد، وضع علامة على كل ما ينطبق)***
  - ☐ فقر دم
  - ☐ ارتفاع ضغط الدم
  - ☐ تسمم الحمل preeclampsia (يعرف بارتفاع ضغط الدم، والبروتين الزائد في البول.)
  - ☐ مخاض قبل الأوان
  - ☐ خثار الدم DVT (جلطة الدم التي تشكل في أحد الأوردة العميقة في الجسم)
  - ☐ سكر الحمل
  - ☐ المشيمة المنزاحة (حالة يكون فيها الكيس في أسفل الرحم).Placenta praevia
  - ☐ تمزق المشيمة
  - ☐ التهاب المسالك البولية
  - ☐ آلام شديدة في الظهر
  - ☐ نزول الماء قبل الأوان
  - ☐ اكتئاب
  - ☐ أخرى، *(يرجى التحديد)* ____________________________(تشمل مضاعفات الجنين)
- ☐ لا، لم يكن لديك أي مضاعفات طبية أثناء الحمل هذه

9. أثناء هذا الحمل، أي من الخدمات التالية إستخدمتي ؟

	نعم	لا
دروس الحمل/ الولادة	☐	☐
موعد مع أخصائي الرعاية الصحية	☐	☐
بنوك الطعام	☐	☐
مساعدة البحث عن المسكن	☐	☐
الأدوية التقليدية/طقوس	☐	☐
خدمات الأسرة (مثلاً رعاية الأطفال، والإرشاد، وصفوف تربية الأطفال)	☐	☐
الاختبارات الطبية أثناء الحمل (مثلاً فحص الدم، اختبار عنق الرحم)	☐	☐
فحوص للكشف عن العيوب الولادية (Down Syndrome/Trisomy)	☐	☐
التصوير بالموجات فوق الصوتية (Echographie)	☐	☐
خدمات الدعم (مثلاً خدمات الصحة النفسية)	☐	☐
أخرى، *(يرجى التحديد)* ____________________	☐	☐

10.* أثناء هذا الحمل هل أحببتي استخدام أية من الخدمات التالية، ولكن لم تستخدميها؟
(اقرأ جميع الخيارات بصوت عالي وضع علامة على كل ما ينطبق)

- ☐ دروس الحمل/ الولادة
- ☐ موعد مع أخصائي الرعاية الصحية
- ☐ بنوك الطعام
- ☐ مساعدة البحث عن المسكن
- ☐ الأدوية التقليدية / طقوس
- ☐ خدمات الأسرة (مثلاً رعاية الأطفال، والإرشاد، وصفوف تربية الأطفال)
- ☐ الاختبارات الطبية أثناء الحمل (مثلاً فحص الدم، pap test)
- ☐ فحوص للكشف عن العيوب الولادية (Down Syndrome/Trisomy)
- ☐ التصوير بالموجات فوق الصوتية (Echographie)
- ☐ خدمات الدعم (مثلاً خدمات الصحة النفسية)
- ☐ أخرى، *(يرجى التحديد)* ______________________
- ☐ لا *(انتقل إلى Q12)*

11. ***إذا لم تحصلي على الرعاية التي أردتيها خلال الحمل، ما كانت العوائق؟**
(اسمح الأم للرد، وضع علامة على كل ما ينطبق)

- ☐ لم تقدم الخدمات في منطقتك
- ☐ كانت الخدمات ممتلئة
- ☐ لم تعلمي بوجود الخدمات
- ☐ لم تعلمي أنك كنت مؤهلة للحصول على هذه الخدمات
- ☐ كنت غير مؤهلة للحصول على الخدمات
- ☐ لم تعرفي أين تقدم هذه الخدمات
- ☐ كنت خائفة أن يتأثر طلب الهجرة
- ☐ مقدم الخدمة ألغى موعدك
- ☐ لم تتوفر خدمة رعاية الطفل
- ☐ كان هناك حاجز اللغة
- ☐ لم يكن لديك وسيلة نقل
- ☐ لأسباب مادية
- ☐ كنت تعملين
- ☐ لم يكن لديك الوقت
- ☐ كنت بحاجة أن تبقي في المنزل
- ☐ الخوف من الفحوصات الطبية
- ☐ حصلت على المساعدة والإرشاد من أصدقاء والعائلة
- ☐ العناية التي حصلتي عليها لم تكن كما كنت تتوقعينه من نظام الرعاية الصحية
- ☐ واجهتي صعوبات في فهم كيفية عمل نظام الرعاية الصحية أو واجهتي مشاكل في إستخدام خدماته
- ☐ شعرتي بالحرج
- ☐ أسباب إدارية (مثلاً عدم وجود التأمين)
- ☐ أخرى، *(يرجى التحديد)*______________________
- ☐ N/A

---

12. ***خلال هذا الحمل، من أو ما كان أهم مصدر لك للحصول على معلومات عن الحمل، المخاض والولادة؟**
(اسمح الأم للرد، وضع علامة على كل ما ينطبق)

- ☐ حمل سابق
- ☐ عائلة أو أصدقاء
- ☐ مرشد روحي
- ☐ طبيب نسائي
- ☐ طبيب صحة عامة/طبيب عائلي
- ☐ قابلة مولدة
- ☐ ممرضة
- ☐ دروس الحمل/ الولادة
- ☐ الكتب
- ☐ التلفزيون
- ☐ الانترنت
- ☐ أخرى، *(يرجى التحديد)*______________________

---

13. M ***أثناء هذا الحمل، هل أعطاك أخصائيو الرعاية الصحية معلومات في لغتك؟**

- ☐ نعم ________________ *(يرجى التحديد)*
- ☐ لا

14.* أثناء هذا الحمل، قبل المخاض والولادة، هل كان لديك معلومات كافية حول المواضيع التالية؟

	نعم	لا	لا أعرف
التغييرات الجسدية خلال الحمل	☐	☐	☐
التغييرات العاطفية خلال الحمل	☐	☐	☐
علامات بداية المخاض	☐	☐	☐
الأدوية	☐	☐	☐
ما يمكن توقعه خلال المخاض أو الولادة	☐	☐	☐
علاج الآلام دون مسكنات/أدوية	☐	☐	☐
الفحوصات الطبية اللازمة	☐	☐	☐
التغذية أثناءالحمل	☐	☐	☐
صحتك والتعافي بعد الولادة	☐	☐	☐
تغيرات في المزاج	☐	☐	☐
كيفية حمل طفلك	☐	☐	☐
كيفية التعرف على مشاكل في صحة و نمو طفلك	☐	☐	☐
الرضاعة الطبيعية	☐	☐	☐
الحليب البودرة/ الرضاعة الإصطناعية	☐	☐	☐
بمن تتصلي إذا كان لديك تساؤلات حول صحتك أو صحة طفلك	☐	☐	☐
تنظيم الأسرة/ منع الحمل	☐	☐	☐
فيروس نقص المناعة المكتسبة (VIH/HIV) والأمراض الأخرى المنقولة جنسياً	☐	☐	☐

15.* هل سألوك أخصائيو الرعاية الصحية كيف تخططين لإطعام طفلك؟

- ☐ نعم
- ☐ لا
- ☐ لا أعلم/ لا أذكر
- ☐ N/A (عدم وجود أخصائي الصحة)

16.*هل سألوك أخصائيو الرعاية الصحية إذا كنت تفضلين متابعة أي رعاية أو ممارسة معينة أثناء الحمل؟

- ☐ نعم
- ☐ لا
- ☐ N/A (عدم وجود أخصائي الصحة)

المجموعة التالية من الاسئلة هي عن المخاض و ولادة آخر طفلك. هناك 16 سؤال في هذ القسم.

17.* كم أسبوع حامل كنت عندما ولدتي؟ ______ (اسابيع)

- ☐ لا أعلم

18. *** كم طفل ولدتي في أخر حملك؟ _______________ (مثلاً: طفل واحد، توأم إلخ...)**

19. *** ما كان وزن طفلك (أو أطفالك) عند الولادة؟**

______(كيلو) ______ (غرام)/ ______ (رطلا) ______ (أوقية)

______(كيلو) ______ (غرام)/ ______(رطلا)______ (أوقية) ***(إذا انجبت أكثر من طفل)***

~~**20.** *** أين كنت عندما ولدتي؟**~~
~~***(اقرأ جميع الخيارات بصوت عالي وضع علامة على ما ينطبق)***~~

21. *** أي نوع من أخصائي الرعاية الصحية قدم الرعاية خلال معظم مخاضك ؟**
(اسمح الأم للرد، وضع علامة على ما ينطبق)

- ☐ طبيب نسائي
- ☐ طبيب صحة عامة/طبيب عائلي
- ☐ القابلة المولدة
- ☐ ممرضة
- ☐ أخرى، *(يرجى التحديد)*___________________
- ☐ لا أحد
- ☐ لا أحد، لا مخاض، قيصري مخطط
- ☐ لا أعلم

22. *** أي نوع من أخصائي الرعاية الصحية قدم الرعاية خلال معظم ولادة طفلك؟**
(اسمح الأم للرد، وضع علامة على ما ينطبق)

- ☐ طبيب نسائي
- ☐ طبيب صحة عامة/طبيب عائلي
- ☐ القابلة المولدة
- ☐ ممرضة
- ☐ أخرى، *(يرجى التحديد)*___________________
- ☐ لا أحد
- ☐ لا أعلم

23. * **هل خضعتي لأي من الاجراءات التالية أثناء المخاض والولادة؟**

	نعم	لا
تحريض المخاض (لتبدأ التقلصات)	☐	☐
زيادة المخاض (زيادة سرعة وقوة تقلصاتك)	☐	☐
إستخدام ملقط (أداة معدنية لإزالة الطفل)	☐	☐
الإستخراج بأداة شفط (ventouse)	☐	☐
الولادة القيصرية	☐	☐
بضع الفرج (Episiotomy/episiotomie) شق بالقرب من فتحة المهبل	☐	☐
Epidural للوجع خلال المخاض	☐	☐
تبنيج العمود الفقري للولادة القيصرية	☐	☐
تبنيج عام	☐	☐
أخرى، (*يرجى التحديد*)________________	☐	☐

24. * **هل كانت هناك أي تعقيدات طبية أثناء المخاض والولادة؟**
(مثلاً:، نزيف بعد الولادة إلتهاب، تمزق الرحم، مشاكل بالطفل)

☐ نعم، (*يرجى التحديد*) ____
☐ لا

إذا ولدت مهبلياً ، إنتقل إلى Q26

25. * **إذا ولدتي قيصرياً، ما السبب الرئيسي لذلك؟**
(اسمح الأم للرد، وضع علامة على ما ينطبق)

☐ كان مخططاً لأن الطبيب اقترحها لأسباب طبية
☐ كان مخططاً لكن لست متأكدة لماذا
☐ كان مخططاً لأنك اردتي ذلك، ولكن ليس لسبب طبي
☐ لم يكن مخططاً له، ولكن مخاضك قد طول جداً
☐ لم يكن مخططاً له، ولكن الطفل كان في خطر
☐ لم يكن مخططاً له، ولكنك كنت في خطر
☐ لم يكن مخططاً له، ولا تعلمي لماذا حصل
☐ أخرى، (*يرجى التحديد*)__________________________
☐ N/A ولدت مهبلياً

26. **أثناء المخاض هل كان بامكانك التحرك أو إختيار وضع مريح؟**
(اقرأ جميع الخيارات بصوت عالي وضع علامة على ما ينطبق)

☐ نعم، دائماً
☐ نعم،أحياناً
☐ نعم، نادراً
☐ لا، لأسباب طبية
☐ لا، لا أعلم لماذا
☐ لا مخاض، قيصري مخطط

27.أثناء المخاض، هل سألوك اخصائيي الرعاية الصحية كيف تريدين مجاوزة الألم؟

☐ نعم
☐ لا
☐ لا أعلم/لا أذكر
☐ لا مخاض، قيصري مخطط

28.أثناء المخاض، هل كنت راضية كيف ساعدوك اخصائيي الرعاية الصحية على مجاوزة الألم؟

☐ نعم
☐ لا
☐ أحياناً
☐ لا مخاض، قيصري مخطط

29.أثناء المخاض، هل سمحت بإختيار أفراد العائلة أو فريق دعم للبقاء معك؟

☐ نعم
☐ لا
☐ أحياناً
☐ لا، لا مخاض، قيصري مخطط

30.* هل كان لديك مرافق معك أثناءالمخاض والولادة؟
(اقرأ جميع الخيارات بصوت عالي وضع علامة على ما ينطبق)

☐ نعم، دائماً
☐ نعم، أحياناً
☐ نعم، نادراً
☐ لا
☐ لا أعلم/لا أذكر

31.* إذا نعم، من؟
(إذا أكثر من واحد يرجى تحديد جميعهم.)

________________ (علاقته لك)
________________ (علاقته لك)
________________ (علاقته لك)
☐ N/A

32.*هل سألوك اخصائيو الرعاية الصحية إذا كنت تفضلين متابعة أي رعاية أو ممارسة معينة أثناء المخاض أو الولادة؟

☐ نعم
☐ لا
☐ لا، لأنني سألتهم قبل أن يسألوني

المجموعة التالية من الاسئلة هي عن فترة ما بعد ولادة طفلك. هناك 14 سؤال في هذا القسم.

33.* هل إحتاج طفلك لعناية خاصة في مكان منفصل عنك؟
(اسمح الأم للرد، وضع علامة على ما ينطبق)

- ☐ نعم، في وحدة العناية الفائقة لحديثي الولادة
- ☐ نعم، في وحدة خاصة لرعاية الأطفال
- ☐ نعم، في دار حضانة حيث يمكن للاطباء والممرضين الاعتناء بهم وغير أطفال
- ☐ نعم (ولكنك لا تذكري/تعرفي أين)
- ☐ لا
- ☐ لا تذكري/تعرفي

34.~~إلى متى بقيتي في المستشفى أو العيادة بعد ولادة طفلك؟~~

35.~~هل تشعرين أن هذا القدر من الوقت كان قصير جدا/طويل جدا/تمام؟~~

☐

36.هل طلب منك اخصائيو الرعاية الصحية إذا كان لديك تفضيلات غذائية (على سبيل المثال: درجة حرارة الطعام، الطعام المعد وفقاً لمعتقداتك الدينية، الأكل النباتي، أوغيرها) خلال بقاءك في المستشفى أو مركز الولادة؟

- ☐ نعم
- ☐ لا
- ☐ لا أعلم/لا أذكر
- ☐ N/A (ولادة في المنزل)

37. *هل سألوك اخصائيو الرعاية الصحية إذا كنت تفضلين متابعة أي رعاية أو ممارسة معينة بعد الولادة؟

- ☐ نعم
- ☐ لا
- ☐ لا أعلم/لا أذكر

38.في الساعة الأولى بعد الولادة، هل أعطوك طفلك لتحمليه الجلد إلى الجلد (مع جلد الطفل يلمس جلدك العاري)؟

- ☐ نعم
- ☐ لا. إذا لا، ما السبب:________________________________

39. ***متى ساعدك ممارس الرعاية الصحية أو متى قدم لمساعدتك لبدء الرضاعة؟**
(اسمح الأم للرد، وضع علامة على ما ينطبق)

- ☐ في الساعة الأولى بعد الولادة
- ☐ ليس على الفور، ولكن بينما كنت لا أزال في المكان الذي ولدت فيه (مركز الولادة، المستشفى، أو المنزل)
- ☐ في وقت لاحق خلال موعد رعاية صحية
- ☐ لم يساعدوا أو لم يقدموا المساعدة
- ☐ لا أعرف / لا أتذكر
- ☐ لم أريد أن أرضع طفلي

---

40. *** هل أعطاك ممارس الرعاية الصحية معلومات حول مصادر للرضاعة في مجتمعك؟**

- ☐ نعم
- ☐ لا، ولكن لم أكن في حاجة إلى معلومات *(تخطى إلى Q42)*
- ☐ لا، ولكن أردت المعلومات *(تخطى إلى Q42)*
- ☐ لا أعرف / لا أتذكر

~~**41.** *إذا نعم، هل إستخدمتي هذه المصادر للرضاعة؟~~

☐

~~**42.** *هل انت أو طفلك زرتم طبيب مختص منذ الولادة لأي سبب مرتبط بهذا الحمل (بما فيه الرعاية الروتينية)؟~~

~~**43.** * إذا نعم، لماذا؟~~ ______________________

~~**44.** *إذا نعم، يرجى تحديد من زرت؟~~
~~*(اسمح الأم للرد، وضع علامة على كل ما ينطبق)*~~

~~**45.** *منذ الولادة، هل أردتي زيارة طبيب مختص لك أو لطفلك لكنك لم تستطيعي ؟~~

☐

---

46. ~~*إذا كنت لا تستطيعي زيارة طبيب مختص، يرجى التحديد لماذا؟~~
~~*(اسمح الأم للرد، وضع علامة على كل ما ينطبق)*~~

هذه المجموعة من الاسئلة هي عن <u>تجربتك الشاملة مع رعاية الأمومة</u> أثناء حملك الأخيرة. هناك 20 سؤالاً في هذا القسم.

47. **عندما تفكري بالتجربة الأن، هل هناك أي نصيحة/دعم/معلومات التي كنت قد ترغبي بالحصول عليها؟**

____

____

48. * **بشكل عام، عندما كنت تجتمعي مع أخصائيي الرعاية الصحية، هل كنت تشعري مرحب بك؟**

a) **أثناء الحمل**
- ☐ دائماً
- ☐ أحياناً
- ☐ نادراً
- ☐ أبداً

b) **أثناء المخاض والولادة**
- ☐ دائماً
- ☐ أحياناً
- ☐ نادراً
- ☐ أبداً

c) **بعد الولادة**
- ☐ دائماً
- ☐ أحياناً
- ☐ نادراً
- ☐ أبداً

49. *بشكل عام، هل كانوا اخصائيي الرعاية الصحية محترمون؟

a) أثناء الحمل
- ☐ دائماً
- ☐ أحياناً
- ☐ نادراً
- ☐ أبداً

b) أثناء المخاض والولادة
- ☐ دائماً
- ☐ أحياناً
- ☐ نادراً
- ☐ أبداً

c) بعد الولادة
- ☐ دائماً
- ☐ أحياناً
- ☐ نادراً
- ☐ أبداً

---

50. * بشكل عام، هل كانوا اخصائيي الرعاية الصحية خدومون ؟

a) أثناء الحمل
- ☐ دائماً
- ☐ أحياناً
- ☐ نادراً
- ☐ أبداً

b) أثناء المخاض والولادة
- ☐ دائماً
- ☐ أحياناً
- ☐ نادراً
- ☐ أبداً

c) بعد الولادة
- ☐ دائماً
- ☐ أحياناً
- ☐ نادراً
- ☐ أبداً

51. * بشكل عام، كنت راضية بالرعاية الصحية التي تلقيتها

a) أثناء الحمل
- ☐ دائماً
- ☐ أحياناً
- ☐ نادراً
- ☐ أبداً

b) أثناء المخاض والولادة
- ☐ دائماً
- ☐ أحياناً
- ☐ نادراً
- ☐ أبداً

c) بعد الولادة
- ☐ دائماً
- ☐ أحياناً
- ☐ نادراً
- ☐ أبداً

52.*خلال فترة الحمل، المخاض، أوالولادة، هل طلب منك اخصائيي الرعاية الصحية ان تفعلي شيئاً كنت لا تريدي فعله؟
- ☐ نعم
- ☐ لا
- ☐ لا أعلم/لا أذكر

---

53. إذا نعم، يرجى تحديد ما كان

____

☐ N/A

---

54. هل سألوك اخصائيو الرعاية الصحية إن كنت تفضلي مقدم رعاية صحية أنثى أو ذكر؟

a) أثناء الحمل
- ☐ دائماً
- ☐ أحياناً
- ☐ نادراً
- ☐ أبداً
- ☐ تعليق____________________________

b) أثناء المخاض والولادة
- ☐ دائماً
- ☐ أحياناً
- ☐ نادراً
- ☐ أبداً
- ☐ تعليق____________________________

c) أول يوم بعد الولادة
- ☐ دائماً
- ☐ أحياناً
- ☐ نادراً
- ☐ أبداً
- ☐ تعليق____________________________

55. *هل فهمت المعلومات التي قدموها اخصائيي الرعاية الصحية؟

a) **أثناء الحمل**
- ☐ دائماً
- ☐ أحياناً
- ☐ نادراً
- ☐ أبداً
- ☐ تعليق____________________________

b) **أثناء المخاض والولادة**
- ☐ دائماً
- ☐ أحياناً
- ☐ نادراً
- ☐ أبداً
- ☐ تعليق____________________________

c) **أول يوم بعد الولادة**
- ☐ دائماً
- ☐ أحياناً
- ☐ نادراً
- ☐ أبداً
- ☐ تعليق ____________________________

---

56. M *هل كنت قد فهمتي المعلومات المقدمة من اخصائيي الرعاية الصحية أفضل في لغة أخرى؟
- ☐ نعم، أية لغة____________________ (مثلاً: اللغة الأم)
- ☐ لا
- ☐ لا أعرف/لا أتذكر

---

57. M *هل عرض عليك اخصائيو الرعاية الصحية خدمة الترجمة؟

a) **أثناء الحمل**
- ☐ نعم
- ☐ لا
- ☐ N/A

b) **أثناء المخاض والولادة**
- ☐ نعم
- ☐ لا
- ☐ N/A

c) **أول يوم بعد الولادة**
- ☐ نعم
- ☐ لا
- ☐ N/A

58. ^{M} ***كم من الأحيان كان شخصاً معك يتكلم لغتك ويترجم لك؟**

a) أثناء الحمل

- ☐ دائماً
- ☐ أحياناً
- ☐ نادراً
- ☐ أبداً
- ☐ N/A

b) أثناء المخاض والولادة

- ☐ دائماً
- ☐ أحياناً
- ☐ نادراً
- ☐ أبداً
- ☐ N/A

c) أول يوم بعد الولادة

- ☐ دائماً
- ☐ أحياناً
- ☐ نادراً
- ☐ أبداً
- ☐ N/A

---

59. ^{M} ***إذا كان لديك شخصاً ليترجم لك، من كان؟**

(اقرأ جميع الخيارات بصوت عالي وضع علامة على ما ينطبق)

a) أثناء الحمل

- ☐ زوج/شريك
- ☐ أفراد الاسرة/صديق
- ☐ ممارس الرعاية الصحية
- ☐ ولدك
- ☐ مترجم محترف
- ☐ مريضٌ آخر أو أفراد أسرة مريضٌ آخر/صديق
- ☐ أخرى، (*يرجى التحديد*)___________________
- ☐ N/A

b) أثناء المخاض والولادة

- ☐ زوج/شريك
- ☐ أفراد الاسرة/صديق
- ☐ ممارس الرعاية الصحية
- ☐ ولدك
- ☐ مترجم محترف
- ☐ مريضٌ آخر أو أفراد أسرة مريضٌ آخر/صديق
- ☐ أخرى، (*يرجى التحديد*)____________________
- ☐ N/A

c) أول يوم بعد الولادة

- ☐ زوج/شريك
- ☐ أفراد الاسرة/صديق
- ☐ ممارس الرعاية الصحية
- ☐ ولدك
- ☐ مترجم محترف
- ☐ مريضٌ آخر أو أفراد أسرة مريضٌ آخر/صديق
- ☐ أخرى، (*يرجى التحديد*)____________________
- ☐ N/A

60. هل كنت راضية بالترجمة؟ M

- ☐ نعم
- ☐ لا
- ☐ لا أعلم/لا أتذكر
- ☐ N/A

61. * أثناء المخاض والولادة، أو بعد الولادة، هل كنت تفضلين متابعة أي رعايةأو ممارسة معينة ولكن لم تستطيعي لأن اخصائيي الرعاية الصحية لم يسمحوا بها/لم يرتبوا ذلك؟

- ☐ نعم
- ☐ لا *(تخطى إلى Q64)*
- ☐ لا أعلم/لا أتذكر

62. إذا نعم، ما كانت التفضيلات ؟

i. ____________
ii. ____________
iii. ____________
☐ N/A

63. إذا نعم، ما الاسباب التي أعطوك أخصائيو الرعاية الصحية لعدم سماحهم لمتابعة هذه التفضيلات؟

i. ________________
ii. ________________
iii. ________________
☐ N/A

64. *هل هناك أي شيء تعتقدين أن أخصائيي الرعاية الصحية يمكنهم أن يفعلوه بشكل مختلف أو أفضل؟

a) أثناء الحمل
- ☐ نعم *(أكمل 65a)*
- ☐ لا
- ☐ لا أعلم/لا أتذكر

b) أثناء المخاض والولادة
- ☐ نعم *(أكمل 65b)*
- ☐ لا
- ☐ لا أعلم/لا أتذكر

c) بعد الولادة
- ☐ نعم *(أكمل 65c)*
- ☐ لا
- ☐ لا أعلم/لا أتذكر

.65 **إذا نعم، يرجى تحديد ما يمكن عمله بشكل مختلف أو أفضل ومن قبل من؟**

(a **أثناء الحمل**

____

(b **أثناء المخاض والولادة**

____

(c **بعد الولادة**

____

.66 ***يرجى وصف أي شيء عن عنايتك أثناء الحمل، الولادة، أو فترة ما بعد الولادة الذي:**

a) **يسعدك**

____

b) **يحزنك**

____

بأخذ اخر حملك في عين الإعتبار، يرجى التحديد إذا العبارات التالية كانت دائماً، أحياناً، نادراً صحيحة، أو أبداً غير صحيحة.

.67 ***أخصائيو الرعاية الصحية سألوني إذا كان لدي أي أسئلة**

- ☐ دائماً
- ☐ أحياناً
- ☐ نادراً
- ☐ أبداً

.68 **أخصائيو الرعاية الصحية كانوا في عجلة**

- ☐ دائماً
- ☐ أحياناً
- ☐ نادراً
- ☐ أبداً

69. *شعرت أن أخصائيو الرعاية الصحية اخذوا مخاوفي جدياً

a) أثناء الحمل

- ☐ دائماً
- ☐ أحياناً
- ☐ نادراً
- ☐ أبدا
- ☐ N/A (لا رعاية حمل)

b) أثناء المخاض والولادة

- ☐ دائماً
- ☐ أحياناً
- ☐ نادراً
- ☐ أبداً
- ☐ N/A (لا أخصائي رعاية صحية)

c) بعد الولادة

- ☐ دائماً
- ☐ أحياناً
- ☐ نادراً
- ☐ أبداً
- ☐ N/A (لا أخصائي رعاية صحية)

70.إنتظرت وقتا طويلا لتلقي الرعاية

a) أثناء الحمل

- ☐ دائماً
- ☐ أحياناً
- ☐ نادراً
- ☐ أبدا
- ☐ N/A (لا رعاية حمل)

b) أثناء المخاض والولادة

- ☐ دائماً
- ☐ أحياناً
- ☐ نادراً
- ☐ أبداً
- ☐ N/A (لا أخصائي رعاية صحية)

c) بعد الولادة

- ☐ دائماً
- ☐ أحياناً
- ☐ نادراً
- ☐ أبداً
- ☐ N/A (لا أخصائي رعاية صحية)

71.*أخصائيو الرعاية الصحية ابقوني على علم بما كان يجري

a) أثناء الحمل

- ☐ دائماً
- ☐ أحياناً
- ☐ نادراً
- ☐ أبدا
- ☐ N/A (لا رعاية حمل)

b) أثناء المخاض والولادة

- ☐ دائماً
- ☐ أحياناً
- ☐ نادراً
- ☐ أبداً
- ☐ N/A (لا أخصائي رعاية صحية)

c) بعد الولادة

- ☐ دائماً
- ☐ أحياناً
- ☐ نادراً
- ☐ أبداً
- ☐ N/A (لا أخصائي رعاية صحية)

72.*شعرت بالراحة لأسأل عن أمور لم أكن أفهمها

a) أثناء الحمل

- ☐ دائماً
- ☐ أحياناً
- ☐ نادراً
- ☐ أبدا
- ☐ N/A (لا رعاية حمل)

b) أثناء المخاض والولادة

- ☐ دائماً
- ☐ أحياناً
- ☐ نادراً
- ☐ أبداً
- ☐ N/A (لا أخصائي رعاية صحية)

c) بعد الولادة

- ☐ دائماً
- ☐ أحياناً
- ☐ نادراً
- ☐ أبداً
- ☐ N/A (لا أخصائي رعاية صحية)

73.*أخصائيو الرعاية الصحية اخذوا قرارات دون الأخذ بعين الإعتبار رغباتي

a) أثناء الحمل

- ☐ دائماً
- ☐ أحياناً
- ☐ نادراً
- ☐ أبدا
- ☐ N/A (لا رعاية حمل)

b) أثناء المخاض والولادة

- ☐ دائماً
- ☐ أحياناً
- ☐ نادراً
- ☐ أبداً
- ☐ N/A (لا أخصائي رعاية صحية)

c) بعد الولادة

- ☐ دائماً
- ☐ أحياناً
- ☐ نادراً
- ☐ أبداً
- ☐ N/A (لا أخصائي رعاية صحية)

74.*أخصائيو الرعاية الصحية كانوا مشجعين ومطمئنين

a) أثناء الحمل

- ☐ دائماً
- ☐ أحياناً
- ☐ نادراً
- ☐ أبدا
- ☐ N/A (لا رعاية حمل)

b) أثناء المخاض والولادة

- ☐ دائماً
- ☐ أحياناً
- ☐ نادراً
- ☐ أبداً
- ☐ N/A (لا أخصائي رعاية صحية)

c) بعد الولادة

- ☐ دائماً
- ☐ أحياناً
- ☐ نادراً
- ☐ أبداً
- ☐ N/A (لا أخصائي رعاية صحية)

75.***هل كان أخصائيي الرعاية الصحية يقضوا وقتا كافيا لتقديم التوضيحات؟**

a) أثناء الحمل

- ☐ دائماً
- ☐ أحياناً
- ☐ نادراً
- ☐ أبدا
- ☐ N/A (لا رعاية حمل)

b) أثناء المخاض والولادة

- ☐ دائماً
- ☐ أحياناً
- ☐ نادراً
- ☐ أبداً
- ☐ N/A (لا أخصائي رعاية صحية)

c) بعد الولادة

- ☐ دائماً
- ☐ أحياناً
- ☐ نادراً
- ☐ أبداً
- ☐ N/A (لا أخصائي رعاية صحية)

76.***بشكل عام، هل شعرتي أن أخصائيي الرعاية الصحية عاملوك بشكل مختلف عن الآخرين؟ (على سبيل المثال بسبب اللغة أو اللهجة، الحضارة، العرق أو لون البشرة، الدين،وضع الهجرة، أو وضع التأمين الصحي .)**

- ☐ دائماً (*يرجى تحديد لماذا في Q77*)
- ☐ أحياناً (*يرجى تحديد لماذا في Q77*)
- ☐ نادراً (*يرجى تحديد لماذا فيQ77*)
- ☐ أبداً (*تخطى إلى Q78*)

77.* **إذا نعم، لأي سبب كان؟**
(اسمح الأم للرد، وضع علامة على كل ما ينطبق)

- ☐ لغة أو لهجة
- ☐ الحضارة
- ☐ العرق
- ☐ لون البشرة
- ☐ الدين
- ☐ وضع الهجرة
- ☐ وضع التأمين الصحي
- ☐ أسباب أخرى، (*يرجى التحديد*)__________________
- ☐ N/A

السلسلة التالية من الأسئلة هي حول تاريخ الولادة الخاصة بك. هناك 8 أسئلة في هذا القسم.

78. * ما عدد المرات التي كنت حامل (بما في ذلك هذا الحمل)؟ ________

~~**79.** *كم حمل إنتهى بترويح الولد ***(fausse-couche/miscarriage)***؟ ________~~

~~*عند طرح هذا السؤال، تأكد من عدم وجود غير أشخاص*~~

~~**80.** *كم حمل أنهيتي (إجهاض) ؟ ________~~

~~**81.** * كم حمل إنتهى بموت الطفل قبل أن يولد؟ ________~~

82. * ما عدد المواليد الأحياء الذي كان لديك قبل 37 أسبوعا؟ ________

- ☐ N/A

83. * ما عدد المواليد الأحياء الذي كان لديك بعد 37 أسبوعا(بما في ذلك المولود الجديد)؟ ________

- ☐ N/A

84. * هل إختبرتي أي مضاعفات طبية في حالات الحمل السابقة؟

- ☐ نعم
- ☐ لا *(تخطى إلى Q86)*
- ☐ N/A *(تخطى إلى Q86)*

85. * إذا كان لديك أي مضاعفات طبية في الحمل السابق، ما كانوا؟
(اسمح الأم للرد، وضع علامة على كل ما ينطبق)

- ☐ الولادة القيصرية
- ☐ فقر دم
- ☐ ارتفاع ضغط الدم
- ☐ تسمم الحمل /preeclampsia (يعرف بإرتفاع ضغط الدم، والبروتين الزائد في البول)
- ☐ مخاض قبل الأوان
- ☐ خثار الأوردة العميقة DVT (جلطة الدم التي تشكل في أحد الأوردة العميقة في الجسم)
- ☐ السكري الحملي
- ☐ مشيمة منزاحة (حالة يكون فيها الكيس في أسفل الرحم)/Placenta praevia
- ☐ تمزق المشيمة
- ☐ التهاب المسالك البولية
- ☐ آلام شديدة في الظهر
- ☐ نزول الماء قبل الأوان
- ☐ اكتئاب
- ☐ أخرى، *(يرجى التحديد)*________________________
- ☐ لا أعلم
- ☐ **N/A**

آخر مجموعة من الأسئلة هي <u>عنك وعن عائلتك</u>. هناك 27 أسئلة في هذا القسم.

86.* ما هو وضعك العائلي؟

- ☐ متزوجة
- ☐ شركاء غير متزوجين
- ☐ أرملة
- ☐ منفصلة
- ☐ مطلقة
- ☐ عزباء

87.* مع من تسكنين؟

	نعم	لا
زوج/شريك ذكر	☐	☐
شريكة أنثى	☐	☐
أمك/أبوك	☐	☐
الإخوة / الأخوات	☐	☐
والد/والدة الشريك	☐	☐
إخوة/أخوات الشريك	☐	☐
أصدقاء	☐	☐
الأطفال (عدا المولود الجديد)	☐	☐
أخرى (*يرجى التحديد*)_________________________	☐	☐
لا أحد، أسكن وحدي مع طفلي	☐	☐
لا أحد، أسكن وحدي	☐	☐

88.* كم من أطفالك يعيشون معك (بما في ذلك المولود الجديد)؟_____________

89. M ***كم من أطفالك ولدوا في هذا البلد (بما في ذلك المولود الجديد)؟**_____________

90.* ما تاريخ ميلادك؟______( ) __________ **(شهر)** _____ **(سنة)**

91. M ***في أي بلد ولدت والدتك؟** ____________

92. M ***في أي بلد ولد والدك؟**____________

هذه الأسئلة تطرح بتفصيلا عن تاريخ هجرتك. نحن مهتمون بهذه المعلومات لمعرفة المزيد عن تجارب المهاجرين إلى هذا البلد. أي معلومات تقدميه ستبقى خاصة ولن تعطى أي معلومات إلى مكتب الهجرة. والإجابة على هذه الأسئلة لن تأثر طلب هجرتك إذا كنت تقدمن للحصول على وضع لاجئة، الإقامة الدائمة، أو المواطنة.

93. M ***ما هو وضع هجرتك الحالي؟**
(اسمح الأم للرد، وضع علامة على ما ينطبق)

- ☐ مهاجرة (permanent resident/landed immigrant)
- ☐ لاجئة
- ☐ طالبة لجوء (Asylum seeker/demandeur d'asile)
- ☐ عاملة مؤقتة/مقدمة رعاية في المنزل (Work permit/live-in caregiver)
- ☐ مقيمة مؤقتة
- ☐ تلميذة
- ☐ زائرة
- ☐ لا وضع
- ☐ لا وثائق
- ☐ مواطنة
- ☐ أخرى، *(يرجى التحديد)*______________

94. M**منذ متى لديك وضع الهجرة ؟ _____ (أيام) ______ (اسابيع) ______ (اشهر) ______ (سنين)**

95. M**هل تغير وضع هجرتك منذ وصولك؟**

- ☐ نعم
- ☐ لا *(تخطى إلى Q97)*

96. M ***إذا نعم، ما كان وضع هجرتك في ما قبل ؟**

- ☐ مهاجرة (Permanent resident/landed immigrant)
- ☐ لاجئة
- ☐ طالبة لجوء (Asylum seeker/demandeur d'asile)
- ☐ عاملة مؤقتة/مقدمة رعاية في المنزل (Work permit/live-in caregiver)
- ☐ مقيمة مؤقتة
- ☐ تلميذة
- ☐ زائرة
- ☐ لا وضع
- ☐ لا وثائق
- ☐ مواطنة
- ☐ أخرى، *(يرجى التحديد)*__________________
- ☐ N/A (لم يتغير الوضع)

97. M**هل كان لديك وضع هجرة كلاجئة؟**

- ☐ نعم
- ☐ لا
- ☐ لا أعلم/لا أتذكر

98. ᴹ**هل قضيتي أي وقت في مركز لاحتجاز المهاجرين؟**

- ☐ نعم
- ☐ لا *(تخطى إلى Q101)*

99. ᴹ**إذا نعم، كم المدة؟ ____ (أيام) _____ (اسابيع) _____ (اشهر) _____ (سنين)**

- ☐ N/A

100. ᴹ***إذا نعم، هل قضيتي أي وقت في مركز لاحتجاز المهاجرين أثناء هذا الحمل؟**

- ☐ نعم
- ☐ لا
- ☐ N/A

101. ***من يتكفل بخدماتك الصحية؟**

	نعم	*لا*	*لا أعلم*
التأمين الصحي العام (RAMQ)	☐	☐	☐
تأمين صحي خاص	☐	☐	☐
تأمين صحي تموله الحكومة خصيصا للاجئين وطالبي اللجوء	☐	☐	☐
انت تدفعين لخدماتك الصحية	☐	☐	☐

102. *** ما هو أعلى مستوى من التعليم الذي أكملتيه؟**

- ☐ مدرسة إبتدائية
- ☐ دبلوم الثانوية
- ☐ دبلوم بعد الثانوية. (على سبيل المثال، مدرسة التجارة، الكلية، الجامعة)
- ☐ دبلوم الدراسات العليا (الماجستير، الدكتوراه)
- ☐ لا شيء

103. ᴹ **هل مسموح لك أن تعملي قانونياً في هذا البلد؟**

- ☐ نعم
- ☐ لا
- ☐ لا أعلم

104. *** ما كانت آخر وظيفة مدفوعة الأجر قبل ولادة طفلك؟ (على سبيل المثال طبيب، معلمة، كاتبة إدخال البيانات،مساعدة في دار رعاية للمسنين، مدبرة منزل، مزارعة الخضار، مشغلة آلة صباغة النسيج، منظفة فنادق، عاملة مركز اتصال.)**

- ☐ *يرجى التحديد*____________________
- ☐ N/A *(لم تعمل)*

.105 ~~*هل عدتي إلى العمل منذ ولادة طفلك؟~~

،

.106 ~~*إذا نعم، ما وظيفتك الحالية؟ (على سبيل المثال طبيب، معلمة، كاتبة إدخال البيانات،مساعدة في دار رعاية للمسنين، مدبرة منزل، مزارعة الخضار، مشغلة آلة صباغة النسيج، منظفة فنادق، عاملة مركز اتصال.)~~

.107 ***بالتفكير في الأسرة بأكملها، إلى أي مجموعة دخل (قبل الضرائب) تنتمين؟**
(أدخل الأرقام المحلية المناسبة بين القوسين وإقرأ الخيارات عالياً)

□ > _$_11,000_
□ ___$11,000__ إلى ___20,999$___ (أي منخفض جداً)
□ ___21,000$__ إلى ___$40,999___ (أي منخفض)
□ ___41,000$__ إلى ___$60,999___ (أي متوسط)
□ ___61,000$__ إلى ___$80,999___ (أي متوسط-عالي)
□ ≥ __81,000$__ (أي عالي)

.108 ***كم شخص يدعم هذا الدخل (بما في ذلك المولود الجديد)؟_______________**

.109 *** أي لغة/لغات تتكلمون معظم الأحيان في المنزل؟ ______________**

.110 **M * إلى أي مدى تتقنين لغة هذا البلد؟**

English

	بفصاحة	*جيداً*	*بصعوبة*	*إطلاقاً*
التكلم	☐	☐	☐	☐
القراءة	☐	☐	☐	☐
الكتابة	☐	☐	☐	☐
الفهم	☐	☐	☐	☐

.111 M إلى أي مدى تتقنين لغة هذا البلد؟

French

	بفصاحة	جيداً	بصعوبة	إطلاقاً
التكلم	☐	☐	☐	☐
القراءة	☐	☐	☐	☐
الكتابة	☐	☐	☐	☐
الفهم	☐	☐	☐	☐

.112 * وهذا يختتم مقابلتنا. هل هناك أي شيء آخر تودي أن تسألي أو تضيفي حول المواضيع الذي ناقشناه؟
