# Supplementary material for: Migrant-friendly maternity care in Montreal, Canada: A cross-sectional study on migrant women’s care perspectives
Source: PLoS One. 2025 Aug 21;20(8):e0330830. doi: 10.1371/journal.pone.0330830 (PMC12370051; doi:10.1371/journal.pone.0330830)
Supplement: S3 Appendix — (PDF) [file pone.0330830.s003.pdf]

Migrant Friendly Maternity Care Questionnaire

MFMCQ-MTL-English – February 2014

Instructions for Interviewers

1. As you conduct the interview, please read each question to the woman slowly and clearly. Follow the instructions below for answer options as follows:
   - For questions that say *read aloud and check all that apply,* read each answer and allow her to reply *yes* or *no* to each of the answer options;
   - For questions that say *allow mother to answer and check all that apply*, ask the question without giving her any of the options and then decide which answer best matches hers. If she can't think of an answer read out 2-3 of the answer options to prompt her.

2. Follow directions about skip options.

   For example:
   Q10 asks which services the mother would have liked to use but didn't. Only ask Q11 "If you did not receive the care you wanted for this pregnancy, what factors or barriers affected this?" if the mother indicated in Q10 that there was a service she wanted to use.

3. For any question to do with time frames (e.g., Q2, 6), prompt the woman for the best response she can give.

4. If you are unsure about which box to check (e.g., medical complication or procedure), check "Other" and write an explanation.

5. For words the woman might not understand, use these definitions/explanations:
   - **Family planning**: planning when to have children and using birth control
   - **Sexually transmitted infections**: illness or infections shared by having sexual intercourse
   - **Anaesthesia**: medication given to make a person lose feeling or feel numb in an area (local) or everywhere (general)
   - **Special care baby unit**: mother and baby were separated because baby needed to be watched by the healthcare staff for a medical concern
   - **Miscarriage**: pregnancy loss prior to 20 weeks of pregnancy
   - **Terminated pregnancy**: removal of baby before it develops; abortion
   - **Immigration status** (Q93): starting the day they arrived in this country, NOT the day they received their papers
   - **Immigration detention centre**: Possible prompts: Have you ever been locked up by immigration authorities? Have you ever been imprisoned for immigration reasons?
   - **Income**: earnings of everyone living in the household (e.g., sister, husband, mother) before taxes
   - **How many does it support**: includes sister, her babies, etc.
   - **Labour**: when contractions (pain) start until the birth of the baby

---

NOTES: (1) Questions marked with * (n=86) were identified (during a Delphi consensus process with international perinatal health research experts) as a minimum set of questions for use in international comparisons; (2) Questions marked with M are those only relevant for migrant women or identified as recommended migration indicators to capture in analyses of perinatal health (see Gagnon AJ, Zimbeck M, Zeitlin J. Migration and Perinatal Health Surveillance: An International Delphi Survey. *European Journal of Obstetrics & Gynecology and Reproductive Biology*. 2010;149(1), 37-43).

Interview Start: Project Summary for Mothers

I am working with a research team interested in migrant women's maternity experiences in their new country. I will be asking you questions about your experiences during your pregnancy, labour and birth, the period after your baby was born, and your overall maternity experiences. Then there will be a series of questions about your obstetrical and migration history. Throughout the interview you are free to ask me to re-read a question, or clarify or explain a question. I would also like to repeat that all the information you give will remain private. You may withdraw your participation from the study at any time, and you may choose to not answer any questions you don't feel comfortable answering.

Please let me know at any point if you have any questions. Do you have any questions before we begin?
Alright, let's get started!

MFMCQ English Version		Study ID:	
START TIME:		Interviewer's Name:	
END TIME:		Interview date:	

1. ***M In which country were you born?**

____

2. *** M How long have you lived in this country?**
(TOTAL amount of time mother lived in this country - often in migration women come and go, before entirely moving to a country)

________ (days) ________ (weeks) ________(months) ________ (years)

This first set of questions is about your MOST RECENT PREGNANCY in this country, there are 14 questions in this section.

3. **M Did you arrive in this country pregnant with the recent baby?**

- ☐ Yes, how many weeks pregnant were you? _______
- ☐ No
- ☐ Don't know

4. ***Did you receive care for this pregnancy from a healthcare professional (such as a doctor, nurse, or midwife)?**

- ☐ Yes ____(in which countries)
- ☐ No *(Skip to Q8)*

5. *Who gave the care for this pregnancy in this country?
(Allow mother to answer and check all that apply; prompt if necessary)

- ☐ Family doctor, General practitioner, GP
- ☐ Obstetrician, Gynaecologist, OBGYN
- ☐ Midwife
- ☐ Nurse Practitioner/Nurse
- ☐ Other *(please specify):* _________
- ☐ N/A

---

6. *How many weeks pregnant were you when you first received care for this pregnancy?_____________(weeks); in this country? _______________(weeks)
(Don't count a visit that was ONLY for a pregnancy test)

- ☐ N/A (no care received during this pregnancy)

---

7. *How many visits did you have with a doctor, nurse, or midwife for this pregnancy? _________

- ☐ N/A (no care received during this pregnancy)

---

8. *Did you have any medical complications during this pregnancy?

- ☐ Yes ***(allow mother to answer and check all that apply)***
  - ☐ Anaemia
  - ☐ High blood pressure
  - ☐ Preeclampsia (gestational hypertension)
  - ☐ Preterm labour
  - ☐ Deep vein thrombosis
  - ☐ Gestational diabetes
  - ☐ Placenta praevia
  - ☐ Placental abruption
  - ☐ Urinary tract infection
  - ☐ Severe back pain
  - ☐ Preterm rupture of membranes
  - ☐ Depression
  - ☐ Other *(please specify):* __________________________ (include fetal complications)
- ☐ No, you did not have any medical complications during this pregnancy

9. During this pregnancy, which of the following services did you use?

	Yes	*No*
Pregnancy classes/childbirth classes	☐	☐
Appointment with a healthcare professional	☐	☐
Food banks	☐	☐
Assistance finding housing	☐	☐
Traditional medicines/ rituals	☐	☐
Family services (e.g., child care, counselling, parenting courses)	☐	☐
Medical tests during pregnancy<br>(e.g., a full physical, blood tests, cervical exam/pap test)	☐	☐
Screening for birth defects (e.g., Down's Syndrome)	☐	☐
Ultrasound scans	☐	☐
Support services (e.g., mental health services)	☐	☐
Other *(please specify):* ____________________	☐	☐

10. *During this pregnancy, would you have liked to use any of the following services, but didn't?
(Read aloud and check all that apply)

- ☐ Pregnancy classes/childbirth classes
- ☐ Appointment with a health care professional
- ☐ Food banks
- ☐ Assistance finding housing
- ☐ Traditional medicines/ rituals
- ☐ Family services (e.g., child care, counselling, parenting courses)
- ☐ Medical tests during pregnancy (e.g., a full physical, blood tests, cervical exam/pap test)
- ☐ Screening for birth defects (e.g., Down's Syndrome)
- ☐ Ultrasound scans
- ☐ Support services (e.g., mental health services)
- ☐ Other *(please specify):* ____________________
- ☐ No *(Skip to Q12)*

11. ***If you did not receive the care you wanted for this pregnancy, what factors or barriers affected this?**
(Allow mother to answer and check all that apply)

☐ Services were not offered in your area
☐ Services were already full
☐ You did not realize services were offered
☐ You did not realize you were eligible for these services
☐ You were not eligible for these services
☐ You did not know where these services were offered
☐ You were scared your immigration application would be affected
☐ Your appointment was cancelled by the provider
☐ Child care was not available
☐ There was a language barrier
☐ You did not have access to transportation
☐ For financial reasons
☐ You were working
☐ You did not have the time
☐ You needed to stay at home
☐ Fear of medical tests or examinations
☐ You got advice & help from family and friends instead
☐ The care you received was not what you were expecting from the healthcare system.
☐ You had difficulties understanding how the healthcare system works and/or problems using the healthcare system
☐ You felt embarrassed
☐ Administrative reasons (e.g., no insurance)
☐ Other *(please specify):* ________________
☐ N/A

---

12. ***During this pregnancy, who or what were your most important source(s) of information about pregnancy, labour and birth?**
(Allow mother to answer and check all that apply)

☐ Previous pregnancy
☐ Family or friends
☐ Spiritual leader
☐ Obstetrician, Gynaecologist, OBGYN
☐ Family doctor, General practitioner, GP
☐ Midwife
☐ Nurse/nurse practitioner
☐ Pregnancy/childbirth classes
☐ Books
☐ Television
☐ Internet
☐ Other *(please specify):* _________________

---

13. *** ^{M} During this pregnancy did a healthcare professional in this country give you information in your language?**

☐ Yes _______________ *(please specify)*
☐ No

---

14. *During THIS pregnancy, before your labour and the birth, did you have enough information about the following topics?

	Yes	*No*	*Don't know*
Physical changes during pregnancy	☐	☐	☐
Emotional changes during pregnancy	☐	☐	☐
Signs of beginning of labour	☐	☐	☐
Medications	☐	☐	☐
What to expect during labour/birth	☐	☐	☐
Non-medication pain therapy	☐	☐	☐
Required medical tests	☐	☐	☐
Nutrition during pregnancy	☐	☐	☐
Your own health and recovery after birth	☐	☐	☐
Mood changes you may have	☐	☐	☐
How to handle your baby	☐	☐	☐
How to recognize problems with the baby's health and development	☐	☐	☐
Breastfeeding	☐	☐	☐
Formula feeding	☐	☐	☐
Who to contact if you have questions about your or your baby's health	☐	☐	☐
Family planning/ birth control	☐	☐	☐
HIV and other sexually transmitted infections/ diseases?	☐	☐	☐

15. *Did your healthcare professional(s) ask you about how you planned to feed your baby?

☐ Yes
☐ No
☐ Don't know/ don't remember
☐ N/A (no healthcare professional)

16. *Did your healthcare professional(s) ask you if you had any preferences about care, or if you wanted to follow any particular custom or practice during pregnancy?

☐ Yes
☐ No
☐ N/A (no healthcare professional)

The next set of questions is about the LABOUR & BIRTH of your MOST RECENT baby. There are 16 questions in this section.

17. *How many weeks pregnant were you when you gave birth? ____________ (weeks)

☐ Don't know

18. ***How many babies did you give birth to? _______________ (i.e., single baby, twins, etc.)**

19. ***How much did your baby(ies) weigh at birth?**

________(kg) ________(grams)/ _______(lbs) ________(oz)

_________(kg) ________(grams)/ _______(lbs) ________(oz) ***(if gave birth to multiples)***

~~**20. *Where were you when you gave birth?**~~
~~***(Read aloud and check one)***~~

21. ***Which type of healthcare professional provided care during most of <u>your LABOUR</u>?**
(Allow mother to answer and check one)

☐ Obstetrician, Gynaecologist, OBGYN
☐ Family doctor, General practitioner, GP
☐ Midwife
☐ Nurse or nurse practitioner
☐ Other *(please specify):* ___________________
☐ None
☐ None, no labour, planned c/s
☐ Don't know

22. ***Which type of healthcare professional provided most of your care during the <u>BIRTH</u> of your baby?**
(Allow mother to answer and check one)

☐ Obstetrician, Gynaecologist, OBGYN
☐ Family doctor, General practitioner, GP
☐ Midwife
☐ Nurse or nurse practitioner
☐ Other *(please specify):* ___________________
☐ None
☐ Don't know

23. *Did you have any of the following procedures during your labour and birth?

	Yes	*No*
Induction of labour (making your contractions start)	☐	☐
Augmentation of labour (making contractions you already have stronger and more frequent)	☐	☐
Use of forceps (metal tool to remove the baby)	☐	☐
Use of vacuum extraction (suction tool to remove the baby)	☐	☐
Caesarean section	☐	☐
Episiotomy (cut near the opening of the vagina)	☐	☐
Epidural for pain during labour	☐	☐
Spinal anaesthesia for Caesarean section	☐	☐
General anaesthesia	☐	☐
Other *(please specify):* __________	☐	☐

24. *Were there any medical complications during your labour and birth?
(Such as: perineal tear, uterine rupture, infection, postpartum haemorrhage, or baby problems)

☐ Yes, *(please specify)* ____

☐ No

If delivered vaginally, skip to Q26

25. *If your baby was born by caesarean section, what was the primary reason for it?
(Allow mother to answer and check one)

☐ It was planned because the doctor suggested it for medical reasons
☐ It was planned but you are not sure why
☐ It was planned because you wanted it, but not for a medical reason
☐ It wasn't planned, but your labour was taking too long
☐ It wasn't planned, but the baby was in danger
☐ It wasn't planned, but you were in danger
☐ It wasn't planned, and you don't know why it happened
☐ Other *(please specify):*____________________
☐ N/A (delivered vaginally)

26. During labour were you allowed to move around or choose comfortable positions?
(Read aloud and check one)

☐ Yes, always
☐ Yes, sometimes
☐ Yes, rarely
☐ No, for medical reasons
☐ No, don't know why
☐ No labour, planned c/s

27. **During labour did the healthcare professionals ask you how you wanted to manage your pain?**

- ☐ Yes
- ☐ No
- ☐ Don't know/ don't remember
- ☐ No, no labour, planned c/s

---

28. **During labour, were you satisfied with how the healthcare professionals helped you to manage your pain?**

- ☐ Yes
- ☐ No
- ☐ Sometimes
- ☐ No, no labour, planned c/s

---

29. **During labour were you allowed to have your choice of family members and/or support people with you?**

- ☐ Yes
- ☐ No
- ☐ Sometimes
- ☐ No, no labour, planned c/s

---

30. ***Did you have any companion with you <u>during labour and birth</u>?**
(Read aloud and check one)

- ☐ Yes, always
- ☐ Yes, sometimes
- ☐ Yes, rarely
- ☐ No
- ☐ Don't know/ don't remember

---

31. ***If YES, Who?**
(If more than one, please mention all)

___________________________(relationship to you)
___________________________(relationship to you)
___________________________(relationship to you)

- ☐ N/A

---

32. ***Did the healthcare professionals ask you if you had any preferences about care, or if you wanted to follow any particular custom or practice during labour or birth?**

- ☐ Yes
- ☐ No
- ☐ No, because I asked them before they asked me

This next set of questions is about your POSTPARTUM period, the time since your baby was born. There are 14 questions in this section.

33. ***Did your baby need to receive special care in an area separate from you?**
(Read aloud and check one)

☐ Yes, in a neonatal intensive care unit
☐ Yes, in a special care baby unit
☐ Yes, in a nursery
☐ Yes (but you don’t know/remember where)
☐ No
☐ Don’t know/ don’t remember

~~34. How long did you stay in the hospital or clinic after your baby was born?~~

~~35. Do you feel that this amount of time was too short/too long/just right?~~

36. **Did the healthcare professionals ask you if you had any food preferences (For example: food temperature, food prepared according to your religious faith, vegetarian, or other food types) during your stay in the hospital/birthing centre?**

☐ Yes
☐ No
☐ Don’t know/ don’t remember
☐ N/A (home birth)

37. ***Did the healthcare professionals ask you if you had any preferences about care, or if you wanted to follow any particular custom or practice after birth?**

☐ Yes
☐ No
☐ Don’t know/ don’t remember

38. **In the first hour after birth, were you given your baby to hold skin-to-skin (with the baby’s bare skin directly next to your bare skin)?**

☐ Yes
☐ No. If no, why not: ____

39. *When did your healthcare professional help you or offer to help you start breastfeeding?
(Allow mother to answer and check one)

- ☐ In the first hour after birth
- ☐ Not immediately, but while I was still in the place where I gave birth (i.e., hospital, birthing centre, or home)
- ☐ At a later date during a healthcare visit/appointment
- ☐ They did not help or offer help
- ☐ Don't know/ don't remember
- ☐ I did not want to breastfeed my baby

40. *Did your healthcare professional give you information about breastfeeding resources in your community?

- ☐ Yes
- ☐ No but I did not need information (*Skip to Q42)*
- ☐ No but I wanted information *(Skip to Q42)*
- ☐ Don't know/ don't remember

41. ~~*If YES, did you use these breastfeeding resources?~~

42. ~~*Have you or your baby seen a healthcare professional since giving birth for any reason (including routine care) associated with this pregnancy?~~

43. ~~*If YES, why? ______________________~~

44. ~~*If YES, who did you see?~~
~~(Allow mother to answer and check all that apply)~~

45. ~~*Since giving birth have you wanted to see a healthcare professional for you or your baby but could not?~~

46. ~~***If you could not see a healthcare professional, why?**~~
~~***(Allow mother to answer and check all that apply)***~~

This set of questions is about your OVERALL MATERNITY HEALTHCARE EXPERIENCE, during your most recent pregnancy. There are 20 questions in this section.

47. Thinking about it now, was there any other advice / support / information you wish you had received?

____
____
____

48. *Overall, when you met with the healthcare professionals, did you feel welcomed by them?

a) During pregnancy

- ☐ Always
- ☐ Sometimes
- ☐ Rarely
- ☐ Never

b) During labour and birth

- ☐ Always
- ☐ Sometimes
- ☐ Rarely
- ☐ Never

c) After birth

- ☐ Always
- ☐ Sometimes
- ☐ Rarely
- ☐ Never

---

49. *Overall, were the healthcare professionals respectful?

a) During pregnancy

- ☐ Always
- ☐ Sometimes
- ☐ Rarely
- ☐ Never

b) During labour and birth

- ☐ Always
- ☐ Sometimes
- ☐ Rarely
- ☐ Never

c) After birth

- ☐ Always
- ☐ Sometimes
- ☐ Rarely
- ☐ Never

---

50. *Overall, were the healthcare professionals helpful?

a) During pregnancy

- ☐ Always
- ☐ Sometimes
- ☐ Rarely
- ☐ Never

b) During labour and birth

- ☐ Always
- ☐ Sometimes
- ☐ Rarely
- ☐ Never

c) After birth

- ☐ Always
- ☐ Sometimes
- ☐ Rarely
- ☐ Never

51. *Overall, you were happy with the healthcare you received

a) During pregnancy

- ☐ Always
- ☐ Sometimes
- ☐ Rarely
- ☐ Never

b) During labour and birth

- ☐ Always
- ☐ Sometimes
- ☐ Rarely
- ☐ Never

c) After birth

- ☐ Always
- ☐ Sometimes
- ☐ Rarely
- ☐ Never

---

52. *During your pregnancy, labour, or birth did the healthcare professionals ever ask you to do something you did not want to do?

- ☐ Yes
- ☐ No
- ☐ Don’t know/ don’t remember

---

53. If YES, what was this?

____

- ☐ N/A

---

54. Did the healthcare professionals ask your preferences about having a female or male healthcare provider?

a) During pregnancy

- ☐ Always
- ☐ Sometimes
- ☐ Rarely
- ☐ Never

Comment________________

b) During labour and birth

- ☐ Always
- ☐ Sometimes
- ☐ Rarely
- ☐ Never

Comment___________

c) In the first day after birth

☐ Always
☐ Sometimes
☐ Rarely
☐ Never
Comment________________

---

55. ***Did you understand the information provided by the healthcare professionals?**

a) During pregnancy

☐ Always
☐ Sometimes
☐ Rarely
☐ Never
Comment______________________

b) During labour and birth

☐ Always
☐ Sometimes
☐ Rarely
☐ Never
Comment__________________

c) In the first day after birth

☐ Always
☐ Sometimes
☐ Rarely
☐ Never
Comment___________________

---

56. * M Would you have understood the information provided by the healthcare professionals better in another language?

☐ Yes, which language ___________________ (e.g., your native language)
☐ No
☐ Don’t know/ don’t remember

---

57. * M Did the healthcare professionals offer you an interpreting service?

a) During pregnancy

☐ Yes
☐ No
☐ N/A

b) During labour and birth

☐ Yes
☐ No
☐ N/A

c) In the first day after birth

☐ Yes
☐ No
☐ N/A

---

58. ***[M] How often was there someone with you who spoke your language and could interpret for you?**

a) During pregnancy

☐ Always
☐ Sometimes
☐ Rarely
☐ Never
☐ N/A

b) During labour and birth

☐ Always
☐ Sometimes
☐ Rarely
☐ Never
☐ N/A

c) In the first day after birth

☐ Always
☐ Sometimes
☐ Rarely
☐ Never
☐ N/A

---

59. ***[M] If you did have someone to interpret for you, who was it?**
(Read aloud and check all that apply)

a) During pregnancy

☐ Husband/partner
☐ Other family member/friend
☐ Healthcare practitioner
☐ Your child
☐ Professional interpreter
☐ Another patient or patient's family member/friend
☐ Other *(please specify):*___________________
☐ N/A

b) During labour and birth

☐ Husband/partner
☐ Other family member/friend
☐ Healthcare practitioner
☐ Your child
☐ Professional interpreter
☐ Another patient or patient's family member/friend
☐ Other *(please specify)*:___________________
☐ N/A

c) In the first day after birth

☐ Husband/partner
☐ Other family member/friend
☐ Healthcare practitioner
☐ Your child
☐ Professional interpreter
☐ Another patient or patient's family member/friend
☐ Other *(please specify):*___________________
☐ N/A

---

60. [M] **Were you satisfied with their interpretation?**

☐ Yes
☐ No
☐ Don't know/ don't remember
☐ N/A

---

61. ***During labour and birth, or after birth, did you have any preferences about care or any particular custom or practice you wanted to follow but couldn't because the healthcare professional(s) wouldn't allow it/arrange it?**

☐ Yes
☐ No *(Skip to Q64)*
☐ Don't know/ don't remember

---

62. **If YES, what were those preferences?**

*i)*____________

*ii)*___________

*iii)*___________

☐ N/A

---

63. **If YES, what reason did the healthcare provider(s) give as to why you were not allowed to follow those preferences?**

*i)*____________

*ii)*___________

*iii)*___________

☐ N/A

---

64. ***Is there anything you think the healthcare professionals could do differently or better?**

a) During pregnancy

☐ Yes *(Complete Q65a)*
☐ No
☐ Don't know/ don't remember

b) During labour and birth

☐ Yes *(Complete Q65b)*
☐ No
☐ Don't know/ don't remember

---

c) After birth

☐ Yes *(Complete Q65c)*
☐ No
☐ Don't know/ don't remember

---

65. If YES, please describe what could be done differently or better and by whom:

a) During pregnancy

____

b) During labour and birth

____

c) After birth

____

---

66. *Please describe anything about your care during pregnancy, birth, or after birth that you are:

a) Particularly happy with

____

b) Particularly unhappy with

____

Thinking about your most recent pregnancy, please tell me how often the following 11 statements were true.

67. *The healthcare professionals asked me if I had any questions

☐ Always
☐ Sometimes
☐ Rarely
☐ Never

---

68. The healthcare professionals were rushed

☐ Always
☐ Sometimes
☐ Rarely
☐ Never

69. *I felt my worries were taken seriously by the healthcare professionals

a) During pregnancy
- ☐ Always
- ☐ Sometimes
- ☐ Rarely
- ☐ Never
- ☐ N/A (did not have pregnancy care)

b) During labour and birth
- ☐ Always
- ☐ Sometimes
- ☐ Rarely
- ☐ Never
- ☐ N/A (no healthcare professional present)

c) After birth
- ☐ Always
- ☐ Sometimes
- ☐ Rarely
- ☐ Never
- ☐ N/A (no healthcare professional present)

---

70. I had to wait too long to receive care

a) During pregnancy
- ☐ Always
- ☐ Sometimes
- ☐ Rarely
- ☐ Never
- ☐ N/A (did not have pregnancy care)

b) During labour and birth
- ☐ Always
- ☐ Sometimes
- ☐ Rarely
- ☐ Never
- ☐ N/A (no healthcare professional present)

c) After birth
- ☐ Always
- ☐ Sometimes
- ☐ Rarely
- ☐ Never
- ☐ N/A (no healthcare professional present)

71. ***The healthcare professionals kept me informed about what was happening**

a) During pregnancy

- ☐ Always
- ☐ Sometimes
- ☐ Rarely
- ☐ Never
- ☐ N/A (did not have pregnancy care)

b) During labour and birth

- ☐ Always
- ☐ Sometimes
- ☐ Rarely
- ☐ Never
- ☐ N/A (no healthcare professional present)

c) After birth

- ☐ Always
- ☐ Sometimes
- ☐ Rarely
- ☐ Never
- ☐ N/A (no healthcare professional present)

---

72. ***I felt comfortable asking about things I did not understand.**

a) During pregnancy

- ☐ Always
- ☐ Sometimes
- ☐ Rarely
- ☐ Never
- ☐ N/A (did not have pregnancy care)

b) During labour and birth

- ☐ Always
- ☐ Sometimes
- ☐ Rarely
- ☐ Never
- ☐ N/A (no healthcare professional present)

c) After birth

- ☐ Always
- ☐ Sometimes
- ☐ Rarely
- ☐ Never
- ☐ N/A (no healthcare professional present)

73. *Decisions were made by the healthcare professionals without my wishes being taken into account

a) During pregnancy

- ☐ Always
- ☐ Sometimes
- ☐ Rarely
- ☐ Never
- ☐ N/A (did not have pregnancy care)

b) During labour and birth

- ☐ Always
- ☐ Sometimes
- ☐ Rarely
- ☐ Never
- ☐ N/A (no healthcare professional present)

c) After birth

- ☐ Always
- ☐ Sometimes
- ☐ Rarely
- ☐ Never
- ☐ N/A (no healthcare professional present)

---

74. *The healthcare professionals were very encouraging and reassuring

a) During pregnancy

- ☐ Always
- ☐ Sometimes
- ☐ Rarely
- ☐ Never
- ☐ N/A (did not have pregnancy care)

b) During labour and birth

- ☐ Always
- ☐ Sometimes
- ☐ Rarely
- ☐ Never
- ☐ N/A (no healthcare professional present)

c) After birth

- ☐ Always
- ☐ Sometimes
- ☐ Rarely
- ☐ Never
- ☐ N/A (no healthcare professional present)

75. *Did the healthcare professionals spend enough time providing explanations?

a) During pregnancy

- ☐ Always
- ☐ Sometimes
- ☐ Rarely
- ☐ Never
- ☐ N/A (did not have pregnancy care)

b) During labour and birth

- ☐ Always
- ☐ Sometimes
- ☐ Rarely
- ☐ Never
- ☐ N/A (no healthcare professional present)

c) After birth

- ☐ Always
- ☐ Sometimes
- ☐ Rarely
- ☐ Never
- ☐ N/A (no healthcare professional present)

---

76. *Overall, do you feel that you were treated differently to other people by healthcare professionals? (For example: because of your language or accent, culture, race or skin colour, religion, migration status, or health insurance status?)

- ☐ Always *(please specify why in Q77)*
- ☐ Sometimes *(please specify why in Q77)*
- ☐ Rarely *(please specify why in Q77)*
- ☐ Never *(Skip to Q78)*

---

77. *If YES, for which reason or reasons do you think it was?
(Allow mother to answer and check all that apply)

- ☐ Language or accent
- ☐ Culture
- ☐ Race/ethnic background
- ☐ Skin colour
- ☐ Religion
- ☐ Migration status
- ☐ Health insurance status
- ☐ Other reason (*please specify*): ___________
- ☐ N/A

The following series of questions is about your OBSTETRICAL HISTORY. There are 8 questions in this section.

78. *How many pregnancies have you had in total (including this one)? _______

~~**79. *How many pregnancies ended in miscarriage? _______**~~

~~***Be sure no one else is present when asking this question***~~

~~**80. *How many pregnancies were terminated? _______**~~

~~**81. *How many pregnancies ended in stillbirths (baby died before being born)? ________**~~

82. *How many of your live babies were born, before 37 completed weeks? _______

☐ N/A

83. *How many live births did you have after 37 completed weeks?________

☐ N/A

84. *Did you have any medical complications in your <u>previous pregnancies</u>?

☐ Yes
☐ No *(Skip to Q86)*
☐ N/A (*Skip to Q86)*

85. ***If you had any medical complications in your previous pregnancy, what were they?**
(Allow mother to answer and check all that apply)

- ☐ Caesarean section
- ☐ Anaemia
- ☐ High blood pressure
- ☐ Preeclampsia (gestational hypertension)
- ☐ Preterm labour
- ☐ Deep vein thrombosis
- ☐ Gestational diabetes
- ☐ Placenta praevia
- ☐ Placental abruption
- ☐ Urinary tract infection
- ☐ Severe back pain
- ☐ Preterm rupture of membranes
- ☐ Depression
- ☐ Other *(please specify):*________________________
- ☐ Don't know
- ☐ N/A

The last set of questions is about YOU AND YOUR FAMILY. There are 27 questions in this section

86. ***What is your marital status?**

- ☐ Married
- ☐ Consensual union (unmarried partners)
- ☐ Widowed
- ☐ Separated
- ☐ Divorced
- ☐ Single

87. ***Who do you live with?**

	Yes	*No*
Husband/ male partner	☐	☐
A female partner	☐	☐
Your mother/father	☐	☐
Your brothers/sisters	☐	☐
Partner's mother/father	☐	☐
Partner's brothers/sisters	☐	☐
Friend(s)	☐	☐
Children (other than newborn)	☐	☐
Other *(please specify)*_______________	☐	☐
No one, I live alone with my baby	☐	☐
No one, I live alone	☐	☐

88. ***How many children of your own are living with you (including your new baby)? ____________**

89.* [M] **How many of your children were born in this country (including your new baby)? ____________**

90.*What is your birth date? ~~_______( )~~ ______________________(month) ___________(year)

91.* [M] **In which country was your mother born? ____________**

92.* [M] **In which country was your father born? ____________**

These next questions ask more detailed information about your immigration history. We are interested in this information because we want to learn more about the experiences of international migrants to this country. Any answers you provide will remain confidential, no information will be given to the immigration office. Answering these questions will not affect your immigration application if you are in the process of applying for refugee status, permanent residency, or citizenship.

93.* [M] **What is your current immigration status?**
(Allow mother to answer and check one)

- ☐ Immigrant (permanent/landed status)
- ☐ Refugee
- ☐ Refugee claimant/Asylum-seeker
- ☐ Temporary worker/Live-in caregiver
- ☐ Temporary resident
- ☐ Student
- ☐ Visitor
- ☐ No status
- ☐ Undocumented
- ☐ Citizen
- ☐ Other *(please specify):* _________________

94.[M] **How long have you had this status? ________ (days) ________ (weeks) ________(months) ________ (years)**

95.[M] **Has your immigration status changed since you arrived?**

- ☐ Yes
- ☐ No *(Skip to Q97)*

96.* M If YES, what was your immigration status prior to this status?

- ☐ Immigrant (permanent/landed status)
- ☐ Refugee
- ☐ Refugee claimant/Asylum-seeker
- ☐ Temporary worker/Live-in caregiver
- ☐ Temporary resident
- ☐ Student
- ☐ Visitor
- ☐ No status
- ☐ Undocumented
- ☐ Other, *(please specify):* ____________
- ☐ N/A (did not change status)

97.M Did you ever have refugee status?

- ☐ Yes
- ☐ No
- ☐ Don't know/ don't remember

98.M Have you ever spent time in an immigration detention centre?

- ☐ Yes
- ☐ No *(Skip to Q101)*

99.M If YES, for how long? ________ (days) ________ (weeks) ________(months) ________ (years)

- ☐ N/A

---

100. * M If YES, did you spend time in an immigration detention centre during this pregnancy?

- ☐ Yes
- ☐ No
- ☐ N/A

---

101. *Who pays for your health services?

	Yes	*No*	*Don't know*
Publicly funded health insurance (RAMQ)	☐	☐	☐
Private health insurance	☐	☐	☐
Special government funded health insurance for refugees and asylum seekers	☐	☐	☐
You pay for your health services	☐	☐	☐

---

102. ***What is your highest level of education completed?**

- ☐ Primary school
- ☐ Secondary diploma
- ☐ Postsecondary diploma (e.g., trade school, college, university)
- ☐ Graduate diploma (Master's, Doctoral)
- ☐ None

---

103. **M Are you legally allowed to work in this country?**

- ☐ Yes
- ☐ No
- ☐ Don't know

---

104. ***What was your last paid job before the baby was born? (e.g., medical doctor, teacher, data entry clerk, nursing home aid, housekeeper, vegetable grower, textile dyeing machine operator, hotel cleaner, call centre caller)**
(please specify): ____________________

- ☐ N/A (did not work)

~~**105.** ***Have you returned to work since the baby was born?**~~

~~**106.** ***If YES, what is your current job? (e.g., medical doctor, teacher, data entry clerk, nursing home aid, housekeeper, vegetable grower, textile dyeing machine operator, hotel cleaner, call centre caller)**~~

---

107. ***Thinking about your entire household, which income group (before taxes) do you belong to?**
(Insert values appropriate to local settings in below and read the numerical range options out loud)

- ☐ < $11,000
- ☐ $11,000 to $20,999
- ☐ $21,000 to $40,999
- ☐ $41,000 to $60,999
- ☐ $61,000 to $80,999
- ☐ ≥ $81,000

---

108. ***How many people does this income support (including the new baby)?** ______________

---

109. ***What language(s) do you speak most often at home?**

____

110. *** M How well do you know this country's language?**

English:

	Fluent	*Well*	*With difficulty*	*Not at all*
Speak	☐	☐	☐	☐
Read	☐	☐	☐	☐
Write	☐	☐	☐	☐
Understand	☐	☐	☐	☐

111. **M How well do you know this country's language?**

French:

	Fluent	*Well*	*With difficulty*	*Not at all*
Speak	☐	☐	☐	☐
Read	☐	☐	☐	☐
Write	☐	☐	☐	☐
Understand	☐	☐	☐	☐

112. ***That concludes our interview. Is there anything else you'd like to say about the topics we've covered? Or anything else you would like to add?**
