# Supplementary material for: Migrant-friendly maternity care in Montreal, Canada: A cross-sectional study on migrant women’s care perspectives
Source: PLoS One. 2025 Aug 21;20(8):e0330830. doi: 10.1371/journal.pone.0330830 (PMC12370051; doi:10.1371/journal.pone.0330830)
Supplement: S4 Appendix — (PDF) [file pone.0330830.s004.pdf]

Soins de maternité qui répondent aux besoins des migrantes
Questionnaire

Les instructions pour les intervieweurs

1. Pendant que vous menez l'entrevue, veuillez lire doucement chaque question à la personne de façon claire. Suivez les instructions indiquées ci-dessous pour les questions à choix multiples:
   - Pour les questions de type *lisez à voix haute puis cochez toutes les réponses qui s'appliquent,* il faut suggérer les choix donnés et recevoir une réponse de type *oui* ou *non* de la personne avant de passer au choix suivant.
   - Pour les questions de type *laissez la personne répondre et cochez toutes les réponses qui s'appliquent*, il faut poser la question sans suggérer à la personne les réponses, puis lorsqu'elle répond vous cochez celles qui sont les plus proches. Si elle n'arrive pas à trouver des réponses, vous pouvez lui en lire deux ou trois pour l'aider.
2. Il faut suivre les instructions des questions de type conditionnel.

   Par exemple:
   Q10 porte sur la présence de services qu'elle aurait aimé recevoir mais qu'elle n'a pas reçus. Il faut uniquement poser Q11 « Si vous n'avez pas bénéficié des services dont vous auriez voulu bénéficier pendant cette grossesse: Pour quelle raison n'avez-vous pas utilisé ou reçu ces services? » si la personne a répondu dans Q10 qu'il y avait des services qu'elle aurait aimé utiliser.

3. Pour les questions qui traitent de temps (ex: Q2, 6), demandez à la femme de donner la meilleure réponse possible.
4. Si vous ne savez pas quelle case cocher (ex: complication médicale, ou procédure), cochez la case « autre » et donnez une explication.
5. Vous pouvez vous référez aux définitions ou explications suivantes lorsque la personne ne comprend pas certains termes:
   - **Planification familiale**: utiliser les méthodes de contrôle de naissance afin de choisir à quel moment les parents veulent avoir des enfants
   - **Infection sexuellement transmissible:** infection qui se transmet entre partenaires au cours des rapports sexuels
   - **Anesthésie:** médicament qui provoque la perte de sensibilité dans un organe (anesthésie locale) ou l'ensemble du corps (anesthésie générale).
   - **Pouponnière de soins spéciaux**: la mère et son nouveau-né ne sont pas ensemble dans la même salle puisque le nouveau-né doit être suivit par l'équipe de santé pour une question médicale
   - **Fausse couche**: interruption involontaire de la grossesse avant la 20e semaine
   - **Grossesse interrompue**: interruption d'une grossesse (avortement)
   - **Statut d'immigration** (Q93): à partir du jour d'arrivée au Canada et non pas du jour de réception des papiers
   - **Centre de détention de l'immigration**: Questions à lui poser: Avez-vous été retenu par les autorités de l'immigration? Étiez-vous emprisonnée pour une question d'immigration?
   - **Salaire:** le salaire total de toutes les personnes qui résident à la maison (ex. sœur, mari, mère, etc.) avant les taxes.
   - **Combien de personnes vivent de ce revenu**: ceci inclus la sœur, ses enfants, etc.
   - **Le travail**: du moment où les contractions (la douleur) commencent jusqu'à ce que le bébé naisse.

NOTES: (1) Les questions marquées par le symbole* (n = 86) ont été identifiées (au cours d'un processus de consensus Delphi avec des experts internationaux de recherche en santé périnatale) comme étant un ensemble de questions de base à utiliser dans les comparaisons internationales; (2) Les questions identifiées par un M concernent uniquement les femmes migrantes, ou sont des questions prescrits comme étant des indicateurs de migration nécessaires dans le cadre d'une analyse de santé périnatale (voir Gagnon AJ, Zimbeck M, Zeitlin J. Migration and Perinatal Health Surveillance: An International Delphi Survey. *European Journal of Obstetrics & Gynecology and Reproductive Biology*. 2010;149(1), 37-43).

Début de l'entrevue: Résumé du projet pour les mères

Je travaille pour une équipe de recherche qui s'intéresse à l'expérience de maternité chez la femme immigrante dans son nouveau pays. Les questions de cette entrevue portent sur votre expérience vécue pendant la grossesse, le travail, l'accouchement, la période après la naissance de votre bébé et votre expérience générale de cette période de maternité. La deuxième partie de ce questionnaire porte sur votre passé obstétrique et votre immigration. Tout au long de l'entrevue je vous encourage à me demander de répéter, d'expliquer ou de clarifier une question au besoin. J'aimerais insister sur le fait que toutes informations que vous partagez restent confidentielles. Vous avez le droit d'arrêter de participer à cette étude à n'importe quel moment. De plus vous avez le droit de ne pas répondre aux questions qui vous mettent mal à l'aise.

Veuillez, s'il vous plaît, m'interrompre si jamais vous avez des questions. Auriez-vous des questions à poser avant de commencer?
Bien, on commence!

MFMCQ French Version		Nº d'indentification:	
L'HEURE AU DEBUT:		Nom de l'intervieweur:	
L'HEURE A LA FIN:		Date de l'entrevue:	

1. ***M Quel est votre pays de naissance?**

____

2. ***M Combien de temps avez-vous vécu dans ce pays?**

(Le nombre TOTAL durant laquelle la personne a vécu dans le pays - souvent les immigrants font beaucoup de va et vient avant de s'installer dans un pays)

_______(jours) _______(semaines) ________(mois) ______(années)

Voici la première série de questions abordant le sujet de votre GROSSESSE dans ce pays. Il y a 14 questions dans cette section.

3. **M Étiez-vous enceinte de votre bébé le plus récent à votre arrivée dans ce pays?**

- ☐ Oui, vous étiez à combien de semaines de grossesse? ________
- ☐ Non
- ☐ Vous ne savez pas

4. ***Durant cette grossesse, aviez-vous reçu des soins de la part d'un professionnel de la santé (tel qu'un médecin, une infirmière, une sage-femme)?**

- ☐ Oui __________________________________ (dans quels pays)
- ☐ Non (*Passez à la Q8*)

5. * Qui vous a donné les soins pour cette grossesse dans ce pays?
(Laissez répondre puis cochez toutes les réponses qui s'appliquent. Lui lire les options si nécessaire.)

☐ Médecin de famille, omnipraticien
☐ Obstétricien/gynécologue
☐ Sage-femme
☐ Infirmier(ière) clinicien(ne)/infirmière
☐ Autre (*veuillez préciser*): _________
☐ S/O

---

6. *A combien de semaines de grossesse avez-vous reçu des soins pour la première fois? _________ (semaines); Dans ce pays:_________________(semaines)
(Une visite qui avait uniquement pour objectif de faire le test de grossesse ne compte pas.)

☐ S/O (pas de soins reçus pendant la grossesse)

---

7. *Combien de fois aviez-vous consulté le médecin, l'infirmière, ou la sage-femme pendant cette grossesse? _________

☐ S/O (pas de soins reçus pendant la grossesse)

---

8. *Avez-vous eu des complications sur le plan médical pendant cette grossesse?

☐ Oui, ***(laissez répondre puis cochez toutes les réponses qui s'appliquent)***
- ☐ Anémie
- ☐ Hypertension arterielle
- ☐ Pre-eclampsie (hypertension artérielle gravidique)
- ☐ Travail prématuré
- ☐ Thrombose veineuse profonde
- ☐ Diabète de grossesse
- ☐ Placenta praevia
- ☐ Décollement placentaire
- ☐ Infection urinaire
- ☐ Douleurs sévères au dos
- ☐ Rupture prématurée des membranes
- ☐ Dépression
- ☐ Autre (*veuillez préciser*): ________________________ (inclure les complications du fœtus)

☐ Non, vous n'avez pas eu de complications sur le plan médical pendant cette grossesse.

9. **Pendant cette grossesse, lesquels parmi les services ci-dessous avez-vous utilisé?**

	Oui	*Non*
Cours pendant la grossesse/ cours prénataux	☐	☐
Rendez-vous avec le professionnel de santé	☐	☐
Banques alimentaires	☐	☐
De l'aide pour trouver un logement	☐	☐
Médicaments et rituels traditionnels	☐	☐
Services à la famille (ex. service de garde, aide psychosociale, enseignements du rôle parental)	☐	☐
Tests médicaux pendant la grossesse (tests sanguins, examen médical, examen cervical, test PAP)	☐	☐
Dépistage de maladies héréditaires (ex: Trisomie et autre)	☐	☐
Échographie	☐	☐
Services de soutien (santé mentale)	☐	☐
Autre, *(veuillez préciser)* ____________________	☐	☐

10. ***Pendant cette grossesse, y avait-il des services, dans la liste ci-dessous, dont vous auriez aimé profiter mais que vous n'avez pas reçus?**
(Lisez à voix haute puis cochez toutes les réponses qui s'appliquent)

☐ Cours pendant la grossesse/ cours prénataux
☐ Rendez-vous avec le professionnel de santé
☐ Banques alimentaires
☐ De l'aide pour trouver un logement
☐ Médicaments et rituels traditionnels
☐ Services à la famille (ex. service de garde, aide psychosociale, enseignements du rôle parental)
☐ Tests médicaux pendant la grossesse (ex. tests sanguins, examen médical, examen cervical, test PAP)
☐ Dépistage de maladies héréditaires (ex. Trisomie et autre)
☐ Échographie
☐ Services de soutien (santé mentale)
☐ Autre (*veuillez préciser*): ________________________________
☐ Non (*Passez à la Q12)*

11. *Si vous n'avez pas bénéficié des services dont vous auriez voulu bénéficier pendant cette grossesse: Pour quelle raison n'avez-vous pas utilisé ou reçu ces services?
(*Laissez répondre puis cochez toutes les réponses qui s'appliquent.)*

- ☐ Les services n'étaient pas offerts dans votre région
- ☐ Les services étaient déjà complets
- ☐ Vous ne saviez pas que ces services étaient offerts
- ☐ Vous ne saviez pas que vous aviez droit à ces services
- ☐ Vous n'aviez pas le droit à ces services
- ☐ Vous ne saviez pas où ces services étaient offerts
- ☐ Vous aviez peur que ça affecte votre demande d'immigration
- ☐ Votre rendez-vous a été annulé par le professionnel de la santé
- ☐ Les services de garde n'étaient pas disponibles
- ☐ Il y avait une barrière linguistique
- ☐ Vous n'aviez pas de moyen de transport
- ☐ Problème financier
- ☐ Vous travailliez
- ☐ Vous n'aviez pas le temps
- ☐ Vous aviez besoin de rester à la maison
- ☐ Vous aviez peur des examens médicaux
- ☐ Vous receviez des conseils et de l'aide de la part de votre famille ou de vos amis(ies) à la place
- ☐ Les soins reçus étaient différent de ce à quoi que vous vous attendiez de la part du système de santé
- ☐ Vous aviez du mal à comprendre le fonctionnement du « système de santé » ou vous aviez de la difficulté à avoir recours au « système de santé »
- ☐ Vous étiez gênée
- ☐ Problèmes administratives (ex: pas d'assurance maladie)
- ☐ Autre, (*veuillez préciser*):_______________
- ☐ S/O

---

12. *Pendant cette grossesse, qui ou qu'est-ce qui a été votre source primaire d'information concernant la grossesse, le travail et l'accouchement?
(Laissez répondre puis cochez toutes les réponses qui s'appliquent)

- ☐ Ancienne grossesse
- ☐ Famille ou amis(ies)
- ☐ Chef spirituel
- ☐ Obstétricien/gynécologue
- ☐ Médecin de famille, omnipraticien
- ☐ Sage-femme
- ☐ Infirmier(ière)/infirmier(ière) clinicien(ne)
- ☐ Cours pendant la grossesse/cours prénataux
- ☐ Livres
- ☐ Télévision
- ☐ Internet
- ☐ Autre (*veuillez préciser*): _________________

---

13. * M Au cours de cette grossesse, un professionnel de la santé vous a-t-il offert des informations dans votre langue?

- ☐ Oui ____________ (*veuillez préciser*)
- ☐ Non

14. *Pendant CETTE grossesse, avant le travail et l'accouchement, aviez-vous suffisamment d'information concernant les sujets suivants ?

	Oui	*Non*	*Vous ne savez pas*
Changements physiques durant la grossesse	☐	☐	☐
Changements émotionnels durant la grossesse	☐	☐	☐
Premiers signes de travail	☐	☐	☐
Médicaments	☐	☐	☐
À quoi s'attendre durant le travail et l'accouchement	☐	☐	☐
Traitement de la douleur sans médicaments	☐	☐	☐
Tests médicaux nécessaires	☐	☐	☐
Nutrition durant la grossesse	☐	☐	☐
Votre propre santé et vote rétablissement après la naiss nce	☐	☐	☐
Vos changements d'humeurs	☐	☐	☐
Comment tenir votre bébé	☐	☐	☐
Comment repérer les problèmes de développement et de santé chez le bébé	☐	☐	☐
L'allaitement	☐	☐	☐
Lait maternisé/préparation pour nourrissons	☐	☐	☐
Qui contacter si vous avez es questions à propos de la santé de votre bébé	☐	☐	☐
Planification familiale/ contrôle de naissance	☐	☐	☐
Le VIH et autres maladies / infections transmises sexuellement?	☐	☐	☐

15. *Les professionnels de la santé vous ont-ils demandé comment vous comptez nourrir votre bébé?

☐ Oui
☐ Non
☐ Vous ne savez pas/ne vous souvenez pas
☐ S/O (pas de professionnel de la santé)

16. *Votre professionnel(s) de la santé vous a-t-il (ont-ils) demandé si vous aviez des préférences par rapport aux soins durant la grossesse, ou si vous aimeriez suivre une méthode ou une coutume particulière?

☐ Oui
☐ Non
☐ S/O (pas de professionnel de la santé)

La prochaine série de questions abordent les sujets du travail et de l'accouchement suite à votre dernière grossesse. Il y a 16 questions dans cette section.

17. *À combien de semaines de grossesse avez-vous accouché? ____________ (semaines)

☐ Vous ne savez pas/ne vous souvenez pas

18. *Vous avez accouché de combien de bébé(s)? ____________ (ex : un seul bébé, des jumeaux, etc.)

19. *Quel était le poids de votre (vos) bébé(s) à la naissance?

________ (kg) ________ (grammes)/ ________ (oz) ________(lbs)

________ (kg) ________ (grammes)/ ________ (oz) ________(lbs) ***(si plusieurs bébés)***

~~**20. *Où étiez-vous lorsque vous avez accouché?**~~
~~**(*Lisez tout puis cochez une réponse*)**~~

21. *Quel type de professionnel de la santé s'est le plus occupé de vous pendant la plupart de votre TRAVAIL?
(Laissez répondre puis cochez toutes les réponses qui s'appliquent)

- ☐ Obstétricien, gynécologue
- ☐ Médecin de famille, omnipraticien
- ☐ Sage-femme
- ☐ Infirmier(ière) ou infirmier(ière) clinicien(ne)
- ☐ Autre (*veuillez préciser*): ________________________
- ☐ Aucun
- ☐ Aucun, il n'y a pas eu de travail, il s'agissait d'une césarienne planifiée
- ☐ Ne sait pas

22. *Quel type de professionnel de la santé s'est le plus occupé de vos soins pendant votre ACCOUCHEMENT?
(Laissez répondre puis cochez toutes les réponses qui s'appliquent)

- ☐ Obstétricien, gynécologue
- ☐ Médecin de famille, omnipraticien
- ☐ Sage-femme
- ☐ Infirmier(ière) ou infirmier(ière) clinicien(ne)
- ☐ Autre (*veuillez préciser*): ________________________
- ☐ Aucun
- ☐ Ne sait pas

23. ***A-t-on employé une ou plus d'une des procédures ci-dessous lors du travail et de l'accouchement?**

	Oui	*Non*
Déclenchement artificiel du travail (déclencher les contractions)	☐	☐
Augmentation du travail (accélérer et augmenter l'intensité des contractions existantes)	☐	☐
Utilisation du forceps (outil en métal pour faire sortir le bébé)	☐	☐
Utilisation d'une ventouse (outil en caoutchouc pour faire sortir le bébé)	☐	☐
Césarienne	☐	☐
Épisiotomie (faire une incision pour élargir l'ouverture du vagin)	☐	☐
Anesthésie péridurale pour la douleur durant le travail	☐	☐
Anesthésie rachidienne (de la colonne vertébrale) pour une césarienne	☐	☐
Anesthésie générale	☐	☐
Autre *(veuillez préciser)*: ________________________	☐	☐

24. ***Y avait-il des complications sur le plan médical durant le travail et l'accouchement?**
(Tel que : déchirure périnéale, rupture utérine, infection, hémorragie post-partum, ou problèmes avec le bébé)

☐ Oui *(veuillez préciser)*: ________________________
☐ Non

Si la mère a accouché par voie vaginale, aller à la Q26

25. ***Si le bébé est né par méthode césarienne, quelle en était la raison principale?**
(Laissez répondre puis cochez une réponse)

☐ C'était prévu puisque le médecin l'a conseillé pour des raisons médicales
☐ C'était prévu, mais vous ne savez pas pourquoi
☐ C'était prévu puisque vous l'avez souhaité, ce n'était pas une raison médicale
☐ Ce n'était pas prévu, mais le travail durait trop longtemps
☐ Ce n'était pas prévu, mais le bébé était en danger
☐ Ce n'était pas prévu mais j'étais en danger
☐ Ce n'était pas prévu, et vous ne savez pas pourquoi on vous a fait une césarienne
☐ Autre *(veuillez préciser)*: ________________________
☐ S/O (accouchement par voie vaginale)

26. **Durant le travail, vous a-t-on permis de vous déplacer et de vous mettre dans une position confortable de votre choix?**
(Lisez à voix haute puis cochez une réponse)

☐ Oui, toujours
☐ Oui, parfois
☐ Oui, rarement
☐ Non, pour des raisons médicales
☐ Non, pour des raisons que vous ignorez,
☐ S/O, il n'y a pas eu de travail, il s'agissait d'une césarienne planifiée

27. Durant le travail, les professionnels de la santé vous ont-ils demandé comment vous vouliez gérer votre douleur ?

- ☐ Oui
- ☐ Non
- ☐ Vous ne savez pas/ne vous souvenez pas
- ☐ S/O, il n'y a pas eu de travail, il s'agissait d'une césarienne planifiée

---

28. Durant le travail, étiez-vous satisfaite de la manière dont les professionnels de la santé vous ont aidé à gérer la douleur?

- ☐ Oui
- ☐ Non
- ☐ Parfois
- ☐ S/O, il n'y a pas eu de travail, il s'agissait d'une césarienne planifiée

---

29. Durant votre travail, vous a-t-on permis d'avoir les membres de votre famille et/ou d'autres personnes de soutien de votre choix à vos côtés?

- ☐ Oui
- ☐ Non
- ☐ Parfois
- ☐ S/O, il n'y a pas eu de travail, il s'agissait d'une césarienne planifiée

---

30. *Aviez-vous un compagnon avec vous pendant le travail et l'accouchement?
(Lisez à voix haute puis cochez une réponse)

- ☐ Oui, toujours
- ☐ Oui, parfois
- ☐ Oui, rarement
- ☐ Non
- ☐ Vous ne savez pas/ne vous souvenez pas

---

31. *Si OUI, qui était-ce?
(S'il y en a plusieurs, veuillez les mentionner tous)

____________________________ (relation avec vous)
____________________________ (relation avec vous)
____________________________ (relation avec vous)

- ☐ S/O

---

32. *Votre professionnel de la santé vous a-t-il demandé si vous avez des préférences par rapport aux soins, ou si vous souhaiterez suivre une méthode ou coutume particulière durant le travail ou l'accouchement?

- ☐ Oui
- ☐ Non
- ☐ Non, parce que je le leur ai demandé avant qu'ils me le demandent

La prochaine série de questions abordent le sujet de votre période postpartum (le temps depuis la naissance de votre enfant). Il y a 14 questions dans cette section.

33. *Votre bébé avait-il besoin de soins spéciaux qu'il a reçus dans une salle séparée de la vôtre?
(Lisez à voix haute puis cochez toutes les réponses qui s'appliquent)

- ☐ Oui, à l'unité néonatale des soins intensifs
- ☐ Oui, à la pouponnière de soins spéciaux
- ☐ Oui, à la pouponnière
- ☐ Oui, (mais vous ne savez pas où/vous ne vous en souvenez pas)
- ☐ Non
- ☐ Vous ne savez pas/ne vous souvenez pas

~~34. Combien de temps êtes-vous restées à l'hôpital ou à la clinique après la naissance de votre bébé?~~

~~_______(heures) _______(jours) _______(semaines) ________(mois)~~

~~35. D'après vous, cette période était-elle très courte, très longue ou satisfaisante?~~

36. Les professionnels de la santé vous ont-ils demandé si vous aviez des préférences par rapport à votre nourriture pendant votre séjour à l'hôpital/à la maison de naissance (ex.: la température de la nourriture, nourriture préparée par rapport à vos croyances religieuses, végétarienne, ou autre type de nourriture)?

- ☐ Oui
- ☐ Non
- ☐ Vous ne savez pas/ne vous souvenez pas
- ☐ S/O (naissance à domicile)

37. *Les professionnels de la santé vous ont-ils demandé si vous avez des préférences par rapport aux soins, ou si vous aimerez suivre une méthode ou coutume particulière après l'accouchement?

- ☐ Oui
- ☐ Non
- ☐ Vous ne savez pas/ne vous souvenez pas

38. Dans la première heure après l'accouchement, vous a-t-on donné votre bébé pour que vous puissiez avoir un contact de peau-à-peau (votre peau contre la sienne) ?

- ☐ Oui
- ☐ Non. Si non, pourquoi pas: __________________________________

39. ***À quel moment le professionnel de soins de santé vous a-t-il aidé ou proposé de vous aider à commencer à allaiter?**
(Laissez répondre puis cochez une réponse)

- ☐ Dans l'heure suivant la naissance.
- ☐ Pas immédiatement, mais quand j'étais toujours à l'endroit où j'ai accouché (ex : hôpital, centre de naissance, maison)
- ☐ Un autre jour, lors d'une visite ou d'un rendez-vous
- ☐ Ils ont ni aidé ni proposé d'aider
- ☐ Vous ne savez pas/ne vous souvenez pas
- ☐ Je ne voulais pas allaiter mon enfant

---

40. ***Vos professionnels de la santé vous ont-ils informé au sujet des ressources en allaitement dans votre communauté?**

- ☐ Oui
- ☐ Non, mais vous n'aviez pas besoin d'être informée (*Passez à la Q42*)
- ☐ Non, mais vous vouliez être informée (*Passez à la Q42*)
- ☐ Vous ne savez pas/ne vous souvenez pas

~~**41.** ***Si OUI, vous êtes-vous servis de ces services de soutien à l'allaitement?**~~

~~**42.** ***Depuis l'accouchement avez-vous ou votre bébé consulté un professionnel de la santé pour une raison quelconque (soins routiniers compris) reliée à cette grossesse ou à cet accouchement?**~~

~~**43.** ***Si OUI, pourquoi?** ________________________~~

~~**44.** ***Si OUI, qui avez-vous consulté?**~~
~~***(Laissez répondre puis cochez toutes les réponses qui s'appliquent)***~~

~~**45.** ***Depuis l'accouchement, voulez-vous consulter un professionnel de la santé pour vous ou votre bébé sans en être capable?**~~

---

46. ~~*Si c'est votre cas, pour quelle(s) raison(s) étiez-vous incapable de voir un professionnel de la santé ?~~
~~(Laissez répondre puis cochez toutes les réponses qui s'appliquent)~~

La prochaine série de questions abordent votre expérience globale avec les soins de santé maternels au cours de votre dernière grossesse. Il y a 20 questions dans cette section.

47. Lorsque vous y pensez maintenant, y avait-il d'autre conseils/soutien/information que vous auriez voulu recevoir?

____
____
____

48. *Dans l'ensemble, lorsque vous rencontriez les professionnels de la santé, vous ont-ils bien accueilli?

a) Pendant la grossesse

☐ Toujours
☐ Parfois
☐ Rarement
☐ Jamais

b) Pendant le travail et l'accouchement

☐ Toujours
☐ Parfois
☐ Rarement
☐ Jamais

c) Après l'accouchement

- ☐ Toujours
- ☐ Parfois
- ☐ Rarement
- ☐ Jamais

---

49. *Dans l'ensemble, les professionnels de la santé étaient-ils respectueux?

a) Pendant la grossesse

- ☐ Toujours
- ☐ Parfois
- ☐ Rarement
- ☐ Jamais

b) Pendant le travail et l'accouchement

- ☐ Toujours
- ☐ Parfois
- ☐ Rarement
- ☐ Jamais

c) Après l'accouchement

- ☐ Toujours
- ☐ Parfois
- ☐ Rarement
- ☐ Jamais

---

50. *Dans l'ensemble, les professionnels de la santé étaient-ils serviables?

a) Pendant la grossesse

- ☐ Toujours
- ☐ Parfois
- ☐ Rarement
- ☐ Jamais

b) Pendant le travail et l'accouchement

- ☐ Toujours
- ☐ Parfois
- ☐ Rarement
- ☐ Jamais

c) Après l'accouchement

- ☐ Toujours
- ☐ Parfois
- ☐ Rarement
- ☐ Jamais

51. *Dans l'ensemble, je suis satisfaite des soins de santé que j'ai reçus

a) Pendant la grossesse

☐ Toujours
☐ Parfois
☐ Rarement
☐ Jamais

b) Pendant le travail et l'accouchement

☐ Toujours
☐ Parfois
☐ Rarement
☐ Jamais

c) Après l'accouchement

☐ Toujours
☐ Parfois
☐ Rarement
☐ Jamais

---

52. *Durant votre grossesse, votre travail, ou votre accouchement, les professionnels de la santé vous ont-ils demandé de faire quelque chose que vous n'aviez pas envie de faire?

☐ Oui
☐ Non
☐ Vous ne savez pas/ne vous souvenez pas

---

53. Si OUI, qu'est-ce que c'était?

____

☐ S/O

---

54. Les professionnels de la santé vous ont-ils demandé si vous préfériez avoir un professionnel de la santé femme ou homme?

a) Pendant la grossesse

☐ Toujours
☐ Parfois
☐ Rarement
☐ Jamais
Commentaires____________________________

b) Durant le travail et l'accouchement

☐ Toujours
☐ Parfois
☐ Rarement
☐ Jamais
Commentaires____________________________

c) Le premier jour après l'accouchement

☐ Toujours
☐ Parfois
☐ Rarement
☐ Jamais
Commentaires___________________________

---

55. *Avez-vous compris les renseignements fournis par les professionnels de la santé?

a) Pendant la grossesse
☐ Toujours
☐ Parfois
☐ Rarement
☐ Jamais
Commentaires___________________________

b) Pendant le travail et l'accouchement
☐ Toujours
☐ Parfois
☐ Rarement
☐ Jamais
Commentaires___________________________

c) Le premier jour après l'accouchement
☐ Toujours
☐ Parfois
☐ Rarement
☐ Jamais
Commentaires_______________________

---

56. * M Auriez-vous mieux compris les renseignements fournis par les professionnels de la santé s'ils avaient été offerts dans une autre langue?

☐ Oui, laquelle ______________________? (ex : votre langue maternelle)
☐ Non,
☐ Vous ne savez pas/ne vous souvenez pas

---

57. * M Les professionnels de la santé vous ont-ils offert un service d'interprète?

a) Pendant la grossesse
☐ Oui
☐ Non
☐ S/O

b) Durant le travail et l'accouchement
☐ Oui
☐ Non
☐ S/O

c) Le premier jour après l'accouchement
☐ Oui
☐ Non
☐ S/O

58. * [M] **Est-ce qu'il y avait quelqu'un avec vous qui parlait votre langue et qui pouvait interpréter pour vous?**

a) Pendant la grossesse

☐ Toujours
☐ Parfois
☐ Rarement
☐ Jamais
☐ S/O

b) Durant le travail et l'accouchement

☐ Toujours
☐ Parfois
☐ Rarement
☐ Jamais
☐ S/O

c) Le premier jour après l'accouchement

☐ Toujours
☐ Parfois
☐ Rarement
☐ Jamais
☐ S/O

---

59. * [M] **Si vous aviez accès à un interprète, c'était qui?**
(Lisez à voix haute puis cochez toutes les réponses qui s'appliquent)

a) Pendant la grossesse

☐ Mari/conjoint
☐ Autre membre de la famille/ami(e)
☐ Professionnel(le) de la santé
☐ Votre fils ou fille
☐ Interprète professionnel
☐ Un(e) autre patient(e) ou sa famille/ami(e)
☐ Autre *(veuillez préciser)*: ________________________
☐ S/O

b) Durant le travail et l'accouchement

☐ Mari/conjoint
☐ Autre membre de la famille/ami(e)
☐ Professionnel(le) de la santé
☐ Votre fils ou fille
☐ Interprète professionnel
☐ Un(e) autre patient(e) ou sa famille/ami(e)
☐ Autre *(veuillez préciser):* ________________________
☐ S/O

c) Le premier jour après l'accouchement

☐ Mari/conjoint
☐ Autre membre de la famille/ami(e)
☐ Professionnel(le) de la santé
☐ Votre fils ou fille
☐ Interprète professionnel
☐ Un(e) autre patient(e) ou sa famille/ami(e)
☐ Autre, *(veuillez préciser):* ________________________
☐ S/O

---

60. **M Etiez-vous satisfait de leur interprétation ?**

- ☐ Oui
- ☐ Non
- ☐ Vous ne savez pas/ne vous souvenez pas
- ☐ S/O

---

61. ***Durant le travail et l'accouchement, ou après l'accouchement, aviez-vous des préférences par rapport aux soins ou une coutume particulière que vous auriez aimé suivre mais que vous n'aviez pas pu parce que les professionnels de la santé ne vous l'ont pas permis?**

- ☐ Oui
- ☐ Non (*Passez à la Q64*)
- ☐ Vous ne savez pas/ne vous souvenez pas

---

62. **Si oui, quels étaient ces préférences?**

i) ____________
ii) ___________
iii) ___________

☐ S/O

---

63. **Si OUI, quelle était la raison que les professionnels de la santé vous ont donné pour laquelle ils ne vous ont pas permis de pratiquer ces préférences?**

i) ____________

ii) ___________

iii) ___________

☐ S/O

---

64. ***À votre avis, il y a-t-il quelque chose que les professionnels de la santé pourraient faire différemment ou mieux?**

a) Pendant la grossesse

- ☐ Oui (*Compléter Q65a*)
- ☐ Non
- ☐ Vous ne savez pas/ne vous souvenez pas

b) Durant le travail et l'accouchement

- ☐ Oui (*Compléter Q64b*)
- ☐ Non
- ☐ Vous ne savez pas/ne vous souvenez pas

c) Après l'accouchement

☐ Oui (*Compléter Q65c*)
☐ Non
☐ Vous ne savez pas/ne vous souvenez pas

---

65. Si OUI, veuillez décrire ce qui peut se faire différemment ou mieux, et par qui:

a) Pendant la grossesse

____

b) Durant le travail et l'accouchement

____

c) Après l'accouchement

____

---

66. *Pour ce qui est des soins, quels sont les points forts et les points faibles de votre grossesse, de votre accouchement, de la période après votre accouchement?

a) Points forts:

____

b) Points faibles:

____

Pensez à votre grossesse récente et dites-moi combien de fois les 11 déclarations suivantes sont justes.

---

67. *Les professionnels de la santé m'ont demandé si j'avais des questions à poser

☐ Toujours
☐ Parfois
☐ Rarement
☐ Jamais

---

68. Les professionnels de la santé étaient pressés

☐ Toujours
☐ Parfois
☐ Rarement
☐ Jamais

69. *Je sentais que les professionnels de la santé prenaient mes inquiétudes au sérieux

a) Pendant la grossesse

- ☐ Toujours
- ☐ Parfois
- ☐ Rarement
- ☐ Jamais
- ☐ S/O (pas de soins pendant la grossesse)

b) Durant le travail et l'accouchement

- ☐ Toujours
- ☐ Parfois
- ☐ Rarement
- ☐ Jamais
- ☐ S/O (pas de professionnel de la santé présent)

c) Après l'accouchement

- ☐ Toujours
- ☐ Parfois
- ☐ Rarement
- ☐ Jamais
- ☐ S/O (pas de professionnel de la santé présent)

---

70. J'ai attendu trop longtemps pour recevoir des soins

a) Pendant la grossesse

- ☐ Toujours
- ☐ Parfois
- ☐ Rarement
- ☐ Jamais
- ☐ S/O (pas de soins pendant la grossesse)

b) Durant le travail et l'accouchement

- ☐ Toujours
- ☐ Parfois
- ☐ Rarement
- ☐ Jamais
- ☐ S/O (pas de professionnel de la santé présent)

c) Après l'accouchement

- ☐ Toujours
- ☐ Parfois
- ☐ Rarement
- ☐ Jamais
- ☐ S/O (pas de professionnel de la santé présent)

71. ***Les professionnels de la santé me tenaient informé sur ce qu'il se passait**

a) Pendant la grossesse

- ☐ Toujours
- ☐ Parfois
- ☐ Rarement
- ☐ Jamais
- ☐ S/O (pas de soins pendant la grossesse)

b) Durant le travail et l'accouchement

- ☐ Toujours
- ☐ Parfois
- ☐ Rarement
- ☐ Jamais
- ☐ S/O (pas de professionnel de la santé présent)

c) Après l'accouchement

- ☐ Toujours
- ☐ Parfois
- ☐ Rarement
- ☐ Jamais
- ☐ S/O (pas de professionnel de la santé présent)

---

72. ***Je me sentais à l'aise quand venait le temps de poser des questions à propos de choses que je ne comprenais pas.**

a) Pendant la grossesse

- ☐ Toujours
- ☐ Parfois
- ☐ Rarement
- ☐ Jamais
- ☐ S/O (pas de soins pendant la grossesse)

b) Durant le travail et l'accouchement

- ☐ Toujours
- ☐ Parfois
- ☐ Rarement
- ☐ Jamais
- ☐ S/O (pas de professionnel de la santé présent)

c) Après l'accouchement

- ☐ Toujours
- ☐ Parfois
- ☐ Rarement
- ☐ Jamais
- ☐ S/O (pas de professionnel de la santé présent)

---

73. ***Les professionnels de la santé prenaient des décisions sans tenir compte de mes souhaits**

a) Pendant la grossesse

- ☐ Toujours
- ☐ Parfois
- ☐ Rarement
- ☐ Jamais
- ☐ S/O (pas de soins pendant la grossesse)

b) Durant le travail et l'accouchement

- ☐ Toujours
- ☐ Parfois
- ☐ Rarement
- ☐ Jamais
- ☐ S/O (pas de professionnel de la santé présent)

c) Après l'accouchement

- ☐ Toujours
- ☐ Parfois
- ☐ Rarement
- ☐ Jamais
- ☐ S/O (pas de professionnel de la santé présent)

---

74. ***Les professionnels de la santé étaient très encourageants et rassurants**

a) Pendant la grossesse

- ☐ Toujours
- ☐ Parfois
- ☐ Rarement
- ☐ Jamais
- ☐ S/O (pas de soins pendant la grossesse)

b) Durant le travail et l'accouchement

- ☐ Toujours
- ☐ Parfois
- ☐ Rarement
- ☐ Jamais
- ☐ S/O (pas de professionnel de la santé présent)

c) Après l'accouchement

- ☐ Toujours
- ☐ Parfois
- ☐ Rarement
- ☐ Jamais
- ☐ S/O (pas de professionnel de la santé présent)

75. *Les professionnels de la santé ont-ils passé assez de temps à fournir des explications?

a) Pendant la grossesse

- ☐ Toujours
- ☐ Parfois
- ☐ Rarement
- ☐ Jamais
- ☐ S/O (pas de soins pendant la grossesse)

b) Durant le travail et l'accouchement

- ☐ Toujours
- ☐ Parfois
- ☐ Rarement
- ☐ Jamais
- ☐ S/O (pas de professionnel de la santé présent)

c) Après l'accouchement

- ☐ Toujours
- ☐ Parfois
- ☐ Rarement
- ☐ Jamais
- ☐ S/O (pas de professionnel de la santé présent)

---

76. *Dans l'ensemble, avez-vous l'impression d'avoir reçu un traitement différent de la part des professionnels de la santé? (Ex.: à cause de votre langue ou accent, culture, origine ou couleur de peau, religion, statut d'immigration, ou de votre statut d'assurance médicale)

- ☐ Toujours (*veuillez préciser pourquoi à la Q77*)
- ☐ Parfois (*veuillez préciser pourquoi à la Q77*)
- ☐ Rarement (*veuillez préciser pourquoi à la Q77*)
- ☐ Jamais (*Passer à la Q78*)

---

77. *Si OUI, quelle en était la (ou les) raison(s) à votre avis?
(Laissez répondre puis cochez toutes les réponses qui s'appliquent)

- ☐ Langue ou accent
- ☐ Culture
- ☐ Origine
- ☐ Couleur de la peau
- ☐ Religion
- ☐ Statut d'immigration
- ☐ Statut de l'assurance médicale
- ☐ Autre raison (*veuillez préciser*): __________
- ☐ S/O

Les questions suivantes abordent votre HISTORIQUE OBSTÉTRIQUE. Il y a 8 questions dans cette section.

78. *Combien de fois étiez-vous enceintes en tout (y compris cette grossesse) ? _______

~~**79. *Combien de vos grossesses ont été interrompues à cause d'une fausse couche? _______**~~

~~***Assurez-vous d'être seules lorsque vous posez cette question***~~

~~**80.*Combien de vos grossesses ont été interrompues? _______**~~

~~**81. *Combien de mort-nés avez-vous eu? ________**~~

82. *Combien de vos enfants sont nés avant d'avoir compléter les 37 semaines de gestation ? _______

- ☐ S/O

83. *Combien de vos enfants sont nés après 37 semaines? ________

- ☐ S/O

84.*Aviez-vous eu des complications médicales pendant votre (vos) grossesse(s) précédente(s)?

- ☐ Oui,
- ☐ Non *(Passez à la Q86)*
- ☐ S/O **(***Passez à la Q86)*

85. * Si vous avez eu des complications médicales pendant votre (vos) grossesse(s) précédente(s) c'était lesquels? (*Laissez répondre puis cochez toutes les réponses qui s'appliquent*)

- ☐ Césarienne
- ☐ Anémie
- ☐ Hypertension artérielle
- ☐ Pre-éclampsie (hypertension artérielle gravidique)
- ☐ Travail prématuré
- ☐ Thrombose veineuse profonde
- ☐ Diabète de grossesse
- ☐ Placenta praevia
- ☐ Décollement placentaire
- ☐ Infection urinaire
- ☐ Douleurs sévères au dos
- ☐ Rupture prématurée des membranes
- ☐ Dépression
- ☐ Autre *(veuillez préciser)*________________________
- ☐ Ne sait pas
- ☐ S/O

Les dernières questions qu'on vous pose sont sur vos données démographiques. Il y a 27 questions.

86. *Quel est votre état matrimonial?

- ☐ Mariée
- ☐ Union libre
- ☐ Veuve
- ☐ Séparée
- ☐ Divorcée
- ☐ Célibataire

87. *Avec qui habitez-vous?

	Oui	*Non*
Partenaire/mari (homme)	☐	☐
Partenaire (femme)	☐	☐
Votre mère ou père	☐	☐
Votre frère(s) ou soeur(s)	☐	☐
La mère ou le père de votre partenaire	☐	☐
Frère(s) ou soeur(s) de votre partenaire	☐	☐
Ami(s)	☐	☐
Enfant (autre que le nouveau-né)	☐	☐
Autre *(veuillez préciser)*_____________________	☐	☐
Personne, j'habite seule avec mon bébé	☐	☐
Personne, j'habite seule	☐	☐

88. ***Combien de vos enfants habitent avec vous (y compris votre nouveau bébé)? ____________**

89. *** M Combien de vos enfants sont nés dans ce pays (y compris votre nouveau bébé)? ____________**

90. ***Quelle est votre date de naissance? ~~_______( )~~ ____________________(mois) ____________(année)**

91. *** M Quel est le pays natal de votre mère? ____________**

92. *** M Quel est le pays natal de votre père? ____________**

Les questions suivantes sont plus détaillées et portent sur votre immigration. Ces informations nous intéressent parce que nous voulons en apprendre plus sur l'expérience des femmes migrantes dans ce pays. Toute information fournie restera confidentielle, et aucune information ne sera donnée à l'office de l'immigration. Vos réponses à ces questions n'affecteront pas votre demande d'immigration si votre demande de statut de réfugié, de résidence permanente, ou de citoyenneté est en cours.

93. *** M Quel est votre statut d'immigration actuel ?**
(Veuillez cocher une des réponses suivantes)

- ☐ Immigrante (résidente permanente)
- ☐ Réfugiée
- ☐ Demandeur de statut de réfugiée / Demandeur d'asile
- ☐ Travailleur temporaire/aides familiaux résidents
- ☐ Résident temporaire
- ☐ Étudiante
- ☐ Visiteur
- ☐ Aucun statut
- ☐ Sans papiers
- ☐ Citoyenne Canadienne
- ☐ Autre *(veuillez préciser)*________________________

94. **M Depuis combien de temps avez-vous ce statut?**

_______(jours) _______(semaines) ________(mois) ______ (années)

95. **M Avez-vous changé de statut d'immigration depuis votre arrivée?**

- ☐ Oui
- ☐ Non *(Passez à la Q97)*

96. *** M Si votre statut d'immigration a changé, quel était votre statut auparavant ?**

- ☐ Immigrante (résidente permanente)
- ☐ Réfugiée
- ☐ Demandeur de statut de réfugiée / Demandeur d'asile
- ☐ Travailleur temporaire/ aides familiaux résidents
- ☐ Résident temporaire
- ☐ Étudiante
- ☐ Visiteur
- ☐ Aucun statut
- ☐ Sans papiers
- ☐ Autre, (*veuillez préciser*) _________________
- ☐ S/O (pas changé de statut)

---

97. **M Avez-vous déjà eu le statut de réfugié?**

- ☐ Oui
- ☐ Non
- ☐ Vous ne savez pas/ne vous souvenez pas

---

98. **M Avez-vous déjà vécu dans un centre de détention de l'immigration?**

- ☐ Oui
- ☐ Non (*Passez à Q101*)

---

99. **M Si OUI, pendant combien de temps?**

_______(jours) _______(semaines) ________(mois) ______ (années)

- ☐ S/O

---

100. *** M Si OUI, avez-vous vécu dans un centre de détention de l'Immigration pendant cette grossesse?**

- ☐ Oui
- ☐ Non
- ☐ S/O

---

101. ***Qui paie pour vos soins de santé?**

	Oui	*Non*	*Vous ne savez pas*
Assurance maladie publique (RAMQ)	☐	☐	☐
Assurance maladie privée	☐	☐	☐
Assurance gouvernementale pour les réfugiés et les demandeurs d'asile	☐	☐	☐
Vous payez pour vos services de santé	☐	☐	☐

102. ***Quel est le plus haut niveau d'éducation que vous avez complété?**

- ☐ École primaire
- ☐ École secondaire
- ☐ Diplôme post-secondaire (ex: diplôme d'études professionnelles, cégep, diplôme de premier cycle)
- ☐ Diplôme de deuxième cycle (ex: Maitrise, Doctorat)
- ☐ Aucun

---

103. **MAvez-vous l'autorisation légale de travailler dans ce pays?**

- ☐ Oui
- ☐ Non
- ☐ Vous ne savez pas/ne vous souvenez pas

---

104. ***Quel était votre dernier emploi rémunéré avant la naissance de votre bébé? (e.g., médecin, institutrice, opérateur de saisie de données, personnel infirmiers, gouvernante, exploitante agricole, conductrice de machine à teinture de fibres textiles, nettoyeuse d'hôtel, centre d'appel)**

(*veuillez préciser*):____________________

- ☐ S/O (Vous n'avez pas travaillé)

~~**105. *Êtes-vous retournée au travail depuis la naissance de votre bébé?**~~

~~**106. *Si OUI, quel est votre emploi actuel? (e.g., médecin, institutrice, opérateur de saisie de données, personnel infirmiers, gouvernante, exploitante agricole, conductrice de machine à teinture de fibres textiles, nettoyeuse d'hôtel, centre d'appel)**~~

---

107. ***D'après vous, dans quel groupe se situe le revenu total de votre maisonnée (avant taxes/impôts) ?** ***(Insérer entre les parenthèses les valeurs qui s'appliquent à la situation locale et lire les choix à voix haute.)***

- ☐ <$11,000____
- ☐ __$11,000____ à _$20,999____
- ☐ __$21,000____ à _$40,999____
- ☐ __$41,000___ à __$60,999____
- ☐ __$61,000____ à _$80,999____
- ☐ ≥ _$81,000___

---

108. ***Combien de personnes vivent de ce revenu (y compris le nouveau-né)?** ______________

109. ***Quelle(s) langue(s) parlez-vous le plus souvent à la maison?**

____

110. *** M Quel est votre niveau de connaissance de la langue officielle de ce pays ?**

Anglais:

	Couramment	*Bon*	*Avec difficulté*	*Pas du tout*
Parlé	☐	☐	☐	☐
Lu	☐	☐	☐	☐
Écrit	☐	☐	☐	☐
Compris	☐	☐	☐	☐

111. **M Quel est votre niveau de connaissance de la langue officielle de ce pays**

Français:

	Couramment	*Bon*	*Avec difficulté*	*Pas du tout*
Parlé	☐	☐	☐	☐
Lu	☐	☐	☐	☐
Écrit	☐	☐	☐	☐
Compris	☐	☐	☐	☐

112. ***Ceci conclut notre entrevue. Avez-vous autre chose à dire à propos des sujets abordés ? Avez-vous d'autres commentaires, ou autre chose à ajouter?**
