# Supplementary material for: Migrant-friendly maternity care in Montreal, Canada: A cross-sectional study on migrant women’s care perspectives
Source: PLoS One. 2025 Aug 21;20(8):e0330830. doi: 10.1371/journal.pone.0330830 (PMC12370051; doi:10.1371/journal.pone.0330830)
Supplement: S5 Appendix — (PDF) [file pone.0330830.s005.pdf]

प्रवासी मित्रवत मातृत्व देखभाल(माय्ग्रंट फ्रेंडली मैटरनिटी केयर) प्रश्नावली

इंटरव्यूवर के निर्देशन

1) इंटरव्यू के दौरान कृपया हर प्रश्न को धीरे से और ध्यान से पढ़िए। प्रश्नों के नीचे दिए निर्देशों का पालन करें।

- जिन प्रश्नों के निर्देशन है – *(कृपया संपूर्ण विकल्प जोर से पढ़ें फिर सभी योग्य लागु जवाबोंको टिक करें)*, उन प्रशोणों के हर एक जवाब को एक एक करके पढ़ें और माँ को हाँ या ना में जवाब करने दे;
- जिन प्रश्नों के निर्देशन है – *(प्रश्न का उत्तर माँ को करने दे और कृपया सभी योग्य लागु जवाबों को टिक करें)*, उन प्रश्नों के लिए उत्तारोंकी यदि ना पढ़ें. माँ को प्रश्न का उत्तर स्वयं करनें दें. यादि माँ को जवाब देने नहीं आ रहा है, तो लिखे गए उत्तरों से २ या ३ उत्तरे पढें|

2) विकल्पों को उत्तर ना करने और दुसरे प्रश्न पर जाने के बारे में निर्देशों का पालन करें |

उद्धरण के लिए:
प्रश्न नंबर 10 में माँ को पूछा गया है कि क्या वे स्वास्थ्य देखभाल से जुडी सेवाओं का उपयोग करना पसंद करती परंतु उनका उपयोग नहीं किया?इस प्रकार के प्रश्नों के लिए अगला प्रश्न (प्रश्न-11: यदि किसी कारन उपलब्ध सेवाओं द्वारा आयोजित परवरिश आप प्राप्त नहीं करसके , हम उन कारणों के बारे में जानना चाहेंगे) तभी पूछें अगर माँ का जवाब होगा 'हाँ'|

3) यदि कोई प्रश्न वक्त जे जुड़ा है (जैसे प्रश्न नंबर 2, 6), तो माँ को सबसे बेहतर अनुमान देने प्रेरित करिये|

4) यदि आप निश्चित नहीं है कि किस जवाब को टिक करना चाहिए (जैसे कि मेडिकल कोम्प्लिकेशन या कोई प्रक्रिया), तो “अन्य” प्रक्रिया या “अन्य” मेडिकल कोम्प्लिकेशन के विकल्प को टिक करें और उसके लिए स्पष्टीकरण लिखे |

5) जिन शब्दों का अर्थ माँ को समझ नहीं आता है, कृपया निचे दिए गए स्पष्टीकरणों का इस्तमाल करें:

- **परिवार नियोजन**: माँ बनने के लिए योजना बनाना, जैसे निर्णय लेना कि कोंट्रासेपशन का इस्तेमाल कब करना है और कब नहीं करना है
- **एनेस्थीसिया**:शरीर के भागको सुन्न करने या बेहोश करने के लिए सुई लगाना
- **नियोनेटल युनिट**: नवजात गहन चिकित्सा इकाई यानि जहाँ माँ और बच्चे को विशेष देखभाल के लिए "स्वास्थ्य देखभाल" पेशेवर या अन्य चिकित्सा पेशेवर के कहने से अलग रखा जाता है
- **मिसकेरेज**: गर्भावस्था को 20 हफ्ते होने से पहले बच्चा गिरजाना
- **गर्भावस्था समाप्त करना**: पेट में बच्चा बड़ा होने से पहले उसे गिरा देना या पेट से निकाल देना (अबोरशन)
- **इम्मिग्रेशन स्टेटस** (प्रश्न 93): स्टेटस जिस दिन से वे इस देश में आए, ना कि जब इम्मिग्रेशन कि कगजद मिले थे

- **इम्मिग्रेशन डीटेनशन/हिरासत केंद्र**: जवाब देने प्रोत्साहित करने: क्या आपको कभी इम्मिग्रेशन अधिकारियों ने हिरासत में बंद रखा था? क्या आपको कभी इम्मिग्रेशन के कारणों से गिरफ्तार किया था?
- **इनकम/आय**: घर के सारे सदस्यों कि कमायि (जैसे कि बहन, पती, माँ) बीना टैक्स के
- यह कमाई घर के कितने सदस्यों पर खर्च होती है?
- **प्रसव**: संकुचन(दर्द) का शुरू होने से लेकर बच्चे के जन्म तक

NOTES: (1) Questions marked with * (n=86) were identified (during a Delphi consensus process with international perinatal health research experts) as a minimum set of questions for use in international comparisons; (2) Questions marked with M are those only relevant for migrant women or identified as recommended migration indicators to capture in analyses of perinatal health (see Gagnon AJ, Zimbeck M, Zeitlin J. Migration and Perinatal Health Surveillance: An International Delphi Survey. *European Journal of Obstetrics & Gynecology and Reproductive Biology*. 2010;149(1), 37-43).

साक्षात्कार (इंटरव्यू) का प्रारंभ: माताओं के लिए परियोजना (प्रोजैक्ट) का सारांश

नमस्ते, मैं एक अनुसंधान (रिसर्च) टीम के साथ काम करती हूँ | यह टीम हाल ही में परदेस आए महिलाओं के मातृत्व (मैटरनिटी) से जुड़े अनुभवों को समझना चाहते है | इसलिए अपनी टीम की ओर से, मैं आपके गर्भावस्था/ प्रेग्नन्सी (यानि वो स्थिति जिसमें आपके पेट में बच्चा पलता है), प्रसव (यानि बच्चे को जन्म देते समय होने वाली शारीरिक घटनाए), बच्चे का जन्म और अन्य समग्र मातृत्व (मैटरनिटी) से जुड़े अनुभवों के बारेमें पूछना चाहूंगी | इसके आलावा आपके प्रसूति (डिलीवरी) और प्रवास (इमीग्रेशन) के इतिहास के बारे में भी मैं पूछना चाहूंगी | इस बीच, यदि आपको कोई बात या शब्द का अर्थ समझ न आया हो, तो आप मुझे बिना किसी झिझक के प्रश्नों को दोहराने, स्पष्ट करने, या प्रश्न को दोबारा समझाने के लिए कह सकते है. मैं फिरसे दोहराना चाहूंगी की आपने बताई सभी जानकारी हमारे पास निजी रहेगी | आप किसी भी समय इस अध्ययन (अभ्यास) से भागीदारी वापस ले सकते हैं | यदि आप किसी प्रश्न का उत्तर देने आरामदेह नहीं महसूस करते है, तो उस प्रश्न को छोड़ सकते है |

यदि आपको किसी समय कोई भी प्रश्न हो, तो कृपया मुझे बताए | क्या आप अभी कोई प्रश्न पूछना चाहती है ? ठीक है, तो फिर हम शुरू करते है!

MFMCQ Hindi Version		अभ्यास आईडी नंबर:	
प्रारंभ का समय:		साक्षात्कारकर्ता (इंटरव्यूवर) का नाम:	
अंत का समय:		साक्षात्कार (इंटरव्यू) की तारीख:	

1. ***M आपका जन्म किस देश मैं हुआ था (जन्मभूमि)?**

____

2. ***M आपको इस देश में रहते कितने साल हुए है?**
(यदि माइग्रेट होने से पहले माँ का इस देश में आना-जाना रहा है, तो यहाँ हुए माँ के संपूर्ण सालों के रहने की गीनती हमे बताईये)
_______ (दिन) _______ (हफ्ते) _______ (महीने)_______ (साल)

प्रथम प्रश्नोंका समुह आपके इस देश में सबसे हाल ही में हुए गर्भावस्था/प्रेग्नन्सी (स्थिति जिसमें आपके पेट में बच्चा पलता है) के बारे में है | इस अनुभाग में 14 प्रश्न है |

3. **M क्या आप इस देश में गर्भवती/ प्रेग्नंट पेट में बच्चा लेकर आए थे?**

- ☐ हाँ, (यदि आपका जवाब हाँ है, तो हमे बताइए आपका कौनसा हफ्ता चल रहा था?) _______
- ☐ नहीं
- ☐ पता नहीं

4. ***क्या आप इस गर्भावस्था में, किसी "स्वास्थ्य देखभाल पेशेवर (हेल्थ केयर प्रोफेशनल) से मिलते थे? (जैसे की डॉक्टर, नर्स, आया, दाई)?**

- ☐ हाँ, ______________________ (कौन से देश में)
- ☐ नहीं *(अगर आपका जवाब नहीं है, तो प्रश्न नंबर 8 से उत्तर लिखना शुरू करिए)*

5. ***इस गर्भावस्था में आपकी परवरिश इस देश में किसने मदद की?**
***(इस प्रश्न का जवाब माँ को करने दे और योग्य लागु होने वाले जवाबों को 'टिक' करें* / यदि जरुरत पड़ी तो माँ को जवाब देने में मदद करें)**

- ☐ परिवार के डॉक्टर
- ☐ प्रसूति या स्त्री रोग के डॉक्टर
- ☐ दाई
- ☐ नर्स / नर्स प्रेक्टिशनर
- ☐ अन्य व्यक्ति *(कृपया स्पष्ट करें)*:_________
- ☐ आपको प्रश्न लागु नहीं करता

---

6. ***इस गर्भावस्था के दौरान जब पहली बार आप स्वास्थ्य देखभाल पेशेवर से मिले, आपका कौनसा हफ्ता चाल रहा था?**
__________ (हफ्तें); इस देश में_______________ (हफ्तें)
(यदि आपकी केवल गर्भावस्था के परीक्षण सम्बंधित नियुक्ति (अपॉइंटमेंट) रही हो, तो उसे गिनती में शामिल न करें)

- ☐ आपको प्रश्न लागु नहीं करता (इस गर्भावस्था के दौरान आप किसी स्वास्थ्य देखभाल पेशेवर से नहीं मिले थे)

---

7. ***इस गर्भावस्था में आप किसी डाक्टर, नर्स या दाई से कितनी बार मिले थे?** _________

- ☐ आपको प्रश्न लागु नहीं करता (इस गर्भावस्था के दौरान आप किसी स्वास्थ्य देखभाल पेशेवर से नहीं मिले थे)

---

8. ***क्या इस गर्भावस्था के दौरान आपको किसी प्रकार की चिकित्सा जटिलताऐं (मेडिकल कॉम्प्लिकेशन) घटित हुई थी?**

- ☐ हाँ ***(इस प्रश्न का जवाब माँ को करने दे और सभी योग्य लागु होने वाले जवाबों को 'टिक' करें)***
  - ☐ रक्ताल्पता (खून में हिमोग्लोबिन की कमी)
  - ☐ उच्च रक्तचाप (बिपि)
  - ☐ प्री-एक्लाम्पसिया (जेस्तेशनल हयपर्तेन्श्न)
  - ☐ अपरिपक्व प्रसव (सामान्य समय से पूर्व होने वाला प्रसव/ लेबर)
  - ☐ डीप वेन थ्रोम्बोसिस (पैरोंके गहरी शिरा में होने वाली खून की गांठ)
  - ☐ जेस्तेशनल डायबेटीस (खून में होनेवाली शक़्कर की अधिकता या कमी)
  - ☐ प्लासेन्टा पराविया (बच्चे की नाल, गर्भाशय के अनुचित भाग से जुड़ना)
  - ☐ प्लेसेंटल अबरपशन (बच्चे के जन्म समय, नाल का अनुचित ढंग से टूटना)
  - ☐ युरीनरी ट्रेक इन्फेक्शन (मूत्र मार्ग रोग)
  - ☐ तीव्र पीठ दर्द
  - ☐ प्री-टर्म रप्चर ऑफ मेम्ब्रेन (पानी के थैली का समय से पहले टूट जाना)
  - ☐ डिप्रेशन (उदासी महसूस करना)
  - ☐ आपसे या पेट में पल रहे बच्चे से जुड़ी अन्य चिकित्सा जटिलताऐं *(कृपया स्पष्ट करें)* __________________
- ☐ नहीं, आपको गर्भावस्थामें में किसी प्रकार की चिकित्सा जटिलताऐं (मेडिकल कॉम्प्लिकेशन) घटित नहीं हुई थी

9. **इस गर्भावस्था के दौरान निम्नलिखित सेवाओं में से आपने किन सेवाओं का उपयोग किया है ?**

	हाँ	नहीं
गर्भावस्था या बच्चे के जन्म से जुडी जानकारी देने वाली कक्षाएं	☐	☐
"स्वास्थ्य देखभाल" पेशेवर के साथ नियुक्ति (समय से पहले मिलने की तारीख पक्की करना - अपॉइंटमेंट लेना)	☐	☐
खाद्य बैंक (जगह जहाँ आपको राशन मिलता है)	☐	☐
घर ढूंड ने की सहायता	☐	☐
पारंपारीक दवाइ / रस्में	☐	☐
परिवार से जुडी सेवाए (जैसे बच्चे की देखभाल, माता-पिता की भूमिका योग्य ढंग से पालन करने की सिख देनेवाली कक्षाए)	☐	☐
गर्भावस्था के दौरान होने वाले मेडिकल परीक्षण (जैसे पूर्ण शारीरिक परीक्षण, रक्त परीक्षण, पैप टेस्ट)	☐	☐
बच्चे के जन्म से जुड़े दोष/ कमी का पता लगाने के लिए स्क्रीनिंग (जैसे, डाउन सिंड्रोम)	☐	☐
अल्ट्रासाउंड स्कैन	☐	☐
समर्थन/ सहारा देनेवाली सेवाए (जैसे मानसिक स्वास्थ्य सेवाएं)	☐	☐
अन्य सेवाएँ (कृपया स्पष्ट करें) ____________________	☐	☐

10. ***इस गर्भावस्था के दौरान, क्या इन उपर्युक्त सेवाओं का आप उपयोग करना पसंद करते परंतु आपने उपयोग नहीं किया?** ***(कृपया संपूर्ण विकल्प पहले जोर से पढ़े और सभी योग्य लागु जवाबों को 'टिक' करें)***

☐ गर्भावस्था या बच्चे के जन्म से जुडी जानकारी देने वाली कक्षाएं
☐ स्वास्थ्य देखभाल पेशेवर के साथ नियुक्ति (समय से पाहिले मिलने की तारीख पक्की करना - अपॉइंटमेंट लेना)
☐ खाद्य बैंक
☐ घर धुंद ने की सहायता
☐ पारंपरिक दवाइ / रस्में
☐ परिवार से जुडी सेवाए (जैसे बच्चे की देखभाल, परामर्श, माता-पिताकी भूमिका योग्य शैली से पालन करनेके पाठ्यक्रम)
☐ गर्भावस्था के दौरान होने वाले मेडिकल परीक्षण (जैसे पूर्ण शारीरिक, रक्त परीक्षण, ग्रीवा परीक्षा / पैप परीक्षण)
☐ बच्चे के जन्म से जुड़े दोष/ कमी का पता लगाने के लिए स्क्रीनिंग (जैसे, डाउन सिंड्रोम)
☐ अल्ट्रासाउंड स्कैन
☐ समर्थन/ सहारा देनेवाली सेवाए (जैसे मानसिक स्वास्थ्य सेवाएं)
☐ अन्य सेवाएँ *(कृपया स्पष्ट करें):* ____________________
☐ यदि आपने गर्भावस्था के दौरान मन चाही स्वास्थ्य देखभाल सेवओं का उपयोग किया था, तो कृपया प्रश्न नंबर 12 पर जाइए

11. ***यदि किसी कारण, उपलब्ध सेवाओं द्वारा आयोजीत परवरिश आप प्राप्त नहीं कर सके, हम उन कारणों के बारेमें जानना चाहेंगे? (इस प्रश्न का जवाब माँ को करने दे और सभी योग्य लागु होनेवाले जवाबों को 'टिक' करें)**

- ☐ आपके इलाके में सेवाएँ उपलभ्ध नहीं थी
- ☐ सेवाएं पहले से ही भरी थी
- ☐ सेवाओं की पेशकश की जानकारी आपको पता नहीं थी
- ☐ आपको पता नहीं था कि यह सेवाएं आपके लिए उपलब्ध थी
- ☐ यह सेवाएं आपके लिए उपलब्ध नहीं थी
- ☐ इन सेवाओं की पेशकश की जगह आपको पता नहीं थी
- ☐ आपके इमीग्रेशन के कागजाद प्रभावित होगे ईस बात से आप डर गए थे
- ☐ आपकी नियुक्ति (अपॉइंटमेंट) स्वस्थ देखभाल पेशेवर द्वारा रद्द करदी गयी थी
- ☐ बाल देखभाल सेवा उपलब्ध नहीं थी
- ☐ भाषा की बाधा आपको सेवा उपलब्ध करने से रोक रही थी
- ☐ आपको आने-जाने के लिए वाहन उपलब्ध नहीं था
- ☐ आपकी पैसों से सम्बंधित करणे थी (जैसे पैसों कि कमी)
- ☐ आप काम कर रहे थे
- ☐ आपके पास समय नहीं था
- ☐ आपको घर पर रहने की जरुरत थी
- ☐ आपको चिकित्सा (मेडिकल) परीक्षण या परीक्षाओं का डर लगता है
- ☐ आपको परिवार या दोस्तों की सलाह और सहायता मिली थी
- ☐ आपको दी गयी सेवाएँ आपके उम्मिदोंनुसार नहीं थी
- ☐ आपको स्वास्थ्य देखभाल योजनाओं के कार्य समझने या उसका उपयोग करनेमें कठिनाई हुई
- ☐ आपको शर्मिंदगी महसूस हुई
- ☐ प्रशासनिक कारणों से (उदाहरण के लिए- आपका स्वास्थ्य बीमा नहीं था)
- ☐ अन्य कारने *(कृपया स्पष्ट करें)* ________________
- ☐ आपको प्रश्न लागु नहीं करता

12. *इस गर्भावस्था के दौरान, आपको बच्चे के जनम और प्रसव पीड़ा सम्बंधित जानकारी देनेवाले सबसे महत्वपूर्ण सूत्र (जैसे लोग, साधन, अनुभव) कौन थे?
(आपको जानकारी कैसे, कहाँ से और किस से मिली?) (इस प्रश्न का जवाब माँ को करने दे और सभी योग्य लागु होनेवाले जवाबों को 'टिक' करें)

- ☐ पिछली गर्भावस्था
- ☐ परिवार के सदस्य या मित्र
- ☐ साधू-संत
- ☐ प्रसूति या स्त्री रोग डाक्टर,
- ☐ परिवार के डाक्टर
- ☐ दाई
- ☐ नर्स / नर्स प्रेक्टिशनर
- ☐ गर्भावस्था / बच्चे के जन्म सम्बंधित जानकारी देनेवाली कक्षाएं
- ☐ पुस्तकें
- ☐ टेलीविजन
- ☐ इंटरनेट
- ☐ अन्य व्यक्ति *(कृपया स्पष्ट करें)* : _________________

13. *M क्या इस गर्भावस्था के दौरान आपको स्वास्थ्य देखभाल पेशेवरोंने इस देश में आपकी भाषा में जानकारी दी गई?

- ☐ हाँ, *(कृपया स्पष्ट करें)* : _________________
- ☐ नहीं

14.*इस गर्भावस्थामें, क्या आपको प्रसव और बच्चे को जन्म देने से पहले निम्नलिखित विषयों के बारे में पर्याप्त जानकारी थी?

गर्भावस्था के दौरान होनेवाले शारीरिक परिवर्तन	हाँ	नहीं	पता नहीं
गर्भावस्था के दौरान होनेवाले भावनात्मक परिवर्तन	☐	☐	☐
प्रसव की शुरुआत के संकेत	☐	☐	☐
दवाएँ	☐	☐	☐
प्रसव पीड़ा और बच्चे के जन्म के दौरान क्या उम्मीद करनी है	☐	☐	☐
दवाइयों के अलावा दर्द कम करनेवाली उपलब्ध चिकित्साऐ	☐	☐	☐
आवश्यक चिकित्सा (मेडिकल) परीक्षण	☐	☐	☐
गर्भावस्था के दौरान पोषण	☐	☐	☐
जन्म के बाद होनेवाली स्वास्थ्य वसूली	☐	☐	☐
गर्भावस्था के दौरान होनेवाली मनोदशा में बदलाव	☐	☐	☐
बच्चे को उचित ढंगसे संभालना	☐	☐	☐
बच्चे के स्वास्थ्य और विकास सम्बंधित समस्याओं को पहचानना	☐	☐	☐
स्तनपान (छाती से दूध पिलाना)	☐	☐	☐
फॉर्मूला फीडिंग	☐	☐	☐
अपने बारे में या आपके बच्चे के स्वास्थ्य के लिए प्रश्न पूछना हो, तो उपयुक्त व्यक्ति से संपर्क करेने की जानकारी?	☐	☐	☐
परिवार नियोजन / कोंट्रासेप्शन	☐	☐	☐
एचआईवी और अन्य इस प्रकार के रोगों की जानकारी?	☐	☐	☐
गर्भावस्था के दौरान होनेवाले शारीरिक परिवर्तन	☐	☐	☐

15.*क्या स्वास्थ्य देखभाल पेशेवरोने बच्चे को खिलाने केलिए आपके द्वारा बनायीं योजनाओं के बारे में पूछा था?

- ☐ हाँ
- ☐ नहीं
- ☐ पता नहीं/ याद नहीं
- ☐ आपको प्रश्न लागु नहीं करता (इस गर्भावस्था के दौरान आप किसी स्वास्थ्य देखभाल पेशेवर से नहीं मिले थे)

16.*क्या स्वास्थ्य पेशेवारोंने आपसे, परवरिश या गर्भावस्था से जुडी रस्मों-रिवाजों को पूरा करने की इच्छाओ के बारे में पूछा था?

- ☐ हाँ
- ☐ नहीं
- ☐ आपको प्रश्न लागु नहीं करता (इस गर्भावस्था के दौरानआप किसी स्वास्थ्य देखभाल पेशेवर के पास नहीं गए थे)

आगले प्रश्नों का समुह आपके सबसे हाल ही में हुए प्रसव पीड़ा और बच्चे को जन्मसे सम्मंधित है. इस अनुभाग में 16 प्रश्न है /

17.*जब आपने बच्चे को जन्म दिया, आप कितने सप्ताह गर्भवती थे? ___________ (सप्ताह)

□ पता नहीं

18. *ईस गर्भावस्था में आपने कितने बच्चों को जन्म दिया? ______________ (जैसे अकेला बच्चा, जुडवा)

19.*आपका बच्चा / बच्चे कितने पाउंड/ किलो/ ग्राम का था?

________(किलो) ________(ग्राम) / ________(पाउंड) ________(आउन्स)

________(किलो) ________(ग्राम) / ________(पाउंड) ________(आउन्स) *(यदि आपने एक से अधिक बच्चे को जन्म दिया हो)*

~~**20.***बच्चे को जन्म देते समय आप कहाँ थे?~~

~~*(संपूर्ण विकल्प पहले जोर से पढ़े और केवल एक योग्य लागु होनेवाले जवाब को 'टिक' करें)*~~

21.*किस स्वास्थ्य पेशेवरने आपकी करीबन पूरी प्रसव पीडा के दौरान देखभाल कि?

(इस प्रश्न का जवाब माँ को करने दे और केवल एक योग्य लागु होनेवाले जवाब को 'टिक' करें)

□ प्रसूति या स्त्री रोग के डॉक्टर
□ परिवार के डॉक्टर
□ दाई
□ नर्स / नर्स प्रैक्टिशनर
□ अन्य व्यक्ति *(कृपया स्पष्ट करें)* ________________________
□ कोई नहीं
□ कोई नहीं, मुझे प्रसव पीड़ा नहीं हुई, मेरा योजनाबद्ध सिजेरियन हुआ
□ पता नहीं

22.*किस स्वास्थ्य पेशेवरने बच्चे के जन्म दौरान आपकी सबसे अधिक देखभाल कि ?

(इस प्रश्न का जवाब माँ को करने दे और केवल एक योग्य लागु होनेवाले जवाब को 'टिक' करें)

□ प्रसूति या स्त्री रोग के डॉक्टर
□ परिवार के डॉक्टर, जनरल प्रैक्टिशनर
□ दाई
□ नर्स / नर्स प्रैक्टिशनर
□ अन्य व्यक्ति *(कृपया स्पष्ट करें)*:________________________
□ कोई नहीं
□ पता नहीं

23. ***प्रसव पीड़ा और बच्चे के जन्म के दौरान, निम्नलिखित प्रक्रियाओंमें से किस प्रक्रिया का संचालन हुआ था?**

	हाँ	नहीं
इनडकशन ऑफ लेबर - प्रसव का प्रेरण (आपकी प्रसव पीड़ा शुरू कर दी गयी	☐	☐
औग्मेंटेशन ऑफ लेबर - प्रसव के विस्तार को बढ़ाना (आपके पहले से जारी संकुचन अधिक मजबूत और तेज बनाए गए)	☐	☐
फोरसेप डीलीवरी - डीलीवरी करने संदंश(चिमटी) का उपयोग (बच्चे को निकालने के लिए साधन का उपयोग किया गया)	☐	☐
वैक्यूम का उपयोग (बच्चे को निकालने के लिए सक्शन- हवे के दबाव से बच्चे को निकालने के लिए साधन)	☐	☐
सिजेरियन	☐	☐
एपिसिओटोमि (योनि/मूत्र भाग के उद्घाटन के पास काट दिया गया)	☐	☐
प्रसव के दौरान दर्द को कम करने के लिए एपिड्यूरल- पीठ में सुई (इंजेक्शन)	☐	☐
सिजेरियन करने के लिए पीठ में सुई (इंजेक्शन) लगाना जिस से शारीर का भाग सुन्न हो जाता है	☐	☐
जनरल एनेस्थीसिया (बेशुद्ध करना)	☐	☐
अन्य प्रक्रिया *(कृपया स्पष्ट करें): ________*	☐	☐

24. ***प्रसव पीड़ा और बच्चे के जन्म के दौरान क्या आपको किसी भी प्रकार की चिकित्सा जटिलताएं (कोम्प्लिकेश्न्स) हुई थी?** ***(जैसे: पेरिनियम (मूत्र भाग) का चीरना, युटेराइन रप्चर (गर्भाशय का फूटना), इन्फेक्शन (रोग/संक्रमण), हिमोरेज (अधिक तादाद में खून बहना या बच्चे से जुड़ी कोई कोम्प्लिकेशन)***

☐ हाँ, *(कृपया स्पष्ट करें:* ____

☐ नहीं

यदि आपका सिजेरियन नहीं हुआ था, *तो प्रश्न नंबर 26 पर जाइये*

25. ***यदि बच्चे का जन्म होने के लिए सीजेरियन करना पड़ा, तो इसके लिए मुख्य कारण क्या था?**
(इस प्रश्न का जवाब माँ को करने दे / कृपया किसी केवल एक योग्य लागु जवाब को ' टिक' करें)

☐ यह योजना बनाई गई थी | डॉक्टरने चिकित्सा कारणों के लिए यह सुझाव दिया
☐ यह योजना बनाई गयी थी | लेकिन इसका कारन आपके जानकारी से बाहर है
☐ यह योजना कि मांग आपने स्वयं कि थी | इसके पीछे कोई चिकित्सा से जुडा कारण नहीं था
☐ यह नियोजित नहीं था, लेकिन प्रसव पीड़ा अधिक समय ले रही थी
☐ यह नियोजित नहीं था, परंतु आपके बच्चे के जान को खतरा था
☐ यह नियोजित नहीं था, परंतु आपके जान को खतरा था
☐ यह नियोजित नहीं था, परंतु सीजेरियन होने का कारन आपके जानकारी से बाहर है
☐ अन्य करने *(कृपया स्पष्ट करें)* : ____________________
☐ आपको प्रश्न लागु नहीं करता (आपका सिजेरियन नहीं हुआ था)

26. प्रसव के दौरान क्या आपको घूमने या आरामदायक तरह से लेटने की अनुमति दी गई थी?

(कृपया जोर से पढ़ें और किसी केवल एक योग्य लागु जवाब को 'टिक' करें)

- ☐ हाँ, हमेशा
- ☐ हाँ, कभी कभी
- ☐ हाँ, शायद ही कभी
- ☐ नहीं, चिकित्सा कारणों के लिए नहीं
- ☐ नहीं, नहीं, मैं नहीं जानती कि क्यों
- ☐ नहीं, आपको प्रसव पीड़ा नहीं हुई| आपका योजनाबन्ध सिजेरियन हुआ था

---

27. क्या आपको स्वास्थ्य पेशेवरोंने पूछा था की प्रसव के दौरान आप दर्द का प्रबंधन कैसे करना चाहते थे?

- ☐ हाँ
- ☐ नहीं
- ☐ पता नहीं/ याद नहीं
- ☐ आपको प्रसव पीड़ा नहीं हुई | आपका योजनाबन्ध सिजेरियन हुआ था

---

28. प्रसव पीड़ा के दौरान, स्वास्थ्य पेशेवरों द्वारा दिए गए दर्द प्रबंधक उपचारोंसे क्या आप संतुष्ट थे?

- ☐ हाँ
- ☐ नहीं
- ☐ कभी कभी
- ☐ आपको प्रसव पीड़ा नहीं हुई| आपका योजनाबन्ध सिजेरियन हुआ था

---

29. प्रसव के दौरान आपके साथ होनेवाले परिवार के सदस्यों या अन्य लोगों को चुनने की अनुमति क्या आपको दी थी?

- ☐ हाँ
- ☐ नहीं
- ☐ कभी कभी
- ☐ आपको प्रसव पीड़ा नहीं हुई| आपका योजनाबद्ध सिजेरियन हुआ था

---

30. *<u>प्रसव के दौरान या बच्चे को जन्म देते समय</u> क्या आपके साथ कोई मौजूद था?

(संपूर्ण विकल्प पहले जोर से पढ़े और केवल एक योग्य लागू जवाब को टिक करें)

- ☐ हाँ, हमेशा
- ☐ हाँ, कभी कभी
- ☐ हाँ, शायद ही कभी
- ☐ नहीं
- ☐ पता नहीं/ याद नहीं

---

31. *यदि आपका जवाब हाँ है , तो हमे बताईये आपके साथ कौन मौजूद थे ?

(यदि एक से अधिक सदस्य थे, तो सारे सदस्यों का उल्लेख करिए)

__________________________(आपसे कैसे संबंधित थे)

__________________________(आपसे कैसे संबंधित थे)

__________________________(आपसे कैसे संबंधित थे)

- ☐ आपको प्रश्न लागु नहीं करता

---

32. *क्या स्वास्थ्य पेशेवारोंने आपसे, परवरिश या बच्चे के जन्म या प्रसव (लेबर) से जुडी रस्मों-रिवाजों को पूरा करने की इच्छाओ के बारे में पूछा था?

- ☐ हाँ
- ☐ नहीं
- ☐ नहीं, क्योंकि मैंने उनके पुछनेसे पहले ही पुछ लिया था

अगले प्रश्न का समुह प्रसवोत्तर अवधि (बच्चेके जनम के बाद वाला समय) से सम्मंधित है| इस अनुभाग में आपसे 14 प्रश्न पूछे जाएँगे |

33. *क्या विशेष देखभाल के लिए बच्चे को अलग कमरे में रखने की जरूरत थी?

(संपूर्ण विकल्प पहले जोर से पढे और केवल एक योग्य लागू जवाब को टिक करें)

- ☐ हाँ, नियोनेटल यूनिट (नवजात गहन चिकित्सा इकाई) में
- ☐ हाँ, स्पेशल बेबी केयर यूनिट (विशेष शिशु देखभाल इकाई) में
- ☐ हाँ, नर्सरी में
- ☐ हाँ (लेकिन आपको पता नहीं / याद नहीं कहाँ)
- ☐ नहीं
- ☐ याद नहीं/ पता नहीं

34. ~~बच्चे के जन्म के बाद, कब तक आप अस्पताल या दवाखाने (क्लिनिक) में रहे थे?~~

35. ~~आपके अनुसार यह समय बहुत कम, बहुत ज्यादा, बिलकुल योग्य था?~~

- ☐
- ☐
- ☐
- ☐

36. आपके अस्पताल / बिर्थिंग केंद्र में रहते दौरान, क्या स्वास्थ्य देखभाल पेशेवरने भोजन सम्मंधित आपके इच्छाओं के बारे में पूछा था? *(जैसे भोजन का तापमान, खाने से जुडे धार्मिक विश्वास, शाकाहारी भोजन, अन्य खाद्य प्रकार)*

- ☐ हाँ
- ☐ नहीं
- ☐ पता नहीं/ याद नहीं
- ☐ आपको प्रश्न लागु नहीं करता (आपके बच्चे का जनम घर में हुआ था)

37. *क्या स्वास्थ्य पेशेवारोंने आपसे, परवरिश या बच्चे के जन्म के बाद से जुडी रस्मों-रिवाजों को पूरा करने की इच्छाओ के बारे में पूछा था?

- ☐ हाँ
- ☐ नहीं
- ☐ पता नहीं/ याद नहीं

38. **जन्म के पहले घंटे में, क्या आपको और आपके शिशु को बिना कपडे, सीने से लगाने दिया था?**

- ☐ हाँ
- ☐ नहीं (यदि आपका जवाब नहीं है, तो हम इसका कारण जानना चाहेंगे) ________________________________

39. ***स्वास्थ्य पेशेवरने आपको स्तनपान शुरू करने में मदद या मदद करने की पेशकश कब की थी?**

(इस प्रश्न का जवाब माँ को करने दे और किसी एक योग्य लागु जवाब को 'टिक' करें)

- ☐ जन्म के बाद पहले घंटे में
- ☐ कोई स्वास्थ्य पेशेवर तुरंत नहीं आए, लेकिन जब वे आए, मैं बच्चे को जन्म दिए स्थान पर ही थी (जैसे कि अस्पताल, बिर्थिंग केंद्र या घर पे)
- ☐ बाद में, नियुक्ति (अपॉइंटमेंट) के दौरान,
- ☐ किसी ने मदद या मदद की पेशकश नहीं की
- ☐ याद नहीं/ पता नहीं
- ☐ मैं अपने बच्चे को स्तनपान नहीं करना चाहती थी

40. ***क्या अपके स्वास्थ्य देखभाल पेशेवरने स्तनपान सम्बंधित समाज में उपलब्ध सेवाओं के बारे में जानकारी दी थी ?**

- ☐ हाँ
- ☐ नहीं, लेकिन मुझे जानकारी की जरुरत नहीं थी (प्रश्न नंबर 42 पर जाइये)
- ☐ नहीं, लेकिन मुझे जानकारी की जरुरत थी (प्रश्न नंबर 42 पर जाइये)
- ☐ पता नहीं/ याद नहीं

41. ~~***यदि आपका जवाब हाँ है, तो क्या आपने उन बताये गए स्तनपान समर्थन सेवाओं का उपयोग किया था?**~~

☐

42. ~~***क्या आप या आपके बच्चे को, बच्चे के जन्म के बाद, इस गर्भावस्था के साथ जुड़े किसी भी कारण (नियमित देखभाल को मिलाकर), "स्वास्थ्य देखभाल" पेशेवर को मिलना पड़ा था?**~~

☐

43. ~~***यदि आपका जवाब हां है, तो हम उसका कारन जानना चाहेंगे?**~~ ________________________

44. ~~*यदि आपका जवाब हां है, तो हम जानना चाहेंगे की आप किस से मिले थे?~~
~~*(इस प्रश्न का जवाब माँ को करने दे और सभी योग्य लागु जवाबोंको को 'टिक' करें)*~~

☐

45. ~~*बच्चे को जन्म देने के बाद क्या आप स्वास्थ्य देखभाल पेशेवर को "अपने या बच्चे (शिशु) के स्वास्थ्य सम्बंधित मिलना चाहते थे लेकिन नहीं मिल सके?~~

☐

46. ~~*यदि आप "स्वास्थ्य देखभाल" पेशेवर से नहीं मिल सके, हम इसका कारन आपसे जानना चाहेंगे?~~
~~*(इस प्रश्न का जवाब माँ को करने दे और सभी योग्य लागु होने वाले जवाबों को 'टिक' करें.)*~~

☐
☐
☐
☐
☐
☐
☐
☐
☐
☐
☐
☐
☐
☐
☐
☐
☐
☐
☐
☐
☐

प्रश्नों का यह सेट आपके सबसे हाल ही में हुए गर्भावस्था के दौरान, समग्र मातृत्व स्वास्थ्य देखभाल अनुभवों के बारे में है | इस खंड में 20 प्रश्न हैं |

47. यदि आप बीते हुए समय को याद करते है, तो क्या आपको लगता है की कोई अन्य सलाह/ मदद /जानकारी आपको मिलनी चाहिए थी, जो नहीं मिली हो?

____

____

____

48. *कुल मिलाकर, जभी आप स्वास्थ्य पेशेवरों को मिले, क्या उन्होंने सही ढंग से आपका स्वागत किया है?

अ) गर्भावस्था के दौरान

- ☐ हमेशा
- ☐ कभी कभी
- ☐ शायद ही कभी
- ☐ कभी नहीं

ब) प्रसव पीड़ा और जन्म के दौरान

- ☐ हमेशा
- ☐ कभी कभी
- ☐ शायद ही कभी
- ☐ कभी नहीं

क) बच्चे के जन्म के बाद

- ☐ हमेशा
- ☐ कभी कभी
- ☐ शायद ही कभी
- ☐ कभी नहीं

49. *कुल मिलाकर, क्या स्वास्थ्य पेशेवारोंने आपका सम्मान किया था?

अ) गर्भावस्था के दौरान

- ☐ हमेशा
- ☐ कभी कभी
- ☐ शायद ही कभी
- ☐ कभी नहीं

ब) प्रसव पीड़ा और जन्म के दौरान

- ☐ हमेशा
- ☐ कभी कभी
- ☐ शायद ही कभी
- ☐ कभी नहीं

क) बच्चे के जन्म के बाद

- ☐ हमेशा
- ☐ कभी कभी
- ☐ शायद ही कभी
- ☐ कभी नहीं

---

50. *कुल मिलाकर, क्या स्वास्थ्य पेशेवर मददगार थे?

अ) गर्भावस्था के दौरान

- ☐ हमेशा
- ☐ कभी कभी
- ☐ शायद ही कभी
- ☐ कभी नहीं

ब) प्रसव पीड़ा और जन्म के दौरान

- ☐ हमेशा
- ☐ कभी कभी
- ☐ शायद ही कभी
- ☐ कभी नहीं

क) बच्चे के जन्म के बाद

- ☐ हमेशा
- ☐ कभी कभी
- ☐ शायद ही कभी
- ☐ कभी नहीं

51. *कुल मिलाकर, आप प्राप्त स्वास्थ्य सेवा के साथ खुश थी?

अ) गर्भावस्था के दौरान

- ☐ हमेशा
- ☐ कभी कभी
- ☐ शायद ही कभी
- ☐ कभी नहीं

ब) प्रसव पीड़ा और जन्म के दौरान

- ☐ हमेशा
- ☐ कभी कभी
- ☐ शायद ही कभी
- ☐ कभी नहीं

क) बच्चे के जन्म के बाद

- ☐ हमेशा
- ☐ कभी कभी
- ☐ शायद ही कभी
- ☐ कभी नहीं

52.*अपनी गर्भावस्था, प्रसव पीड़ा या बच्चे के जन्म के दौरान, क्या कभी स्वास्थ्य पेशेवरोंने आपसे ऐसे कुछ करने को कहाँ जो आप करना नहीं चाहती थी?

- ☐ हाँ
- ☐ नहीं
- ☐ पता नहीं/ याद नहीं

53.यदि आपका जवाब हाँ है, तो हम जानना चाहेंगे कि वह कौनसी चीजें है?

____

☐ आपको प्रश्न लागु नहीं करता

54. क्या स्वास्थ्य पेशेवरोंने आपसे पूछा की आप परवरिश के लिए, महिला या पुरुष स्वास्थ्य पेशेवर चाहेंगे?

अ) गर्भावस्था के दौरान

- ☐ हमेशा
- ☐ कभी कभी
- ☐ शायद ही कभी
- ☐ कभी नहीं

टिप्पणी________________

ब) प्रसव पीड़ा और जन्म के दौरान

- ☐ हमेशा
- ☐ कभी कभी
- ☐ शायद ही कभी
- ☐ कभी नहीं

टिप्पणी________________

क) बच्चे के जन्म के बाद पहला दिन

- ☐ हमेशा
- ☐ कभी कभी
- ☐ शायद ही कभी
- ☐ कभी नहीं

टिप्पणी________________

55. *क्या आपको स्वास्थ्य पेशेवरों द्वारा उपलब्ध कराई गई जानकारी समझ आयी थी?

अ) गर्भावस्था के दौरान

- ☐ हमेशा
- ☐ कभी कभी
- ☐ शायद ही कभी
- ☐ कभी नहीं

टिप्पणी_______________

ब) प्रसव पीड़ा और जन्म के दौरान

- ☐ हमेशा
- ☐ कभी कभी
- ☐ शायद ही कभी
- ☐ कभी नहीं

टिप्पणी_______________

क) बच्चे के जन्म के बाद पहला दिन

- ☐ हमेशा
- ☐ कभी कभी
- ☐ शायद ही कभी
- ☐ कभी नहीं

टिप्पणी_______________

---

56. *स्वास्थ्य पेशेवरों द्वारा उपलब्ध कराई गई जानकारी यदि कोई अन्य भाषा में होती तो क्या आप उसे बेहतर समझते?**

- ☐ यदि आपका जवाब हाँ है, तो हम जानना चाहेंगे की वह कौनसी भाषा है _________________(जैसे आपकी मातृ भाषा)
- ☐ नहीं
- ☐ पता नहीं/ याद नहीं

57. क्या स्वास्थ्य पेशेवरोंने आपको कोई व्याख्या सेवा (अपरिचित भाषा को आसानी से समझ आने वाली भाषा में बदलना) प्रदान की?

अ) गर्भावस्था के दौरान

- □ हाँ
- □ नहीं
- □ आपको प्रश्न लागु नहीं करता

ब) प्रसव पीड़ा और जन्म के दौरान

- □ हाँ
- □ नहीं
- □ आपको प्रश्न लागु नहीं करता

क) बच्चे के जन्म के बाद पहला दिन

- □ हाँ
- □ नहीं
- □ आपको प्रश्न लागु नहीं करता

58. कितनी बार आपके साथ कोई ऐसा व्यक्ति मौजूद था जो आप की भाषा को समझकर आप के लिए व्याख्या कर सकता था?

अ) गर्भावस्था के दौरान

- □ हमेशा
- □ कभी कभी
- □ शायद ही कभी
- □ कभी नहीं
- □ आपको प्रश्न लागु नहीं करत

ब) प्रसव पीड़ा और जन्म के दौरान

- □ हमेशा
- □ कभी कभी
- □ शायद ही कभी
- □ कभी नहीं
- □ आपको प्रश्न लागु नहीं करता

क) बच्चे के जन्म के बाद पहला दिन

- □ हमेशा
- □ कभी कभी
- □ शायद ही कभी
- □ कभी नहीं
- □ आपको प्रश्न लागु नहीं करता

59. *M यदि आपके साथ भाषा की व्याख्या के लिए कोई व्यक्ति मौजूद था, तो वह कौन था?

(संपूर्ण विकल्प पहले जोर से पढ़े और सभी योग्य लागु जवाबों को 'टिक' करें)

अ) गर्भावस्था के दौरान

- ☐ पति / साथी
- ☐ परिवार के अन्य सदस्य / दोस्त
- ☐ हेल्थकेयर व्यवसायी
- ☐ आपका बच्चा
- ☐ अपरिचित भाषा को आसानी से समझ आने वाली भाषा में बदलनेवाला व्यवसाहिक व्यक्ति
- ☐ मरीज या मरीज के परिवार के सदस्य / दोस्त
- ☐ अन्य, *(कृपया स्पष्ट करें)* : __________________
- ☐ आपको प्रश्न लागु नहीं करता

ब) प्रसव पीड़ा और जन्म के दौरान

- ☐ पति / साथी
- ☐ परिवार के अन्य सदस्य / दोस्त
- ☐ हेल्थकेयर व्यवसायी
- ☐ आपका बच्चा
- ☐ व्यावसायिक दुभाषिया
- ☐ एक और मरीज या मरीज के परिवार के सदस्य / दोस्त
- ☐ अन्य, *(कृपया स्पष्ट करें)*: __________________
- ☐ आपको प्रश्न लागु नहीं करता

क) बच्चे के जन्म के बाद पहला दिन

- ☐ पति / साथी
- ☐ परिवार के अन्य सदस्य / दोस्त
- ☐ हेल्थकेयर व्यवसायी
- ☐ आपका बच्चा
- ☐ व्यावसायिक दुभाषिया
- ☐ एक और मरीज या मरीज के परिवार के सदस्य / दोस्त
- ☐ अन्य, *(कृपया स्पष्ट करें)*: __________________
- ☐ आपको प्रश्न लागु नहीं करता

---

60. **M क्या आप उनकी व्याख्या से संतुष्ट थे?**

- ☐ हाँ
- ☐ नहीं
- ☐ पता नहीं/ याद नहीं
- ☐ आपको प्रश्न लागु नहीं करता

---

61. ***प्रसव पीड़ा, बच्चे के जन्म के दौरान या जन्म के बाद, क्या "स्वास्थ्य देखभाल" पेशेवरोंने आपको परवरिश या बच्चे के जन्म से**

जुडी रस्मों-रिवाजों को पूरा करने की इच्छाओं के बारे में पूछा था?

- ☐ हाँ
- ☐ नहीं (*प्रश्न नंबर 64 से पढना शुरू करिए*)
- ☐ पता नहीं/ याद नहीं

---

62. यदि आपका जवाब हाँ है, तो हम उन इच्छाओं के बारे में जानना चाहेंगे जिन्हें आप पूरा नहीं कर सके ?

१) ____________

*२)*___________

३) ___________

- ☐ आपको प्रश्न लागु नहीं करता

---

63. यदि आपका जवाब हाँ है, तो "स्वास्थ्य देखभाल पेशेवरोने इन इच्छाओं का पालन करने की अनुमति नहीं देने का कारण क्या आपको बताया था?

१) ____________

*२)*___________

३) ___________

- ☐ आपको प्रश्न लागु नहीं करता

---

64. *क्या आपको लगता है की स्वास्थ्य देखभाल पेशेवर कुछ चीजें अलग ढंगसे या बेहतर रूप से कर सकते थे?

अ) गर्भावस्था के दौरान

- ☐ हाँ *(प्रश्न नंबर 65 'अ' को पूरा करें)*
- ☐ नहीं
- ☐ पता नहीं/याद नहीं

ब) प्रसव पीड़ा और जन्म के दौरान

- ☐ हाँ *(प्रश्न नंबर 65 'ब' को पूरा करें)*
- ☐ नहीं
- ☐ पता नहीं/याद नहीं

क) बच्चे के जन्म के बाद

- ☐ हाँ *(प्रश्न नंबर 65 'क' को पूरा करें)*
- ☐ नहीं
- ☐ पता नहीं/याद नहीं

---

65. यादी आपका जवाब हाँ है, तो हम जानना चाहेंगे कि आपके अनुसार वे कौनसी चीजें है जो अलग या बेहतर ढंग से की जा सकती है|

इसे करने में कौन मदद कर सकता है?

अ) गर्भावस्था के दौरान ________________________

ब) प्रसव पीड़ा और जन्म के दौरान ________________________

क) बच्चे के जन्म के बाद ________________________

66. *गर्भावस्था के दौरान मिली देखभाल सेवाएँ, बच्चे का जन्म या जन्म के बाद वाले समय से जुड़ेकिसी भी अनुभवों के बारे में वर्णन करें जिससे आप:

अ) बहुत खुश है ________________________

ब) बहुत नाखुश है ________________________

आपके हाल ही में हुए गर्भावस्था के बारे में सोचते हुए हमें बताईएँ कि नीचे लिखे हुए 11 वाक्य कितनी बार आपके लिए सही ठहरें हैं |

67. *यदि मेरे मन में कोई सवाल हो, तो स्वास्थ्य पेशेवरों ने मुझे उनसे पूछने के लिए कहा

- ☐ हमेशा
- ☐ कभी कभी
- ☐ शायद ही कभी
- ☐ कभी नहीं

68. स्वास्थ्य पेशेवर जल्दी में रहते थे

- ☐ हमेशा
- ☐ कभी कभी
- ☐ शायद ही कभी
- ☐ कभी नहीं

69. *मैंने अपनी चिंताओं को स्वास्थ्य पेशेवरों द्वारा गंभीरता से लिए जाते महसूस किया

अ) गर्भावस्था के दौरान

- □ हमेशा
- □ कभी कभी
- □ शायद ही कभी
- □ कभी नहीं
- □ आपको प्रश्न लागू नहीं करता (गर्भावस्था में देखभाल प्राप्त नहीं हुई)

ब) प्रसव पीड़ा और जन्म के दौरान

- □ हमेशा
- □ कभी कभी
- □ शायद ही कभी
- □ कभी नहीं
- □ आपको प्रश्न लागू नहीं करता (कोई स्वास्थ्य देखभाल पेशेवर मौजूद नहीं था)

क) बच्चे के जन्म के बाद

- □ हमेशा
- □ कभी कभी
- □ शायद ही कभी
- □ कभी नहीं
- □ आपको प्रश्न लागू नहीं करता (कोई स्वास्थ्य देखभाल पेशेवर मौजूद नहीं था)

70.मुझे गर्भावस्था सम्बंधित देखभाल प्राप्त करने के लिए अधिक समय तक रुकना पड़ा

अ) गर्भावस्था के दौरान

- ☐ हमेशा
- ☐ कभी कभी
- ☐ शायद ही कभी
- ☐ कभी नहीं
- ☐ आपको प्रश्न लागू नहीं करता (गर्भावस्था में देखभाल प्राप्त नहीं हुई)

ब) प्रसव पीड़ा और जन्म के दौरान

- ☐ हमेशा
- ☐ कभी कभी
- ☐ शायद ही कभी
- ☐ कभी नहीं
- ☐ आपको प्रश्न लागू नहीं करता (कोई स्वास्थ्य देखभाल पेशेवर मौजूद नहीं था)

क) बच्चे के जन्म के बाद

- ☐ हमेशा
- ☐ कभी कभी
- ☐ शायद ही कभी
- ☐ कभी नहीं
- ☐ आपको प्रश्न लागू नहीं करता (कोई स्वास्थ्य देखभाल पेशेवर मौजूद नहीं था)

71.*स्वास्थ्य पेशेवर किसी बदलाव या अन्य घटनाओं के बारे में मुझे सूचित करते थे

अ) गर्भावस्था के दौरान

- ☐ हमेशा
- ☐ कभी कभी
- ☐ शायद ही कभी
- ☐ कभी नहीं
- ☐ आपको प्रश्न लागू नहीं करता (गर्भावस्था में देखभाल प्राप्त नहीं हुई)

ब) प्रसव पीड़ा और जन्म के दौरान

- ☐ हमेशा
- ☐ कभी कभी
- ☐ शायद ही कभी
- ☐ कभी नहीं
- ☐ आपको प्रश्न लागू नहीं करता (कोई स्वास्थ्य देखभाल पेशेवर मौजूद नहीं था)

क) बच्चे के जन्म के बाद

- ☐ हमेशा
- ☐ कभी कभी
- ☐ शायद ही कभी
- ☐ कभी नहीं
- ☐ आपको प्रश्न लागू नहीं करता (कोई स्वास्थ्य देखभाल पेशेवर मौजूद नहीं था)

72.*मुझे समझ में नहीं आए चीजों के बारे में पूछने में, मुझे आसानी महसूस हुई

अ) गर्भावस्था के दौरान

- □ हमेशा
- □ कभी कभी
- □ शायद ही कभी
- □ कभी नहीं
- □ आपको प्रश्न लागू नहीं करता (गर्भावस्था में देखभाल प्राप्त नहीं हुई)

ब) प्रसव पीड़ा और जन्म के दौरान

- □ हमेशा
- □ कभी कभी
- □ शायद ही कभी
- □ कभी नहीं
- □ आपको प्रश्न लागू नहीं करता (कोई स्वास्थ्य देखभाल पेशेवर मौजूद नहीं था)

क) बच्चे के जन्म के बाद

- □ हमेशा
- □ कभी कभी
- □ शायद ही कभी
- □ कभी नहीं
- □ आपको प्रश्न लागू नहीं करता (कोई स्वास्थ्य देखभाल पेशेवर मौजूद नहीं था)

73. *निर्णय लेते समय मेरी इच्छाओं को ध्यान में नहीं रखा जा रहा था

अ) गर्भावस्था के दौरान

- □ हमेशा
- □ कभी कभी
- □ शायद ही कभी
- □ कभी नहीं
- □ आपको प्रश्न लागू नहीं करता (गर्भावस्था में देखभाल प्राप्त नहीं हुई)

ब) प्रसव पीड़ा और जन्म के दौरान

- □ हमेशा
- □ कभी कभी
- □ शायद ही कभी
- □ कभी नहीं
- □ आपको प्रश्न लागू नहीं करता (कोई स्वास्थ्य देखभाल पेशेवर मौजूद नहीं था)

क) बच्चे के जन्म के बाद

- □ हमेशा
- □ कभी कभी
- □ शायद ही कभी
- □ कभी नहीं
- □ आपको प्रश्न लागू नहीं करता (कोई स्वास्थ्य देखभाल पेशेवर मौजूद नहीं था)

74. *स्वास्थ्य पेशेवर बहुत ही उत्साह और यकीन दिलाते थे

अ) गर्भावस्था के दौरान

- □ हमेशा
- □ कभी कभी
- □ शायद ही कभी
- □ कभी नहीं
- □ आपको प्रश्न लागू नहीं करता (गर्भावस्था में देखभाल प्राप्त नहीं हुई)

ब) प्रसव पीड़ा और जन्म के दौरान

- □ हमेशा
- □ कभी कभी
- □ शायद ही कभी
- □ कभी नहीं
- □ आपको प्रश्न लागू नहीं करता (कोई स्वास्थ्य देखभाल पेशेवर मौजूद नहीं था)

क) बच्चे के जन्म के बाद

- □ हमेशा
- □ कभी कभी
- □ शायद ही कभी
- □ कभी नहीं
- □ आपको प्रश्न लागू नहीं करता (कोई स्वास्थ्य देखभाल पेशेवर मौजूद नहीं था)

75. *क्या स्वास्थ्य पेशेवरों ने आपको स्पष्टीकरण देने में, जितना समय देना चाहिए उतना समय दिया था?

अ) गर्भावस्था के दौरान

- □ हमेशा
- □ कभी कभी
- □ शायद ही कभी
- □ कभी नहीं
- □ आपको प्रश्न लागू नहीं करता (गर्भावस्था में देखभाल प्राप्त नहीं हुई)

ब) प्रसव पीड़ा और जन्म के दौरान

- □ हमेशा
- □ कभी कभी
- □ शायद ही कभी
- □ कभी नहीं
- □ आपको प्रश्न लागू नहीं करता (कोई स्वास्थ्य देखभाल पेशेवर मौजूद नहीं था)

क) बच्चे के जन्म के बाद

- □ हमेशा
- □ कभी कभी
- □ शायद ही कभी
- □ कभी नहीं
- □ आपको प्रश्न लागू नहीं करता (कोई स्वास्थ्य देखभाल पेशेवर मौजूद नहीं था)

76. *कुल मिलाकर, क्या आपको लगता है कि स्वास्थ्य पेशेवरोंने आपके साथ अन्य लोगों की तुलना में अलग तरह से व्यवहार किया

था? (उदाहरण के लिए: क्योंकि आपकी भाषा या उच्चारण अलग था, संस्कृति, जाति या त्वचा के रंग के कारन या धर्म, माइग्रेशन स्थिति, या स्वास्थ्य बीमा के कारन?)

- ☐ हमेशा *(कृपया इसका कारण प्रश्न नंबर 77 में स्पष्ट करें)*
- ☐ कभी कभी *(कृपया इसका कारण प्रश्न नंबर 77 में स्पष्ट करें)*
- ☐ शायद ही कभी *(कृपया इसका कारण प्रश्न नंबर 77 में स्पष्ट करें)*
- ☐ कभी नहीं *(प्रश्न नंबर 78 पर जाएँ)*

77. *यदि आपका जवाब हाँ है, तो आपके अनुसार इसका क्या कारन है ?

(इस प्रश्न का जवाब माँ को करने दे / कृपया सारे योग्य लागु होने वाले जवाबों को 'टिक' करें)

- ☐ भाषा या उच्चारण
- ☐ संस्कृति
- ☐ रेस / जातीय पृष्ठभूमि
- ☐ त्वचा के रंग
- ☐ धर्म
- ☐ माइग्रेशन स्टेटस
- ☐ स्वास्थ्य बीमा की स्टेटस
- ☐ अन्य करनें *(कृपया स्पष्ट करें)*: __________
- ☐ आपको प्रश्न लागू नहीं करता

निम्नलिखित सवालों का सेट अपके प्रसूति के इतिहास के बारे में है / इस खंड में 8 प्रश्न हैं /

78. *आपके कुल मिलाकर (यानि हाल ही में जन्में बच्चे को मिलाकर) कितने गर्भावस्था हुए है? _______

~~**79.** *कितने गर्भावस्था गर्भपात (मिसकेरेज) में समाप्त हो गए? _______ (आपको प्रश्न लागुनाहीं करता)~~

☐

~~यह प्रश्न पूछते समय ध्यान रखें कि आपके साथ कोई और व्यक्ति मौजद नहीं है |~~

~~**80.** *कितने गर्भावस्था समाप्त किए गए? _______ (आपको प्रश्न लागुनाहीं करता)~~

☐

~~**81.** *कितने गर्भावस्था मृत प्रसव में समाप्त हुए (बच्चा पैदा होने से पहले मृत्यु होना - स्टील बर्थ)? ________~~

☐

82. *कितने जीवित शिशु, ३७ (37) सप्ताह से पहले पैदा हुए थे? _______

- ☐ आपको प्रश्न लागू नहीं करता

83. *कितने जीवित शिशु, ३७ (37) सप्ताह पुरे होने के बाद पैदा हुए थे? _______

- ☐ आपको प्रश्न लागू नहीं करता

84. पिछले गर्भावस्था में क्या आपको कोई भी चिकित्सा जटिलता (मेडिकल कोम्प्लिकेशन) हुई थी ?

- □ हाँ
- □ नहीं *(प्रश्न नंबर 86 पर जाएँ)*
- □ आपको प्रश्न लागु नहीं होता *(प्रश्न नंबर 86 पर जाएँ)*

85. ***यदि आपको पिछले गर्भावस्था में किसी प्रकार की कोम्प्लिकेशन घटित हुई थी, तो हमे बताइये वे कौनसी थी?**

(इस प्रश्न का जवाब माँ को करने दे और कृपया सारे योग्य लागु होने वाले जवाबों को 'टिक' करें)

- □ सिजेरियन
- □ रक्ताल्पता (खून में हिमोग्लोबिन की कमी)
- □ उच्च रक्तचाप (बिपि)
- □ प्री-एक्लाम्पसिया (जेस्तेशनल हयपर्तेन्श्न)
- □ अपरिपक्व प्रसव (सामान्य समय से पूर्व होने वाला प्रसव/ लेबर)
- □ डीप वेन थ्रोम्बोसिस (पैरोंके गहरी शिरा में होने वाली खून की गांठ)
- □ जेस्तेशनल डायबेटीस (खून मैं होनेवाली शक्खर की अधिकता या कमी)
- □ प्लासेन्टा पराविया (बच्चे की नाल, गर्भाशय के अनुचित भाग से जुड़ना)
- □ प्लेसेंटल अबरपशन (बच्चे के जन्म समय, नाल का कानुचित ढंग से टूटना)
- □ युरीनरी ट्रेक इन्फेक्शन (मूत्र मार्ग रोग)
- □ तीव्र पीठ दर्द
- □ प्री-टर्म रप्चर ऑफ मेम्ब्रेन (झिल्ली का समय से पहले टूट जाना)
- □ डिप्रेशन (उदासी महसूस करना)
- □ अन्य चिकित्सा जटिलताऐं (मेडिकल कॉम्प्लिकेशन) *(कृपया स्पष्ट करें):* ___________________________
- □ पता नहीं
- □ आपको प्रश्न लागु नहीं होता

प्रश्नों का अंतिम सेट आप और आपके परिवार के बारे में है। इस सेट में २७ प्रश्न हैं।

86. ***आपकी वैवाहिक स्थिति क्या है?**

- □ विवाहित
- □ आम सहमति से संघ (अविवाहित भागीदारों)
- □ विधवा
- □ अलग
- □ तलाकशुदा
- □ कुवारी

87.*आप किसके साथ रहते है?
(पहले जवाबों को पढ़ें और सभी लागु उत्तरों को टिक करें)

	हाँ	नहीं	पता नहीं
पति / पुरुष साथी	☐	☐	☐
एक महिला साथी	☐	☐	☐
आपके माता/ पिता	☐	☐	☐
आपके भाइयों / बहनों के साथ	☐	☐	☐
साथी के माता / पिता	☐	☐	☐
साथी के भाइ / बहन	☐	☐	☐
मित्र (ओं)	☐	☐	☐
बच्चों के साथ (हाल ही में जन्में बच्चे के अलावा अन्य बच्चे)	☐	☐	☐
अन्य जवाब *(कृपया स्पष्ट करें):* ______________	☐	☐	☐
कोई नहीं, मैं अपने बच्चे के साथ अकेली रहती हूँ	☐	☐	☐
कोई नहीं, मैं अकेली रहती हूँ	☐	☐	☐

88.*खून से जुड़े आपके कितने बच्चे आपके साथ रहते है? (अपने हाल ही में जन्में बच्चे सहित हमे गिनती बताइये)? ___________

89.*आपके कितने बच्चों का जन्म इस देश में हुआ है? (अपने हाल ही में जन्में बच्चे सहित हमे गिनती बताइये)? __________

90.*आपके जन्म की तारीख ?____________________( ) ____________________(महीने)_______________(साल)

91.*[M]आपके माताजी का जन्म कौनसे देश में हुआ था? ___________

92.*[M]आपके पिता का जन्म कौनसे देश में हुआ था? ___________

अगले प्रश्नों का समुह आपके इम्मिग्रशन के इतिहास के बरे में, विस्तार(डिटेल) से जाँच करता है / हम इस जानकारी में दिलचस्पी रखते है क्योंकि, हमे इस देश में रहनेवाले अंतरराष्ट्रीय माइग्रेंटो के अनुभवो के बरे में जानना चहते है / आपने बताये सारे जवाब हमारे पास निजे रहेंगे, कोई भी जानकारी इम्मिग्रशन ओफीस में नहीं दी जयेगि / ईन प्रश्नों का उत्तर देने से आपके इम्मिग्रशन स्टेटस पर कोई पराभव नहीं पड़ेगा /

93.*[M]**आपकी वर्तमान आव्रजन स्थिति (इमीग्रेशन स्टेटस) क्या है?**

(इस प्रश्न का जवाब माँ को करने दे और योग्य लागु होनेवाले जवाबों को 'टिक' करें)

- ☐ पर्मेनंट रेजिडेंट
- ☐ रिफ्यूजी
- ☐ शरण साधक (रिफ्यूजी क्लेमंट- यानि आपने कनाडा आने के बाद रेफ्यूजी स्टेटस पाया / एसाइलम सीकर)
- ☐ टेम्परारी वर्कर/ लाइव-इन करेगिवर
- ☐ टेम्परारी रेजिडेंट
- ☐ छात्र
- ☐ विजिटर
- ☐ कोई स्थिति नहीं
- ☐ आपका स्टेटस किसी ककजद पर नहीं लिखा है
- ☐ नागरिक
- ☐ अन्य स्तिति *(कृपया स्पष्ट करें)* :_________________

94.[M]**आपका यह दर्जा कब से है? ______**(दिन) ______(हफ्ते)______(महीने)______(साल)

95. [M]**क्या आपका इमीग्रेशन स्टेटस यहाँ आने के बाद बदला है? _______**

- ☐ हाँ
- ☐ नहीं *(प्रश्न नंबर 97 पर जाएँ)*

96.*[M]**यदि आपका जवाब हाँ हैं,तो इसके पहले आपका इम्मीग्रेशन स्टेटस क्या था ?**

- ☐ पर्मेनंट रेजिडेंट
- ☐ रिफ्यूजी
- ☐ शरण साधक (रिफ्यूजी क्लेमंट/ एसाइलम सीकर)
- ☐ टेम्परारी वर्कर/ लाइव-इन करेगिवर
- ☐ टेम्परारी रेजिडेंट
- ☐ छात्र
- ☐ विजिटर
- ☐ कोई स्थिति नहीं
- ☐ आपका स्टेटस किसी कागजाद पे नहीं लिखा है
- ☐ नागरिक
- ☐ अन्य स्टेटस स्पष्ट करें: _________________
- ☐ आपको प्रश्न लागु नहीं करता (*आपके स्टेटस में कोई बदलाव नहीं हुआ है)*

97.[M]**क्या आपका कभी रेफूजी का स्टेटस रहा है?**

- ☐ हाँ
- ☐ नहीं
- ☐ पता नहीं/ याद नहीं

98.*ᴹ**क्या आपको कभी आव्रजन (इम्मिग्रेशन) हिरासत केंद्र (डिटेंशन सेंटर) में रखा गया है?**

- ☐ हाँ
- ☐ नहीं *(प्रश्न नंबर 101 पर जाएँ)*

99.ᴹ**यदि आपका जवाब हाँ है, तो कितने समय के लिए आपको वहाँ रखा गया था?**

______(दिन) ______(हफ्ते)______(महीने)______(साल)

- ☐ आपको प्रश्न लागु नहीं करता

100. *ᴹ**यदि आपका जवाब हाँ है, क्या आपको इस गर्भावस्था के दौरान हिरासत में रखा गया था?**

- ☐ हाँ
- ☐ नहीं
- ☐ पता नहीं

101. ***स्वास्थ्य सेवाओं के लिए, पैसों का आयोजन कौन करता है? (विकल्प को पहले जोर से पढ़े और सभी योग्य लागु जवाबों को टिक करें)**

	हाँ	नहीं	पता नहीं
सार्वजनिक रूप से फण्ड की गयी स्वास्थ्य बीमा (RAMQ)	☐	☐	☐
निजी स्वास्थ्य बीमा	☐	☐	☐
शरण चाहने वालों के लिए (रिफ्यूजी/ एसाइलम सीकर) विशेष सरकारी रूप से फण्ड की गयी स्वास्थ्य बीमा	☐	☐	☐
आप अपने स्वास्थ्य सेवाओं के लिए स्वयं योजना करते है	☐	☐	☐

102. ***शिक्षा के आपके कौन से उच्चतम स्तर पूरे हो गए है?**

- ☐ प्राथमिक स्कूल - आठ्वी कक्षा
- ☐ माध्यमिक डिप्लोमा – दसवी कक्षा
- ☐ डिप्लोमा (जैसे व्यापार स्कूल, कॉलेज, विश्वविद्यालय) - बारहवी कक्षा
- ☐ ग्रेजुएट डिप्लोमा (मास्टर, डॉक्टरेट)
- ☐ कोई नहीं

103. ᴹ**आपको इस देश में कानूनी तौर पर काम करने की अनुमति है?**

- ☐ हाँ
- ☐ नहीं
- ☐ पता नहीं

104. ***बच्चा पैदा होने से पहले आपकी अंतिम नौकरी कौनसी थी? (जैसे कि डॉक्टर, शिक्षक, डेटा कि एंट्री कर्नेवला क्लर्क, नर्सिंग होम एड, हौसकीपर, सब्जी उत्पादक, कपड़ा रंगाई मशीन ऑपरेटर, होटल क्लीनर, कॉल सेंटर में फोन करने वाल)**

- ☐ *(कृपया स्पष्ट करें)* : ___________________
- ☐ आपको प्रश्न लागु नहीं करता (आप काम नहीं करते थे)

105. ~~*क्या आप बच्चे के जन्म के बाद काम पर लौट आए हो?~~

☐

☐

106. ~~*यदि आपका जवाब हाँ है, तो आप किस क्षेत्रमें काम करते है? (जैसे कि डॉक्टर, शिक्षक, डेटा की एंट्री करनेवाला क्लर्क, नर्सिंग होम एड, हौसकीपर, सब्जी उत्पादक, कपड़ा रंगाई मशीन ऑपरेटर, होटल क्लीनर, कॉल सेंटर में फोन करने वाले)~~

107. *कुलमिलाकर आपके पुरे परिवार का इनकम/ आय कितना होगा?

(स्थानीय सेटिंग्स के हिसाब से उचित मूल्यों को अंको में डालने और विकल्प को जोर से पढ़ें)

- ☐ < __$11,000____
- ☐ _$11,000____ से _$20,999_____ (बहुत कम)
- ☐ __$21,000____ से _$40,999_____ (कम)
- ☐ __$41,000___ से __$60,999____ (मध्यम)
- ☐ __$61,000___ से __$80,999____ (मध्यम उच्च)
- ☐ ≥ _$81,000__ (उच्च)

108. *उपर्युक्त इनकम/ आय, परिवार के कितने सदस्यों पर खर्च होता है? (नए बच्चे का खर्चा मिलाकर) _____________

109. *आप घर में सबसे आधिक किस भाषा (ओं) में संवाद करते हैं? _____________

110. *M कितनी अच्छी तरह आप इस देश की भाषा जानते हैं?

English	*बिना किसी कठिनाई के*	*ठीक ठाक*	कठिनाई के साथ	बिल्कुल नहीं
बोलना	☐	☐	☐	☐
पढना	☐	☐	☐	☐
लिखना	☐	☐	☐	☐
समझना	☐	☐	☐	☐

111. [M]कितनी अच्छी तरह आप इस देश की भाषा जानते हैं?

French

	बिना किसी कठिनाई के	*ठीक ठाक*	कठिनाई के साथ	बिल्कुल नहीं
बोलना	☐	☐	☐	☐
पढना	☐	☐	☐	☐
लिखना	☐	☐	☐	☐
समझना	☐	☐	☐	☐

112. *इससे हमारा यह साक्षात्कार समाप्त होता है| यदि आप इस साक्षात्कार में शामिल किये गए विषयों के अलावा कुछ और कहना या बताना चाहते है, तो हमें जरुर बताइये|
