# Supplementary material for: Migrant-friendly maternity care in Montreal, Canada: A cross-sectional study on migrant women’s care perspectives
Source: PLoS One. 2025 Aug 21;20(8):e0330830. doi: 10.1371/journal.pone.0330830 (PMC12370051; doi:10.1371/journal.pone.0330830)
Supplement: S6 Appendix — (PDF) [file pone.0330830.s006.pdf]

移民妇产护理问卷

采访指示

1. 在您采访参与人时，请明朗地读清每一题. 以下是一些采访注意事项：
   - 若问题指明要求，读出所有选项, 选择所有合适的答案，请清晰、明朗地读出每一个答案并让参与人对每一选项做出回答"是" 或"否"。
   - 若问题指明要求，让参与人独立作答,并选择所有合适的答案，请旁观，让参与人做出回答，然后从列出答案中选出符合的选项。若参与人无法独立作答，请暗示两三个选项作为提示。

2. 请遵守"跳到某题"的提示。

   例如:
   第 10 题问道：参与人有否想要使用但由于某些原因无法享用？紧接着第 11 题问道：请问您认为是哪些原因使您在怀孕期间没有得到某些您想得到的服务？请在参与人在第 10 问回答"有" 的基础上才问第 11 题。

3. 若采访问题需要参与人估算一段时间（例如: 第 2，6 题）,请提示参与人尽她所能地作答.

4. 若不确定参与人给予的答案选项（例如: 妊娠并发症）,请选"其他"并提供备注。

5. 若参与人有不理解的词汇，请积极地给与定义并解释:
   - **家庭计划：**计划怀孕的时间，并使用避孕药物
   - **性传染疾病：**由性交传染的疾病与感染
   - **麻醉：**使人失去局部或者全身感觉或者使人麻木的药物
   - **特殊新生照顾：**由于特殊医疗原因，新生讲被医保人员移到别处并受到特殊照料使母子不能呆在一起
   - **流产：**怀孕流失与在 20 周满之前
   - **终止怀孕：**在胎儿形成前终止怀孕；打胎
   - **移民状态**（第 93 问）：登录新国家的状态，而不是现在的状态 （若有不同）.
   - **移民拘留处：**(可用提示: 请问您有被移民局拘留吗？ 您有因为关于移民的任何原因而被扣留吗？）
   - **收入：**税前家庭总收入（包涵所有居住在一起并有收入的所有人）
   - **收入支持人数：**包括所有被此收入支持生活的人
   - **待产期：**临盆期，当阴部开始收缩时直到分娩开始

NOTES: (1) Questions marked with * (n=86) were identified (during a Delphi consensus process with international perinatal health research experts) as a minimum set of questions for use in international comparisons; (2) Questions marked with M are those only relevant for migrant women or identified as recommended migration indicators to capture in analyses of perinatal health (see Gagnon AJ, Zimbeck M, Zeitlin J. Migration and Perinatal Health Surveillance: An International Delphi Survey. *European Journal of Obstetrics & Gynecology and Reproductive Biology*. 2010;149(1), 37-43).

采访前言：研究项目大纲

我与一研究小组对于从外地来到加拿大的母亲在这个新环境里怀孕和妇产的经历十分关注. 接下来我将会问您一系列问题关于您怀孕, 临盆, 分娩, 产后的体验, 以及您总体经历的感受. 然后, 我将会问您一些关于您产科记录和移民历史的问题. 采访过程中, 您可以随时叫采访人重复读题, 或者提出在您不理解的地方让采访人进行解释. 我重复强调, 您在这次采访中提供的所有信息都将保持隐私. 您在研究采访的任何阶段都可以停止您的参与, 也可以选择不回答任何您不愿作答的提问。

任何时候您都可以向采访人提出疑问。 请问在开始前，您有什么问题吗？
那好, 我们开始吧!

MFMCQ 中文版			参与人编码:	
开始时间:			采访人民:	
结束时间:			采访时间:	

1. ***ᴹ 请问您出生在哪个国家?**

____

2. ***ᴹ 请问您总共在这里生活了多久?**

(若多次登录加拿大, 请估算所有在此居住的时间)

_____________ (天) _______ (星期) _______(月) _______ (年)

第一阶段：关于您在新国家最近一次怀孕的经历
（共 14 问）

3. ***ᴹ 请问您最近期的这次怀孕始于登录这里之前吗?**

- ☐ 是，我登陆时已经怀孕____星期了.
- ☐ 否
- ☐ 不知道/不记得了

4. ***请问您在最近期怀孕期间有得到医务人员的照料和护理吗? (例如: 医生, 护士 或 助产士)**

- ☐ 是，在_______________________________ （请说明在哪个国家）
- ☐ 否 *(请跳到第 8 题)*

5. *请问在新国家最近一次怀孕时，是谁为您提供的医疗服务?
（让参与人独立回答, 选择所有合适的答案。若需要，请给与提示）

- 家庭医生， 全科医生，普通医生
- 产科医生，妇科医生，妇产科医生
- 助产士
- 护士
- 其他（请说明）:_________
- 无法提供

---

6. *请问您第一次受到医疗服务时, 您怀孕了几个星期? _________ （星期）
来到新国家以后呢? _________ （星期）
(不算只有妊娠试验/验孕的医疗服务)

- 无法提供 (怀孕期间没有受到除了验孕以外的服务)

---

7. *请问您在怀孕期间看过几次医生，护士或助产士? ________（次）

- 无法提供 (怀孕期间没有看过医疗人士)

---

8. *请问您怀孕期间有没有医疗/妊娠并发症?

- **有（让参与人独立回答, 选择所有合适的答案）**
  - 贫血/贫血症
  - 高血压
  - 先兆子痫/子痫前期
  - 早产
  - 深部静脉血栓形成
  - 妊娠糖尿病
  - 胎盘前置/低置
  - 胎盘早剥
  - 尿路感染
  - 严重背痛
  - 胎膜早破
  - 抑郁症/精神忧郁
  - 其他（请说明）:__________________________（包括胎儿的症状）
- 没有, 您最近期怀孕没有医疗并发症

9. 请问您怀孕期间有使用这些服务?

	有	没有	不知道
妊娠班/分娩课	☐	☐	☐
预约看病	☐	☐	☐
食品发放	☐	☐	☐
住房寻找协助	☐	☐	☐
传统医疗、中医以及仪式	☐	☐	☐
家庭服务 (儿童看护, 心理辅导, 亲子课程)	☐	☐	☐
怀孕期间医疗测试 (例如: 全体检查, 血液检查, 宫颈检查/PAP 测试)	☐	☐	☐
出生缺陷筛查(例如: 唐氏综合症)	☐	☐	☐
超声波/超声波扫描检查	☐	☐	☐
支援服务 (例如: 心理健康服务)	☐	☐	☐
其他（请说明）: ___________________	☐	☐	☐

10. *请问以下哪些是您在怀孕期间,想得到但是没有享受到的服务

(读出所有选项, 选择所有合适的答案)

- ☐ 妊娠班/分娩课
- ☐ 预约看病
- ☐ 食品发放
- ☐ 住房寻找协助
- ☐ 传统医疗、中医以及仪式
- ☐ 家庭服务 (儿童看护, 心理辅导, 亲子课程)
- ☐ 怀孕期间医疗测试 (例如: 全体检查, 血液检查, 宫颈检查/PAP 测试)
- ☐ 出生缺陷筛查(例如: 唐氏综合症)
- ☐ 超声波/超声波扫描检查
- ☐ 支援服务 (例如: 心理健康服务)
- ☐ 其他（请说明）: ___________________
- ☐ 没有（请跳到第 12 题）

11. *请问哪些选项是使您无法得到那些服务的原因?

(让参与人独立回答并选择所有合适的答案)

- ☐ 附近没有提供我想要的服务
- ☐ 医疗服务人满了
- ☐ 不知道社区有提供这些服务
- ☐ 不知道我够资格得到这些服务
- ☐ 没有资格得到这些服务
- ☐ 不知道哪里有提供这些服务
- ☐ 害怕我的移民申请会受到影响
- ☐ 服务被提供商取消了
- ☐ 儿童看护没有被提供/没有儿童看护
- ☐ 由于有语言障碍
- ☐ 交通不是很方便
- ☐ 经济原因
- ☐ 由于有工作
- ☐ 没有时间
- ☐ 需要呆在家里
- ☐ 害怕医疗检验
- ☐ 我从亲戚或朋友那里得到了帮助
- ☐ 从医疗系统得到的帮助不如我的想象的那样
- ☐ 由于对医疗系统不了解从而对于使用它所提供的服务有困难
- ☐ 觉得不好意思
- ☐ 行政原因 (例如: 没有保险)
- ☐ 其他（请说明）: ________________
- ☐ 此题与我不相关

12. *请问在怀孕期间, 什么人, 媒体或者机构是您得到关于怀孕,待产和分娩最重要的信息来源?

(让参与人独立回答并选择所有合适的答案)

- ☐ 之前怀孕的经历
- ☐ 亲戚和朋友
- ☐ 信仰/精神领导
- ☐ 产科医生，妇科医生，妇产科医生
- ☐ 家庭医生， 全科医生，普通医生
- ☐ 助产师
- ☐ 护士
- ☐ 怀孕/亲子课程
- ☐ 书籍
- ☐ 电视节目
- ☐ 网上信息
- ☐ 其他（请说明）: ________________

13. *M 请问在怀孕期间, 新国家的医保人员有为您提供母语翻译服务吗?

- ☐ 有______________ （请说明）
- ☐ 没有

14. ***请问在这次怀孕期间直到待产之前, 您有足够了解这些关于这些方面的信息吗?**

	有	没有	不知道
怀孕期间, 身体的物理变化	☐	☐	☐
怀孕期间, 情绪上的变化	☐	☐	☐
临盆的征兆	☐	☐	☐
医药补助	☐	☐	☐
临盆和分娩时会发生什么	☐	☐	☐
无药疼痛治疗	☐	☐	☐
必要的医疗检查	☐	☐	☐
怀孕的营养调理	☐	☐	☐
您分娩后的自身健康以及恢复	☐	☐	☐
您会经历的情绪波动	☐	☐	☐
如何照顾您的新生孩子	☐	☐	☐
如何判断和认辩您孩子的健康以及成长问题	☐	☐	☐
母乳喂养	☐	☐	☐
奶粉喂养	☐	☐	☐
当您有对自身以及幼儿健康有疑问时能为您提供帮助的联系人信息	☐	☐	☐
家庭计划/计划生育	☐	☐	☐
艾滋病病毒和其他性传染疾病	☐	☐	☐

15. ***请问为您服务的医务人员有问您计划如何喂养您的孩子吗?**

- ☐ 有
- ☐ 没有
- ☐ 不知道/不记得了
- ☐ 无医务人员/没有得到医疗服务

16. ***请问为您服务的医务人员有没有问，在怀孕期间，您有没有顾虑您在照顾方面有某些个人偏好，或者任何特定的风俗与习惯**

- ☐ 有
- ☐ 没有
- ☐ 无医务人员

第二阶段：关于您在新国家最近一次待产以及分娩的经历

(共 16 问)

17. ***请问您分娩时是怀孕期的第几个星期?** __________(星期)

- ☐ 不知道

18. ***请问您生了几个孩子?** _______________ **(例如: 一胎，双胞胎，三胞胎。。)**

19. ***请问您新生的孩子有多重?**

________________(千克) _______________ （克）/ _______________ （磅） _______________ （盎司)
________________(千克) _______________ （克）/ _______________ （磅） _______________ （盎司)

~~**20.** ***请问您在哪里生的这个孩子?**~~

~~*(读出所有选项，选择一项最合适的答案)*~~

- ☐ ~~在医院病房~~
- ☐ ~~在医院手术室~~
- ☐ ~~在急诊室/急救室~~
- ☐ ~~诊所~~
- ☐ ~~分娩中心（医院以外）~~
- ☐ ~~在家里~~
- ☐ ~~其他（请说明）:________________________~~

21. ***请问哪些医保人员在您待产过程中给与您最多的照顾?**

(让参与人回答并选择一项最合适的答案)

- ☐ 产科医生，妇科医生，妇产科医生
- ☐ 家庭医生， 全科医生，普通医生
- ☐ 助产师
- ☐ 护士
- ☐ 其他（请说明）:________________________
- ☐ 无医保人员
- ☐ 无待产, 计划性的剖腹产
- ☐ 不知道

22. ***请问哪些医保人员在您分娩过程中给与您最多的照顾?**

(让参与人回答并选择一项最合适的答案)

- ☐ 产科医生，妇科医生，妇产科医生
- ☐ 家庭医生， 全科医生，普通医生
- ☐ 助产师
- ☐ 护士
- ☐ 其他（请说明）:________________________
- ☐ 无医保人员
- ☐ 不知道

23. ***待产以及分娩时, 请问您有经历任何以下所列的事项吗?**

	有	没有
引产术 (使子宫收缩)	☐	☐
增大引产术 (使子宫收缩幅度更大, 更频繁)	☐	☐
产钳 (分娩时用来取出新生胎儿的医用工具)	☐	☐
吸盘 (用真空抽吸来取出新生胎儿)	☐	☐
剖腹产	☐	☐
外阴切开术 (切开外阴口)	☐	☐
使用硬膜外麻醉来解除分娩疼痛	☐	☐
剖腹产使用脊髓麻醉	☐	☐
一般麻醉	☐	☐
其他（请说明）:_______________________	☐	☐

24.*请问在待产以及分娩时, 您有没有经历任何医学并发症?

(例如: 阴裂伤, 子宫破裂, 传染, 产后出血, 胎儿症状)

☐ 有, （请详细说明）:____

☐ 没有

若顺产, 请跳到第 26 题

25. ***若您是剖腹产, 请问主要是因为什么原因?**

（让参与人独立回答并选择一项最合适的答案)

☐ 是提前计划的, 考虑到医学原因从而医生推荐的

☐ 是提前计划的, 但不知道为什么

☐ 是提前计划的, 因为我想要剖腹产而不是为了医学原因

☐ 不是提前计划的, 但分娩的时间太长了

☐ 不是提前计划的, 但由于胎儿处于危险状态

☐ 不是提前计划的, 但是由于我处于危险状态

☐ 不是提前计划的, 而我也不知道是为了什么

☐ 其他（请说明）:_______________________

☐ 没有剖腹产

26.请问在待产时， 您有被允许自由移动或是选择更舒服的姿势吗?

(读出所有选项, 选择一项最合适的答案)

☐ 一直有

☐ 有时候有

☐ 很少有

☐ 没有, 因为医学原因

☐ 没有, 不知道为什么

☐ 无待产, 计划性的剖腹产

27. **请问待产时, 医务人员有问您想选择什么方式来减少分娩疼痛?**

- □ 有
- □ 没有
- □ 不知道 / 不记得了
- □ 无待产, 计划性的剖腹产

28. **请问待产时, 您对与医务人员对您应对疼痛的服务满意吗?**

- □ 有
- □ 没有
- □ 有时候有
- □ 无待产, 计划性的剖腹产

29. **请问待产时, 您有被允许家庭人员和/或支持者在场吗?**

- □ 有
- □ 没有
- □ 有时候有
- □ 无待产, 计划性的剖腹产

30. ***请问待产和分娩时, 您有陪伴人在身边吗?**

（读出所有选项, 选择一项最合适的答案）

- □ 一直有
- □ 有时候有
- □ 很少，基本没有
- □ 没有
- □ 不知道/不记得了

31. ***如果有, 请问是谁?**

(若多余一人, 请列出全部)

__________________________(与您的关系)

__________________________(与您的关系)

__________________________(与您的关系)

- □ 没有

32. ***请问为您服务的医保人员是否有询问您在待产或分娩期间有没有某些照顾方面的个人偏好，或者想遵循任何特定的风俗与习惯？（例如: 饮食讲究，水中分娩等）**

- □ 有
- □ 没有
- □ 没有，因为在被问之前我已经问过了

第三阶段：关于您在新国家最近一次产后的经历
(共 14 问)

33.*请问您的新生孩子需要受到特殊照顾而不能呆在您的身边吗?

(读出所有选项, 选择一项最合适的答案)

- □ 是, 在新生儿童症监护治疗房
- □ 是, 在儿童特殊护理病房
- □ 是, 在保育室
- □ 是, 但不知道/不记得在哪里
- □ 没有
- □ 不知道/不记得了

~~**34.请问在孩子出生后, 您在医院或是诊所待了多长时间?**~~

~~________ (小时) ________ (日) ________(/星期) ________ (月)~~

~~□ 此题与我不相关（例如：在家里）~~

~~**35.请问您认为您产后待留的时间…**~~

- ~~□ 太短~~
- ~~□ 正好~~
- ~~□ 太长~~
- ~~□ 不确定/无法说明~~

36.请问您在医院/诊所/分娩中心时，医务人员有没有询问您对食物的讲究？(例如: 食物的温度， 预备适应您宗教信仰的食物， 素食讲究， 或者其他)

- □ 有
- □ 没有
- □ 不知道/不记得了
- □ 此题与我不相关（例如：孩子不在医院出生等）

37. *请问为您服务的医保人员是否有询问您在产后有没有某些照顾方面的个人偏好，或者想遵循任何特定的风俗与习惯？（例如: 坐月子）

- □ 有
- □ 没有
- □ 不知道/不记得了

38.请问在产后的一小时内，您有肌肤对肌肤地抱过您的新生孩子吗?

- □ 有
- □ 没有（请说明原因）:________________________

39. *请问为您服务的医务人员是在什么时候告知、或者帮助您了解要如何母乳喂奶的呢?

(让参与人回答并选择一项最合适的答案)

- ☐ 产后的一小时内
- ☐ 不是马上，但在我离开医院/诊所/分娩中心之前
- ☐ 隔了些时日，在我复诊、或是检查的时候
- ☐ 他们没有告知、或者帮助我了解
- ☐ 不知道/不记得了
- ☐ 我没有想用母乳喂奶

40. *请问为您服务的医务人员有没有告知在您社区内关于母乳喂奶支持资源的信息呢?

- ☐ 有
- ☐ 没有，但我不需要这些信息 *(请跳到第 42 题)*
- ☐ 没有，虽然我需要这些信息 *(请跳到第 42 题)*
- ☐ 不知道/不记得了

~~**41. *若选“有”： 请问您有使用关于母乳喂奶的支持资源吗?**~~

- ~~☐ 有~~
- ~~☐ 没有（请说明原因）:________________________~~
- ~~☐ 此题与我不相关（没有给与这方面的信息）~~

~~**42. *请问产后，您或者您的孩子有曾因为这次怀孕到分娩过程中的任何原因 (包括定期检查) 去看医生吗?**~~

- ~~☐ 有~~
- ~~☐ 没有 *(请跳到第 45 题)*~~
- ~~☐ 不知道/不记得了~~

~~**43. *若选“有”: 请问为什么? ___________________________**~~

- ~~☐ 此题与我不相关（例如：没有去看医生）~~

~~**44. *若选“有”： 请问您看的是哪类医生/医保人员?**~~

~~*(让参与人回答并选择所有合适的答案)*~~

- ~~☐ 家庭医生， 全科医生，普通医生~~
- ~~☐ 急救室医生~~
- ~~☐ 产科医生，妇科医生，妇产科医生~~
- ~~☐ 助产师~~
- ~~☐ 护士~~
- ~~☐ 儿童医生~~
- ~~☐ 其他（请说明）:________________________~~
- ~~☐ 此题与我不相关 (例如：没有看医生/医保人员)~~

~~**45. *请问在产后，您有没有为自己或孩子想看医生但没看成?**~~

- ~~☐ 有~~
- ~~☐ 没有 *(请跳到第 47 题)*~~

~~**46.** *请问您看不成医保人员的原因是什么？~~

~~(让参与人独立回答并选择所有合适的答案)~~

- ☐ ~~医疗服务不在我的社区之内~~
- ☐ ~~医疗服务人满了~~
- ☐ ~~不知道医疗服务有被提供~~
- ☐ ~~不知道我有没有资格享用医疗服务~~
- ☐ ~~没有资格使用医疗服务~~
- ☐ ~~不知道哪里有提供医疗服务~~
- ☐ ~~害怕我的移民申请会受到影响~~
- ☐ ~~服务被提供商取消了~~
- ☐ ~~儿童看护没有被提供/没有儿童看护~~
- ☐ ~~由于有语言障碍~~
- ☐ ~~交通不是很方便~~
- ☐ ~~经济原因~~
- ☐ ~~由于有工作~~
- ☐ ~~没有时间~~
- ☐ ~~需要呆在家里~~
- ☐ ~~害怕医疗检验~~
- ☐ ~~我从亲戚或朋友那里得到了帮助~~
- ☐ ~~从医疗系统得到的帮助不如我的想象的那样，或者由于对医疗系统不了解从而对于使用它所提供的服务有困难~~
- ☐ ~~觉得不好意思~~
- ☐ ~~行政原因 (例如: 没有保险)~~
- ☐ ~~其他, (请说明):~~ ________________
- ☐ ~~不知道/不记得了~~

第四阶段：您最近期在新国家的总体妇产医疗经历

（共 20 问）

47. **现在想想看，还有没有其他您想使用、或者得到的服务和信息？**

____

____

____

48. ***总体来讲，请问为您服务的医务人员有让您感觉到温暖并容易接触吗？**

a) **怀孕期间**

- ☐ 一直
- ☐ 有时候
- ☐ 很少
- ☐ 从来没有

b) **待产、分娩期间**

- ☐ 一直
- ☐ 有时候
- ☐ 很少
- ☐ 从来没有

c) **产后**

- ☐ 一直
- ☐ 有时候
- ☐ 很少
- ☐ 从来没有

49. ***总体来讲，请问为您服务的医务人员尊重您吗?**

a) **怀孕期间**

- ☐ 一直
- ☐ 有时候
- ☐ 很少
- ☐ 从来没有

b) **待产、分娩期间**

- ☐ 一直
- ☐ 有时候
- ☐ 很少
- ☐ 从来没有

c) **产后**

- ☐ 一直
- ☐ 有时候
- ☐ 很少
- ☐ 从来没有

50. ***总体来讲，请问为您服务的医务人员有帮助到您吗?**

a) **怀孕期间**

- ☐ 一直
- ☐ 有时候
- ☐ 很少
- ☐ 从来没有

b) **待产、分娩期间**

- ☐ 一直
- ☐ 有时候
- ☐ 很少
- ☐ 从来没有

c) **产后**

- ☐ 一直
- ☐ 有时候
- ☐ 很少
- ☐ 从来没有

51. *总体来讲，您对您所得到的医疗服务很满意。

a) 怀孕期间

- □ 一直
- □ 有时候
- □ 很少
- □ 从来没有

b) 待产、分娩期间

- □ 一直
- □ 有时候
- □ 很少
- □ 从来没有

c) 产后

- □ 一直
- □ 有时候
- □ 很少
- □ 从来没有

---

52. *请问在怀孕、待产、或分娩期间，为您服务的医务人员有没有要求您做您不想做的事?

- □ 有
- □ 没有
- □ 不知道/不记得了

---

53. 若是，请问医务人员要求您做了什么?

____

- □ 此题与我不相关

---

54. 请问医务人员有没有问您想要被什么性别的医务人员服务?

a) 怀孕期间

- □ 一直
- □ 有时候
- □ 很少
- □ 从来没有

注释________________

b) 待产、分娩期间

- □ 一直
- □ 有时候
- □ 很少
- □ 从来没有

注释___________

c) **产后的一天内**

- □ 一直
- □ 有时候
- □ 很少
- □ 从来没有

注释_______________

55. *请问您有理解医务人员为您提供的信息吗?

a) **怀孕期间**

- □ 一直
- □ 有时候
- □ 很少
- □ 从来没有

注释_______________

b) **待产、分娩期间**

- □ 一直
- □ 有时候
- □ 很少
- □ 从来没有

注释_______________

c) **产后的一天内**

- □ 一直
- □ 有时候
- □ 很少
- □ 从来没有

注释_______________

56. *M 请问若医务人员以另外一门语言为您提供信息，会帮助您更好的理解吗?

- □ 是, 请问哪门语言_________________ (例如：您的母语)
- □ 否
- □ 不知道/不记得了

57. *M 请问医务人员有为您提供口译服务吗?

a) **怀孕期间**

- □ 有
- □ 没有
- □ 不相关

b) **待产、分娩期间**

- □ 有
- □ 没有
- □ 不相关

c) **产后的一天内**

- □ 有
- □ 没有
- □ 不相关

58. *ᴹ **请问经常有说您母语的人帮您做口译吗?**

a) **怀孕期间**

- □ 一直
- □ 有时候
- □ 很少
- □ 从来没有
- □ 不相关

b) **待产、分娩期间**

- □ 一直
- □ 有时候
- □ 很少
- □ 从来没有
- □ 不相关

c) **产后的一天内**

- □ 一直
- □ 有时候
- □ 很少
- □ 从来没有
- □ 不相关

59. *ᴹ **请问是谁帮您做口译的呢?**

(读出所有选项, 选择所有合适的答案)

a) **怀孕期间**

- □ 丈夫/伴侣
- □ 亲戚/朋友
- □ 保健医生
- □ 孩子
- □ 专业翻译人员
- □ 其他病人、或者其他病人的亲戚/朋友
- □ 其他, (*请说明*) :__________________
- □ 不相关

b) **待产、分娩期间**

- □ 丈夫/伴侣
- □ 亲戚/朋友
- □ 保健医生
- □ 孩子
- □ 专业翻译人员
- □ 其他病人、或者其他病人的亲戚/朋友
- □ 其他, (*请说明*) :__________________
- □ 不相关

c) **产后的一天内**

- □ 丈夫/伴侣
- □ 亲戚/朋友
- □ 保健医生
- □ 孩子
- □ 专业翻译人员
- □ 其他病人、或者其他病人的亲戚/朋友
- □ 其他, (*请说明*) :__________________
- □ 不相关

60. *M **请问您对他（她）的口译满意吗?**

- □ 满意
- □ 不满意
- □ 不知道/不记得了
- □ 不相关

61. ***请问在您怀孕、分娩和产后过程中，您有没有关于照顾方面的某些个人偏好，或者像遵循任何风俗与习惯，但医保人员没有考虑/安排或者没有允许呢?**

- □ 有
- □ 没有 (请跳到第６４题)
- □ 不知道/不记得了

62. 若选“有”：请问都有哪些个人喜好或习俗?

*i)*___________

*ii)*__________

*iii)*__________

- □ 此题与我不相关

63.若选“有”：请问医务人员说明了哪些原因使您无法遵守您的喜好, 习俗, 或者传统?

*i)*___________

*ii)*__________

*iii)*__________

- □ 此题与我不相关

64. ***请问您认为医务人员可以在这方面做出改变、或者做得更好吗?**

a) **怀孕期间?**

- □ 是 *(请在第 65 题 a 部分注释)*
- □ 否
- □ 不知道/不记得了

b) **待产、分娩期间?**

- □ 是 *(请在第 65 题 b 部分注释)*
- □ 否
- □ 不知道/不记得了

c) **产后?**

- □ 是 *(请在第 65 题 c 部分注释)*
- □ 否
- □ 不知道/不记得了

65.若上题任何一项选“是”, 请说明您认为要做出什么改变、或者怎样更好:

a) 怀孕期间

____

b) 待产、分娩期间

____

c) 产后

____

66. *请形容您这次怀孕, 待产, 或产后:

a) 令我愉快/满意的是:

____

b) 令我不愉快/不太满意的是:

____

根据您自己最近一次怀孕经历，请问以下 11 陈述是否属实?

67. *医务人员有问我是否有任何问题

- □ 一直
- □ 有时候
- □ 很少
- □ 从来没有

68. 医务人员很匆忙、或者在赶时间

- □ 一直
- □ 有时候
- □ 很少
- □ 从来没有

69. *我认为我的担心和疑问有被医务人员正视

a) 怀孕期间

- □ 一直
- □ 有时候
- □ 很少
- □ 从来没有
- □ 此题与我不相关(例如：没有得到怀孕期间的医疗照顾)

b) 待产、分娩期间

- □ 一直
- □ 有时候
- □ 很少
- □ 从来没有
- □ 此题与我不相关(例如：没有医务人员在场)

c) 产后

- □ 一直
- □ 有时候
- □ 很少
- □ 从来没有
- □ 此题与我不相关(例如：没有医务人员在场)

70. 我需要等很久才能得到医疗照顾

a) 怀孕期间

- □ 一直
- □ 有时候
- □ 很少
- □ 从来没有
- □ 此题与我不相关(例如：没有得到怀孕期间的医疗照顾)

b) 待产、分娩期间

- □ 一直
- □ 有时候
- □ 很少
- □ 从来没有
- □ 此题与我不相关(例如：没有医务人员)

c) 产后

- □ 一直
- □ 有时候
- □ 很少
- □ 从来没有
- □ 此题与我不相关(例如：没有医务人员)

71. *医务人员有告知我发生了什么，或者情况变化

a) 怀孕期间

- □ 一直
- □ 有时候
- □ 很少
- □ 从来没有
- □ 此题与我不相关(例如：没有得到怀孕期间的医疗照顾)00

b) 待产、分娩期间

- □ 一直
- □ 有时候
- □ 很少
- □ 从来没有
- □ 此题与我不相关(例如：没有医务人员)

c) 产后

- □ 一直
- □ 有时候
- □ 很少
- □ 从来没有
- □ 此题与我不相关(例如：没有医务人员)

---

72. *我觉得能够很直接地问我不了解的事情

a) 怀孕期间

- □ 一直
- □ 有时候
- □ 很少
- □ 从来没有
- □ 此题与我不相关(例如：没有得到怀孕期间的医疗照顾)

b) 待产、分娩期间

- □ 一直
- □ 有时候
- □ 很少
- □ 从来没有
- □ 此题与我不相关(例如：没有医务人员)

c) 产后

- □ 一直
- □ 有时候
- □ 很少
- □ 从来没有
- □ 此题与我不相关(例如：没有医务人员)

---

73. *医务人员有在我不知情的情况下、或者未遵守我的意愿而为我做出决定

a) 怀孕期间

- □ 一直
- □ 有时候
- □ 很少
- □ 从来没有
- □ 此题与我不相关(例如：没有得到怀孕期间的医疗照顾)

b) 待产、分娩期间

- □ 一直
- □ 有时候
- □ 很少
- □ 从来没有
- □ 此题与我不相关(例如：没有医务人员)

c) 产后

□ 一直
□ 有时候
□ 很少
□ 从来没有
□ 此题与我不相关(例如：没有医务人员)

74. *医务人员会鼓励我、以及令我安心

a) 怀孕期间

□ 一直
□ 有时候
□ 很少
□ 从来没有
□ 此题与我不相关(例如：没有得到怀孕期间的医疗照顾)00

b) 待产、分娩期间

□ 一直
□ 有时候
□ 很少
□ 从来没有
□ 此题与我不相关(例如：没有医务人员)

c) 产后

□ 一直
□ 有时候
□ 很少
□ 从来没有
□ 此题与我不相关(例如：没有医务人员)

75. *医务人员有花足够的时间对您提供解释吗?

a) 怀孕期间

□ 一直
□ 有时候
□ 很少
□ 从来没有
□ 此题与我不相关(例如：没有得到怀孕期间的医疗照顾)00

b) 待产、分娩期间

□ 一直
□ 有时候
□ 很少
□ 从来没有
□ 此题与我不相关(例如：没有医务人员)

c) 产后

- □ 一直
- □ 有时候
- □ 很少
- □ 从来没有
- □ 此题与我不相关 (例如：没有医务人员)

76. ***总体而言，您认为医务人员对您与他人的对待有区别吗？(例如：因为您的语言或口音、文化、种族、肤色、宗教信仰、移民状态、或者健康保险状况)**

- □ 总是有 *(请在第 77 题说明原因)*
- □ 有时候有 *(请在第 77 题说明原因)*
- □ 很少有 *(请在第 77 题说明原因)*
- □ 从来没有 *(请跳到第 78 题)*

77. ***若第 76 题选“有”：请问您认为是因为那些原因？**

（让参与人独立回答, 选择所有合适的答案）

- □ 语言或口音
- □ 文化
- □ 种族/民族背景
- □ 肤色
- □ 信仰
- □ 移民状态
- □ 健康保险状况
- □ 其他原因，（请说明）: __________
- □ 此题与我不相关

第五阶段：您的产科历史（共 8 问）

78. ***请问您总共怀孕过几次(包括这次)?_______**

79. ~~*请问有几次怀孕为流产？ _______~~

~~□ 此题与我不相关~~

~~请确保问此题时，没有外人在场~~

~~**80.** *请问有几次怀孕被终止了（打胎）？ ________~~

~~☐ 此题与我不相关~~

~~**81.** *请问有几次怀孕是死胎？ ________~~

~~☐ 此题与我不相关~~

---

82. *请问您共有几胎是生在怀孕 37 周满之内? _______

☐ 此题与我不相关

---

83. *请问您共有几胎是生在怀孕 37 周满之后? _______

☐ 此题与我不相关

---

84. *请问您在之前的怀孕期间有没有任何医疗/妊娠并发症?

☐ 有

☐ 没有 *(请跳到第 86 题)*

☐ 此题与我不相关*(请跳到第 86 题)*

---

85. *若您在之前怀孕期间内有医疗并发症，请问都有些什么?

(让参与人独立回答, 选择所有合适的答案)

☐ 剖腹产/剖宫产

☐ 贫血

☐ 高血压

☐ 子痫前期（妊娠期高血压）

☐ 早产

☐ 深静脉血栓

☐ 妊娠糖尿病

☐ 前置胎盘

☐ 胎盘早剥

☐ 尿路、尿道感染

☐ 严重背痛

☐ 胎膜早破

☐ 情绪忧郁、低落

☐ 其他，*（请说明）*: ______________________

☐ 不知道

最后阶段: 关于您和您的家庭
(共 27 问)

86. *请问您的婚姻状况是什么?

- □ 已婚
- □ 未婚同居
- □ 寡居
- □ 分居
- □ 离婚
- □ 单身

87. *请问您现在与谁居住?

	是	不是
丈夫/男性伴侣	☐	☐
女性伴侣	☐	☐
您的父亲/母亲	☐	☐
您的兄弟/姐妹	☐	☐
伴侣的父亲/母亲	☐	☐
伴侣的兄弟/姐妹	☐	☐
朋友	☐	☐
孩子(除了新生孩子以外)	☐	☐
其他, (请说明): _______________	☐	☐
没有其他人, 只是我与新生孩子	☐	☐
没有其他人, 我一人居住	☐	☐

88. *请问有几个您的孩子正和您一起居住? (包括您的新生孩子) ____________

89. *ᴹ请问有几个您的孩子是在新国家出生的? (包括您的新生孩子) ____________

90. *请问您的生日是 ________________(月) __________(年)

91. *ᴹ请问您的母亲是在哪个国家出生的? ____________

92.*ᴹ请问您的父亲是在哪个国家出生的？ ____________

由于我们的研究非常关注国际移民来到这个国家的经历，接下来的问题会问到您的移民状态。您提供的信息将不会被告知与移民部门，也不会影响到您的移民, 难民，或市民申请。

93.*ᴹ请问您的移民状态是什么？

（让参与人独立回答, 选择一项合适的答案）

- □ 移民 (永久居民)
- □ 难民
- □ 难民申请人/庇护寻求者
- □ 临时工人/住家保姆
- □ 临时居民
- □ 学生
- □ 探亲/游客
- □ 无状态
- □ 无证/无记录
- □ 公民
- □ 其他，（请说明）: _________________

94.ᴹ请问你持有这状态多久了？ ________(日) ________ (星期) ________(月) ________ (年)

95.ᴹ请问在来到这里以后，您的移民状态有改变吗?

- □ 有
- □ 没有 *(请跳到第 97 题)*

96.*ᴹ若选“是”,请问您之前的移民状态是什么?

- ☐ 移民 (永久居民)
- ☐ 难民
- ☐ 难民申请人/庇护寻求者
- ☐ 临时工人/住家保姆
- ☐ 临时居民
- ☐ 学生
- ☐ 探亲/游客
- ☐ 无状态
- ☐ 无证/无记录
- ☐ 其他, （请说明）: ________________
- ☐ 此题与我不相关 (例如：没有改变状态)

97.ᴹ请问您有过难民身份吗?

- ☐ 有
- ☐ 没有
- ☐ 不知道/不记得了

98.ᴹ请问您有没有没扣留在移民拘留中心?

- ☐ 有
- ☐ 没有 *(请跳到第 101 题)*

99.ᴹ若选“有”,请问您没扣留了多久? _____ (日) ______ (星期) _______(月) _______ (年)

- ☐ 此题与我不相关

100. *ᴹ若选“有”, 请问您是否有在怀孕期间被扣留?

- ☐ 有
- ☐ 没有
- ☐ 此题与我不相关

101. *请问主要是谁支付您的医疗健康服务?

	是	*不是*	*不知道*
公费医疗保险	☐	☐	☐
私人医疗保险	☐	☐	☐
政府资助健康保险 （为难民和庇护寻求者）	☐	☐	☐
自己支付	☐	☐	☐

102. *请问您完成的最高教育是什么?

- □ 小学毕业
- □ 高中毕业
- □ 高中后文凭(例如: 贸易学校, 大专, 大学)
- □ 研究生文凭 (硕士、博士)
- □ 无

103. M 请问您有资格在加拿大工作吗?

- □ 有
- □ 没有
- □ 不知道

104. *请问在您孩子出生前, 您最后的有薪工作是什么? (例如: 医生, 教师, 数据录入员, 敬老院援助, 管家, 农业/农民, 纺织印染机械操作员, 酒店清洁员, 呼叫中心服务员)

(请说明): ___________________

- □ 没有工作

~~**105.** *请问在您孩子出生后, 你已经返回您的工作岗位了吗?~~

- ~~□ 是~~
- ~~□ 否~~

~~不论选项, 请说明原因___________ (若选"否"请跳到第 107 题)~~

~~**106.** *若选"是", 请问您现在的工作职位是什么? (例如: 医生, 教师, 数据录入员, 敬老院援助, 管家, 农业/农民, 纺织印染机械操作员, 酒店清洁员, 呼叫中心服务员)~~

~~*(请说明)*: ___________________~~

- ~~□ 此题与我不相关~~

107. *请问您的家庭总体来讲(在税前) 属于哪个收入组?

(请在括号内输入本地的收入值, 并读出所有选项)

- □ <\$11,000 (非常低)
- □ \$11,000 to \$20,999 (低)
- □ \$21,000 to \$40,999 (中等)
- □ \$41,000 to \$60,999 (中等偏高) □ \$61,000 to \$80,999
- □ >\$81,000 (高)

108. *请问您的收入用来支持几个人的生活? (包括新生孩子)? ______________

109. *请问您在家使用最多的语言是什么?

____

110. *ᴹ请问您知道多少这个国家的语言?

~~(请在这里输入相关的本地语言)~~

语言: English

	流利	*不错*	*有困难*	*完全不会*
口语	☐	☐	☐	☐
阅读	☐	☐	☐	☐
写作	☐	☐	☐	☐
理解	☐	☐	☐	☐

~~请在本地语言多于一门的情况下问此题~~

111. ᴹ请问您知道多少这个国家的语言?

~~(请在这里输入相关的本地语言)~~

语言: French

	流利	*不错*	*有困难*	*完全不会*
口语	☐	☐	☐	☐
阅读	☐	☐	☐	☐
写作	☐	☐	☐	☐
理解	☐	☐	☐	☐

112. *以上是所有的问题，我们的采访就此结束。 请问您在我们的问卷主题以外想要添加些什么吗? 或者其他的信息需要我们知道的吗?
