# Supplementary material for: Migrant-friendly maternity care in Montreal, Canada: A cross-sectional study on migrant women’s care perspectives
Source: PLoS One. 2025 Aug 21;20(8):e0330830. doi: 10.1371/journal.pone.0330830 (PMC12370051; doi:10.1371/journal.pone.0330830)
Supplement: S8 Appendix — (PDF) [file pone.0330830.s008.pdf]

Migrant Friendly Maternity Care Questionnaire

انٹرویو کرنے والے کے لیے ہدایات

1. جب آپ انٹرویو لیں تو خاتون کو سب سوال آرام سے اور واضح طور پر پڑھ کر سنائیں. جوابات کے لیے نیچے دی گئ ہدایات کو پڑھ کر ان پر عمل کریں.

- جن سوالوں کے نیچے یہ لکھا ہے (اونچا پڑھ کر تمام متعلقہ پر نشان لگائیں) ان سب کو باری باری پڑھ کر سنائیں اور خاتون کو ہاں یا نہ میں ہر سوال کا جواب دینے دیں
- جن سوالوں کے نیچے یہ لکھا ہے *(ماں کے جوابات کے مطابق نشان لگائیں)* ان کے صرف سوال پڑھ کر سنائیں، جوابات نہ سنائیں اور خود یہ فیصلہ کریں کہ کہ کونہ جواب، خاتون کے جواب سے زیادہ ملتا جلتا ہے. اگر خاتون کو کوئی جواب سمجھ نہیں آ رہا تو ایک یا دو جوابات مثال کے طور پر پڑھ کر سنائیں.

2. سوال چھوڑ کر کسی اگلے سوال پر جانے کی ہدایات پر عمل کریں. یعنی اگر کسی سوال کا جواب *ہاں* یا *نہ* میں ہے اور اگلا سوال *اگر ہاں* یا *اگر نہ* سے شروع ہوتا ہے تو یہ سوال صرف اس صورت میں پوچھیں اگر پہلے سوال کا جواب، اس سوال کے آغاز سے ملتا ہے.

مثلا
سوال نمبر 10 کسی ایسی سہولت کا بارے میں پوچھتا ہے جو خاتون استعمال کرنا چاہتی تھیں مگر نہیں کر پائیں. سوال نمبر 11 " اگر آپ کو مطلوبہ سہولت نہیں ملی تو او کی کیا وجہ ہے" صرف اس صورت میں پوچھیں اگر خاتون نے سوال نمبر 10 کا جواب *ہاں* میں دیا ہو.

3. ایسے سوال جو عرصے کے بارے میں پوچھتے ہیں (مثلا سوال نمبر 2، 6، 7) ان کے لیے خاتون سے انکا بہترین اندازا بتانے کو کہیں

4. کچھ ایسے سوال ہیں جو خاتون سے کسی بات پر تبصرہ کرنے کو کہتے ہیں. ایسے سوالوں کے جوابات کو ضرورت پڑنے پر ایک علیحدہ صفحہ پر لکھیں لیکن یاد سے ان پر سوال نمبر لکھ کر سوالنامے کے ساتھ لگا دیں.

5. ایسے لفظ جو خاتون کو سمجھ نہ آیں، ان کے لیا مندرجہ ذیل تشریحات استعمال کریں:

- Family planning: بچہ پیدا کرنے کا وقت متعین کرنے کے لیے خاندانی منصوبہ بندی کے طریقے استعمال کرنا
- Sexually transmitted infections: ہم بستری کی وجہ سے ایک فریق کی بیماری کا دوسرے کو لگنا
- Anaesthesia: کسی حصّے کو سن کرنے کے لیے یا مکمل بیہوشی کے لیے دی جانے والی دوائی
- Special care baby unit: بچے کو ماں سے علیحدہ کسی جگہ پر رکھا گیا کیونکہ اسے خاص طبّی نگہداشت کی ضرورت تھی.
- Miscarriage: حمل کا پہلے 20 ہفتوں سے پہلے ختم ہو جانا
  Terminated (pregnancy): بچے کے مکمّل ہونے سے پہلے بچہ گرا دینا
- (Q93) Immigration status : اس ملک میں آنے کے بعد پہلے دن سے شروع ہوتا ہے. کاغذات وصول کرنے والے دن نہیں.
- Immigration detention centre: ممکن تشریحات: کیا امیگریشن کی وجہ سے آپ کو نظربند یا قید رکھا گیا ؟
- Income: گھر میں رہنے والے ہر فرد کی آمدنی (مثلا بہن ، شوھر، ماں) ٹیکس سے پہلے
- How many does it support: اس میں بہن اور بچے وغیرہ سب شامل ہیں

6. جب یہ معلوم ہو جائے کہ خاتون کے لیے اصل ماہر صحت کون تھا/ تھی(مثلا ڈاکٹر یا نرس) تو اس کے بعد صرف اسی کا نام لیں

NOTES: (1) Questions marked with * (n=86) were identified (during a Delphi consensus process with international perinatal health research experts) as a minimum set of questions for use in international comparisons; (2) Questions marked with M are those only relevant for migrant women or identified as recommended migration indicators to capture in analyses of perinatal health (see Gagnon AJ, Zimbeck M, Zeitlin J. Migration and Perinatal Health Surveillance: An International Delphi Survey. *European Journal of Obstetrics & Gynecology and Reproductive Biology*. 2010;149(1), 37-43).

انٹرویو کا آغاز: ماؤں کیلئے پراجیکٹ کا خلاصہ

مین ایک ریسرچ ٹیم کے ساتھ کام کر رہی ہوں جو مہاجر خواتین کے نئے ملک مین زچگی کے تجربے کے بارے مین جاننا چاھتی ھے۔ مین آپ سے آپکے حمل، لیبر، بچے ک پیدائش کے بعد کے وقت اور آپکے مجموعی زچگی کے تجربے کے بارے مین سوالات پوچھوں گی۔ پھر آپ سے آپکے مخصوص مسائل (حمل اور زچگی) اور امیگریشن کے بارے مین سوالات ہوں گے۔ انٹرویو کے دوران آپ کسی بھی وقت مجھ سے سوال ھرانے کا یا سامجھانے کا کہ سکتی ہیں۔ مین اس بات کی دوبارہ وضاحت کر دینا چاھتی ہوں کہ آپکی تمام معلومات اور جوابات خفیہ رہیں گے۔ اگر آپ کسی سوال کا جواب دینا مناسب نا سمجھین تو آپ وہ چھوڑ سکتی ہیں اور اس تحقیق میں شامل رہنے سے کسی بھی وقت انکار کر سکتی ہیں۔

آپ کسی بھی وقت مجھ سے سوال پوچھ سکتی ھیں۔ انٹرویو شروع کرنے سے پہلے آپ کوئی سوال پوچھنا چاھیں گی؟

چلیں شروع کرتے ہیں!

MFMCQ اردو ورژن		مطالعہ کا شناختی نمبر:	
شروع کرنے کا وقت		انٹرویو لینے والے کا نام	
ختم کرنے کا وقت		انٹرویو کی تاریخ	

.1 **M* آپ کس ملک میں پیدا ہوئیں؟**

____

.2 **M * آپ اس ملک میں کب سے رہ رہی ہیں؟**

_______________ (دن) _______________ (ہفتے) _____________ (ماہ) ___________ (سال)

(اس ملک میں گزارا ہوا تمام وقت۔ اکثر لوگ مائیگریشن سے پہلے آتے جاتے رہتے ہیں)

پہلے چند سوالاتؔ اس ملک میں آپکے حالیہ حمل کے بارے میں ہیں۔ اس حصّے میں 14 سوال ہیں۔

.3 **آپ جب اس ملک میں آئیں تو اپنے حالیہ بچے سے حاملہ تھیں؟ M**

- ☐ ________جی۔ آپکاحمل کتنے ہفتے کا تھا؟
- ☐ نہیں
- ☐ معلوم نہیں

.4 ***اس حمل کے دوران کسی ماہر صحت (مثلا ڈاکٹر، نرس یا دایٔ وغیرہ) نے آپ کی دیکھ بھال کی؟**

- ☐ جی
- ☐ نہیں- (سوال نمبر 8 پر جایٔے)

.5 * **حمل کے دوران کس نے آپ کی نگہداشت کی؟**
(ماں کے جوابات کے مطابق متعلقہ پر نشان لگائیں)

- ☐ فیملی ڈاکٹر،جنرل پریکٹیشنر GP
- ☐ ماہر زچگی، گائنیکولوجسٹ، OBGYN
- ☐ دائ
- ☐ نرس پریکٹیشنر / نرس
- ☐ ان کے علاوہ (برائےمہربانی وضاحت کیجئے) ___________________
- ☐ لاگو نہیں ہوتا N/A

.6 **اس حمل میں پہلی دفع جب آپکو نگہداشت ملی، تو آپکا حمل کتنے هفتے کا تھا ؟ ___________(ہفتے)***
(حمل کے ٹیسٹ کو شامل نا کریں)

- ☐ لاگو نہیں ہوتا (اس زچگی کے دوران کوئ نگہداشت نہین ملی)

.7 **اس حمل کے دوران اپ ڈاکٹر یا نرس وغیرہ سے کتنی دفع ملیں؟ __________________**

- ☐ لاگو نہیں ہوتا (اس زچگی کے دوران کوئ نگہداشت نہین ملی)

.8 **اس حمل کے دوران آپ کو کوئ میڈیکل (طبی) مسایٔل پیش اے ؟**

- ☐ جی *(ماں کے جوابات کے مطابق نشان لگائیں)*
  - ☐ خون کی کمی(انیمیا)
  - ☐ هاي بلڈ پریشر .(بلند فشار خون)
  - ☐ پری اکلامپسیا (حمل کے دوران های بلڈ پریشر)
  - ☐ قبل از وقت لیبر
  - ☐ خون کی نالی میں رکاوٹ (deep vein thrombosis)
  - ☐ حمل کے دوران زیابطیس
  - ☐ حم میں نالی کا نیچے پیوست ہونا(placenta praevia)
  - ☐ رحم مین نالی کا اکھڑ جآنا (placental abruption)
  - ☐ پیشاب کی نالی میں انفیکشن
  - ☐ کمر کا شدید درد
  - ☐ قبل از وقت پانی کی تھیلی کا پھٹ جانا
  - ☐ ڈپریشن (نفسیاتی دباؤ)
  - ☐ ان کے علاوہ (برائےمہربانی وضاحت کیجئے) ___________________
- ☐ نہیں۔ اس حمل میں آپکو کوئّ طبی مسلہ پیش نہیں آیا (نومولود کی کوئی پیچیدگی شامل کرتے ہوے)

9. اس حمل کے دوران آپ نے ان میں سے کونسی سہولیات حاصل کیں؟

	جی	نہیں
حمل اور بچے کی پیدائش کے متعلق کلاسیں لیں	☐	☐
کسی ماہر صحت سے رابطہ	☐	☐
لنگر خانہ (فوڈ بینک) (ایسے ادارے جو مفت کھانے پینے کی اشیا فراہم کرتے ہیں)	☐	☐
گھر ڈھونڈنے میں مدد	☐	☐
روایتی علاج یا رسمیں	☐	☐
خاندان کیلئے سھولیات مثلاً بچوں کی ماہری، والدین کی تربیت کے کورس، مشاورت وغیرہ	☐	☐
حمل کے دوران میڈکل ٹیسٹ (مثلاً پورا جسمانی چیک اپ، خون ٹیسٹ، سروکس کا معائینہ، PAP ٹیسٹ)	☐	☐
پیدایشی عیب کی نشاندہی (ڈاوٗن سنڈروم وغیرہ)	☐	☐
الٹراساوٗنڈ سکین	☐	☐
مددگار سہولیات (ذہنی صحت کی سہولیات)	☐	☐
ان کے علاوہ (*براۓمہربانی وضاحت کیجئے*) ___________________	☐	☐

10. اس حمل کے دوران کیا آپ ان میں سے کوئ سہولت استعمال کرنا چھاھتی تھیں لیکن نہیں کر پایں؟
(اونچا پڑھیے اور جو لاگو ہوتی ہیں ان سب پر نشان لگائیں)

- ☐ زچگی / بچے کی پیدائش کے مطلق کلاسیں
- ☐ کسی ماہر صحت سے ملاقات
- ☐ لنگر خانہ (فوڈ بینک)
- ☐ گھر ڈھونڈنے میں مدد
- ☐ روایتی علاج یا رسمیں
- ☐ خاندان کیلئے سھولیات مثلاً بچوں کی نگرانی ، والدین کی تربیت کے کورس، مشاورت وغیرہ
- ☐ حمل کے دوران میڈکل ٹیسٹ (مثلاً پورا جسمانی معائینہ، خون ٹیسٹ، سروکس کا موایٗنا، PAP ٹیسٹ)
- ☐ پیدایشی عیب کی نشاندہی (ڈاوٗن سنڈروم وغیرہ)
- ☐ الٹراساوٗنڈ سکین
- ☐ مددگار سہولیات (ذہنی صحت کی سہولیات)
- ☐ ان کے علاوہ (براۓمہربانی وضاحت کیجئے) ___________________

نہیں (سوال نمبر 12 پر جائیے)

11. *** اگر آپکو دوران حمل مطلوبہ سہولیات میسر نہیں ہوئیں تواس میں کیا رکاوٹیں تھیں؟**
(ماں کے جوابات کے مطابق مندرجہ ذیل پر نشان لگائیں)

- ☐ آپکے علاقے میں سہولت میسّر نہیں تھی
- ☐ مزید مریضوں کی گنجائش نہیں تھی
- ☐ آپکو اس سہولت کا علم نہیں تھا
- ☐ آپکو معلوم نہیں تھا کہ آپ ان سہولیات کی اھل ھیں
- ☐ آپ ان سہولیات کی اھل نہیں تھیں
- ☐ آپکو معلوم نہیں تھا کہ یہ سہولیات کہاں فراھم کی جاتی ھیں
- ☐ آپکو ڈر تھا کہ اپکی امیگریشن متاثر ہو گی
- ☐ سہولت فراہم کرنے والے ادارے نے اپکی ملاقات منسوخ کر دیٗ تھی
- ☐ بچے کی نگرانی کی سہولت میسر نہیں تھی
- ☐ زبان کی رکاوٹ تھی
- ☐ آپکی ٹرانسپورٹ تک رسائی نہیں تھی
- ☐ کوئی مالی وجہ تھی
- ☐ آپ کام میں مصروف تھیں
- ☐ آپ کے پاس وقت نہیں تھا
- ☐ آپکا گھر پر ہونا ضروری تھا
- ☐ آپ کو ٹیسٹ کرانے سے ڈر لگتا تھا
- ☐ آپکو فیملی اور دوستوں سے مدد مل گیّ تھی
- ☐ آپ کو آپکی توقع کے مطابق نگہداشت نہیں ملی۔
- ☐ اپ کو یہ سمجھنے میں مشکل آیی کہ یہاں کا نظام صحت کیسے کام کرتا ہے
- ☐ آپکو شرم محسوس ہوتی تھی۔
- ☐ کوئی انتظامی وجہ مثلاً انشورنس وغیرہ
- ☐ کوئی اور، برائے مہربانی وضاحت کیجیے ___________________
- ☐ لاگو نہیں ہوتا N/A

12. *** اس حمل کے دوران اپکے سب سے اہم معلومات ذرائع کون اور کیا تھے؟**
(ماں کے جوابات کے مطابق مندرجہ ذیل پر نشان لگائیں)

- ☐ گزشتہ حمل
- ☐ فیملی یا دوست
- ☐ مزھبی رہنما
- ☐ گاینیکالوجسٹ، ماہر زچگی ، OBGYN
- ☐ فیملی ڈاکٹر، جنرل پریکٹیشنر، GP
- ☐ دائیّ
- ☐ نرس پریکٹیشنر/ نرس
- ☐ زچگی اور بچے کی پیداش کے متعلق کلاس
- ☐ کتابیں
- ☐ ٹی وی
- ☐ انٹرنیٹ
- ☐ کوئی اور (برائے مہربانی وضاحت کیجیے ___________________

13. *** M اس حمل کے دوران کیا آپ کو آپکی زبان میں معلومات فراھم کی گیں؟**

- ☐ جی ________________ *(برائے مہربانی وضاحت کیجےّ)*
- ☐ نہیں

.14 * اس حمل میں، لیبر اور بچے کی پیدأیش سے پہلے ان موضوعات کے بارے میں آپ کے پاس مناسب معلومات تھیں؟

	جی	نہیں	معلوم نہیں
حمل کے دوران جسمانی تبدیلی	☐	☐	☐
حمل کے دوران جذبات میں تبدیلی	☐	☐	☐
لیبر کے شروع ہونے کی نشانیاں	☐	☐	☐
دوائیاں	☐	☐	☐
لیبر اور پیدایش کے عمل سے متعلق معلومات	☐	☐	☐
ادویات کے بغیر درد کا علاج	☐	☐	☐
ضروری میڈیکل ٹیسٹ	☐	☐	☐
حمل کے دوران غذائی ضروریات	☐	☐	☐
بچے کی پیدائش کے بعد آپکی اپنی صحتیابی	☐	☐	☐
آپکے مزاج میں تبدیلی	☐	☐	☐
بچے کو سمبھالنے کا طریقہ	☐	☐	☐
بچے کی صحت یا نشونما میں مسٔلے کو پہچاننا	☐	☐	☐
ماں کا دودھ پلانا	☐	☐	☐
ڈبے کا دودھ پلانا	☐	☐	☐
بچے کی صحت کے متعلق سوالات کے لیے کس سے رابطہ کیا جاۓ	☐	☐	☐
خاندانی منصوبہ بندی	☐	☐	☐
HIV اور دیگر جنسی بیماریاں	☐	☐	☐

.15 * کیا آپکی ماہر صحت نے اپ سے پوچھا کہ آپ اپنے بچے کو کیسے دودھ پلانا چاہتی ہیں ؟

☐ جی
☐ نہیں
☐ معلوم نہیں / یاد نہیں
☐ N/A کوئی ماہر صحت نہیں تھا

.16 * کیا آپکی/ آپکے ماہرصحت نےآپ پوچھا کہ حمل کے دوران آپکی ترجیحات کیا ہیں، اور اگر آپ کوئ خاص طریقہ کار اختیار کرنا چاہتی ہوں؟

☐ جی
☐ نہیں
☐ N/A کوئی ماہر صحت نہیں تھا

اگلے چند سوال آپکی حالیہ بچے کی پیدائش کے عمل اور پیدا ہونے والے بچے کے بارے میں ہیں. اس حصے میں 16 سوال ہیں.

.17 بچے کی پیدائّش کے وقت آپکا حمل کتنے ہفتے کا تھا؟ ___________ (ہفتے)

☐ معلوم نہیں

.18 آپ نے کتنے بچوں کو جنم دیا؟ ______________ (مثلا ایک ، جڑواں وغیرہ)

.19 * پیدآش کے وقت بچے/بچوں کا وزن کتنا تھا؟

کلو(kg)_____________ گرام (grams) / __________- پاونڈ (lbs) _________ اونس (oz)

(اگر ایک سے زیادہ بچوں کو جنم دیا)
کلو(kg)_____________ گرام (grams) / __________- پاونڈ (lbs) _________ اونس (oz)

~~**.20** *بچے کی پیدایش کے وقت آپ کہاں تھیں؟~~
~~*(اونچا پڑھ کر سنائیں اور کسی ایک پر نشان لگائیں)*~~

☐

.21 * آپکی لیبر کے دوران زیادہ تر کس ماہر صحت نے آپ کی سب سی زیادہ دیکھ بھال کی؟
(اونچا پڑھ کر سنائیں اور کسی ایک پر نشان لگائیں)

- ☐ ماہر زچگی، گائینیکولوجسٹ، OBGYN
- ☐ فیملی ڈاکٹر، جنرل پریکٹیشنر، GP
- ☐ دائی
- ☐ نرس/ نرس پریکٹیشنر
- ☐ کوئی اور، برائے مہربانی وضاحت کیجیے _______________
- ☐ کسی نے نہیں
- ☐ کسی نے نہیں، لیبر نہیں ہوئ (بڑے آپریشن کے ذریے بچہ ہوا)
- ☐ معلوم نہیں

.22 *بچے کی پیدائش کے وقت کس ماہر صحت نے آپ کی سب سے زیادہ دیکھ بھال کی؟
(اونچا پڑھ کر سنائیں اور کسی ایک پر نشان لگائیں)

- ☐ ماہر زچگی، گائینیکولوجسٹ، OBGYN
- ☐ فیملی ڈاکٹر، جنرل پریکٹیشنر، GP
- ☐ دائی
- ☐ نرس/ نرس پریکٹیشنر
- ☐ کوئی اور، برائے مہربانی وضاحت کیجیے _______________
- ☐ کسی نے نہیں
- ☐ کسی نے نہیں، لیبر نہیں ہوئ (بڑے آپریشن کے ذریے بچہ ہوا)
- ☐ معلوم نہیں

23. * **لیبرکے دوران اور جنم دیتے وقت آپ مندرجہ ذیل میں سے کسی عمل سے گزریں؟**

	ہاں	نہیں
لیبر کی درد کا شروع	☐	☐
لیبر کی درد کا بڑھانا(ہونے والی درد کی شدّت کو بڑھانے اور ان کے درمیان وقفہ کو کم کرنے کے لیے)	☐	☐
فورسپ (چمٹی) کا استعمال	☐	☐
بچے کو نکالنے کے لیے ویکیوم پمپ کا استعمال	☐	☐
بڑا آپریشن	☐	☐
جائے مخصوصہ پر کٹ (episiotomy)	☐	☐
درد زہ کی درد کی کمی اپیڈیورل (epidural) ٹیک	☐	☐
بڑے آپریشن کیلٔے کمر کے نیچے ٹیکا	☐	☐
مکمّل بیہوشی کا ٹیکا (general anaesthesia)	☐	☐
کوئی اور، برائے مہربانی وضاحت کیجیے ________________	☐	☐

24. * **لیبر کے دوران اور جنم دیتے وقت آپکو کوئی طبّی مسائل پیش آے ؟ (مثلا نالی کا پھٹنا (perineal tear)، رحم کا پھٹنا (uterine rupture)، انفیکشن (infection) , ، بچے کی پیدائش کے بعد اندرونی خون کا جاری ہونا (postpartum haemorrhage) یا بچے کو کوئی پیچیدگی)**

☐ جی ہاں، *(برائے مہربانی وضاحت کیجیے)* ________________________________
☐ نہیں

____

اگر بچا نارمل ہوا ہے تو اگلا سوال چھوڑ کر سوال نمبر 26 پر جائیں.

25. ***اگر آپکا بچا بڑے آپریشن سے ہوا ہے تو اس کی کیا وجہ تھی؟**
(ماں کے جوابات کے مطابق مندرجہ ذیل پر نشان لگائیں)

☐ پہلے سے طہ تھا کیونکہ طبّی مسلئے کی وجہ سے ڈاکٹر نے اسکا مشورہ دیا
☐ پہلے سے طہ تھا لیکن آپکو وجہ معلوم نہیں
☐ پہلے سے طہ تھا کیونکہ آپ چاہتی تھیں . لیکن کوئی طبّی مسلہ نہیں تھا
☐ پہلے سے طہ تو نہیں تھا لیکن لیبر بہت لمبی ہو گئی تھی
☐ پہلے سے طہ نہیں تھا لیکن بچے کی جا ن کو خطرہ تھا
☐ پہلے سے طہ نہیں تھا لیکن آپ کی جان کو خطرہ تھا
☐ پہلے سے طہ تو نہیں تھا اور آپ کو معلوم نہیں کہ یہ کیوں کیا
☐ ان کے علاوہ (برائے مہربانی وضاحت کیجئے): ______________________
☐ لاگو نہیں ہوتا (N/A) نارمل ڈیلیوری ہوی

26. **لیبر کے دوران آپکو گھومنے پھرنے اورآرامدہ پوزیشن میں آنے کی اجازت تھی ؟**
(اونچا پڑھ کر سنائیں اور کسی ایک پر نشان لگائیں)

☐ جی، ہمیشہ
☐ جی، کبھی کبھار
☐ جی، بہت کم
☐ نہیں - طبّی پیچیدگی کی وجہ سے
☐ نہیں - پتہ نہیں کیوں
☐ نہیں ، لیبر پلان نہیں کی تھی (بڑا آپریشن ہوا)

27. **لیبر کے دوران ماہر صحت نے آپ سے پوچھا کہ آپ اپنے درد کا کیا انتظام کرنا چاہتی ہیں؟**

- ☐ جی ہاں
- ☐ نہیں
- ☐ پتہ نہیں/ یاد نہیں
- ☐ نہیں ، لیبر پلان نہیں کی تھی (بڑا آپریشن ہوا)

28. **لیبر کے دوران ماہر صحت نے آپکو درد برداشت کرنے میں جو مدد کی ، کیا آپ اس سے مطمعین ہیں؟**

- ☐ جی ہاں
- ☐ نہیں
- ☐ کبھی کبھار
- ☐ نہیں ، لیبر پلان نہیں کی تھی (بڑا آپریشن ہوا)

29. **لیبر کے دوران آپکو آپکی مرضی کے لوگ یا خاندان کے افراد ساتھ رکھنے کی اجازت تھی؟**

- ☐ جی ہاں
- ☐ نہیں
- ☐ کبھی کبھار
- ☐ نہیں ، لیبر پلان نہیں کی تھی (بڑا آپریشن ہوا)

30. *** ڈیلیوری کے دوران آپکا کوئی ساتھی تھا/ تھی؟**
(اونچا پڑھ کرسنائیں اور کسی ایک پر نشان لگائیں)

- ☐ جی ہاں ہمیشہ
- ☐ جی ہاں کبھی کبھار
- ☐ جی ہاں بہت کم
- ☐ نہیں
- ☐ پتہ نہیں ، یاد نہیں

31. ***اگر ہاں تو وہ کون تھے/ تھی ؟**
(اگر ایک سے زیادہ ہیں تو سب کا بتائیں)

__________________________ (آپ سے رشتہ ؟)
__________________________ (آپ سے رشتہ ؟)
__________________________ (آپ سے رشتہ ؟)
☐ N/A لاگو نہیں ہوتا

32. *** ماہر صحت نے آپ سے پوچھا کہ آپ لیبر کے دوران اپنا کوئی خاص طریقہ کار یا روایت استعمال کرنا چاہتی ہیں؟**

- ☐ جی ہاں
- ☐ نہیں
- ☐ نہیں، کیونکہ میں نے ان کے پوچھنے سے پہلے ہی بتا دیا تھا

اگلے چند سوال بچے کی پیدائش کے بعد سے متعلق ہیں. اس حصّے میں 14 سوال ہیں

33. * کیا آپ کے بچے کو آپ سے علیحدہ کسی جگہ پر خاص دیکھ بھال کی ضرورت پڑی؟
(اونچا پڑھ کر سنائیں اور کسی ایک پر نشان لگائیں)

- ☐ جی ہاں، نو مولود کی انتہای نگہداشت کا یونٹ (neonatal intensive care unit)
- ☐ جی ہاں، بچوں کی نگہداشت کا مخصوص نگہداشت یونٹ (special care baby unit)
- ☐ جی ہاں، نرسری میں
- ☐ جی ہاں، مگر یاد نہیں کہاں پر
- ☐ نہیں
- ☐ معلوم نہیں/ یاد نہیں

34. ~~بچے کی پیدائش کے بعد آپ کتنی دیر ہسپتال میں رہیں؟~~

35. ~~آپ کے خیال میں یہ عرصہ بہت کم تھا/ بہت طویل تھا/ مناسب تھا ؟~~

☐

36. ماہر صحت نےہسپتال میں آپکے قیام کے دوران کھانے میں آپکی ترجیحات کے بارے میں پوچھا؟ (مثلا ٹھنڈا یا گرم ، آپکی دینی ترجیحات کے مطابق تیار کردہ ، حلال، سبزی، یا کسی اور قسم کا کھانا

- ☐ جی
- ☐ نہیں
- ☐ معلوم نہیں / یاد نہیں
- ☐ لاگو نہیں ہوتا N/A (پیدائش گھر پر ہوئ)

37. * ماہر صحت نے آپ سے آپکی اپنی نگہداشت کے بارے میں ترجیحات کے متعلق پوچھا یا یہ پوچھا کہ آپ پیدائش کے بعد کوئی خاص طریقہ کار یا رسم ادا کرنا چاہتی ہیں؟

- ☐ جی
- ☐ نہیں
- ☐ معلوم نہیں/ یاد نہیں

38. بچے کی پیدائش کے پہلے گھنٹے کے اندر آپ کو اپکا بچہ اس طرح پکڑنے کیلئے دیا گیا کہ آپکی جلد سے اسکی جلد چھو سکے؟

- ☐ جی
- ☐ نہیں. اگر نہیں تو کیوں: ____

.39 * آپکی ماہر صحت نے کب آپکو ماں کا دودہ پلانا شروع کرنے میں مدد کرنا شروع کی ؟
(ماں کے جواب کے مطابق کسی ایک پر نشان لگائیں)

- ☐ پیدائش کے پہلے گھنٹے کے اندر
- ☐ فوری طور پر نہیں لیکن جائے پیدائش پر میرے قیام کے دوران
- ☐ بعد کی تاریخ میں، ماہر صحت کے ساتھ ملاقات کے دوران
- ☐ انہوں نے کوئی مدد نہیں کی
- ☐ معلوم نہیں / یاد نہیں
- ☐ میں اپنے بچے کو ماں کا دودھ نہیں پلانا چاہتی تھی

.40 *آپکی ماہر صحت نے آپکی کمیونٹی میں ماں کا دودھ پلانے میں مددگار وسائل کے بارے میں بتایا؟

- ☐ جی ہاں
- ☐ نہیں ، لیکن مجھے یہ معلومات چاہئیے نہیں تھی (اگر نہیں تو سوال نمبر 42 پر جائیے)
- ☐ نہیں ، لیکن مجھے یہ معلومات چچہئیے تھیں (اگر نہیں تو سوال نمبر 42 پر جائیے)
- ☐ معلوم نہیں / یاد نہیں

~~**.41** *اگر ہاں تو کیا آپ نے ماں کا دودھ پلانے میں مدد کے وہ زرائع استعمال کیے؟~~

☐

~~**.42** *بچے کی پیدائش کے بعد کیا اس حمل سے متعلقہ کسی وجہ سے آپ نے یا آپکے بچے نے کسی ماہر صحت کو دکھایا گیا ہے ؟~~
~~(معمول کے معائنے کو شامل کر کے)~~

☐

~~**.43** *اگر ہاں تو کیوں؟~~ ____________________

~~**.44** * اگر دکھایا تو کس کو؟~~

~~**.45** * کیا پیدائش کے بعد آپ کسی ماہر صحت کو دکھانا چاہتی تھیں لیکن دکھا نہیں سکیں؟~~

.46 ~~* اگر آپ کسی ماہر صحت کو نہیں دکھا سکیں تو کیوں؟~~
~~(ماں کے جوابات کے مطابق مندرجہ ذیل پر نشان لگائیں)~~

اگلے چند سوال آپکی حالیہ زچگی کے دوران مجموعی دیکھ بھال کے متعلق ہیں. اس حصّے میں 20 سوال ہیں.

.47 اپنی حالیہ زچگی پر نظر ثانی کرتے ہوے بتائے کہ کیا کوئی ایسا مشورہ، معلومات یا سہولت ہے، جو آپ چاہتیں کہ آپکو ملتی ؟

____

____

____

.48 *مجموعی طور پر جب آپ ماہرین صحت سے ملتیں تو آپ کو ان کی طرف سے خیر مقدم کا احساس ہوتا؟

(a حمل کے دوران

- ☐ ہمیشہ
- ☐ کبھی کبھی
- ☐ بہت کم
- ☐ کبھی نہیں

(b لیبر اور پیدائش کے دوران

- ☐ ہمیشہ
- ☐ کبھی کبھی
- ☐ بہت کم
- ☐ کبھی نہیں

c) بچے کی پیدائش کے بعد
- ☐ ہمیشہ
- ☐ کبھی کبھی
- ☐ بہت کم
- ☐ کبھی نہیں

49. *مجموعی طور پر ماہرین صحت آپ سے عزت سے پیش آتے تھے؟

a) حمل کے دوران
- ☐ ہمیشہ
- ☐ کبھی کبھی
- ☐ بہت کم
- ☐ کبھی نہیں

b) لیبر اور پیدائش کے دوران
- ☐ ہمیشہ
- ☐ کبھی کبھی
- ☐ بہت کم
- ☐ کبھی نہیں

c) بچے کی پیدائش کے بعد
- ☐ ہمیشہ
- ☐ کبھی کبھی
- ☐ بہت کم
- ☐ کبھی نہیں

50. *مجموعی طور پر ماہرین صحت مددگار تھے؟

a) حمل کے دوران
- ☐ ہمیشہ
- ☐ کبھی کبھی
- ☐ بہت کم
- ☐ کبھی نہیں

b) لیبر اور پیدائش کے دوران
- ☐ ہمیشہ
- ☐ کبھی کبھی
- ☐ بہت کم
- ☐ کبھی نہیں

c) بچے کی پیدائش کے بعد
- ☐ ہمیشہ
- ☐ کبھی کبھی
- ☐ بہت کم
- ☐ کبھی نہیں

.51 * مجموعی طور پر میں اپنی صحت کی دیکھ بھال سے خوش تھی؟

a) حمل کے دوران
- ☐ ہمیشہ
- ☐ کبھی کبھی
- ☐ بہت کم
- ☐ کبھی نہیں

b) لیبر اور پیدائش کے دوران
- ☐ ہمیشہ
- ☐ کبھی کبھی
- ☐ بہت کم
- ☐ کبھی نہیں

c) بچے کی پیدائش کے بعد
- ☐ ہمیشہ
- ☐ کبھی کبھی
- ☐ بہت کم
- ☐ کبھی نہیں

.52 *آپکے حمل، لیبر اور بچے کی پیدائش کے دوران ماہر صحت نے آپ سے کچھ ایسا کرنے کو کہا جو آپ نہیں کرنا چاہتی تھیں؟
- ☐ جی ہاں
- ☐ نہیں
- ☐ معلوم نہیں / یاد نہیں

.53 اگر ہاں تو وہ کیا تھا؟

____

- ☐ N/A لاگو نہیں ہوتا

.54 ماہر صحت نے آپ سے پوچھا کہ آپ اپنی دیکھ بھال کے لیے مرد یا عورت کو ترجیح دیں گی؟

a) حمل کے دوران
- ☐ ہمیشہ
- ☐ کبھی کبھی
- ☐ بہت کم
- ☐ کبھی نہیں
- ☐ تبصرہ ____________________

b) لیبر اور ڈلیوری کے دوران
- ☐ ہمیشہ
- ☐ کبھی کبھی
- ☐ بہت کم
- ☐ کبھی نہیں
- ☐ تبصرہ ____________________

c) بچے کی پیدائش کے پہلے دن
- ☐ ہمیشہ
- ☐ کبھی کبھی
- ☐ بہت کم
- ☐ کبھی نہیں
- ☐ تبصرہ ____________________

.55 *ماہر صحت کی طرف سے دی گئ معلومات آپکو سمجھ آتی تھی؟

a) حمل کے دوران

- ☐ ہمیشہ
- ☐ کبھی کبھی
- ☐ بہت کم
- ☐ کبھی نہیں
- ☐ تبصرہ ____________________

b) لیبر اور ڈلیوری کے دوران

- ☐ ہمیشہ
- ☐ کبھی کبھی
- ☐ بہت کم
- ☐ کبھی نہیں
- ☐ تبصرہ ____________________

c) بچے کی پیدائش کے پہلے دن

- ☐ ہمیشہ
- ☐ کبھی کبھی
- ☐ بہت کم
- ☐ کبھی نہیں
- ☐ تبصرہ ____________________

.56 M *یہ معلومات آپکو کسی اور زبان میں زیادہ بہتر سمجھ آ سکتی تھیں ؟

- ☐ جی ، کونسی زبان؟ ____________________
- ☐ نہیں *(سوال نمبر 61 پر جایں)*
- ☐ پتہ نہیں / یاد نہیں

.57 M *ماہر صحت نے آپکو ترجمانی کی سہولت میسّر کی؟

a) حمل کے دوران

- ☐ جی
- ☐ نہیں
- ☐ لاگو نہیں ہوتا

b) لیبر اور بچے کی پیدائش کے دوران

- ☐ جی
- ☐ نہیں
- ☐ لاگو نہیں ہوتا

c) پیدائش کے بعد پہلے دن

- ☐ جی
- ☐ نہیں
- ☐ لاگو نہیں ہوتا

.58 M * آپکے پاس کتنی دفع کوئی ایسا شخص ہوتا تھا جو آپکی زبان بول سکتا تھا اور آپ کی ترجمانی کر سکتا تھا ؟

a) حمل کے دوران

- ☐ ہمیشہ
- ☐ کبھی کبھی
- ☐ بہت کم
- ☐ کبھی نہیں
- ☐ لاگو نہیں ہوتا

b) لیبر اور بچے کی پیدائش کے دوران

- ☐ ہمیشہ
- ☐ کبھی کبھی
- ☐ بہت کم
- ☐ کبھی نہیں
- ☐ لاگو نہیں ہوتا

c) بچے کی پیدائش کے بعد پہلے دن

- ☐ ہمیشہ
- ☐ کبھی کبھی
- ☐ بہت کم
- ☐ کبھی نہیں
- ☐ لاگو نہیں ہوتا

.59 M *اگر آپکے پاس آپکی ترجمانی کے لیے کوئی موجود تھا تو وہ کون تھا؟

(اونچا پڑھ کر سنائیں اور تمام متلعقہ پر نشان لگائیں)

a) حمل کے دوران

- ☐ شوہر
- ☐ خاندان کا کوئی اور فرد یا دوست
- ☐ صحت کی دیکھ بھال کا عملہ
- ☐ آپکا بچہ
- ☐ پیشہ ور ترجمان
- ☐ کوئی اور مریض یا اسکے خاندان کا فرد یا دوست
- ☐ کوئی اور، براے مہربانی وضاحت کیجئے: ____________________
- ☐ لاگو نہیں ہوتا

b) لیبر اور بچے کی پیدائش کے دوران

- ☐ شوہر
- ☐ خاندان کا کوئی اور فرد یا دوست
- ☐ صحت کی دیکھ بھال کا عملہ
- ☐ آپکا بچہ
- ☐ پیشہ ور ترجمان
- ☐ کوئی اور مریض یا مریض کے خاندان کا فرد یا دوست
- ☐ کوئی اور، براے مہربانی وضاحت کیجئے: ____________________
- ☐ N/A لاگو نہیں ہوتا

c) بچے کی پیدائش کے بعد پہلے دن

- ☐ شوہر
- ☐ خاندان کا کوئی اور فرد یا دوست
- ☐ صحت کی دیکھ بھال کا عملہ
- ☐ آپکا بچہ
- ☐ پیشہ ور ترجمان
- ☐ کوئی اور مریض یا مریض کے خاندان کا فرد یا دوست
- ☐ کوئی اور، براے مہربانی وضاحت کیجئے: ____________________
- ☐ لاگو نہیں ہوتا

.60 آپ ان کی ترجمانی سے مطمین تھیں؟ M

- ☐ جی ہاں
- ☐ نہیں
- ☐ معلوم نہیں/ یاد نہیں
- ☐ N/A

.61 * لیبر اور بچے کی پیدائش کے دوران یا بچے کی پیدائش کی بعد کیا کوئی خاص رسومات/ طریقہ کار تھا جو آپ ادا کرنا چاہتی ہوں مگر ماہر صحت نے اس کی اجازت نہ دی ہو / یا مہییا نہ کر سکا ہو

- ☐ جی ہاں
- ☐ نہیں *(سوال نمبر 64 پر جائیں)*
- ☐ معلوم نہیں / یاد نہیں

.62 اگر ہاں تو وہ کیا تھیں ؟

*i)*___________

*ii)*__________

*iii)*__________

☐ **N/A** لاگو نہیں ہوتا

.63 ماہر صحت نے آپکو یہ طریقہ کار یا رسومات ادا کرنے کی اجازت نہ دینے کی کیا وجہ بتائی ؟

i)___________

ii)__________

iii)__________

☐ **N/A** لاگو نہیں ہوتا

.64 * کیا کوئی ایسی چیز ہے جو آپکے خیال میں ماہر صحت مختلف یا بہتر طور پر کر سکتی/سکتا ہوں؟

a) حمل کے دوران

- ☐ جی ہاں *(سوال نمبر 65 a مکمّل کیجئے)*
- ☐ نہیں
- ☐ معلوم نہیں / یاد نہیں

b) لیبرکے دوران اور بچے کی پیدائش کے وقت ؟

- ☐ جی ہاں *(سوال نمبر 65 b مکمّل کیجئے)*
- ☐ نہیں
- ☐ معلوم نہیں / یاد نہیں

c) بچے کی پیدائش کے بعد ؟

- ☐ جی ہاں *(سوال نمبر 65 c مکمّل کیجئے)*
- ☐ نہیں
- ☐ معلوم نہیں / یاد نہیں

.65 **اگر ہاں تو برائے مہربانی وضاحت کریں کہ کون، کیا مختلف یا بہتر کر سکتا تھا؟**

a) حمل کے دوران

____

b) لیبر اور بچے کی پیدائش کے دوران ؟

____

c) بچے کی پیدائش کے بعد ؟

____

.66 ***اپنے بچے کی پیدائش کے تجربے کے بارے میں کوئی ایسی بات جس سے :**

a) آپ خصوصی طور پر خوش ہیں

____

b) آپ خصوصی طور پر نہ خوش ہیں

____

اپنے حالیہ حمل کو مد نظر رکھتے ہوے مجھے یہ بتائیے کہ آگے آنے والے ۱۱ بیانات کس حد تک درست ہیں ؟

.67 *** ماہرین صحت مجھ سے پوچھتی /پوچھتے تھے کہ میں کوئی سوال پوچھنا چاہتی ہوں؟**

- ☐ ہمیشہ
- ☐ کبھی کبھی
- ☐ بہت کم
- ☐ کبھی نہیں

.68 **ماہر صحت جلدی میں ہوتا / ہوتی تھیں**

- ☐ ہمیشہ
- ☐ کبھی کبھی
- ☐ بہت کم
- ☐ کبھی نہیں

.69 * **مجھے لگتا تھا کہ میری تشویش کو ماہر صحت سنجیدگی سے لیتے تھے**

a) دوران حمل

- ☐ ہمیشہ
- ☐ کبھی کبھی
- ☐ بہت کم
- ☐ کبھی نہیں
- ☐ لاگو نہیں ہوتا (حمل کی نگہداشت نہیں ملی) N/A

b) لیبرکے دوران اور بچے کی پیدائش کے وقت

- ☐ ہمیشہ
- ☐ کبھی کبھی
- ☐ بہت کم
- ☐ کبھی نہیں
- ☐ لاگو نہیں ہوتا (کوئی ماہر صحت موجود نہیں تھی)

c) بچے کی پیدائش کے بعد

- ☐ ہمیشہ
- ☐ کبھی کبھی
- ☐ بہت کم
- ☐ کبھی نہیں
- ☐ N/A لاگو نہیں ہوتا (کوئی ماہر صحت موجود نہیں تھی)

.70 **مجھے نگہداشت حاصل کرنے کے لیے بہت طویل انتظار کرنا پڑا**

a) دوران دوران حمل

- ☐ ہمیشہ
- ☐ کبھی کبھی
- ☐ بہت کم
- ☐ کبھی نہیں
- ☐ لاگو نہیں ہوتا (حمل کی نگہداشت نہیں ملی)

b) لیبرکے دوران اور بچے کی پیدائش کے وقت

- ☐ ہمیشہ
- ☐ کبھی کبھی
- ☐ بہت کم
- ☐ کبھی نہیں
- ☐ لاگو نہیں ہوتا (کوئی ماہر صحت موجود نہیں تھی)

c) بچے کی پیدائش کے بعد

- ☐ ہمیشہ
- ☐ کبھی کبھی
- ☐ بہت کم
- ☐ کبھی نہیں
- ☐ لاگو نہیں ہوتا (کوئی ماہر صحت موجود نہیں تھی)

.71 *ماہر صحت نے مجھے ہونے والے واقعیات کے بارے میں اگاہ رکھا

a) **دوران دوران حمل**
- ☐ ہمیشہ
- ☐ کبھی کبھی
- ☐ بہت کم
- ☐ کبھی نہیں
- ☐ لاگو نہیں ہوتا (حمل کی نگہداشت نہیں ملی)

b) **لیبرکے دوران اور بچے کی پیدائش کے وقت**
- ☐ ہمیشہ
- ☐ کبھی کبھی
- ☐ بہت کم
- ☐ کبھی نہیں
- ☐ لاگو نہیں ہوتا (کوئی ماہر صحت موجود نہیں تھی)

c) **بچے کی پیدائش کے بعد**
- ☐ ہمیشہ
- ☐ کبھی کبھی
- ☐ بہت کم
- ☐ کبھی نہیں
- ☐ لاگو نہیں ہوتا (کوئی ماہر صحت موجود نہیں تھی)

.72 جو باتیں مجھے سمجھ نہیں آتیں ، میں بلا جھجک ان کے بارے میں پوچھ سکتی تھی

a) **دوران دوران حمل**
- ☐ ہمیشہ
- ☐ کبھی کبھی
- ☐ بہت کم
- ☐ کبھی نہیں
- ☐ لاگو نہیں ہوتا (حمل کی نگہداشت نہیں ملی)

b) **لیبرکے دوران اور بچے کی پیدائش کے وقت**
- ☐ ہمیشہ
- ☐ کبھی کبھی
- ☐ بہت کم
- ☐ کبھی نہیں
- ☐ لاگو نہیں ہوتا (کوئی ماہر صحت موجود نہیں تھی)

c) **بچے کی پیدائش کے بعد**
- ☐ ہمیشہ
- ☐ کبھی کبھی
- ☐ بہت کم
- ☐ کبھی نہیں
- ☐ لاگو نہیں ہوتا (کوئی ماہر صحت موجود نہیں تھی)

.73 * ماہر صحت میری خواہشات کو مدّنظر رکھے بغیر فیصلے کرتا / کرتی تھی

a) دوران دوران حمل

- ☐ ہمیشہ
- ☐ کبھی کبھی
- ☐ بہت کم
- ☐ کبھی نہیں
- ☐ لاگو نہیں ہوتا (حمل کی نگہداشت نہیں ملی)

b) لیبرکے دوران اور بچے کی پیدائش کے وقت

- ☐ ہمیشہ
- ☐ کبھی کبھی
- ☐ بہت کم
- ☐ کبھی نہیں
- ☐ لاگو نہیں ہوتا (کوئی ماہر صحت موجود نہیں تھی)

c) بچے کی پیدائش کے بعد

- ☐ ہمیشہ
- ☐ کبھی کبھی
- ☐ بہت کم
- ☐ کبھی نہیں
- ☐ لاگو نہیں ہوتا (کوئی ماہر صحت موجود نہیں تھی)

.74 * ماہر صحت بہت حوصلہ افزا اور یقین دہانی کرانے والی تھی / تھا

a) دوران دوران حمل

- ☐ ہمیشہ
- ☐ کبھی کبھی
- ☐ بہت کم
- ☐ کبھی نہیں
- ☐ لاگو نہیں ہوتا (حمل کی نگہداشت نہیں ملی)

b) لیبرکے دوران اور بچے کی پیدائش کے وقت

- ☐ ہمیشہ
- ☐ کبھی کبھی
- ☐ بہت کم
- ☐ کبھی نہیں
- ☐ لاگو نہیں ہوتا (کوئی ماہر صحت موجود نہیں تھی)

c) بچے کی پیدائش کے بعد

- ☐ ہمیشہ
- ☐ کبھی کبھی
- ☐ بہت کم
- ☐ کبھی نہیں
- ☐ لاگو نہیں ہوتا (کوئی ماہر صحت موجود نہیں تھی)

.75 * کیا ماہر صحت نے آپکو چیزیں سمجھانے میں مناسب وقت صرف کیا؟

a) دوران دوران حمل
- ☐ ہمیشہ
- ☐ کبھی کبھی
- ☐ بہت کم
- ☐ کبھی نہیں
- ☐ لاگو نہیں ہوتا (حمل کی نگہداشت نہیں ملی)

b) لیبرکے دوران اور بچے کی پیدائش کے وقت
- ☐ ہمیشہ
- ☐ کبھی کبھی
- ☐ بہت کم
- ☐ کبھی نہیں
- ☐ لاگو نہیں ہوتا (کوئی ماہر صحت موجود نہیں تھی)

c) بچے کی پیدائش کے بعد
- ☐ ہمیشہ
- ☐ کبھی کبھی
- ☐ بہت کم
- ☐ کبھی نہیں
- ☐ لاگو نہیں ہوتا (کوئی ماہر صحت موجود نہیں تھی)

.76 * کیا آپ سمجھتی ہیں کہ مجموعی طور پر ماہر صحت نے آپکے ساتھ دوسروں سے مختلف برتاؤ کیا گیا ؟ (مثلا آپکی زبان، لہجے، تمدّن ، نسل ، رنگ، مذھب، امیگریشن سٹیٹس، یا صحت کی بیمہ پالیسی سٹیٹس کی وجہ سے)

- ☐ ہمیشہ *(براے مہربانی سوال نمبر 77 میں وضاحت کیجئے)*
- ☐ کبھی کبھی *(براے مہربانی سوال نمبر 77 میں وضاحت کیجئے)*
- ☐ بہت کم *(براے مہربانی سوال نمبر 77 میں وضاحت کیجئے)*
- ☐ کبھی نہیں *(سوال نمبر 78 پر جائیے)*

.77 * اگر ہاں تو آپ کے خیال میں کس وجہ / وجوہات کی بنا پر؟
(ماں کے جوابات کے مطابق نشان لگائیں)

- ☐ زبان یا لہجہ
- ☐ تہذیب
- ☐ نسل / ذات
- ☐ رنگ
- ☐ مذھب
- ☐ امیگریشن سٹیٹس
- ☐ صحت کے بیمہ کا سٹیٹس
- ☐ کوئی اور وجہ، وضاحت کیجئے ___________________________
- ☐ N/A لاگو نہیں ہوتا

اگلے چند سوال آپکے گزشتہ حمل اور زچگیوں کے متعلق ہیں . اس حصّے میں 8 سوال ہیں

78. آپ کتنی بار ماں بننے کے عمل سے گزر چکی ہیں ؟ (اس حمل کو شامل کرتے ہوے) ________________

~~**79.** * آپکے کتنے حمل ضایٔع ہوے؟~~ ____________________

~~یہ سوال پوچھنے سے پہلے تسلی کر لیں کہ کوئی اور موجود نہیں ہے~~

~~**80.** * کتنے حمل ضایٔع کر دئیے گئے؟~~ ____________________

~~**81.** * کتنے حمل میں بچہ مردہ پیدا ہوا ؟~~ ______________________

82. * آپکے کتنےزندہ بچے حمل کے 37 ہفتے پورے ہونے سے پہلے پیدا ہوے؟ _________________________

☐ لاگو نہیں ہوتا N/A

83. * آپکے کتنے زندہ بچے حمل کے 37 ہفتے پورے ہونے کے بعد پیدا ہوے؟ ________________

☐ لاگو نہیں ہوتا N/A

84. * کیا گزشت حمل کے دوران آپ کسی طبّی پیچیدگی سے دوچار ہوئیں؟

☐ جی ہاں
☐ نہیں *(سوال نمبر 86 پر جائیے)*
☐ لاگو نہیں ہوتا N/A *(سوال نمبر 86 پر جائیے)*

85. * اگر آپ اپنے گزشتہ حمل میں کسی طبّی پیچیدگی سے دوچار ہوئیں تو وہ کیا تھے
(ماں کے جوابات کے مطابق نشان لگائیں)

- ☐ بڑا آپریشن (c section)
- ☐ خون کی کمی
- ☐ بلند فشار خون
- ☐ دوران حمل بلند فشار خون (preeclampsia)
- ☐ قبل از وقت لیبر
- ☐ رگوں میں خون کی رکاوٹ (Deep vein thrombosis)
- ☐ دوران حمل ذیابطیس (Gestational diabetes)
- ☐ رحم میں نالی کا نیچے پیوست ہونا (Placenta praevia)
- ☐ بچے کی خوراک کی نالی کا اکھڑ جانا (Placental abruption)
- ☐ پیشاب کی نالی میں انفیکشن
- ☐ کمر میں شدید درد
- ☐ قبل از وقت رحم سے جھلی کا پھٹ جانا
- ☐ ڈپریشن
- ☐ کوئی اور (برائے مہربانی وضاحت کیجئے) ______________________
- ☐ معلوم نہیں
- ☐ لاگو نہیں ہوتا N/A

آخری چند سوال آپکے اور آپکے خاندان سے متعلق ہیں – اس حصّے میں 27 سوال ہیں.

86. * آپکی ازدواجی حیثیت کیا ہے؟

- ☐ شادی شدہ
- ☐ باہمی رضامندی کا ساتھ (شادی کے بغیر جوڑا)
- ☐ بیوہ
- ☐ علیحدہ
- ☐ طلاق یافتہ
- ☐ غیر شادی شدہ

87. *آپ کس کے ساتھ رہتی ہیں ؟

	جی ہاں	نہیں
شوہر / مرد ساتھی	☐	☐
خاتون ساتھی	☐	☐
آپکی ماں / باپ	☐	☐
آپکے بہن / بھای	☐	☐
ساتھی کے ماں / باپ	☐	☐
ساتھی کے بہن/ بھائی	☐	☐
وست	☐	☐
بچے (نومولود کے علاوہ)	☐	☐
کوئی اور وضاحت کریں _______________	☐	☐
کوئی نہیں- میں اپنے بچے کے ساتھ اکیلی رہتی ہوں	☐	☐
کوئی نہی - میں اکیلی رہتی ہوں	☐	☐

.88 *آپکے کتنے بچے آپ کے ساتھ رہتے ہیں (نومولود سمیت)؟ ___________

.89 M*آپکے کتنے بچے اس ملک میں پیدا ہوے (نومولود سمیت)؟ ____________

.90 آپکی تاریخ پیدائش کیا ہے؟ _____________

.91 M*آپکی والدہ کس ملک میں پیدا ہوئیں؟

____

.92 M* آپکے والد کس ملک میں پیدا ہوے ؟

____

اگلے سوال آپکی امیگریشن کی تفصیلات کے متعلق ہیں. ہم یہ سوال اس لیے پوچھ رہے ہیں تاکہ بین الاقوامی مہاجرین کے تجربات کے بارے میں جان سکیں. آپ کی دی ہوئ تمام معلومات خفیہ رکھی جاۓ گی اور کوئی بات بھی امیگریشن افسر کو نہیں بتائی جاۓ گی. ان سوالات کے جواب سے آپکی امیگریشن کی درخواست پر کوئی اثر نہیں پڑے گا اگر آپ نے پناہ گزین حیثیت، مستقل شہریت یا شہری کی درخواست دی ہوئ ہے.

.93 M* آپکا موجودہ امیگریشن (حیثیت) سٹیٹس کیا ہے؟
(ماں کے جواب کے مطابق کسی ایک پر نشان لگائیں)

- ☐ مہاجر (مستقل شہری)
- ☐ پناہ گزین
- ☐ متلاشی پناہ
- ☐ عارضی کام کرنے والا / ساتھ رہ کر دیکھ بھال کرنے والا
- ☐ عارضی باشندہ
- ☐ طالب علم
- ☐ ملاقاتی
- ☐ کوئی حیثیت نہیں
- ☐ Undocumented دستاویزات کے بغیر
- ☐ شہری
- ☐ کوئی اور- وضاحت کیجئے _________________

.94 M آپ کتنے عرصے سے اس حیثیت میں ہیں ؟ _____ (دن) _____ (ہفتے) ______ (ماہ) _______ (سال)

.95 M آپ کے آنے کے بعد آپکے امیگریشن سٹیٹس میں کوئی تبدیلی آیی؟

- ☐ جی ہاں
- ☐ نہیں *(سوال نمبر 97 پر جائیے)*

96. M*اگر ہاں تو اس سے پہلے آپکا امیگریشن سٹیٹس کیا تھا؟

- ☐ مہاجر (مستقل شہری)
- ☐ پناہ گزین
- ☐ متلاشی پناہ
- ☐ عارضی کام کرنے والا / ساتھ رہ کر دیکھ بھال کرنے والا
- ☐ عارضی باشندہ
- ☐ طالب علم
- ☐ ملاقاتی
- ☐ کوئی حیثیت نہیں
- ☐ دستاویزات کے بغیر Undocumented
- ☐ کوئی اور ، وضاحت کیجئے : _____________
- ☐ لاگو نہیں ہوتا (سٹیٹس تبدیل نہیں ہوا)

---

97. M کیا آپکا کبھی پناہ گزین درجہ رہا ہے؟

- ☐ جی ہاں
- ☐ نہیں
- ☐ معلوم نہیں / یاد نہیں

---

98. M آپ نے کبھی امیگریشن کے نظر بندی مرکز میں وقت گزارا ہے؟

- ☐ جی ہاں
- ☐ نہیں *(سوال نمبر ۱۰۱ پر جائیے)*

---

99. M اگر ہاں تو کتنا عرصہ _______ (دن) ______ ہفتے _________(ماہ) __________ سال

- ☐ لاگو نہیں ہوتا N/A

---

100. M * اگر ہاں تو کیا اس دوران آپ حاملہ تھیں؟

- ☐ جی ہاں
- ☐ نہیں
- ☐ لاگو نہیں ہوتا N/A

---

101. * آپکو میسّر سہولیات صحت کے اخراجات کون اٹھاتا ہے؟

	جی ہاں	*نہیں*	*معلوم نہیں*
عوامی فنڈ سے چلنے والا بیمہ صحت (RAMQ)	☐	☐	☐
نجی بیمہ صحت	☐	☐	☐
پناہ گزین کیلئے خاص سرکاری بیمہ صحت	☐	☐	☐
آپ خود اخراجات اٹھاتی ہیں	☐	☐	☐

.102 * آپ نے کتنی تعلیم حاصل کی ہے؟

- ☐ پرائمری اسکول
- ☐ سیکنڈری ڈپلوما
- ☐ پوسٹ سیکنڈری ڈپلوما (کالج، یونیورسٹی تجارتی اسکول)
- ☐ گریجویٹ (ماسٹرز، پی ایچ ڈی)
- ☐ کوئی نہیں

.103 M آپکو قانونی طور پر اس ملک میں کام کرنے کی اجازت ہے؟

- ☐ جی ہاں
- ☐ نہیں
- ☐ معلوم نہیں

.104 * بچے کی پیدائش سے پہلے آپکی آخری تنخواہ دار نوکری کیا تھی؟ (مثلا ڈاکٹر، استاد، اندراج معلومات کی کلرک،ماتحت نرس، ہوٹل یا گھر کی صفائی کرنے والی، کھیت میں کام کرنے والی، کپڑا رنگنے والی مشین کو چلانے والی، کال سینٹر میں کم کرنے والی)

- ☐ برائے مہربانی وضاحت کیجئے ____________________
- ☐ لاگو نہیں ہوتا (کام نہیں کرتی تھیں)

~~**.105** * بچے کی پیدائش کے بعد آپ کام پر واپس گئیں؟~~

.106 ~~* اگر ہاں تو آپکی موجودہ نوکری کیا ہے؟~~

.107 * اپنے پورے گھرانے کی آمدنی کو مدنظر رکھتے ہوئے (ٹیکس سے پہلے) آپ آمدنی کے کس طبقے سے تعلق رکھتی ہیں (مقامی کرنسی کے مطابق درجہ بتائیں ؟ مندرجہ ذیل اونچا پڑھ کر سنائیں)

- ☐ > ____$11, 000____
- ☐ __$11,000____ سے __$20,999____
- ☐ __$21,000____ سے __$40,999____
- ☐ __$41,000____ سے __$60,999____
- ☐ __$61,000____ سے __$80,999____
- ☐ ≤ ___$81,000____

.108 * یہ آمدنی کتنے لوگوں کی کفالت کرتی ہے (نومولود کو شامل کرتے ہوئے)؟ _______________

.109 * اپنے گھر میں آپ عموماً کونسی زبان / زبانیں بولتی ہیں؟

______________ ______________

.110 M*اس ملک میں بولی جانے والی زبانیں آپ کو کتنی اچھی طرح آتی ہیں؟

English:

	روانی سے	ٹھیک طرح سے	مشکل سے	بالکل نہیں
**بولنا**	☐	☐	☐	☐
**پڑھنا**	☐	☐	☐	☐
**لکھنا**	☐	☐	☐	☐
**سمجھنا**	☐	☐	☐	☐

.111 M اس ملک میں بولی جانے والی زبانیں آپ کو کتنی اچھی طرح آتی ہیں؟

French:

	روانی سے	ٹھیک طرح سے	مشکل سے	بالکل نہیں
**بولنا**	☐	☐	☐	☐
**پڑھنا**	☐	☐	☐	☐
**لکھنا**	☐	☐	☐	☐
**سمجھنا**	☐	☐	☐	☐

.112 ہمارا انٹرویو یہاں ختم ہوتا ہے. اس موضوع پر آپ کچھ اور کہنا چاہیں گی؟ اس کے علاوہ کوئی اور تبصرہ کرنا چاہیں گی؟
