# Supplementary material for: Migrant-friendly maternity care in Montreal, Canada: A cross-sectional study on migrant women’s care perspectives
Source: PLoS One. 2025 Aug 21;20(8):e0330830. doi: 10.1371/journal.pone.0330830 (PMC12370051; doi:10.1371/journal.pone.0330830)
Supplement: S9 Appendix — (PDF) [file pone.0330830.s009.pdf]

Sự chăm sóc cho phụ nữ di cư trong quá trình mang thai, chuyển dạ, sinh đẻ, và sau khi sinh con tại Montreal.
“Migrant Friendly Maternity Care/ Soins de maternité qui répondent aux besoins des migrantes” Questionnaire

Hướng dẫn cho người phỏng vấn

1. Khi bạn thực hiện cuộc phỏng vấn này, xin hãy đọc từng câu hỏi từ từ và rõ ràng. Thực hiện theo các hướng dẫn như sau để lựa chọn câu trả lời:
   - Đối với các câu hỏi mà nói "Xin hãy đọc tất cả và đánh dấu những gì là hợp lý với bạn", xin hãy đọc từng câu trả lời và cho phép các bà mẹ trả lời *Có* hoặc *Không* cho mỗi lựa chọn;
   - Đối với các câu mà nói "Xin hãy cho người phụ nữ trả lời câu hỏi này trước, rồi sau đó đánh dấu tất cả những gì là hợp lý với bạn", xin hãy hỏi các bà mẹ câu hỏi trước và không cho cô ấy biết bất kỳ sự lựa chọn nào, rồi sau đó tự quyết định câu trả lời phù hợp nhất. Nếu cô ấy không thể nghĩ ra câu trả lời, xin hãy đọc 2-3 câu lựa chọn để giúp cô ta nghĩ ra câu trả lời.

2. Xin hãy thực hiện theo hướng dẫn về các câu hỏi có lựa chọn bỏ qua.
   Ví dụ, câu hỏi số 10 hỏi về các dịch vụ mà các bà mẹ đã muốn sử dụng nhưng rồi lại không sử dụng dịch vụ này. Xin hãy chỉ đọc câu hỏi số 11 nếu người phụ nữ cho biết trong câu hỏi số 10 rằng có: một dịch vụ cô ấy muốn sử dụng nhưng rồi không dùng.

3. Đối với các câu hỏi đề cập tới những khoảng thời gian (như câu hỏi số 2, 6), hãy nhắc các bà mẹ rằng cô ấy có thể đưa ra câu trả lời tốt nhất.

4. Nếu mà bạn không có chắc chắn câu trả lời nào là phù hợp, hãy điền câu trả lời là "Khác" và việt một lời giải thích.

5. Đối với những từ ngữ mà các bà mẹ có thể không hiểu, xin hãy dùng các định nghĩa và giải thích sau đây:
   - **Kế hoạch gia đình:** kế hoạch gia đình; kế hoạch về sinh đẻ và sử dụng biện pháp tránh thai
   - **Bệnh lây truyền qua đường tình dục:** bị bệnh hoặc nhiễm trùng bởi vì có quan hệ tình dục với một người nào đó bị nhiễm hoặc có căn bệnh này
   - **Gây tê:** thuốc gây tê dùng để không có cảm giác trong một chỗ nhất định hoạc toàn thân
   - **Đơn vị chăm sóc em bé đặc biệt:** khoa chăm sóc em bé, sử dụng khi em bé phải tách riêng biệt với mẹ, ở một căn phòng đặc biệt, với sự chú ý của các nhân viên y tế tại vì vấn đề sức khoẻ không dược tốt
   - **Sẩy thai:** thai bị mất trước khi được 20 tuần
   - **Phá thai:** loại bỏ thai nhi trước khi thai nhi được phát chuyển
   - **Tình trạng di trú** (câu hỏi 93)**:** bắt đầu từ khi đến quốc gia này chứ KHÔNG phải khi nhận được giấy tờ
   - **Trung tâm giam giữ người nhập cư:** Có thể hỏi rằng: Bạn đã bao giờ bị giam giữ bởi cơ quan di trú hay không?
   - **Thu nhập:** thu nhập của mọi người sống trong nhà (ví dụ như, anh, chị, em, chồng, hay mẹ) trước khi khai thuế
     Thu nhập này hỗ trợ được bao nhiêu người: bao gồm chị, em, em bé của chị hay em, v.v.
   - **Chuyển dạ:** khi cơn co thắt (đau) bắt đầu cho đến khi em bé được sinh ra

GHI CHÚ: (1) Các câu hỏi được đánh dấu * (n = 86) được xác định (trong một quá trình đồng thuận của Delphi với các chuyên gia nghiên cứu sức khỏe chu sinh quốc tế) như là một tập câu hỏi tối thiểu sử dụng theo tiêu chuẩn so sánh của quốc tế, (2) Câu hỏi được đánh dấu với M là những câu hỏi chỉ thích hợp cho phụ nữ di cư hoặc được xác định là người di cư để phân tích về sức khỏe của trẻ sơ sinh (xem Gagnon AJ, Zimbeck M, Zeitlin J. Migration and Perinatal Health Surveillance: An International Delphi Survey. *European Journal of Obstetrics & Gynecology and Reproductive Biology.* 2010;149(1), 37-43).

Cuộc phỏng vấn bắt đầu: tóm tắt dự án cho các bà mẹ

Tôi đang làm việc với một nhóm nghiên cứu quan tâm đến kinh nghiệm thai sản (việc sinh đẻ) của những phụ nữ di cư trong một quốc gia mới. Tôi sẽ hỏi bạn những câu hỏi về kinh nghiệm của bạn trong thời gian mang thai, quá trình chuyển dạ, sinh đẻ, sau khi em bé được sinh ra, và toàn bộ kinh nghiệm thai sản của bạn. Và trong đó cũng sẽ có thêm một loạt các câu hỏi về lịch sử sản khoa và lịch sử di cư của bạn. Trong thời gian phỏng vấn, bạn có thể yêu cầu tôi đọc lại những câu hỏi bạn không nghe rõ hoặc giải thích những câu hỏi bạn không hiểu, bất cứ khi nào bạn cần. Tôi cũng muốn nhắc lại rằng tất cả các thông tin bạn cung cấp sẽ được giữ riêng tư. Bạn có thể rút sự tham gia của bạn từ chương trình nghiên cứu này bất cứ lúc nào bạn muốn, và bạn có thể chọn không trả lời bất kỳ câu hỏi nào mà bạn không cảm thấy thoải mái để trả lời.

Bất cứ khi nào bạn có câu hỏi hoạc thắc mắc gì, xin vui lòng cho tôi biết. Bạn có thắc mắc gì trước khi chúng ta bắt đầu không?
Bây giờ chúng ta sẽ bắt đâu!

MFMCQ Vietnamese Version

		ID của người phụ nữ:	
THỜI GIAN BẮT ĐẦU:		Tên của người phỏng vấn:	
THỜI GIAN KẾT THÚC:		Ngày phỏng vấn:	

1. ***M Nơi bạn được sinh ra là**

____

2. *** M Bạn đã sống trong quốc gia này được bao nhiêu lâu rồi?**
(Nếu mà bạn đã từng di chuyển tới một quốc gia khác rồi sau đó quay trở lại xin hãy tính TỔNG CỘNG là bao nhiêu lâu)

________ (ngày) ________ (tuần) ________(tháng) ________ (năm)

Những câu hỏi đầu tiên có liên quan tới việc MANG THAI GẦN ĐÂY NHẤT của bạn tại quốc gia này. Trong phần này gồm có 14 câu hỏi.

3. **M Có phải bạn đã mang thai (em bé gần đây nhất) trước khi tới quốc gia này hay không?**

☐ Phải, tôi đã mang thai em bé được _________ tuần
☐ Không, tôi có thai sau khi tới đây
☐ Tôi không biết

4. *** Trong thời kỳ mang thai này, bạn có gặp các chuyên gia y tế (như bác sĩ, y tá, hoặc nữ hộ sinh- người đỡ đẻ) hay không?**
☐ Có __________________________ (tại quốc gia nào)
☐ Không (*Xin hãy tiếp tục với câu hỏi số 8*)

5. ***Ai đã chăm sóc cho bạn?**
(Xin hãy cho người phụ nữ trả lời câu hỏi này trước, rồi sau đó đánh dấu tất cả những gì là hợp lý với bạn, nếu cần thiết, hãy cho cô ấy biết vài câu lựa chọn để giúp cô ta trả lời)

- ☐ Bác sĩ gia đình, bác sĩ đa khoa
- ☐ Bác sĩ sản khoa, bác sĩ phụ khoa
- ☐ Nữ hộ sinh (người đỡ đẻ)
- ☐ Y tá
- ☐ Người khác *(Xin ghi rõ thêm chi tiết)*: _________
- ☐ Câu hỏi này không áp dụng đối với tôi

---

6. *** Bạn đã có thai được bao nhiêu tuần khi mà bạn gặp chuyên gia y tế lần đầu tiên trong khi mang thai này?**
_________ (tuần); Tại quốc gia này: _________ (tuần)
(Không tính lần đi thử nghiệm mang thai)

- ☐ Câu hỏi này không áp dụng đối với tôi **(**Tôi không nhận được sự chăm sóc trong thời gian mang thai này)

---

7. *** Bạn đã gặp bác sĩ, y tá, hoặc nữ hộ sinh (người đỡ đẻ) bao nhiêu lần trong thời gian mang thai này ? _________**

- ☐ Câu hỏi này không áp dụng đối với tôi **(**Tôi không nhận được sự chăm sóc trong thời gian mang thai này)

---

8. *** Bạn đã có bất cứ vấn đề gì về sức khoẻ trong thời gian mang thai này hay không?**

- ☐ Có ***(Xin hãy đánh dấu tất cả những gì áp dụng với bạn)***
  - ☐ Thiếu máu [*Anaemia/ Anémie*]
  - ☐ Huyết áp cao [*High blood pressure/ Hypertension arterielle*]
  - ☐ Tiền sản giật (tăng huyết áp thai kỳ) [*Preeclampsia (gestational hypertension) / Pre-eclampsie (hypertension artérielle gravidique*]
  - ☐ Sinh non (em bé sinh thiếu tháng) [*Preterm labour/ Travail prématuré*]
  - ☐ Huyết khối tĩnh mạch sâu (nghẽn mạch sâu) [*Deep vein thrombosis/ Thrombose veineuse profonde*]
  - ☐ Tiểu đường thai kỳ [*Gestational diabetes/ Diabète de grossesse*]
  - ☐ Nhau thai tiền đạo [*Placenta praevia/ Placenta praevia*]
  - ☐ Nhau thai bong non (bánh nhau bong ra khi thai chưa được sổ ra ngoài) [*Placental abruption/ Décollement placentaire*]
  - ☐ Nhiễm trùng tiết niệu [*Urinary tract infection/ Infection urinaire*]
  - ☐ Đau lưng [*Severe back pain/ Douleurs sévères au dos*]
  - ☐ Ối vỡ non [*Preterm rupture of membranes/ Rupture premature des membranes*]
  - ☐ Trầm cảm (buồn chán) [*Depression/ Dépression*]
  - ☐ Bệnh khác *(Xin ghi rõ thêm chi tiết)*: _________________________ (bao gồm vấn đề với thai nhi)
- ☐ Không, tôi không có vấn đề về sức khoẻ trong thời gian mang thai này

9. **Trong khi mang thai, bạn đã sử dụng các dịch vụ nào sau đây?**

	Có	*Không*
Các lớp học về mang thai và sinh em bé	☐	☐
Gặp chuyên gia y tế	☐	☐
Ngân hàng thực phẩm (Food Bank)	☐	☐
Hỗ trợ tìm kiếm nhà	☐	☐
Y học cổ truyền/ nghi thức	☐	☐
Dịch vụ dành cho gia đình (ví dụ như Child Care, tư vấn gia đình, khoá học làm cha mẹ)	☐	☐
Kiểm tra sức khoẻ (y tế) trong khi mang thai (ví dụ như, kiểm tra sức khoẻ toàn thân, xét nghiệm máu, kiểm tra cổ tử cung/Pap test)	☐	☐
Kiêm tra tật và bệnh bẩm sinh trong thai nhi (ví dụ như bệnh Down)	☐	☐
Siêu âm	☐	☐
Dịch vụ hỗ trợ (ví dụ như, dịch vụ sức khoẻ tâm thần)	☐	☐
Dịch vụ khác *(Xin ghi rõ thêm chi tiết)*: ____________________	☐	☐

10.* Trong khi mang thai, bạn đã có muốn sử dụng các dịch vụ nào sau đây nhưng rồi không dùng đến hay không?
(Xin hãy đọc tất cả và đánh dấu những gì là hợp lý với bạn)

☐ Các lớp học về mang thai và sinh em bé
☐ Gặp chuyên gia y tế
☐ Ngân hàng thực phẩm (Food Bank)
☐ Hỗ trợ tìm kiếm nhà
☐ Y học cổ truyền/ nghi thức
☐ Dịch vụ dành cho gia đình (ví dụ như, Child Care, tư vấn gia đình, khoá học làm cha mẹ)
☐ Kiểm tra sức khoẻ (y tế) trong khi mang thai (ví dụ như, kiểm tra sức khoẻ toàn thân, xét nghiệm máu, kiểm tra cổ tử cung/Pap test)
☐ Kiêm tra tật và bệnh bẩm sinh trong thai nhi (ví dụ như, bệnh Down)
☐ Siêu âm
☐ Dịch vụ hỗ trợ (ví dụ như, dịch vụ sức khoẻ tâm thần)
☐ Dịch vụ khác *(Xin ghi rõ thêm chi tiết)*: ____________________
☐ Không (*Xin hãy tiếp tục với câu hỏi số 12*)

11. * Nếu bạn không nhận được sự chăm sóc mà bạn muống trong thời gian mang thai gần đây, nhưng yếu tố hoặc sự ngăn chặn nào đã ảnh hưởng tới vấn đề này?
(Xin hãy đánh dấu tất cả những gì là hợp lý vời bạn)

- ☐ Không có dịch vụ nào cung cấp trong khu vực gần nhà tôi
- ☐ Không có đủ chỗ dành cho tôi trong các dịch vụ tôi muốn sử dụng
- ☐ Tôi đã không biết về các dịch vụ này
- ☐ Tôi đã không biết mình được phép để sử dụng các dịch vụ này
- ☐ Tôi đã không được phép để sử dụng các dịch vụ này
- ☐ Tôi đã không biết nơi nào mà các dịch vụ này được dung cấp
- ☐ Tôi sợ rằng hồ sơ di trú của tôi sẽ bị ảnh hưởng
- ☐ Cuộc hẹn bị huỷ bỏ bởi các nhà cung cấp
- ☐ Không tìm được nơi giữ trẻ em (Child Care)
- ☐ Vấn đề về ngôn ngữ
- ☐ Tôi không có các vận chuyển nào để đi tới những nơi này
- ☐ Vì lý do tài chính
- ☐ Tôi đang đi làm
- ☐ Tôi không có thời gian
- ☐ Tôi cần phải ở nhà
- ☐ Tôi lo sợ về sự xét nghiệm y tế và khám bệnh
- ☐ Thay vì các dịch vụ này, tôi đã nhận được sự tư vấn và giúp đỡ từ gia đình và bạn bè
- ☐ Tôi không nghĩ hệ thống chăm sóc sức khoẻ tại đây cung cấp các dịch vụ này
- ☐ Tôi không biết nhiều về hệ thống chăm sóc sức khoẻ và không biết cách sử dụng hệ thống này
- ☐ Tôi cảm thấy xấu hổ
- ☐ Lý do hành chính (ví dụ như, không có bảo hiểm)
- ☐ Lý do khác *(Xin ghi rõ thêm chi tiết)*: ________________
- ☐ Câu hỏi này không áp dụng đối với tôi

12.* Trong thời gian mang thai này, ai hay cái gì đã là nguồn thông tin quan trọng nhất của bạn về việc mang thai, chuyển dạ, và sinh con?
(Xin hãy cho người phụ nữ trả lời câu hỏi này trước, rồi sau đó đánh dấu tất cả những gì là hợp lý với bạn)

- ☐ Thời gian mang thai trước đây
- ☐ Già đình hoặc bạn bè
- ☐ Người lãnh đạo tâm linh (Cha nhà thờ, Thầy nhà chùa)
- ☐ Bác sĩ sản khoa, bác sĩ phụ khoa
- ☐ Bác sĩ gia đình, bác sĩ đa khoa
- ☐ Nữ hộ sinh (người đỡ đẻ)
- ☐ Y tá
- ☐ Các lớp học về mang thai/ sinh em bé
- ☐ Sách
- ☐ Truyền hình (tivi)
- ☐ Internet
- ☐ Nguồn thông tin khác *(Xin ghi rõ thêm chi tiết)*: _________________

13.* M Trong khi mang thai này, bạn có được cung cấp thông tin bằng ngôn ngữ của bạn hay không?

- ☐ Có _______________ *(Xin ghi rõ thêm chi tiết)*
- ☐ Không

14.* Trong khi mang thai này, trước khi chuyển dạ và sau khi sinh em bé, bạn đã có đủ thông tin về các chủ đề sau đây hay không?

	Có	*Không*	*Tôi không biết*
Các thay đổi về cơ thể trong khi mang thai	☐	☐	☐
Các thay đổi về cảm xúc khi mang thai	☐	☐	☐
Dấu hiệu sự bắt đầu chuyển dạ	☐	☐	☐
Thuốc	☐	☐	☐
Những gì xảy ra trong quá trình chuyển dạ và sinh em bé	☐	☐	☐
Cách điều trị những cơn đau mà không dùng đến thuốc	☐	☐	☐
Những kiểm tra sức khoẻ cần thiết	☐	☐	☐
Dinh dưỡng khi mang thai	☐	☐	☐
Sức khoẻ và phục hồi sau khi sinh em bé	☐	☐	☐
Những thay đổi tâm trạng mà có thể xẩy ra	☐	☐	☐
Cách chăm sóc em bé	☐	☐	☐
Làm thế nào để phát hiện các vấn đề về sức khoẻ và sự phát truyển của em bé	☐	☐	☐
Cho em bé bú sữa mẹ	☐	☐	☐
Cho em bé bú sữa ngoài (như sữa bột)	☐	☐	☐
Người liên lạc nếu có thắc mắc về sức khoẻ của bạn hay của em bé	☐	☐	☐
Kế hoạch gia đình, sinh đẻ, và những biện pháp ngăn ngừa có thai	☐	☐	☐
HIV và các bệnh lây truyền qua đường tình dục khác	☐	☐	☐

15.* Chuyên gia y tế của bạn có hỏi kế hoạch của bạn về cách cho em bé ăn (bú) hay không?

☐ Có
☐ Không
☐ Tôi không biết/ Tôi không nhớ
☐ Câu hỏi này không áp dụng đối với tôi (Tôi không có chuyên gia y tế)

16.* Chuyên gia y tế của bạn có hỏi về những yêu cầu của bạn về sự chăm sóc hoặc những phong tục và tập quán gì đặc biệt của bạn trong khi mang thai hay không?

☐ Có
☐ Không
☐ Câu hỏi này không áp dụng đối với tôi (Tôi không có chuyên gia y tế)

Những câu hỏi tiếp theo có liên quan tới quá trình CHUYỂN DẠ của bạn và SINH ĐẺ của em bé mới sinh ra GẦN ĐÂY NHẤT. Trong phần này gồm có 16 câu hỏi.

17.* Bạn đã mang thai được bao nhiêu tuần trong khi sinh em bé? ________ (tuần)

☐ Tôi không biết

18.* **Bạn đã sinh tất cả là bao nhiêu em bé? _______________ (ví dụ như, sinh một em bé, sinh đôi, vv.)**

19.* **Em bé được bao nhiêu cân khi sinh ra?**

________(kg) ________(grams)/ _______(lbs) ________(oz)

_________(kg) ________(grams)/ _______(lbs) ________(oz) ***(Nếu mà đẻ nhiều hơn một em bé)***

~~**20.* Bạn đang ở đâu khi sinh em bé?**~~
~~***(Xin hãy đọc tất cả vào đánh dấu một câu trả lời)***~~

☐

21.* **Chuyên gia y tế nào đã chăm sóc cho bạn nhiều nhất trong quá trình CHUYỂN DẠ của bạn?**
(Xin hãy đọc tất cả và đánh dấu những gì là hợp lý với bạn)

☐ Bác sĩ sản khoa, bác sĩ phụ khoa
☐ Bác sĩ gia đình, bác sĩ đa khoa
☐ Nữ hộ sinh (người đỡ đẻ)
☐ Y tá
☐ Người khác *(Xin ghi rõ thêm chi tiết)*: _________________________
☐ Không có ai
☐ Không có vì đã có kế hoạch mổ để lấy thai nhi [*Caesarean section/ Césarienne*]
☐ Tôi không biết

22.* **Chuyên gia y tế nào đã chăm sóc cho bạn nhiều nhất trong thời gian EM BÉ ĐƯỢC SINH RA?**
(Xin hãy cho người phụ nữ trả lời trước, rồi sau đó lựa chọn một câu trả lời thích hợp nhất)

☐ Bác sĩ sản khoa, bác sĩ phụ khoa
☐ Bác sĩ gia dình, bác sĩ đa khoa
☐ Nữ hộ sinh (người đỡ đẻ)
☐ Y tá
☐ Người khác *(Xin ghi rõ thêm chi tiết)*: _________________________
☐ Không có ai
☐ Tôi không biết

23.* Bạn đã dùng những phương pháp nào sau đây trong quá trình chuyển dạ và sinh con?

	Có	*Không*
Khởi phát chuyển dạ (làm cho cơn co thắt bắt đầu)	☐	☐
Điều chỉnh sự chuyển dạ (làm cho cơn co thắt mạnh hơn và thường xuyên hơn)	☐	☐
Sử dụng kẹp (hoặc đồ kim loại để lấy em bé ra)	☐	☐
Sử dụng máy hút để lấy em bé ra	☐	☐
Mổ để lấy thai nhi [*Caesarean section/ Césarienne*]	☐	☐
Cắt tầng sinh môn (cắt gần cửa mở của âm đạo)	☐	☐
Gây tê ngoài màng cứng để giảm đau trong khi chuyển dạ [*Epidural/ Anesthésie péridurale pour la douleur durant le travail*]	☐	☐
Gây tê tuỷ sống để mổ đẻ	☐	☐
Gây mê	☐	☐
Phương pháp khác *(Xin ghi rõ thêm chi tiết)*: _______________	☐	☐

24.* Bạn có bất kỳ biến chứng gì khi chuyển dạ và sinh em bé hay không?
(Chẳng hạn như rách tầng sinh môn, vỡ tử cung, nhiễm trùng, băng huyết sau sinh, hoặc vấn đề với em bé)

☐ Có *(Xin ghi rõ thêm chi tiết)*:____

☐ Không

Nếu mà bạn đã sinh ngã âm đạo (để tự nhiên), hãy tiếp tục với câu hỏi số 26

25. * Nếu em bé đã được sinh rà bằng cách mổ, nguyên nhân của việc này là gì?
(Xin hãy cho người phụ nữ trả lời trước, rồi sau đó lựa chọn một câu trả lời thích hợp nhất)

☐ Đã có kế hoạch này trước vì bác sĩ đã đề nghị do vấn đề sức khoẻ của tôi hoặc em bé

☐ Đã có kế hoạch này trước nhưng tôi không chắc chắn lý do tại sao

☐ Đã có kế hoạch này trước vì tôi muốn chứ không phải vì lý do sức khoẻ

☐ Không có kế hoạch này nhưng vì quá trình chuyển dạ của tôi đã quá lâu

☐ Không có kế hoạch này nhưng vì lúc đó em bé đang gặp nguy hiểm

☐ Không có kế hoạch này nhưng vì lúc đó tôi đang gặp nguy hiểm

☐ Không có kế hoạch này nhưng tôi không biết lý do tại sao phải mổ

☐ Vì lý do khác *(Xin ghi rõ thêm chi tiết)*:______________________________

☐ Tôi đã sinh em bé ngã âm đạo (đẻ tự nhiên)

26. Trong quá trình chuyển dạ và đau đẻ, bạn có được di chuyển xung quanh hoặc chọn tư thế thoải mái cho mình hay không?
(Xin hãy đọc tất cả vào đánh dấu một câu trả lời)

☐ Có, luôn luôn

☐ Có, đôi khi

☐ Có, hiếm khi

☐ Không

☐ Không, tôi không biết lý do tại sao

☐ Không, đã có kế hoạch mổ để lấy thai nhi [*Caesarean section/ Césarienne*]

27. **Trong quá trình chuyển dạ và đau đẻ, chuyên gia y tế có hỏi bạn về cách mà bạn muốn để làm giảm đau hay không?**

- ☐ Có
- ☐ Không
- ☐ Tôi không biết/ không nhớ
- ☐ Không, đã có kế hoạch mổ để lấy thai nhi [*Caesarean section/ Césarienne*]

---

28. **Trong quá trình chuyển dạ và đau đẻ, bạn có hài lòng về cách mà chuyên gia y tế đã dùng để giúp làm giảm đau cho bạn hay không?**

- ☐ Có
- ☐ Không
- ☐ Đôi khi
- ☐ Không, đã có kế hoạch mổ để lấy thai nhi [*Caesarean section/ Césarienne*]

---

29. **Trong quá trình chuyển dạ và đau đẻ, bạn có được phép lựa chọn người thân (người trong gia đình), hoặc người hỗ trợ mà bạn muốn, để ở trong phòng đẻ ở cùng bạn hay không?**

- ☐ Có
- ☐ Không
- ☐ Đôi khi
- ☐ Không, đã có kế hoạch mổ để lấy thai nhi [*Caesarean section/ Césarienne*]

---

30. *** Bạn có người thân hay ai đó ở bên cạnh mình <u>trong khi sinh</u> em bé hay không?**
(Xin hãy đọc tất cả và lựa chọn một câu trả lời thích hợp nhất)

- ☐ Có, luôn luôn
- ☐ Có, đôi khi
- ☐ Có, hiếm khi
- ☐ Không
- ☐ Tôi không biết/ không nhớ

---

31. *** Nếu CÓ, người đó là ai?**
(Nếu có nhiều hơn một người, xin hãy đề cập đến tất cả)

___________________________ (quan hệ của họ đối với bạn)
___________________________ (quan hệ của họ đối với bạn)
___________________________ (quan hệ của họ đối với bạn)

- ☐ Câu hỏi này không áp dụng đối với tôi (Không có ai)

---

32. *** Chuyên gia y tế có hỏi về những yêu cầu của bạn về sự chăm sóc hoặc những phong tục và tập quán gì đặc biệt của bạn trong khi chuyển dạ và sinh đẻ hay không?**

- ☐ Có
- ☐ Không
- ☐ Không, vì tôi đã không hỏi họ trước khi họ hỏi tôi

Những câu hỏi tiếp theo có liên quan tới khoảng thời gian SAU KHI SINH của bạn kể từ khi em bé được sinh ra. Trong phần này gồm có 14 câu hỏi.

33.* Em bé của bạn khi sinh ra đã phải nhận sự chăm sóc đặc biệt ở một nơi riêng biệt với bạn, có hay không?
(Xin hãy đọc tất cả và lựa chọn một câu trả lời thích hợp nhất)

- ☐ Có, trong một đơn vị chăm sóc tích cực dành cho trẻ sơ sinh
- ☐ Có, trong một đơn vị chăm sóc em bé đặc biệt
- ☐ Có, trong khu sinh dưỡng (vườn ươm) [*Nursery/ La pouponnière*]
- ☐ Có (nhưng tôi không biết/ không nhớ là ở tại đâu)
- ☐ Không
- ☐ Tôi không biết/ không nhớ

~~**34. Bạn đã ở lại trong bệnh viện hoặc phòng khám bao nhiêu lâu sau khi em bé được sinh ra?**~~

~~**35.Bạn nghĩ rằng khoảng thời gian này đã là quá ngắn/ quá dài/ vừa phải?**~~

☐

36.Chuyên gia y tế có hỏi bạn về sở thích và chế độ ăn uống đặc biệt của bạn (ví dụ như nhiệt độ của đồ ăn, đồ ăn theo tôn giáo, đồ ăn chay, hoặc những loại đồ ăn khác) trong thời gian bạn ở trong bệnh viện/trung tâm sinh đẻ hay không?

- ☐ Có
- ☐ Không
- ☐ Tôi không biết/ không nhớ
- ☐ Câu hỏi này không áp dụng đối với tôi (Tôi đã sinh em bé tại nhà)

37.* Chuyên gia y tế có hỏi bạn về bất kỳ các yêu cầu về sự chăm sóc của bạn hoặc những phong tục và tập quán đặc biệt sau khi sinh đẻ của bạn hay không?

- ☐ Có
- ☐ Không
- ☐ Tôi không biết/ không nhớ

38.Trong giờ đầu tiên sau khi em bé đã được sinh ra, bạn có được bế (ôm) em bé da với da chạm nhau hay không? (làn da của em bé trực tiếp chạm vào làn da của bạn)

- ☐ Có
- ☐ Không. Nếu bạn trả lời KHÔNG, lý do tại sao: ____

39.* Trong khoảng thời gian nào, người chuyên gia y tế đã giúp bạn, hoặc đề cập tới sự giúp bạn, bắt đầu cho con bú sữa mẹ?
(Xin hãy cho người phụ nữ trả lời câu hỏi này trước, rồi sau đó đánh dấu tất cả những gì là hợp lý với bạn)

□ Trong giờ đầu tiên sau khi em bé được sinh ra
□ Không phải ngay lập tức, nhưng trong khi tôi vẫn còn ở nơi mà tôi đã sinh em bé (ví dụ như bệnh viện, trung tâm sinh đẻ, hoạc tại nhà)
□ Sau này, trong một cuộc hẹn cùng chuyên gia y tế
□ Họ đã không giúp tôi hoặc đề cập tới việc cho con bú với tôi
□ Tôi không nhớ/ không biết
□ Tôi không muốn cho con tôi bú sữa mẹ

40.* Chuyên gia y tế có cung cấp thông tin cho bạn về các nguồn lực hỗ chợ trong cộng đồng của bạn mà bạn có thể tới để biết thêm chi tiết về việc cho con bú hay không?

□ Có
□ Không nhưng tôi không cần thông tin này (Nếu không, hãy tiếp tục với câu hỏi số 42)
□ Không nhưng tôi muốn biết thông tin này (Nếu không, hãy tiếp tục với câu hỏi số 42)
□ Tôi không biết/ không nhớ

~~41.* Nếu bạn trả lời CÓ, bạn có dùng các thông tin này không?~~

□

~~42.* Bạn hay em bé của bạn có gặp chuyên gia chăm y tế từ khi em bé được sinh ra vì bất kỳ lý do liên quan tới việc mang thai lần này (bao gồm sự chăm sóc thông thường) hay không?~~

~~43.* Nếu bạn trả lời CÓ, lý do tại sao?~~ ________________________

□

~~44.* Nếu bạn trả lời CÓ, chuyên gia mà bạn đã gặp là ai?~~
~~(Xin hãy cho người phụ nữ trả lời câu hỏi này trước, rồi sau đó đánh dấu tất cả những gì là hợp lý với bạn)~~

~~45.* Kể từ khi sinh em bé, bạn đã có muốn gặp các chuyên gia y tế cho bạn hoặc em bé nhưng rồi không gặp được hay không?~~

46.* ~~**Nếu bạn không gặp được các chuyên gia y tế, lý do tại sao?**~~
~~***(Xin hãy cho người phụ nữ trả lời câu hỏi này trước rồi sau đó đánh dấu tất cả những gì là hợp lý với bạn)***~~

Những câu hỏi tiếp theo có liên quan tới TOÀN BỘ KINH NGHIỆM TRONG KHI MANG THAI, QUÁ TRÌNH CHUYỂN DẠ, SINH ĐẺ, VÀ SAU KHI SINH CON của bạn trong thời gian mang thai gần đây nhất. Trong phần này gồm có 20 câu hỏi.

47. Bây giờ suy nghĩ lại, có những lời khuyên, hỗ trợ, hoặc thông tin nào mà bạn ước mình đã biết đến và nhận được hay không?

____
____
____

48.* Nói chung, khi bạn gặp gỡ các chuyên gia y tế, bạn có cảm thấy được sự hoan nghênh từ họ hay không?

a) Trong khi mang thai

- ☐ Lúc nào cũng có (luôn luôn)
- ☐ Đôi khi
- ☐ Hiếm khi
- ☐ Không bao giờ

b) Trong khi chuyển dạ và đang sinh em bé

- ☐ Lúc nào cũng có (luôn luôn)
- ☐ Đôi khi
- ☐ Hiếm khi
- ☐ Không bao giờ

c) Sau khi sinh em bé

- ☐ Lúc nào cũng có (luôn luôn)
- ☐ Đôi khi
- ☐ Hiếm khi
- ☐ Không bao giờ

49.* Nói chung, các chuyên gia y tế có tôn trọng bạn hay không?

a) Trong khi mang thai

- ☐ Lúc nào cũng có (luôn luôn)
- ☐ Đôi khi
- ☐ Hiếm khi
- ☐ Không bao giờ

b) Trong khi chuyển dạ và đang sinh em bé

- ☐ Lúc nào cũng có (luôn luôn)
- ☐ Đôi khi
- ☐ Hiếm khi
- ☐ Không bao giờ

c) Sau khi sinh em bé

- ☐ Lúc nào cũng có (luôn luôn)
- ☐ Đôi khi
- ☐ Hiếm khi
- ☐ Không bao giờ

50.* Nói chung, các chuyên gia y tế có hữu ích hay không?

a) Trong khi mang thai

- ☐ Lúc nào cũng có (luôn luôn)
- ☐ Đôi khi
- ☐ Hiếm khi
- ☐ Không bao giờ

b) Trong khi chuyển dạ và đang sinh em bé

- ☐ Lúc nào cũng có (luôn luôn)
- ☐ Đôi khi
- ☐ Hiếm khi
- ☐ Không bao giờ

c) Sau khi sinh em bé

- ☐ Lúc nào cũng có (luôn luôn)
- ☐ Đôi khi
- ☐ Hiếm khi
- ☐ Không bao giờ

51.* Nói chung, tôi đã hài lòng với sự chăm sóc sức khoẻ tôi đã có

a) Trong khi mang thai

- ☐ Lúc nào cũng thế (luôn luôn)
- ☐ Đôi khi
- ☐ Hiếm khi
- ☐ Không bao giờ

b) Trong khi chuyển dạ và đang sinh em bé

- ☐ Lúc nào cũng thế (luôn luôn)
- ☐ Đôi khi
- ☐ Hiếm khi
- ☐ Không bao giờ

c) Sau khi sinh em bé

- ☐ Lúc nào cũng thế (luôn luôn)
- ☐ Đôi khi
- ☐ Hiếm khi
- ☐ Không bao giờ

52.* Trong thời gian mang thai, quá trình chuyển dạ, hoặc sinh đẻ, những chuyên gia y tế đã bao giờ yêu cầu bạn làm điều gì đó mà bạn không muốn làm hay không?

- ☐ Có
- ☐ Không
- ☐ Tôi không biết/ không nhớ

53.Nếu bạn trả lời CÓ, điều này là gì?

____

- ☐ Câu hỏi này không áp dụng đối với tôi

54.Các chuyên gia y tế có bao giờ hỏi bạn về việc bạn muốn người chuyên gia giúp đỡ mình là phụ nữ hay đàn ông hay không?

a) Trong khi mang thai

- ☐ Lúc nào cũng có (luôn luôn)
- ☐ Đôi khi
- ☐ Hiếm khi
- ☐ Không bao giờ
- ☐ Ý kiến________________

b) Trong khi chuyển dạ và đang sinh em bé

- ☐ Lúc nào cũng có (luôn luôn)
- ☐ Đôi khi
- ☐ Hiếm khi
- ☐ Không bao giờ
- ☐ Ý kiến________________

c) Trong ngày đầu tiên sau khi sinh em bé

- ☐ Lúc nào cũng có (luôn luôn)
- ☐ Đôi khi
- ☐ Hiếm khi
- ☐ Không bao giờ
- ☐ Ý kiến________________

55.* Bạn đã có hiểu về những thông tin mà các chuyên gia y tế đã cung cấp cho bạn hay không?

a) Trong khi mang thai

- ☐ Lúc nào cũng có (luôn luôn)
- ☐ Đôi khi
- ☐ Hiếm khi
- ☐ Không bao giờ
- ☐ Ý kiến________________

b) Trong khi chuyển dạ và đang sinh em bé

- ☐ Lúc nào cũng có (luôn luôn)
- ☐ Đôi khi
- ☐ Hiếm khi
- ☐ Không bao giờ
- ☐ Ý kiến________________

c) Trong ngày đầu tiên sau khi sinh em bé

- ☐ Lúc nào cũng có (luôn luôn)
- ☐ Đôi khi
- ☐ Hiếm khi
- ☐ Không bao giờ
- ☐ Ý kiến________________

56.* M Bạn có thể hiểu các thông tin được cung cấp bởi các chuyên gia y tế tốt hơn trong một ngôn ngữ khác phải không?

- ☐ Phải, ngôn ngữ này là __________________ (ví dụ như ngôn ngữ mẹ đẻ của bạn)
- ☐ Không
- ☐ Tôi không biết/ không nhớ

57.* M Các chuyên gia y tế có cung cấp cho bạn một dịch vụ phiên dịch (thông dịch) hay không?

a) Trong khi mang thai

- ☐ Có
- ☐ Không
- ☐ Câu hỏi không áp dụng đối với tôi

b) Trong khi chuyển dạ và đang sinh em bé

- ☐ Có
- ☐ Không
- ☐ Câu hỏi không áp dụng đối với tôi

c) Trong ngày đầu tiên sau khi sinh em bé

- ☐ Có
- ☐ Không
- ☐ Câu hỏi không áp dụng đối với tôi

58.* M Người phiên dịch có thường xuyên ở bên cạnh bạn hay không?

a) Trong khi mang thai

- ☐ Lúc nào cũng có (luôn luôn)
- ☐ Đôi khi
- ☐ Hiếm khi
- ☐ Không bao giờ
- ☐ Câu hỏi không áp dụng đối với tôi

b) Trong khi chuyển dạ và đang sinh em bé

- ☐ Lúc nào cũng có (luôn luôn)
- ☐ Đôi khi
- ☐ Hiếm khi
- ☐ Không bao giờ
- ☐ Câu hỏi không áp dụng đối với tôi

c) Trong ngày đầu tiên sau khi sinh em bé

- ☐ Lúc nào cũng có (luôn luôn)
- ☐ Đôi khi
- ☐ Hiếm khi
- ☐ Không bao giờ
- ☐ Câu hỏi không áp dụng đối với tôi

59.* M Nếu mà bạn có người phiên dịch cho bạn, người đó là ai?
(Xin hãy đọc tất cả và đánh dấu những gì là hợp lý với bạn)

a) Trong khi mang thai

- ☐ Chồng/ người yêu của tôi
- ☐ Thành viên trong gia đình/bạn bè
- ☐ Nhân viên y tế
- ☐ Con của tôi
- ☐ Thông dịch viên chuyên nghiệp
- ☐ Bệnh nhân khác, hoặc gia đình và bạn bè của một bệnh nhân khác
- ☐ Người khác *(Xin ghi rõ thêm chi tiết)*: ___________________
- ☐ Câu hỏi không áp dụng đối với tôi

b) Trong khi chuyển dạ và đang sinh em bé

- ☐ Chồng/ người yêu của tôi
- ☐ Thành viên trong gia đình/bạn bè
- ☐ Nhân viên y tế
- ☐ Con của tôi
- ☐ Thông dịch viên chuyên nghiệp
- ☐ Bệnh nhân khác, hoặc gia đình và bạn bè của một bệnh nhân khác
- ☐ Người khác *(Xin ghi rõ thêm chi tiết)*: ___________________
- ☐ Câu hỏi không áp dụng đối với tôi

c) Trong ngày đầu tiên sau khi sinh em bé

- ☐ Chồng/ người yêu của tôi
- ☐ Thành viên trong gia đình/bạn bè
- ☐ Nhân viên y tế
- ☐ Con của tôi
- ☐ Thông dịch viên chuyên nghiệp
- ☐ Bệnh nhân khác, hoặc gia đình và bạn bè của một bệnh nhân khác
- ☐ Người khác *(Xin ghi rõ thêm chi tiết)*: ___________________
- ☐ Câu hỏi không áp dụng đối với tôi

60.[M] **Bạn có hài long với cách phiên dịch và giải thích của họ hay không?**

- ☐ Có
- ☐ Không
- ☐ Tôi không biết/ không nhớ
- ☐ Câu hỏi này không áp dụng đối với tôi

61.*Trong khi mang thai, chuyển dạ, sinh em bé, và sau khi sinh em bé, bạn đã có những yêu cầu về sự chăm sóc của bạn hay những phong tục và tập quán đặc biệt, mà rồi không được sự đồng ý, hoặc sắp xếp, của các người chuyên gia y tế hay không?

- ☐ Có
- ☐ Không *(Hãy tiếp tục với câu hỏi số 64)*
- ☐ Tôi không biết/ không nhớ

62.Nếu bạn trả lời CÓ, những yêu cầu này là gì?

*i)*____________
*ii)*___________
*iii)*___________

- ☐ Câu hỏi không áp dụng đối với tôi

63. Nếu bạn trả lời CÓ, các người chuyên gia y tế đã đưa ra những lý do gì mà họ không thể cho phép hoặc sắp xếp cho bạn làm được những yêu cầu này?

*i)*____________
*ii)*___________
*iii)*___________

- ☐ Câu hỏi không áp dụng đối với tôi

64.* Có bất cứ điều gì bạn nghĩ rằng các chuyên gia y tế có thể làm khác hoặc tốt hơn hay không?

a) Trong khi mang thai?

- ☐ Có *(Điền vào câu hỏi 65a)*
- ☐ Không
- ☐ Tôi không biết/ không nhớ

b) Trong khi chuyển dạ và đang sinh em bé?

- ☐ Có *(Điền vào câu hỏi 65b)*
- ☐ Không
- ☐ Tôi không biết/ không nhớ

c) Sau khi sinh em bé?

- ☐ Có *(Điền vào câu hỏi 65c)*
- ☐ Không
- ☐ Tôi không biết/ không nhớ

65. **Nếu bạn trả lời CÓ, xin hãy cho thêm chi tiết về những điều gì mà có thể làm khác hoặc tốt hơn và ai có thể làm những điều này:**

a) **Trong khi mang thai?**

____

b) **Trong khi chuyển dạ và đang sinh em bé?**

____

c) **Sau khi sinh em bé?**

____

66. *** Xin hãy cho biết bất cứ điều gì về sự chăm sóc của bạn trong thời gian có thai, sinh đẻ, và sau khi sinh em bé mà bạn đã:**

a) **Hài lòng với**

____

b) **Không hài lòng với**

____

Suy nghĩ về việc mang thai gần đây nhất của bạn, xin hãy cho tôi biết các câu sau đây có thường xuyên đúng với sự thật hay không.

67. *** Các chuyên gia y tế thường xuyên hỏi tôi về bất kỳ thắc mắc nào mà tôi có.**

☐ Lúc nào cũng vậy (luôn luôn)
☐ Đôi khi
☐ Hiếm khi
☐ Không bao giờ

68. **Các chuyên gia y tế hay thường xuyên vội vã.**

☐ Lúc nào cũng vậy (luôn luôn)
☐ Đôi khi
☐ Hiếm khi
☐ Không bao giờ

69.* Tôi cảm thấy các sự lo lắng của tôi được sự chú ý và quan tâm nghiêm túc bởi các chuyên gia y tế.

a) Trong khi mang thai

- ☐ Lúc nào cũng vậy (luôn luôn)
- ☐ Đôi khi
- ☐ Hiếm khi
- ☐ Không bao giờ
- ☐ Câu hỏi không áp dụng đối với tôi (Không có sự chăm sóc trong thời gian mang thai)

b) Trong khi chuyển dạ và đang sinh em bé

- ☐ Lúc nào cũng vậy (luôn luôn)
- ☐ Đôi khi
- ☐ Hiếm khi
- ☐ Không bao giờ
- ☐ Câu hỏi không áp dụng đối với tôi (Không có sự chăm sóc trong thời gian mang thai)

c) Sau khi sinh em bé

- ☐ Lúc nào cũng vậy (luôn luôn)
- ☐ Đôi khi
- ☐ Hiếm khi
- ☐ Không bao giờ
- ☐ Câu hỏi không áp dụng đối với tôi (Không có sự chăm sóc trong thời gian mang thai)

70. Tôi đã phải chờ đợi quá lâu để nhận được sự chăm sóc.

a) Trong khi mang thai

- ☐ Lúc nào cũng vậy (luôn luôn)
- ☐ Đôi khi
- ☐ Hiếm khi
- ☐ Không bao giờ
- ☐ Câu hỏi không áp dụng đối với tôi (Không có sự chăm sóc trong thời gian mang thai)

b) Trong khi chuyển dạ và đang sinh em bé

- ☐ Lúc nào cũng vậy (luôn luôn)
- ☐ Đôi khi
- ☐ Hiếm khi
- ☐ Không bao giờ
- ☐ Câu hỏi không áp dụng đối với tôi (Không có sự chăm sóc trong thời gian mang thai)

c) Sau khi sinh em bé

- ☐ Lúc nào cũng vậy (luôn luôn)
- ☐ Đôi khi
- ☐ Hiếm khi
- ☐ Không bao giờ
- ☐ Câu hỏi không áp dụng đối với tôi (Không có sự chăm sóc trong thời gian mang thai)

71. * Các chuyên gia y tế đã thường xuyên thông báo cho tôi biết về các sự kiện đang xảy ra.

a) Trong khi mang thai

☐ Lúc nào cũng vậy (luôn luôn)
☐ Đôi khi
☐ Hiếm khi
☐ Không bao giờ
☐ Câu hỏi không áp dụng đối với tôi (Không có sự chăm sóc trong thời gian mang thai)

b) Trong khi chuyển dạ và đang sinh em bé

☐ Lúc nào cũng vậy (luôn luôn)
☐ Đôi khi
☐ Hiếm khi
☐ Không bao giờ
☐ Câu hỏi không áp dụng đối với tôi (Không có sự chăm sóc trong thời gian mang thai)

c) Sau khi sinh em bé

☐ Lúc nào cũng vậy (luôn luôn)
☐ Đôi khi
☐ Hiếm khi
☐ Không bao giờ
☐ Câu hỏi không áp dụng đối với tôi (Không có sự chăm sóc trong thời gian mang thai)

72.* Tôi cảm thấy thoải mái để hỏi về những điều tôi không hiểu.

a) Trong khi mang thai

☐ Lúc nào cũng vậy (luôn luôn)
☐ Đôi khi
☐ Hiếm khi
☐ Không bao giờ
☐ Câu hỏi không áp dụng đối với tôi (Không có sự chăm sóc trong thời gian mang thai)

b) Trong khi chuyển dạ và đang sinh em bé

☐ Lúc nào cũng vậy (luôn luôn)
☐ Đôi khi
☐ Hiếm khi
☐ Không bao giờ
☐ Câu hỏi không áp dụng đối với tôi (Không có sự chăm sóc trong thời gian mang thai)

c) Sau khi sinh em bé

☐ Lúc nào cũng vậy (luôn luôn)
☐ Đôi khi
☐ Hiếm khi
☐ Không bao giờ
☐ Câu hỏi không áp dụng đối với tôi (Không có sự chăm sóc trong thời gian mang thai)

73. * Các chuyên gia y tế hay đưa ra quyết định mà không đề cập tới sự mong muốn của tôi.

a) Trong khi mang thai

☐ Lúc nào cũng vậy (luôn luôn)
☐ Đôi khi
☐ Hiếm khi
☐ Không bao giờ
☐ Câu hỏi không áp dụng đối với tôi (Không có sự chăm sóc trong thời gian mang thai)

b) Trong khi chuyển dạ và đang sinh em bé

☐ Lúc nào cũng vậy (luôn luôn)
☐ Đôi khi
☐ Hiếm khi
☐ Không bao giờ
☐ Câu hỏi không áp dụng đối với tôi (Không có sự chăm sóc trong thời gian mang thai)

c) Sau khi sinh em bé

☐ Lúc nào cũng vậy (luôn luôn)
☐ Đôi khi
☐ Hiếm khi
☐ Không bao giờ
☐ Câu hỏi không áp dụng đối với tôi (Không có sự chăm sóc trong thời gian mang thai)

74.* Các chuyên gia y tế đã cho tôi những sự khuyến khích, động viên, cũng như sự an ủi.

a) Trong khi mang thai

☐ Lúc nào cũng vậy (luôn luôn)
☐ Đôi khi
☐ Hiếm khi
☐ Không bao giờ
☐ Câu hỏi không áp dụng đối với tôi (Không có sự chăm sóc trong thời gian mang thai)

b) Trong khi chuyển dạ và đang sinh em bé

☐ Lúc nào cũng vậy (luôn luôn)
☐ Đôi khi
☐ Hiếm khi
☐ Không bao giờ
☐ Câu hỏi không áp dụng đối với tôi (Không có sự chăm sóc trong thời gian mang thai)

c) Sau khi sinh em bé

☐ Lúc nào cũng vậy (luôn luôn)
☐ Đôi khi
☐ Hiếm khi
☐ Không bao giờ
☐ Câu hỏi không áp dụng đối với tôi (Không có sự chăm sóc trong thời gian mang thai)

75.* Các chuyên gia y tế có dành đủ thời gian để cung cấp những lời giải thích cho bạn hay không?

a) Trong khi mang thai

- ☐ Lúc nào cũng vậy (luôn luôn)
- ☐ Đôi khi
- ☐ Hiếm khi
- ☐ Không bao giờ
- ☐ Câu hỏi không áp dụng đối với tôi (Không có sự chăm sóc trong thời gian mang thai)

b) Trong khi chuyển dạ và đang sinh em bé

- ☐ Lúc nào cũng vậy (luôn luôn)
- ☐ Đôi khi
- ☐ Hiếm khi
- ☐ Không bao giờ
- ☐ Câu hỏi không áp dụng đối với tôi (Không có sự chăm sóc trong thời gian mang thai)

c) Sau khi sinh em bé

- ☐ Lúc nào cũng vậy (luôn luôn)
- ☐ Đôi khi
- ☐ Hiếm khi
- ☐ Không bao giờ
- ☐ Câu hỏi không áp dụng đối với tôi (Không có sự chăm sóc trong thời gian mang thai)

76.*Nói chung, bạn có cảm thấy những chuyên gia y tế đối xử khác biệt với bạn so với những người khác hay không? (Ví dụ vì ngôn ngữ hay giọng nói của bạn, văn hoá, chủng tộc hay màu da, tôn giáo, tình trạng di cư, hoặc tình trạng bảo hiểm y tế?)

- ☐ Có, luôn luôn *(Xin hãy điền rõ lý do tại sao trong câu hỏi tiếp theo, số 77)*
- ☐ Có, đôi khi *(Xin hãy điền rõ lý do tại sao trong câu hỏi tiếp theo, số 77)*
- ☐ Có, hiếm khi *(Xin hãy điền rõ lý do tại sao trong câu hỏi tiếp theo, số 77)*
- ☐ Không, không bao giờ *(Hãy tiếp tục với câu hỏi số 78)*

77.* Nếu bạn trả lời CÓ, bạn nghĩ vì những lý do nào sau đây?
(Xin hãy cho người phụ nữ trả lời câu hỏi này trước, rồi sau đó đánh dấu tất cả những gì là hợp lý với bạn)

- ☐ Ngôn ngữ hay giọng nói
- ☐ Văn hoá
- ☐ Chủng tộc/ sắc tộc
- ☐ Màu da
- ☐ Tôn giáo
- ☐ Tình trạng di cư
- ☐ Tình trạng bảo hiểm y tế
- ☐ Lý do khác *(Xin ghi rõ thêm chi tiết)*:__________
- ☐ Câu hỏi không áp dụng đối với tôi

Những câu hỏi tiếp theo có liên quan tới LỊCH SỬ SẢN KHOA của bạn. Trong phần này gồm có 8 câu hỏi.

78.* Bạn đã mang thai tổng cộng là bao nhiêu lần (bao gồm lần mang thai này)? _______

~~**79.* Bạn đã bị sẩy thai bao nhiêu lần? _______**~~

~~***Xin hãy chắc chắn rằng không có ai khác có mặt khi đưa ra câu hỏi này***~~

~~**80.* Bạn đã phá thai bao nhiêu lần? _______**~~

~~**81.* Bạn đã bị thai chết lưu bao nhiêu lần (sinh em bé chết trong bụng mẹ)? ________**~~

82.* Trong những người con bạn đang có, còn sống, bao nhiêu người đã đước sinh ra trước 37 tuần? _______

☐ Câu hỏi không áp dụng đối với tôi

83.* Bao nhiêu người đã sinh ra sau 37 tuần?________

☐ Câu hỏi không áp dụng đối với tôi

84.* Bạn đã có gặp những rắc rối về sứ khoẻ (hay nhứng biến chứng) gì trong <u>thời kỳ mang thai trước đây</u> hay không?

☐ Có
☐ Không *(Hãy tiếp tục với câu hỏi số 86)*
☐ Câu hỏi không áp dụng đối với tôi *(Hãy tiếp tục với câu hỏi số 86)*

85.* Nếu bạn đã gặp những rắc rối về sức khoẻ (hay những biến chứng) trong thời kỳ mang thai trước đây, những điều này là gì?
(Xin hãy cho người phụ nữ trả lời câu hỏi này trước, rồi sau đó đánh dấu tất cả những gì là hợp lý với bạn)

☐ Mổ để lấy thai nhi [*Caesarean section/ Césarienne*]
☐ Thiếu máu [*Anaemia/ Anémie*]
☐ Huyết áp cao [*High blood pressure/ Hypertension arterielle*]
☐ Tiền sản giật (tăng huyết áp thai kỳ) [*Preeclampsia (gestational hypertension) / Pre-eclampsie (hypertension artérielle gravidique*]
☐ Sinh non (em bé sinh thiếu tháng) [*Preterm labour/ Travail prématuré*]
☐ Huyết khối tĩnh mạch sâu (nghẽn mạch sâu) [*Deep vein thrombosis/ Thrombose veineuse profonde*]
☐ Tiểu đường thai kỳ [*Gestational diabetes/ Diabète de grossesse*]
☐ Nhau thai tiền đạo [*Placenta praevia/ Placenta praevia*]
☐ Nhau thai bong non (bánh nhau bong ra khi thai chưa được sổ ra ngoài) [*Placental abruption/ Décollement placentaire*]
☐ Nhiễm trùng tiết niệu [*Urinary tract infection/ Infection urinaire*]
☐ Đau lưng [*Severe back pain/ Douleurs sévères au dos*]
☐ Ối vỡ non [*Preterm rupture of membranes/ Rupture premature des membranes*]
☐ Trầm cảm (buồn chán) [*Depression/ Dépression*]
☐ Bệnh khác *(Xin ghi rõ thêm chi tiết)*: ________________________
☐ Tôi không biết
☐ Câu hỏi không áp dụng đối với tôi

Những câu hỏi cuối cùng sau đây có liên quan tới BẠN VÀ GIA ĐÌNH CỦA BẠN. Trong phần này gồm có 27 câu hỏi.

86. * Tình trạng hôn nhân của bạn hiện tại là như thế nào?

☐ Đã kết hôn
☐ Sự kết hợp đồng thuận (có người yêu nhưng chưa kết hôn)
☐ Ở goá
☐ Tách rời nhau
☐ Đã ly hôn (ly dị)
☐ Độc thân

87.* Bạn đang sống với ai?

	Có	*Không*
Chồng/ người yêu nam	☐	☐
Người yêu nữ	☐	☐
Cha/ Mẹ của tôi	☐	☐
Anh/ Chị/ Em của tôi	☐	☐
Cha/ Mẹ của người yêu	☐	☐
Anh/ Chị/ Em của người yêu	☐	☐
Bạn của tôi	☐	☐
Con của tôi (ngoài trừ em bé sơ sinh)	☐	☐
Người khác*(Xin ghi rõ thêm chi tiết)*: ____________________________	☐	☐
Không có ai, tôi sống một mình với con của tôi	☐	☐
Không có ai, tôi sống một mình	☐	☐

88. *** Bao nhiêu người con của bạn đang sống vời bạn (bao gồm cả em bé mời sinh)? ____________**

89.* M **Bao nhiêu người con của bạn đã được sinh ra tại quốc gia này (bao gồm cả em bé mới sinh)? ____________**

90.* **Ngày tháng năm sinh của bạn là gì? ~~________( )~~ ________(tháng) ________ (năm)**

91.* M **Mẹ của bạn đã sinh ra ở nơi đâu (quốc gia nào)? ____________**

92.* M **Cha của bạn đã sinh ra ở nơi đâu (quốc gia nào)? ____________**

Những câu hỏi tiếp theo đề cấp chi tiết hơn về lịch sử nhập cư của bạn. Chúng tôi quan tâm đến thông tin này bởi vì chúng tôi muốn tìm hiểu thêm về những kinh nghiệm của những người mới đến quốc gia này. Bất kỳ thông tin nào mà bạn cung cấp cho chúng tôi sẽ được giữ bí mật, không có thông tin nào sẽ được cung cấp cho cơ quan xuất nhập cảnh hay cơ quan di trú. Nếu bạn trả lời những câu hỏi này, việc này sẽ không ảnh hưởng đến quá trình đăng ký tị nạn, thường trú, hoặc công dân của bạn.

93.* M **Tình trạng di trú của bạn hiện tại là như thế nào?**
(Xin hãy cho người phụ nữ trả lời trước, rồi sau đó lựa chọn một câu trả lời thích hợp nhất)

- ☐ Người nhập cư (tình trạng thường trú)
- ☐ Người tị nạn
- ☐ Người thỉnh cầu tị nạn/ Người tìm tị nạn
- ☐ Công nhân tạm thời/ Người chăm sóc sống tại nhà của chủ (Caregiver)
- ☐ Công dân tạm thời
- ☐ Sinh viên
- ☐ Khách du lịch
- ☐ Không có tình trạng nào
- ☐ Không có giấy tờ
- ☐ Người công dân (có quốc tịch)
- ☐ Tình trạng của tôi khác với những điều ghi ở trên *(Xin ghi rõ thêm chi tiết)*:_________________

94.M **Tình trạng di trú này của bạn đã được bao nhiêu lâu rồi?**

________ (ngày) ________ (tuần) ________(tháng) ________ (năm)

95.M **Tình trạng di trú của bạn đã có thay đổi kể từ khi bạn đến đây hay không?**

- ☐ Có
- ☐ Không *(Xin hay tiếp tục với câu hỏi số 97)*

96.* [M] Tình trạng di trú của bạn trước đây là như thế nào?

☐ Người nhập cư (tình trạng thường trú)
☐ Người tị nạn
☐ Người thỉnh cầu tị nạn/ Người tìm tị nạn
☐ Công nhân tạm thời/ Người chăm sóc sống tại nhà của chủ (Caregiver)
☐ Cong dân tạm thời
☐ Sinh viên
☐ Khách du lịch
☐ Không có tình trạng nào
☐ Không có giấy tờ
☐ Tình trạng của tôi khác với những điều ghi ở trên *(Xin ghi rõ thêm chi tiết)*:_________________
☐ Câu hỏi không áp dụng đối với tôi (Tình trạng của tôi không có thay đổi)

97.[M] Bạn đã bao giờ có tình trạng là người tị nạn hay không?

☐ Có
☐ Không
☐ Tôi không biết/ không nhớ

98.[M] Bạn đã bao giờ bị giam tại một trung tâm giam giữ người nhập cư hay không?

☐ Có
☐ Không *(Xin hay tiếp tục với câu hỏi số 101)*

99.[M] Nếu bạn trả lời CÓ, bạn đã bị giam bao nhiêu lâu?

________ (ngày) ________ (tuần) ________(tháng) ________ (năm)

☐ Câu hỏi không áp dụng đối với tôi

100. * [M] Nếu bạn trả lời CÓ, bạn đã bị giam trong khi mang thai lần này, phải hay không?

☐ Phải
☐ Không
☐ Câu hỏi không áp dụng đối với tôi

101. * Người trả tiền cho các dịch vụ sức khoẻ của bạn là ai?

	Có	*Không*	*Tôi không biết*
Bảo hiểm y tế của công chúng (RAMQ)	☐	☐	☐
Bảo hiểm y tế tư nhân	☐	☐	☐
Bảo hiểm đặc biệt do trính phủ tài trợ cho những người tị nạn và người đang tìm tị nạn	☐	☐	☐
Tôi là người trả tiền cho các dịch vụ sức khoẻ của tôi	☐	☐	☐

102. *** Cấp độ giáo dục cao nhất mà bạn đã hoàn thành là gì?**

- ☐ Trường tiểu học
- ☐ Trường trung học
- ☐ Bằng tốt nghiệp trường sau trung học (ví dụ như, bằng tốt nghiệp trường thương mại, trường cao đẳng, hay trường đại học)
- ☐ Bằng tốt nghiệp sau đại học (như bằng thạc sĩ hay tiến sĩ)
- ☐ Không có gì hết

103. **[M] Bạn có được phép (hợp pháp) làm việc tại quốc gia này hay không?**

- ☐ Có
- ☐ Không
- ☐ Tôi không biết

104. *** Công việc (có tiền lương) mà bạn đã làm trước khi sinh em bé là gì? (ví dụ như bác sĩ, giáo viên, nhân viên nhập dữ tài liệu, người hỗ trợ tại nhà điều dưỡng, quản gia, người trồng rau, người điều hành máy dệt nhuộm, người dọn dẹp khách sạn, người làm cho một trung tâm điện thoại)**

***(Xin ghi rõ thêm chi tiết)*: ___________________**

- ☐ Câu hỏi này không áp dụng đối với tôi (Tôi không có làm việc)

~~**105.** *** Bạn đã có trở lại đi làm từ khi em bé được sinh ra hay không?**~~

~~**106.** *** Nếu bạn trả lời CÓ, công việc hiện tại của bạn là gì? (ví dụ như bác sĩ, giáo viên, nhân viên nhập dữ tài liệu, người hỗ trợ tại nhà điều dưỡng, quản gia, người trồng rau, người điều hành máy dệt nhuộm, người dọn dẹp khách sạn, người làm cho một trung tâm điện thoại)**~~

107. *** Suy nghĩ về toàn bộ những người trong nhà của bạn, bạn thuộc về nhóm thu nhập (trước khi khai thuế) nào sau đây?**
(Xin hãy đọc các lựa chọn sau đây. Điền vào ô trống số tiền thu nhập áp dụng với địa phương)

- ☐ < __$11,000________
- ☐ __$11,000___ tới ___$20,999___
- ☐ __$21,000___ tới ___$40,999___
- ☐ __$41,000___ tới ___$60,999___
- ☐ __$61,000___ tới __$80,999____
- ☐ ≥ _$81,000___

108. *** Thu nhập này hỗ trợ được bao nhiêu người (bao gồm em bé mới sinh)? ______________**

109. *** Bạn thường xuyên sử dụng ngôn ngữ nào nhiều nhất ở nhà?**

____

110. *** M Bạn biết được bao nhiêu về ngôn ngữ của quốc gia này?**

English

	Thông thạo	*Tốt*	*Có khó khăn*	*Không biết một chút nào*
Nói chuyện	☐	☐	☐	☐
Đọc	☐	☐	☐	☐
Viết	☐	☐	☐	☐
Hiểu	☐	☐	☐	☐

111. **M Bạn biết được bao nhiêu về ngôn ngữ của quốc gia này?**

French

	Thông thạo	*Tốt*	*Có khó khăn*	*Không biết một chút nào*
Nói chuyện	☐	☐	☐	☐
Đọc	☐	☐	☐	☐
Viết	☐	☐	☐	☐
Hiểu	☐	☐	☐	☐

112. *** Cuộc phỏng vấn đã kết thúc. Nếu bạn muốn nói thêm điều gì liên quan tới những chủ đề chúng tôi đã hỏi hoặc chia sẽ thêm bất cứ điều gì, xin hãy ghi những điều đó sau đây.**
