# Supplementary material for: Migrant-friendly maternity care in Montreal, Canada: A cross-sectional study on migrant women’s care perspectives
Source: PLoS One. 2025 Aug 21;20(8):e0330830. doi: 10.1371/journal.pone.0330830 (PMC12370051; doi:10.1371/journal.pone.0330830)
Supplement: S10 Appendix — (PDF) [file pone.0330830.s010.pdf]

**MFMC Montreal Supplemental Questions - Arabic Version**		Study ID:	
START TIME:		Interviewer's Name:	
END TIME:		Interview date:	

نحن ممتنون أنك أجبتي واحدة من الاستبيانات. لدينا بعض الأسئلة الإضافية حول مواضيع لا يغطيها ذلك الاستبيان. أول مجموعة من الأسئلة تشير إلى صحتك العامة ما قبل الحمل.

1. **هل لديك أي حالة أو مرض (*مثلا: السكري، أمراض القلب، الربو، التهاب المفاصل، ملاريا، مرض السل، فيروس نقص المناعة، إلتهاب الكبد الوبائي ج، ديدان*)؟**

- ❑ نعم (*يرجى التحديد*) ________________
- ❑ لا (*إنتقل إلى Q4*)

2. **هل تلقيتي علاج لكل هذه الأمراض؟**

- ❑ نعم، تم علاج كل الأمراض
- ❑ لا، لم يتم علاج كل الأمراض، أو تم علاج بعض منها.

3. **هل سبق وأوقفتي رعاية أو علاج لأي من حالاتك أو أمراضك؟**

- ❑ نعم (*يرجى التحديد*) ________________
- ❑ لا

4. **ما هو وزنك المعتاد (وانت لست حامل)؟**

_________ كيلو (kg) _________غرام (g)/ _________ رطلا (lbs) _______ أوقية (oz)

5. **ما هو طولك؟**

_________ قدم (ft) _________ بوصة (inches)/ ________ متر (m) _______ سنتيمتر(cm)

6. **أي من العبارات التالية تنطبق على منزلك؟**

	نعم	*لا*
هو كبير كفاية لعدد الأفراد المقيمين معي	☐	☐
أنه دافئ كفاية في فصل الشتاء	☐	☐
إنه هادئ كفاية	☐	☐
أنه خالي من العفن والحشرات الضارة (مثلاً: الحشرات أو الفئران)	☐	☐
أنه خالي من الدخان في الداخل (بما في ذلك دخان السجائر)	☐	☐
أنه آمن بنيويا (أي المبنى قوي)	☐	☐
أنه في حي خالي من تلوث الهواء/حيث تكون نسبة التلوث منخفضة	☐	☐
إنه في حي آمن (أي خالي من الجريمة)	☐	☐

7. **رمزك البريدي يساعدنا على معرفة المزيد عن منطقتك، ما هو رمزك البريدي؟**

☐☐☐ ☐☐☐

لدينا 4 اسئلة حول التخطيط للحمل التي نود أن نسأل.

8. عندما حملتي بهذا الطفل، هل كنت تريدين أن تحملي في ذلك الوقت؟

- ❑ نعم *(انتقل إلى Q12)*
- ❑ لا
- ❑ غير متأكدة

9. إذا كنت غير متأكدة أو لم ترغبي في أن تحملي، هل استخدمتي شيئاً لمنع الحمل؟
(راجع القائمة في Q10 للحصول على أمثلة إذا لزم الأمر)

- ❑ نعم
- ❑ لا *(انتقل إلى Q11)*

10. إذا كان نعم، ماذا إستخدمتي؟
(اسمح للأم بالرد، وضع علامة على كل ما ينطبق، ومن ثم انتقل إلى Q12)

- ❑ واق (Préservatif)
- ❑ الرضاعة
- ❑ حبوب منع الحمل
- ❑ حقن ديبو بروفيرا (Depo Provera)
- ❑ أداة داخل الرحم
- ❑ مراقبة الدورة الشهرية
- ❑ إعتقاد أن أحدكم كان عقيم
- ❑ سحب الجهاز التناسلي خلال الجماع
- ❑ غطاء عنق الرحم
- ❑ إدراج كبسولة تحت جلد الذراع (Norplant)
- ❑ الامتناع عن الجماع
- ❑ أخرى *(يرجى التحديد)*________________________________
- ❑ N/A

11. إذا لم تستخدمي شيئاً لمنع الحمل، ما السبب؟
(اسمح للأم بالرد، وضع علامة على كل ما ينطبق)

- ❑ عدم إمكانية زيارة عيادة أو مقدم رعاية صحية
- ❑ التأثيرات الجانبية
- ❑ عدم إمكانية تحمل النفقات
- ❑ أسباب دينية
- ❑ الزوج لا يسمح/العائلة لا تسمح بها
- ❑ أخرى *(يرجى التحديد)* ________________________________
- ❑ N/A

لدينا 5 اسئلة حول صحة لثتك وأسنانك التي نود أن نسأل.

12. **بشكل عام، كيف تقيمي صحة أسنانك ولثتكي؟**
(اقرأ بصوت عالي وضع علامة على ما ينطبق)

- ❑ ممتازة
- ❑ جيدة جداً
- ❑ جيدة
- ❑ مقبولة
- ❑ ضعيفة
- ❑ لا أعلم

13. **هل تعتقدي أن لديك أمراض اللثة؟**

- ❑ نعم
- ❑ لا
- ❑ لا أعلم

14. **هل تلقيتي علاج لأمراض اللثة مثل مثل تحجيم الجذر، وسحل الجذر، وتسمى أحياناً "التنظيف العميق"؟**

- ❑ نعم
- ❑ لا
- ❑ لا أعلم

15. **هل سبق وقيل لك من قبل ممارس طب الأسنان أنك فقدتي العظم حول الأسنان؟**

- ❑ نعم
- ❑ لا
- ❑ لا أعلم

16. **عدا عن تنظيف أسنانك بفرشاة الأسنان، في الأيام السبعة الماضية، كم مرة إستخدمتي خيط تنظيف الأسنان أو أي أداة أخرى لتنظيف ما بين الأسنان؟**

__________ (عدد المرات)

- ❑ لا أعلم

في بعض البلدان، هناك تقليد حيث تخضع الفتاة الشابة لبتر جزء من اعضاؤها التناسلية الانثوية لأسباب تقليدية (أي ختان الاناث). نود أن نسألك سؤالين عن هذه العادة.

17. **هل حصل هذا لك من قبل؟**

- ❑ نعم
- ❑ لا *(انتقل إلى Q19)*

18. **إذا نعم، هل تمت خياطة هذه المنطقة؟**

- ❑ نعم
- ❑ لا
- ❑ لا أعلم

لدينا 9 اسئلة حول التنقل إلى بلد جديد التي نود أن نسأل.

19. قبل ولادتك الأخيرة، أين ومتى ولدتي؟

________________(بلد)، ___________ (سنة)
________________(بلد)، ___________ (سنة)
________________(بلد)، ___________ (سنة)
________________(بلد)، ___________ (سنة)

❑ **N/A** لا ولادات سابقة

20. كم كان عمرك عندما أتيتي للإقامة في كندا؟ __________ (سنة)

21. هل هناك شخصاً قبل أن يكون مسؤلاً عنك أو متكفلاً بك هنا قدم طلباً لتأتي إلى كندا(Parrainage)؟

❑ نعم
❑ لا *(انتقل إلى Q23)*

22. إذا نعم، من؟
(اسمح للأم بالرد، وضع علامة على ما ينطبق)

❑ زوج
❑ والدين
❑ ولد
❑ مؤسسة خاصة (مثلاً كنيسة، منظمة غير حكومية)
❑ حكومة
❑ أخرى *(يرجى التحديد)*____________________

23. في أي بلد ولد والد طفلك؟ ___________________(بلد)

❑ لا أعلم

24. هل والد الطفل يسكن معك؟

❑ نعم
❑ لا

25. هل هناك روابط دم بينك وبين والد طفلك؟

❑ نعم
❑ لا

26. إذا كان لديك وظيفة مدفوعة الأجر قبل إنجاب طفلك، متى أوقفتي العمل؟

__________ (الشهر)/ ________(السنة)

❑ لم أكن أعمل
❑ لم أوقف العمل

27. إذا تكفلتي بنفقات الخدمات الطبية في كندا خلال آخر حمل، ولادة، أو فترة ما بعد الولادة، ما هي الخدمات التي تكفلت بها، وكم كلفتها؟
(اقرأ بصوت عالي وضع علامة على كل ما ينطبق)

☐ موعد مع أخصائي صحة عامة $________
☐ فحص جسدي $________
☐ تحليل دم $________
☐ فحص عنق الرحم $________
☐ الكشف عن العيوب الولادية (مثلاً: متلازمة داون/Down Syndrome) $________
☐ التصوير بالموجات فوق الصوتية $________
☐ خدمات الصحة النفسية $________
☐ دروس الحمل/الولادة $________
☐ أدوية $________
☐ خدمات عند الولادة $________
☐ أخرى *(يرجى التحديد)*__________________ $________
☐ N/A

لدينا 7 اسئلة حول الصحة خلال الحمل التي نود أن نسأل.

28. أي من الخيارات التالية تصف عادات تدخين التبغ أثناء حملك الأخير؟
(اقرأ بصوت عالي وضع علامة على ما ينطبق)

☐ لم تدخني
☐ دخنتي أحياناً
☐ دخنتي التبغ كل يوم *(يرجى تحديد عدد المرات التي كنت تدخيني فيها يومياً)*______________________

29. ما كان وزنك في نهاية حملك؟

________ كيلو (kg) ________غرام (g)/ ________ رطلا (lbs) ________ أوقية (oz)

30. كم مرة في الأسبوع الماضي اكلتي/شربتي الأطعمة التالية؟
(اقرأ بصوت عالي ودون عدد المرات)

______ العدس والفاصوليا
______ الخضار الورقية الخضراء (كالسبانخ)
______ كبد
______ الحمضيات (كالبرتقال)
______ الخبز الكامل الحبوب
______ عصير الليمون المحصن بالفيتامين D
______ حليب البقر

31. هل أخذتي فيتامينات ما قبل الولادة، أو تكملة حمض الفوليك يومياً شهراً قبل أن تحملي على الأقل؟

☐ نعم *(انتقل إلى Q33)*
☐ لا

32. إذا لا، ما السبب؟
(اسمح الأم للرد وضع علامة على كل ما ينطبق)

☐ لم تعرف لما كان يستخدم
☐ لم تجده
☐ لم تملك المال لشراءه
☐ لم يكن متوفر
☐ لم تكن بحاجة إليه
☐ لم يقال لها أن تأخذه
☐ أخرى *(يرجى التحديد)*________________________
☐ N/A

---

33. <u>خلال حملك</u>، هل أخذتي فيتامينات ما قبل الولادة يومياً؟

☐ نعم *(انتقل إلى Q35)*
☐ لا

---

34. إذا لا، ما السبب؟
(اسمح الأم للرد وضع علامة على كل ما ينطبق)

☐ لم تعرف لما كان يستخدم
☐ لم تجده
☐ لم تملك المال لشراءه
☐ لم يكن متوفر
☐ لم تكن بحاجة إليه
☐ لم يقال لها أن تأخذه
☐ أخرى *(يرجى التحديد)*________________________
☐ N/A

---

35. وهذا يختتم مقابلتنا. هل هناك أي شيء أخر تودي أن تسألي أو تضيفي حول المواضيع الذي ناقشناه؟
