# Supplementary material for: Migrant-friendly maternity care in Montreal, Canada: A cross-sectional study on migrant women’s care perspectives
Source: PLoS One. 2025 Aug 21;20(8):e0330830. doi: 10.1371/journal.pone.0330830 (PMC12370051; doi:10.1371/journal.pone.0330830)
Supplement: S11 Appendix — (PDF) [file pone.0330830.s011.pdf]

| MFMC Montreal Supplemental Questions - English Version | | Study ID: | |
|---|---|---|---|
| START TIME: | | Interviewer's Name: | |
| END TIME: | | Interview date: | |

***We are grateful that you have answered one of our questionnaires. We have some additional questions on topics not completely covered in that questionnaire. The first set of questions refers to your pre-pregnancy general health.***

**1. Do you have any conditions or illnesses (e.g., diabetes, heart disease, asthma, arthritis, malaria, TB, HIV, Hepatitis C, worms)?**

☐ Yes *(please specify)* ___________________________
☐ No *(Skip to Q4)*

**2. Have you received treatment for all of these?**

☐ Yes, all were treated
☐ No, none or only some were treated

**3. Did you ever stop care or treatment for any of your conditions or illnesses?**

☐ Yes *(please specify)* ___________________________
☐ No

**4. What is your usual weight (when you are not pregnant)?**

_________(kg) ________ (g)/ _________(lbs) _______(oz)

**5. What is your height?**

_______ (ft) _______(inches)/ ______ (m) _____ (cm)

**6. Which of the following statements are true about your home?**

| | *Yes* | *No* |
|---|---|---|
| It is large enough for the number of people who live with me | ☐ | ☐ |
| It is warm enough in the winter | ☐ | ☐ |
| It is quiet enough | ☐ | ☐ |
| It is free of mould and pests (e.g., insects or rats) | ☐ | ☐ |
| It is free of indoor smoke (including cigarette smoke) | ☐ | ☐ |
| It is structurally safe (i.e., building is strong) | ☐ | ☐ |
| It is in a neighbourhood with low/no air pollution | ☐ | ☐ |
| It is in a safe neighbourhood (i.e., there is no crime) | ☐ | ☐ |

**7. Your postal code helps us know more about your neighborhood, what is your postal code?**

☐☐☐ ☐☐☐

***We have 4 questions about planning to become pregnant that we would like to ask.***

**8. When you got pregnant for this baby, did you want to get pregnant at that time?**

- ☐ Yes *(Skip to Q12)*
- ☐ No
- ☐ Unsure

---

**9. If you were unsure or did not want to get pregnant, did you use something to prevent pregnancy?**
***(See list in Q10 for examples if needed)***

- ☐ Yes
- ☐ No *(Skip to Q11)*

---

**10. If YES, what did you use?**
***(Allow mother to answer and check all that apply, then Skip to Q12)***

- ☐ Condom
- ☐ Breastfeeding
- ☐ The pill
- ☐ Depo-Provera injection
- ☐ Intrauterine device (IUD)
- ☐ Observation of monthly cycle
- ☐ Self or partner thought to be sterile
- ☐ Withdrawal (“pull-out”)
- ☐ Diaphragm/cervical cap
- ☐ Insert under the skin of the arm (Norplant)
- ☐ Abstinence
- ☐ Other *(please specify)* _________________________
- ☐ N/A

---

**11. If you did not use something to prevent pregnancy, why not?**
***(Allow mother to answer and check all that apply)***

- ☐ No access to clinic or healthcare provider
- ☐ Side effects
- ☐ Could not afford it
- ☐ Religious reasons
- ☐ Husband/family do not allow it
- ☐ Other *(please specify)* _________________________________
- ☐ N/A

---

***We have 5 questions about the health of your gums and teeth that we would like to ask.***

**12. Overall, how would you rate the health of your teeth and gums?**
***(Read aloud and check one)***

- ☐ Excellent
- ☐ Very good
- ☐ Good
- ☐ Fair
- ☐ Poor
- ☐ Don't know

**13. Do you think you might have <u>gum disease</u>?**

- ☐ Yes
- ☐ No
- ☐ Don't Know

**14. Have you ever had <u>treatment for gum disease</u> such as root scaling and root planing, sometimes called "deep cleaning"?**

- ☐ Yes
- ☐ No
- ☐ Don't Know

**15. Have you ever been told by a dental professional that you <u>lost bone around your teeth</u>?**

- ☐ Yes
- ☐ No
- ☐ Don't Know

**16. Aside from brushing your teeth with a toothbrush, in the last seven days, how many times did you use <u>dental floss</u> or any other device to clean between your teeth?**

___________**(number of times)**

- ☐ Don't Know

***In some countries, there is a practice in which a young girl may have part of her private area cut for traditional reasons (i.e., female circumcision). We would like to ask you 2 questions about this practice.***

**17. Was this ever done to you?**

- ☐ Yes
- ☐ No *(Skip to Q19)*

**18. If YES, was this area sewn closed?**

- ☐ Yes
- ☐ No
- ☐ Don't know

***We have 9 questions about moving to a new country that we would like to ask.***

**19. Prior to your most recent birth, where and when did you give birth?**

___________________________ (country), __________ (year)
___________________________ (country), __________ (year)
___________________________ (country), __________ (year)
___________________________ (country), __________ (year)

☐ N/A (no previous births)

**20. How old were you when you came to Canada to stay? ____________(years)**

**21. Did someone apply for you to come to Canada who was responsible for you here (i.e., "sponsored" you)?**

☐ Yes
☐ No *(Skip to Q23)*

**22. If YES, who?**
***(Allow mother to answer and check one)***

☐ Husband
☐ Parent
☐ Child
☐ Private organization (e.g., church, non-government organization)
☐ Government
☐ Other *(please specify)* __________________________

**23. In which country was the father of your baby born? __________________________ (country)**

☐ Don't know

**24. Is the father of the baby living with you?**

☐ Yes
☐ No

**25. Is the father of your baby blood related to you?**

☐ Yes
☐ No

**26. If you had a paid job before the baby was born, when did you stop working?**

**___________________(month)/___________ (year)**

☐ Was not working
☐ Did not stop working

**27. If you paid for care or medical services <u>in CANADA</u> during your most recent pregnancy, birth, or after birth, what did you pay for and how much did you pay?**
***(Read aloud and check all that apply)***

- ☐ Appointment with healthcare professional $ ________
- ☐ Physical exam $ ________
- ☐ Blood test $ ________
- ☐ Cervical exam/pap test $ ________
- ☐ Screening for birth defects (e.g., Down Syndrome) $ ________
- ☐ Ultrasound scans $ ________
- ☐ Mental health services $ ________
- ☐ Pregnancy/childbirth classes $ ________
- ☐ Medication $ ________
- ☐ Services for the birth $ ________
- ☐ Other *(please specify)* ________________________ $ ________
- ☐ N/A

***We have 7 questions about pregnancy health that we would like to ask.***

**28. Which of the following best describes your tobacco smoking habits during your most recent pregnancy?**
***(Read aloud and check one)***

- ☐ You did not smoke
- ☐ You smoked occasionally
- ☐ You smoked tobacco each day *(please specify number of times you smoked in a day)* _________

**29. What was your weight at the end of your pregnancy just before giving birth?**

_________(kg) ________ (g)/ __________(lbs) _______(oz)

**30. How many times in the last week did you eat/drink any of the following foods?**
***(Read aloud and record number of times)***

- Lentils and beans _______
- Dark green leafy vegetables (e.g., spinach) _______
- Liver _______
- Citrus fruits (e.g., oranges) _______
- Whole grain bread _______
- Vitamin D fortified orange juice _______
- Cow's milk _______

**31. <u>At least one month before you became pregnant</u>, did you take a prenatal vitamin or folic acid supplement daily?**

- ☐ Yes *(Skip to Q33)*
- ☐ No

**32. If NO, why not?**
***(Allow mother to answer and check all that apply)***

- ☐ Did not know what it was for
- ☐ Could not find it
- ☐ Did not have money to buy it
- ☐ Not available
- ☐ Did not need it
- ☐ Was not told to take it
- ☐ Other *(please specify)* ______________________
- ☐ N/A

---

**33. <u>During your pregnancy</u>, did you take a prenatal vitamin daily?**

- ☐ Yes *(Skip to Q35)*
- ☐ No

---

**34. If NO, why not?**
***(Allow mother to answer and check all that apply)***

- ☐ Did not know what it was for
- ☐ Could not find it
- ☐ Did not have money to buy it
- ☐ Not available
- ☐ Did not need it
- ☐ Was not told to take it
- ☐ Other *(please specify)* ______________________
- ☐ N/A

---

**35. That concludes our interview. Is there anything else you would like to say about the topics we've covered? Or anything else you would like to add?**

---
