# Supplementary material for: Migrant-friendly maternity care in Montreal, Canada: A cross-sectional study on migrant women’s care perspectives
Source: PLoS One. 2025 Aug 21;20(8):e0330830. doi: 10.1371/journal.pone.0330830 (PMC12370051; doi:10.1371/journal.pone.0330830)
Supplement: S12 Appendix — (PDF) [file pone.0330830.s012.pdf]

MFMC Montréal Questions Supplémentaires – Version française		Study ID:	
L'HEURE AU DEBUT:		Nom de l'intervieweur:	
L'HEURE A LA FIN:		Date de l'entrevue:	

Nous vous remercions d'avoir répondu à un de nos questionnaires. Nous avons quelques questions supplémentaires qui abordent des sujets qui n'ont pas été entièrement abordés dans l'autre questionnaire. Le premier groupe de questions porte sur votre santé générale avant votre grossesse.

1. Souffrez-vous d'une ou plusieurs maladies ou d'affections médicales chroniques? *(ex: diabète, maladie du coeur, asthme, arthrite, malaria, tuberculose, VIH, hépatite C, schistosomiase, strongyloïdose)*

☐ Oui *(Veuillez préciser)* ___________________________
☐ Non *(Veuillez passer à la Q4)*

2. Avez-vous reçu un traitement pour toutes ces maladies?

☐ Oui, elles ont toutes été traitées.
☐ Non, aucune ou seulement quelques-unes d'entre-elles ont été traitées.

3. Avez-vous déjà arrêté un traitement pour vos maladies?

☐ Oui *(Veuillez préciser)* ___________________________
☐ Non

____

4. Combien pesez-vous normalement (lorsque vous n'êtes pas enceinte)?

_________kg __________(g)/_________(livres) __________(onces)

5. Combien mesurez-vous?

_______ (pieds)________ (pouces)/_______ (m)_______ (cm)

6. Lesquelles de ces remarques s'appliquent au cas de votre maison?

	Oui	*Non*
Elle est assez grande pour le nombre de gens qui y habitent avec moi	☐	☐
Il y fait assez chaud l'hiver	☐	☐
C'est suffisamment silencieux.	☐	☐
Il n'y a pas de moisissures ni de vermine (ex: cafards, rats)	☐	☐
Il n'y a pas de fumée (fumée de cigarette inclus)	☐	☐
La maison est sécuritaire au niveau de sa structure (l'immeuble est solide)	☐	☐
Elle se situe dans un quartier sans, ou avec peu de pollution dans l'air	☐	☐
Elle se situe dans un quartier sécuritaire (il n'y a pas de crime)	☐	☐

7. Votre code postal nous aide à mieux cerner votre quartier. Quel est votre code postal?

☐☐☐ ☐☐☐

Nous avons 4 questions que nous voudrions vous poser au sujet de la planification d'une grossesse.

8. Au moment où vous êtes tombée enceinte de ce bébé, vouliez-vous tomber enceinte?

- ☐ Oui *(Veuillez passer à Q12)*
- ☐ Non
- ☐ Incertaine

9. Si vous ne vouliez pas, ou si vous étiez incertaine de vouloir être enceinte, avez-vous utilisé quelque chose pour empêcher les grossesses?
(Si besoin, consultez la liste à la Q10 pour des exemples)

- ☐ Oui
- ☐ Non *(Veuillez passer à Q11)*

10. Si OUI, qu'avez-vous utilisé?
(Laissez répondre puis cochez toutes les réponses qui s'appliquent, puis passez à Q12

- ☐ Préservatif
- ☐ Allaitement
- ☐ Pilule contraceptive
- ☐ Contraceptif injectable Depo-Provera
- ☐ Dispositif intra-utérin (DIU)
- ☐ Observation du cycle mensuel
- ☐ Vous pensiez que vous ou votre partenaire étiez sterile (chirurgie pour empêcher la grossesse)
- ☐ Rétraction ("pull-out")
- ☐ Diaphragm
- ☐ Insertion sous la peau du bras (Norplant)
- ☐ Abstinence
- ☐ Autre *(Veuillez préciser)* ________________________
- ☐ S/O

11. Si vous n'avez pas utilisé quelque chose pour empêcher les grossesses, pourquoi pas?
(Laissez répondre puis cochez toutes les réponses qui s'appliquent)

- ☐ Aucun accès à une clinique ou à un fournisseur de soins de santé
- ☐ Effets secondaires
- ☐ Vous n'aviez pas les moyens
- ☐ Raisons religieuses
- ☐ Votre mari/votre famille vous l'ont interdit
- ☐ Autre *(Veuillez préciser)* ________________________________
- ☐ S/O

Nous avons 5 questions que nous voudrions vous poser au sujet de la santé de vos gencives et de vos dents.

12. Dans l'ensemble comment noteriez-vous la santé de vos dents et de vos gensives?
(Lisez à voix haute puis cochez une réponse)

- ☐ Excellente
- ☐ Très bien
- ☐ Bien
- ☐ Satisfaisante
- ☐ Faible
- ☐ Ne sais pas

13. Pensez-vous avoir la gingivite?

- ☐ Oui
- ☐ Non
- ☐ Ne sais pas

14. Avez-vous déjà eu un traitement pour la gingivite tel que le retrait des substances sur la racine de la dent (ex: détartrage, curettage du cément radiculaire)?

- ☐ Oui
- ☐ Non
- ☐ Ne sais pas

15. Est-ce qu'un professionnel dentaire vous a déjà dit que vous avez perdu de l'os autour de vos dents?

- ☐ Oui
- ☐ Non
- ☐ Ne sais pas

16. Mise à part le nettoyage avec une brosse à dents, dans les sept derniers jours combien de fois avez-vous utilisé du fil dentaire ou un autre dispositif de nettoyage pour nettoyer entre vos dents?

Nombre de fois:___________

- ☐ Ne sais pas

Dans certains pays, il y a une pratique qui existe selon laquelle une jeune fille peut avoir une partie de son corps intime coupé pour des raisons traditionnelles (i.e., excision féminine). Nous voudrions vous poser 2 questions au sujet de cette pratique.

17. Est-ce que ça vous est arrivé?

- ☐ Oui
- ☐ Non *(Veuillez passer à Q19)*

18. Si OUI, est ce que cet endroit a été recousu?

- ☐ Oui
- ☐ Non
- ☐ Ne sais pas

Nous avons 9 questions au sujet du déménagement vers un nouveau pays que nous voudrions vous poser.

19. Avant votre accouchement le plus récent, où et quand avez-vous accouché?

_____________________________ (pays), __________ (année)

_____________________________ (pays), __________ (année)

_____________________________ (pays), __________ (année)

_____________________________ (pays), __________ (année)

☐ S/O (aucune grossesse auparavant)

20. Quelle âge aviez-vous lorsque vous êtes venue au Canada pour y vivre? ____________(années)

21. Est-ce que quelqu'un a fait application pour vous aider à venir au Canada, et est-ce que cette personne était responsable de vous ici? (ex: est-ce que vous aviez un "parain")?

☐ Oui
☐ Non *(Veuillez passer à Q23)*

22. Si OUI, qui?
(Laissez répondre puis cocher une réponse)

☐ Mari
☐ Parent
☐ Enfant
☐ Organisation privée (ex: église, organization non-gouvernementale)
☐ Gouvernement
☐ Autre *(Veuillez préciser)* _________________________

23. Dans quel pays est né le père de votre bébé? _________________________(pays)

☐ Ne sais pas

24. Est-ce que le père du bébé habite avec vous?

☐ Oui
☐ Non

25. Est-ce que vous avez un lien de parenté sanguin avec le père de votre bébé?

☐ Oui
☐ Non

26. Si vous aviez un travail payant avant que le bébé naisse, quand aviez-vous arrêté le travail?

______________(mois)/______________ (année)

☐ Ne travaillait pas
☐ N'a pas arrêté de travailler.

27. **Si vous avez payé pour vos soins ou pour des services médicaux au CANADA durant votre dernière grossesse, votre dernier accouchement, ou après le dernier accouchement, quels sont les services pour lesquels vous avez payé, et combien avez-vous payé?**
(Lisez à voix haute puis cochez une réponse)

- ☐ Rendez-vous avec un professionnel de la santé $ ________
- ☐ Examen physique $ ________
- ☐ Tests sanguins $ ________
- ☐ Examen vaginal/frottis cervical $ ________
- ☐ Dépistage pour des anomalies à la naissance (ex: trisomy) $ ________
- ☐ Échographie $ ________
- ☐ Services de santé mentale $ ________
- ☐ Cours de grossesse/cours d'accouchement $ ________
- ☐ Medicament $ ________
- ☐ Services pour la naissance $ ________
- ☐ Autre *(Veuillez préciser)*_________________________ $ ________
- ☐ S/O

Nous avons 7 questions à propos de votre santé pendant votre grossesse que nous voudrions vous poser

28. **Lesquels de ces déclarations décrivent le mieux vos habitudes de consommation de tabac durant votre grossesse la plus récente.**
(Lisez à voix haute puis cochez une réponse)

- ☐ Vous n'avez pas fumé
- ☐ Vous fumiez parfois
- ☐ Vous fumiez du tabac chaque jour *(Veuillez préciser le nombre de fois que vous avez fumé dans la journée)* _________

29. **Combien pesiez-vous avant d'accoucher?**

_________(kg) _________(g)/__________(livres)_________(onces)

30. **Durant les derniers sept jours combien de fois avez-vous mange/bu un ou plusieurs des aliments dans la liste ci-dessous?**
(Lisez à voix haute et noter le nombre de fois)

Lentilles et haricots _______
Légumes à feuilles vertes foncées (ex: épinards) _______
Foie _______
Agrumes (ex: oranges) _______
Pain complet _______
Jus d'orange enrichi de vitamine D _______
Lait de vache _______

31. **Au moins un mois avant que vous tombiez enceinte, avez-vous pris un comprimé prénatal ou de l'acide folique chaque jour?**

- ☐ Oui *(Veuillez passer à Q33)*
- ☐ Non

32. **Si NON, pour quoi pas?**
(Laissez répondre et cochez toutes les réponses qui s'appliquent)

- ☐ Ne savais pas à quoi ça servait
- ☐ Ne pouvais pas le retrouver
- ☐ N'avais pas les moyens de l'acheter
- ☐ Pas disponible
- ☐ N'en avais pas besoin
- ☐ On ne m'a pas dit d'en prendre
- ☐ Autre *(Veuillez préciser)* ______________________
- ☐ S/O

---

33. **Durant votre grossesse, avez-vous pris un comprimé prénatal chaque jour?**

- ☐ Oui *(Veuillez passer à Q35)*
- ☐ Non

---

34. **Si NON, pour quoi pas?**
(Laissez répondre et cochez toutes les réponses qui s'appliquent)

- ☐ Ne savais pas à quoi ça servait
- ☐ Ne pouvais pas le retrouver
- ☐ N'avais pas les moyens de l'acheter
- ☐ Pas disponible
- ☐ N'en avais pas besoin
- ☐ On ne m'a pas dit d'en prendre
- ☐ Autre *(Veuillez préciser)* ______________________
- ☐ S/O

---

35. **Cela conclus notre entrevue. Y-a-t-il autre chose que vous voulez dire par rapport aux sujets que nous avons abordé? Ou encore, y-a-t-il autre chose que vous voulez ajouter?**
