# Supplementary material for: Migrant-friendly maternity care in Montreal, Canada: A cross-sectional study on migrant women’s care perspectives
Source: PLoS One. 2025 Aug 21;20(8):e0330830. doi: 10.1371/journal.pone.0330830 (PMC12370051; doi:10.1371/journal.pone.0330830)
Supplement: S13 Appendix — (PDF) [file pone.0330830.s013.pdf]

		अभ्यास आईडी नंबर:	
प्रारंभ का समय:		साक्षात्कारकर्ता (इंटरव्यूवर) का नाम:	
अंत का समय::		साक्षात्कार (इंटरव्यू) की तारीख:	

चंद प्रश्नावलियों में से एक प्रश्नावली को आप उत्तर कर चुके हैं इसके लिए हम आपके आभारी है। हम आपको इसके अतिरिक्त थोड़े और सवाल पूछना चाहेंगे जो पिछले प्रश्नावली में नहीं पूछे गए थे। प्रथम प्रश्नों का सेट गर्भवस्था से पहले होनेवाले आपके स्वास्थ्य के बारे में है।

1. **क्या आपको कोई रोग या बीमारी है जिस का कभी इलाज नहीं हुआ है?** (जैसे, मधुमेह- डायबिटिस, हृदय का रोग, दमा, जोड़ों का दर्द, मलेरिया, टीबी, एचआईवी, हेपेटाइटिस सी, कीड़े)

☐ हाँ *(कृपया स्पष्ट करें)* ____________________________

☐ नहीं *(प्रश्न नंबर 4 पर जाये)*

---

2. **क्या इन सभी बीमारियों का इलाज किया गया है?**

☐ हाँ, *सबका इलाज हुआ है*

☐ नहीं, कोई बीमारी का इलाज नहीं किया है, सिर्फ़ थोड़े बीमारियों का इलाज हुआ है

---

3. **क्या अपने आपके बीमारियों का इलाज करना कभी बंद किया है?**

☐ हाँ *(कृपया स्पष्ट करें)* ____________________________

☐ नहीं

---

4. **जब आप गर्भावति नहीं थे तब आपका सामान्य रुप से क्या वजन होता था?**

__________ (किलो) ________(ग्राम) __________ (पाउंड)__________(अउंस)

---

5. **आपकी ऊँचाई कितनी है?**

__________(फीट) __________(इंच) __________(मीटर)__________ (सेंटीमीटर)

---

6. **निम्मलिखित वक्यो में से आपके घर के बारे में कौनसा वाक्य सबसे अधिक लागू होता है?**

	हाँ	*नहीं*
मेरे साथ रहने वाले लोगों की संख्या में काफी बडा है	☐	☐
सर्दियो में काफी गर्म रहता है	☐	☐
घर में काफी शांति रहतीं है	☐	☐
घर मोल्ड और कीट से मुक्त है (जैसे कीड़े या चूहे)	☐	☐
घर के अंदर होने वाले धुएँ से घर मुक्त है (जैसे कि सिगरेट का धुँआ)	☐	☐
घर का बांधकाम सुरक्षित है (घर मजबूत है)	☐	☐
घर के अगल-बगल के पर्यावरण में धुँआ प्रदूषण की मात्रा बहुत कम या शून्य है	☐	☐
आपका घर एक सुरक्षित पड़ोस में है (यानी, कोई अप्राध नहीं होता)	☐	☐

7. आपका पोस्टल कोड़ आपके पड़ोस की जानकारी प्राप्त करने में हमें मदद करेगा | आपका पोस्टल कोड क्या है?

☐☐☐ ☐☐☐

आपके गर्भवती बनने के इरादों के बारे में हम आपसे 4 प्रश्न पूछना चाहेंगे|

8. जब आप इस बच्चे को लेकर गर्भवति हुए उस वक्त क्या आप गर्भवती होना चाहते थे?

- ☐ हाँ *(कृपया प्रश्न नंबर 12 पर जाएँ)*
- ☐ नहीं
- ☐ पता नहीं

9. यदि आप अनिश्चित थे या आप गर्भवति नहीं होना चहते थे, तो क्या आपने गर्भावस्था को टालने के लिए कोई योजना बनायी थी?

*(योजनाओं कि यदि देखने के लिए प्रश्न नंबर **10** के विकल्प पढ़ें)*

- ☐ हाँ
- ☐ नहीं *(कृपया प्रश्न नंबर 11 पर जाएँ)*

10. अगर आपका जवाब हाँ है तो आपने किस योजना का इस्तेमाल किया था?

(कृपया माँ को जवाब देने दें और सभी लागू जवाबों को टिक करें)

- ☐ कोंड्म
- ☐ स्तनपान
- ☐ गर्भनिरोधक गोली
- ☐ देपो-प्रोवेरा इंजेक्शन
- ☐ इंट्रायुटेराइन डिवाइस (आई.यू.डी)
- ☐ हर महीने आने वाले प्राकृतिक साइकिल कि स्व्यं निगरानी रखना
- ☐ स्वयं या साथी को बाँझ मानना
- ☐ निकासी ("पुल आउट")
- ☐ डायाफ्राम / ग्रीवा टोपी
- ☐ हाथ की त्वचा के अंदर (नोरप्लांट) सम्मिलित करना
- ☐ संयम रखना
- ☐ अन्य *(कृपया स्पष्ट करें)* ________________________
- ☐ आपको प्रश्न लागू नहीं करता

11.यदि आपने किस योजना का इस्तेमाल नहीं किया था तो हम इसका कारण जानना चाहेंगे |
(*कृपया माँ को जवाब देने दें और सभी लागू जवाबों को टिक करें*)

- ☐ क्लिनिक आपके पहुँच के बाहर था या स्वास्थ्य सेवा पेशेवर नहीं मौजूद थे
- ☐ साइड इफेक्ट
- ☐ आपके पास यह खरीदने के लिए पैसे नहीं थे
- ☐ धार्मिक कारणों से
- ☐ पति / परिवार उससे सहमत नहीं थे
- ☐ अन्य (*कृपया स्पष्ट करें*)______________________________
- ☐ आपको प्रश्न लागू नहीं करता

हम आपके दाँतों और मसूड़ों के स्वास्थ्य के बारे में 5 प्रश्न पूछना चाहेंगे |

12.कुल मिलाकर आपके के हिसाब से आपके दाँतों और मसूड़ों कि स्थिति कैसी है?
(*कृपया सारे विकल्प जोर से पढ़ें और किसी एक लगु उत्तर को टिक करें*)

- ☐ उत्कृष्ट
- ☐ बहुत अच्छा
- ☐ अच्छा
- ☐ ठीक ठाक
- ☐ खराब
- ☐ पता नहीं

13.क्या आपको लगता है कि आपको मसूड़ों का रोग है?

- ☐ हाँ
- ☐ नहीं
- ☐ पता नहीं

14.क्या आपने कभी मसूड़ों के लिए औशधि उपचार किए है जैसे रूट स्केलिंग, रूट प्लानिंग जिसे कभी कभी "दीप क्लिनिंग" भी कहा जता है?

- ☐ हाँ
- ☐ नहीं
- ☐ पता नहीं

15.क्या आपको कभी अपके दाँतों के डॉक्टर ने बताया है कि आप के दाँतों को मज़बूती देनेवाली हड्डी कम हो गयी है?

- ☐ हाँ
- ☐ नहीं
- ☐ पता नहीं

16.अपने दाँतों को ब्रश करने के अलावा, पिछले सात दिनों में, आपने कितनी बार दाँतों के बीच जमी सड़न को निकालने के लिये फ्लोस् किया है या किसी धातु का उपयोग किया है?

__________(बार)

☐ पता नहीं

कई देशों में लड़कियों के गुप्त हिस्से पर पारंपारिक कारणों से काट दिया जाता है (यानि फिमेल सरकमसिजन)। ईस लिए हम आपको *2* प्रश्न इन प्रथाओं के बारे में पूछना चाहेंगे।

17.क्या इस तरह कि प्रक्रिया आप पर हुई है?

☐ हाँ

☐ नहीं *(प्रश्न नंबर 19 पर जाएँ)*

18.यदि आपका जवाब हाँ है तो क्या ईस भाग को सिला कर बंद किया था?

☐ हाँ

☐ नहीं

☐ पता नहीं

नए देश में सेटल होने के बारे में हम आपसे 9 प्रश्न पूछना चाहेंगे।

19.आपके हाल ही में हुए गर्भावस्था से पहले आपने बच्चे को कब और कहाँ जन्म दिया था?

_________________________ (देश), _________ (साल)

_________________________ (देश), _________ (साल)

_________________________ (देश), _________ (साल)

_________________________ (देश), _________ (साल)

☐ आपको प्रश्न लागू नहीं करता *(आपका यह पहला गर्भवसथा से जुडा अनुभव था)*

20.आप कितने साल के थे जब आप कनाडा रहने आए? __________(साल)

21.कनाडा आने के लिए क्या आपके लिए किसी ने अपलाय (स्पॉन्सर) किया था (जैसे कोई व्यक्ति या संगठन जो आपके कनाडा आने के बाद आपके लिए जिम्मेदार थे?)

☐ हाँ

☐ नहीं *(प्रश्न नंबर 23 पर जाएँ)*

22. यदि आपका जवाब हाँ है तो वह कौन थे?

(कृपया माँ को जवाब देने दें और किसी एक लागू जवाब को टिक करें)

- ☐ पति
- ☐ माता पिता
- ☐ आपके बच्चे
- ☐ निजी (प्राइवेट) संगठन (जैसे चर्च या संगठन जो सरकारी नहीं है)
- ☐ सरकार
- ☐ *अन्य* ________________________*(कृपया स्पष्ट करें)*

---

23. आपके बच्चे के पिता का जन्म कौन से देश में हुआ था? ________________________ (देश)

- ☐ पता नहीं

---

24. क्या बच्चे के पिता आपके साथ रहते है?

- ☐ हाँ
- ☐ नहीं

---

25. कया बच्चे के पिताका और आपका खून से जुडा रिश्ता है?

- ☐ हाँ
- ☐ नहीं

---

26. *यदी आप बच्चे के जन्म से पहले काम पर जाते थे तो आप ने काम पर जाना कब बंद किया था?*

__________(महीना) __________(साल)

- ☐ आप काम नहीं करते थे
- ☐ आपने काम करना बंद नहीं किया था

---

27. यदि आप सबसे हाल ही में कनाडा में हुई मेडिकल (चिकित्सा) सेवाओं के लिए स्वयं पैसे भरते थे, तो आपको कितने पैसे जमा करने पड़ते थे?

☐ स्वास्थ्य देखभाल पेशेवर (जैसे डॉक्टर, नर्स, दाई) के साथ नियुक्ति (अपोइंटमेंट)	$
☐ शारीरिक परीक्षा	$
☐ रक्त परीक्षण	$
☐ सरवाइकल परीक्षा / पैप परीक्षण	$
☐ बच्चे के जन्म से जुड़े दोष के लिए स्क्रीनिंग (जैसे, डाउन सिंड्रोम)	$
☐ अल्ट्रासाउंड स्कैन	$
☐ मानसिक स्वास्थ्य सेवाएँ	$
☐ गर्भावस्था / प्रसव (लेबर) के बारे में जानकारी देनेवाली कक्षाएँ	$
☐ दवा	$
☐ बच्चे के जन्म के लिए उपलब्ध सेवाएँ	$
☐ अन्य________________ *(कृपया स्पष्ट करें)*	$
☐ स्वास्थ्य देखभाल पेशेवर के साथ नियुक्ति	$

गर्भवस्था के दौरान होनेवाली आपकी स्वास्थ्य के बारे में हम आपको *7* प्रश्न पूछना चाहेंगे

28. निम्मलिखित विकल्पों में से कौन से विकल्प में, आपकी गर्भवस्था के दौरान सिगरेट पीने की आदत का वर्णन किया गया है?

(सारे विकल्पों को जोर से पढ़ें और किसी एक लागू उत्तर को टिक करें)

- ☐ आप सिगरेट नही पीते थे
- ☐ आप कभी कभी सिगरेट पीते थे
- ☐ आप रोज़ सिगरेट पीते थे *(कृपया स्पष्ट करें के आप दिनके कितने सिगरेट पीते थे)*_______________

29. गर्भावस्था के अंत में बच्चे को जन्म देने से बिलकुल पहले आपका वजन क्या था?

_________ (किलो) __________ (ग्राम) __________(पाउंड) __________(आऊंस)

30. पिछले हफ्ते में कितने बार निम्मलिखित विकल्पो में दिए गए खाद्य पदार्थों को आपने खाया?

(*सारे विकल्पों को जोर से पढ़ें और कितनी बार खाया है उसकी संख्या नीचे लिखे*)

फलियाँ (जैसे बीन्स)	_______
हरे पत्तेवाली सब्जियाँ (जैसे पालक)	_______
कलेजा (लिवर)	_______
खट्टे फल (जैसे संतरा)	_______
पुरे अनाज (व्होले ग्रेन) की रोटी या ब्रेड (पाव)	_______
विटामिन 'डी' वाला संतरे का रस	_______
गाय का दूध	_______

31. गर्भवती होने से कम से कम एक महीने पहले क्या आपने प्रिनेटल विटामीन या फोलिक एसिड के सप्लिमेंट रोज़ लिए थे?

- ☐ हाँ *(प्रश्न नंबर 33 पर जाएँ)*
- ☐ नहीं

32 यदि आपका जवाब नहीं है तो हम जानना चाहेंगे कि क्यों *नहीं*

(कृपया माँ को जवाब देने दें और सभी लागू जवाबों को टिक करें)

- ☐ यहा क्या चीजें थी आपको पता नहीं था
- ☐ यह आपको मिल नहीं रहा था
- ☐ इसे खरीदने के लिए आपके पास पैसे नहीं थे
- ☐ उपलब्ध नहीं था
- ☐ आपको इसकी जरूरत नहीं थी
- ☐ इसे लेने के लिए कहा नही गया था
- ☐ अन्य *(कृपया स्पष्ट करें)* _____________________
- ☐ आपको जवाब लागू नहीं करता

33 **<u>गर्भवस्था के दौरान</u> क्या आप प्रिनेटल विटामीन रोज़ लेते थे?**

- ☐ हाँ *(प्रश्न नंबर 35 पर जाएँ)*
- ☐ नहीं

---

34. यदि आपका जवाब नहीं है तो हम जानना चाहेंगे कि क्यों
(*कृपया माँ को जवाब देने दें और सभी लागू जवाबों को टिक करें***)**

- ☐ यह क्या चीजें है आपको पता नहीं था
- ☐ यह आपको मिल नहीं रहा था
- ☐ इसे खरीदने के लिए आपके पास पैसे नहीं थे
- ☐ उपलब्ध नहीं था
- ☐ आपको इसकी जरूरत नहीं थी
- ☐ इसे लेने के लिए कहा नही गया था
- ☐ अन्य *(कृपया स्पष्ट करें)* ____________________
- ☐ आपको जवाब लागू नहीं करता

---

35. इस से हमारा यह साक्षात्कार (इंटरव्यू) समाप्त होता है | इस साक्षात्कार में शामिल किये गए विषयों के अलावा यदि आप हमें कुछ और बातें कहना या बताना चाहते है, तो हमें जरुर बताइये |
