# Supplementary material for: Migrant-friendly maternity care in Montreal, Canada: A cross-sectional study on migrant women’s care perspectives
Source: PLoS One. 2025 Aug 21;20(8):e0330830. doi: 10.1371/journal.pone.0330830 (PMC12370051; doi:10.1371/journal.pone.0330830)
Supplement: S14 Appendix — (PDF) [file pone.0330830.s014.pdf]

MFMC 蒙特利尔附加问卷 – 中文版		参与人编码:	
开始时间:		采访人名:	
结束时间:		采访日期:	

非常感谢您参与我们第一轮的调查问卷。为了补充上次调查, 请回答:

第一部分: 您怀孕前的状况

1. 请问您有否曾患有疾病？

(例如：*糖尿病, 心脏病, 哮喘, 关节炎, 疟疾, 结核病, 艾滋病, 丙肝, 肠胃寄生虫*)

☐ 有 (*请说明*) ________________________

☐ 没有 (*请跳到第 4 题*)

2. 请问您有没有针对您所有的状况接受治疗?

☐ 有，所有的病症都有得到治疗 ________________________

☐ 没有, 或者只有一些有得到治疗

3. 请问您有没有停止任何针对您病症的治疗？

☐ 有 （*请说明*） ________________________

☐ 没有

4. 请问您还未怀孕时,您的体重一般为多少?

________千克 ________克 / ________磅 ________盎司

5. 请问您身高多少?

________ 英尺 ________ 英寸 / ________ 米 _______ 分米

6. 请问以下哪些陈述能 形容您的家居状况？

	是	否
房间足够容纳和您同住的人	☐	☐
冬天里房屋足够温暖	☐	☐
周围的环境足够安静	☐	☐
居住的地方无霉无害虫（例如：蟑螂或老鼠）	☐	☐
室内无烟气 （例如：炒菜油烟）	☐	☐
住家的结构安全 (例如：房屋结构结实)	☐	☐
社区周围无空气污染	☐	☐
居住在一个安全社区(无犯罪活动)	☐	☐

7. 为了全面地了解您的社区, 请问您居住地的邮政编码是什么?

☐☐☐ ☐☐☐

第二部分：以下是 4 道关于您怀孕计划的问题

8. 请问在您怀上这个孩子之前, 您愿意怀孕吗?

- ☐ 是 *(请跳到第 12 题)*
- ☐ 不是
- ☐ 不确定

9. 请问您若不确定或不想怀孕的时候, 您有使用任何避孕方式吗?
（若需要, 请参考第 10 题 ）

- ☐ 有
- ☐ 没有 *(请跳到第 11 题)*

10. 若选 "有", 请问您用了什么方式？
(请让参与人独立回答并选择所有适合的答案，然后跳到第 12 题)

- ☐ 避孕套
- ☐ 母乳喂奶
- ☐ 避孕药
- ☐ 孕酮注射液
- ☐ 宫内节育器
- ☐ 自然经期观察（安全期）
- ☐ 认为有一方是不孕的
- ☐ 体外射精
- ☐ 隔膜/宫颈帽
- ☐ 皮下埋植 （ Norplant ）
- ☐ 节制
- ☐ 其他 *(请说明)*- ________________________
- ☐ 此题与我无关

11. 若您没有使用避孕方式，请问为什么？

(请让参与人独立回答并选择所有适合的答案)

- ☐ 没办法医生
- ☐ 副作用
- ☐ 没有足够经济资源
- ☐ 信仰原因
- ☐ 丈夫/家庭人员不支持
- ☐ 其他 *(请说明)* ________________________________
- ☐ 此题与我无关

*第三部分：以下是 5 道关于*您口腔健康的*问题*

12. 总体来讲，您认为您的口腔健康如何？

(请读出问题并选一项最适合的答案)

- ☐ 非常好
- ☐ 很好
- ☐ 好
- ☐ 不怎么样
- ☐ 不好
- ☐ 不知道

13. 请问您认为您有牙龈问题吗？

- ☐ 有
- ☐ 没有
- ☐ 不知道

14. 请问您有没有做过任何牙龈治疗？ (例如: 清洗牙根)

- ☐ 有
- ☐ 没有
- ☐ 不知道

15. 请问有没有牙医曾经说您有失去牙周的骨骼？

- ☐ 有
- ☐ 没有
- ☐ 不知道

16. 请问除了有牙刷刷牙以外，您上星期有用多少次牙线？

___________**(次)**

☐ 不知道

第四部分：在一些少数国家，*年轻女性会因为传统原因*除去一部分自己的*隐私部位*

以下的是两道关于这项习俗的问题

17. 请问您有经历过这类行为吗？

☐ 有

☐ 没有 *(请跳到第 19 题)*

18. 若选 "有", 请问被切部位有被缝好吗？

☐ 有

☐ 没有

☐ 不知道

第五部分：以下是 9 道关于您定居到新国家的问题

19. 请问在您最近期怀孕之前，您是在什么时候/地方分娩的呢？

____________ (国家), __________ (年)

____________ (国家), __________ (年)

____________ (国家), __________ (年)

____________ (国家), __________ (年)

☐ 此题与我无关 (无怀孕历史)

20. 请问搬来加拿大的时候，您多大? ____________**(年)**

21. 请问是否有他人帮您申请来加拿大？ (例如：赞助)

☐ 是

☐ 不是 *(请跳到第 23 题))*

22. 若选 “是”, 请问是谁？

(请让参与人独立回答并选择一项适合的答案)

☐ 丈夫

☐ 父母

☐ 孩子

☐ 独立组织 (例如：教堂，非政府组织)

☐ 政府

☐ 其他 （*请说明*）) ________________________

---

23. 请问孩子的父亲是在哪个国家出生的？________________________(国家)

☐ 不知道

---

24. 请问孩子的父亲与您同居吗？

☐ 是

☐ 不是

---

25. 请问孩子的父亲与您有血缘关系吗？

☐ 有

☐ 没有

---

26. 若您怀孕前有份有薪工作，请问您什么时候停止工作的?

__________ **月**__________ **年**

☐ 没有工作

☐ 没有停止工作

---

27. 若您在怀孕，分娩以及产后期间有在加拿大为医疗服务付费，是为了哪些服务并支付了多少？

☐ 预约	$
☐ 体检	$
☐ 化验	$
☐ 宫颈测试/PAP 测试	$
☐ 出生缺陷筛查（例如：唐筛）	$
☐ 超声波	$
☐ 心理健康检验	$
☐ 怀孕/亲子课程	$
☐ 药物	$
☐ 分娩费用	$
☐ 其他 *(请说明)* ________________________	$
☐ 此题与我无关	

第六部分：以下是 7 道关于您怀孕健康的问题

28. 请问以下哪一项最好地形容您最近一次怀孕期的抽烟习惯？
(请读出问题并选一项最适合的答案)

☐ 我没有吸烟

☐ 我偶尔吸烟

☐ 我每天吸烟 *(请说明每天抽多少)* _________

---

29. 请问您分娩前的体重是多少？

_________千克 / _________克 / _________磅 / _________盎司

---

30. 请问上星期您有食用多少次以下列出的食物？
(请读出选项并记录次数)

豆类（例如：大豆） _______
深绿色带叶菜（例如：菠菜） _______
肝 _______
柑橘类 (例如：橙子) _______
全麦面包 _______
豆类（例如：大豆维他命 D 橙汁 _______
牛奶 _______

---

31. 请问在您在怀孕的至少一个月以前，您有每天补充孕妇维他命或者叶酸吗？

☐ 有 *(请跳到第 33 题)*

☐ 没有

---

32. 若选 “没有”, 请问是因为什么？
(请让参与人独立回答并选一项最适合的答案)

☐ 不知道为什么需要补充营养

☐ 找不到

☐ 没有足够的资金去买

☐ 没有可用的 ？

☐ 不需要补充

☐ 没人说要补充

☐ 其他 *(请说明)* ______________________

☐ 此题与我无关

---

33. 请问在怀孕期，您有每天补充孕妇维他命吗？

- ☐ 有 *(请跳到第 35 题)*
- ☐ 没有

34. 若选 "没有", 请问是因为什么？
(请让参与人独立回答并选一项最适合的答案)

- ☐ 不知道为什么需要补充营养
- ☐ 找不到
- ☐ 没有足够的资金去买
- ☐ 没有可用的 ？
- ☐ 不需要补充
- ☐ 没人说要补充
- ☐ 其他 *(请说明)* ______________________
- ☐ 此题与我无关

35. 以上是全部采访内容。请问您对我们所概括的有什么建议或者需要注释的吗？您认为还有什么你想添加的吗？
