# Supplementary material for: Migrant-friendly maternity care in Montreal, Canada: A cross-sectional study on migrant women’s care perspectives
Source: PLoS One. 2025 Aug 21;20(8):e0330830. doi: 10.1371/journal.pone.0330830 (PMC12370051; doi:10.1371/journal.pone.0330830)
Supplement: S15 Appendix — (PDF) [file pone.0330830.s015.pdf]

MFMC Montreal Supplemental Questions - Spanish Version		ID del estudio:	
HORA DE INICIO:		Nombre del entrevistador:	
HORA DE CONCLUSION:		Fecha de la entrevista:	

Le agradecemos haber respondido nuestro cuestionario. Tenemos algunas preguntas adicionales sobre temas que no fueron cubiertos en dicho cuestionario. La primera serie de preguntas es sobre su salud en general antes del embarazo.

1. ¿Tiene alguna condición o enfermedad?
(ej., diabetes, enfermedades del corazón, asma, artritis, malaria, tuberculosis, VIH, hepatitis C, parasitos)

- ☐ Sí *(por favor especifique)* ___________________________
- ☐ No *(Pasa a la P4)*

2. ¿Ha recibido tratamiento para todas estas?

- ☐ Sí, todas fueron tratadas
- ☐ No, ninguna o solo algunas fueron tratadas

3. ¿Alguna vez ha parado tratamientos o cuidados que recibía para alguna condición que tenía?

- ☐ Sí *(por favor especifique)* ___________________________
- ☐ No

4. ¿Cuál es su peso normal (cuándo no está embarazada)?

_________(kg)_____(g) /__________(libras)______(onzas)

5. ¿Cuál es su estatura?

_______ (pies)______(pulgadas)/ ________ (m)_______(cm)

6. Cuáles de las siguientes afirmaciones son ciertas sobre su hogar

	Sí	*No*
Es suficientemente grande para las personas que viven conmigo	☐	☐
Es suficientemente caliente en invierno	☐	☐
Es suficientemente silencioso	☐	☐
Está libre de moho y plagas (ej. insectos o ratas)	☐	☐
Está libre de humo al interior (incluyendo el humo de cigarrillos)	☐	☐
Es estructuralmente fuerte (es decir el inmueble es estable)	☐	☐
Está en un vecindario sin o con poca contaminación	☐	☐
Está en un vecindario seguro (es decir no hay crimen)	☐	☐

7. Su código postal nos ayuda a saber más sobre su vecindario, ¿cuál es su código postal?

☐☐☐ ☐☐☐

Tenemos 4 preguntas sobre la planificación del embarazo que nos gustaría preguntarle.

8. **¿Cuándo se embarazo de este bebé, usted quería embarazarse en ese momento?**

- ☐ Sí *(Pasa a la P12)*
- ☐ No
- ☐ No estoy segura

9. **Si no está segura o no quería embarazarse, ¿hacía algo para prevenir el embarazo?**
(Usa la lista en P10 como ejemplos si lo es necesario)

- ☐ Sí
- ☐ No *(Pasa a la P11)*

10. **Si la respuesta es Sí, ¿que usó?**
(Deja que la madre responda y marca las casillas correspondientes, luego pasa a la P12)

- ☐ Condón
- ☐ Amamantar
- ☐ Pastillas anticonceptivas (mini píldora)
- ☐ Inyección anticonceptiva (Depo-provera)
- ☐ Dispositivo intrauterino(DIU)
- ☐ Observación del ciclo menstrual
- ☐ Ser o pareja pensó ser estéril
- ☐ Coitus Interruptus (eyaculación al exterior de la vagina)
- ☐ Diafragma / capuchón cervical
- ☐ Capsula insertada debajo de la piel del brazo (Implante Norplant)
- ☐ Abstinencia
- ☐ Otro *(por favor especifique)* ___________________________
- ☐ N/A

11. **Si no uso ningún método anticonceptivo, ¿por qué no?**
(Deja que la madre responda y marca las casillas correspondientes)

- ☐ No tuvo acceso a una clínica o proveedor de salud
- ☐ Efectos secundarios
- ☐ No podía permitírselo (económicamente)
- ☐ Razones religiosas
- ☐ El esposo o la familia no se lo permite
- ☐ Otro *(por favor especifique)* ___________________________________
- ☐ N/A

Tenemos 5 preguntas sobre la salud de sus encías y dientes que nos gustaría preguntarle

12. En general, ¿cómo califacaría que es el estado de la salud de sus dientes y encías?
(Lee en voz alta y marca la casilla correspondiente)

- ☐ Excelente
- ☐ Muy bien
- ☐ Bien
- ☐ Aceptable
- ☐ Pobre
- ☐ No sé

13. ¿Piensa usted que tal vez sufra de enfermedades de las encías?

- ☐ Sí
- ☐ No
- ☐ No sé

14. ¿Alguna vez ha tenido tratamiento para enfermedades de las encías de tipo raspado o alisado de las raíces, que a veces se conoce como "limpieza profunda"?

- ☐ Sí
- ☐ No
- ☐ No sé

15. ¿Alguna vez le ha dicho un profesional de la salud dental que usted ha perdido hueso alrededor de los dientes?

- ☐ Sí
- ☐ No
- ☐ No sé

16. Aparte del cepillado de sus dientes, ¿cuántas veces ha usado la seda/hilo dental o algún otro medio o utensilio para limpiarse entre los dientes en los últimos siete días?

__________(Cuantas veces)

- ☐ No sé

En algunos países, hay una práctica en la cual una niña podría tener una parte de su área privada cortada por razones tradicionales (es decir circuncisión femenina). Nos gustaría hacerle 2 preguntas sobre esta práctica.

17. ¿Le han hecho esto a usted?

- ☐ Sí
- ☐ No *(Pasa a la P19)*

18. Si la respuesta es Sí, ¿el área fue cerrada por costura?

- ☐ Sí
- ☐ No
- ☐ No sé

Tenemos 9 preguntas sobre la mudanza a un nuevo país que nos gustaría preguntarle

19. Antes de su parto más reciente, cuándo y donde dio a luz?

____________________ (país), __________ (año)
____________________ (país), __________ (año)
____________ ________(país), __________ (año)
____________________ (país), __________ (año)

☐ N/A (no hubo nacimientos previos)

20. ¿Qué edad tenía cuándo vino a Canadá para quedarse? ____________(años)

21. ¿Alguien aplicó para que usted veniera a Canadá, y que fue responsable por usted aquí (es decir, que la "apadrinó")?

☐ Sí
☐ No *(Pasa a las P23)*

22. Si la respuesta es Sí, quién fue?
(Deja que la madre responda y marca la casilla correspondiente)

☐ Esposo
☐ Padre/madre
☐ Hijo(a)
☐ Organización privada (ej., iglesia, organización no gubernamental)
☐ Gobierno
☐ Otro *(por favor especifique)* _________________________

23. ¿En qué país nació el padre de su bebé? _________________________(país)

☐ No sé

24. ¿El padre de su bebé vive con usted?

☐ Sí
☐ No

25. ¿El padre de su bebé está relacionado por sangre a usted?

☐ Yes
☐ No

26. ¿Si tuvo un trabajo pagado antes de que su bebé naciese, cuándo dejo de trabajar?
______________(mes)/___________(año)

☐ No trabajaba
☐ No dejo de trabajar

27. **¿Si pagó por su cuidado o servicios médicos en CANADÁ durante el embarazo, nacimiento y después del nacimiento, para qué servicios pagó y cuánto pagó?**
(Lee en voz alta y marca las casillas correspondientes)

☐ Cita con un profesional de la salud	$ ________
☐ Examen físico	$ ________
☐ Examen de sangre	$ ________
☐ Examen cervical o la prueba del Papanicolau	$ ________
☐ Pruebas para la detección de defectos congenitos (ej., síndrome de Down)	$ ________
☐ Ecografía	$ ________
☐ Servicios de salud mental	$ ________
☐ Clases prenatales	$ ________
☐ Medicación	$ ________
☐ Servicios durante el nacimiento	$ ________
☐ Otro *(por favor especifique)*___________________	$ ________
☐ N/A	

Tenemos 7 preguntas sobre salud durante el embarazo que nos gustaría preguntarle

28. **¿Cuál de los siguientes describe mejor sus hábitos con respecto al fumar tabaco durante su embarazo más reciente?**
(Lee en voz alta y marca la casilla correspondiente)

☐ No fumaba
☐ Fumaba de vez en cuando
☐ Fumaba todos los días *(por favor especifique cuántas veces al día fumaba)* _________

29. **¿Cuál era su peso al final de su embarazo?**

_________(kg)_____(g) /__________(libras)______(onzas)

30. **¿Cuántas veces en la semana pasada, comió o bebió alguna de las siguientes comidas o bebidas?**
(Lee en voz alta y escribe el número de veces)

Lentejas y frijoles	_______
Verduras de hoja verde oscura (ej., espinaca)	_______
Hígado	_______
Frutas cítricas (ej., naranjas)	_______
Pan integral	_______
Jugo de naranja fortificado con vitamina D	_______
Leche de vaca	_______

31. **Al menos un mes antes de que se embarazara, ¿tomó vitaminas prenatales o ácido fólico como suplemento diariamente?**

☐ Sí *(Pasa a la P33)*
☐ No

32. **Si la respuesta es no, ¿por qué no?**
(Deja que la madre responda y marca las casillas correspondientes)

- ☐ No sabía para qué era(n)
- ☐ No podía encontrarlo(s)
- ☐ No tenía los recursos para comprarlo(s)
- ☐ No estaba(n) disponible
- ☐ No lo(s) necesitaba
- ☐ Le dijeron que no lo(s) tomará
- ☐ Otro *(por favor especifique)* ______________________
- ☐ N/A

---

33. **¿Durante su embarazo, tomó vitaminas prenatales diariamente?**

- ☐ Sí *(Pasa a la P35)*
- ☐ No

---

34. **Si la respuesta es No, ¿por qué no?**
(Deja que la madre responda y marca las casillas correspondientes)

- ☐ No sabía para qué era(n)
- ☐ No podía encontrarlo(s)
- ☐ No tenía los recursos para comprarlo(s)
- ☐ No estaba(n) disponible
- ☐ No lo(s) necesitaba
- ☐ Le dijeron que no lo tomará
- ☐ Otro *(por favor especifique)* ______________________
- ☐ N/A

---

35. **Terminamos nuestra entrevista. ¿Hay alguna otra cosa que quisiera decir sobre los temas que hemos cubierto? ¿O alguna cosa más que quisiera añadir?**
