# Supplementary material for: Migrant-friendly maternity care in Montreal, Canada: A cross-sectional study on migrant women’s care perspectives
Source: PLoS One. 2025 Aug 21;20(8):e0330830. doi: 10.1371/journal.pone.0330830 (PMC12370051; doi:10.1371/journal.pone.0330830)
Supplement: S16 Appendix — (PDF) [file pone.0330830.s016.pdf]

**MFMC Montreal Supplemental Questions - Urdu Version**		شناختی نمبر	
شروع کرنے کا وقت		انٹرویو لینے والے کا نام	
ختم کرنے کا وقت		انٹرویو کی تاریخ	

ہم آپکے شکر گزر ہیں کہ آپ نے ہمارا ایک سوالنامہ حل کیا۔ ہمارے پاس کچھ اضافی سوال ہیں جو پہلے سوالنامے میں نہیں پوچھے گئے ۔ پہلے چند سوال حمل سے پہلے آپکی مجموعی صحت پر ہیں۔

1. **کیا آپ کو کوئی بیماری ہے ؟** (مثلا ذیابطیس، دل کی بیماری، دمہ، جوڑوں کا درد، ملیریا، ٹی بی، HIV، ہیپاٹیٹس C، پیٹ کے کیڑے)

☐ جی ہاں (برائے مہربانی وضاحت کیجئے) ______________________________

☐ نہیں (براے مہربانی سوال نمبر 4 پر جائیے)

2. **کیا ان کا کبھی کوئی علاج کیا گیا ہے؟**

☐ جی ہاں (برائے مہربانی وضاحت کیجئے) ______________________________

☐ نہیں

3. **کیا آپ نے کبھی اپنی کسی بیماری کا علاج یا نگہداشت روکی؟**

☐ جی ہاں (برائے مہربانی وضاحت کیجئے) ______________________________

☐ نہیں

4. **عام طور پر آپکا وزن کتنا ہوتا ہے؟ (جب آپ حاملہ نہیں ہوتیں)**

__________ کلو ________________ گرام - __________ پاؤنڈ ______ اونس

5. **آپکا قد کتنا ہے ؟**

_______ فٹ ____________ انچ- _____________ میٹر ________ سینٹمیٹر

6. **آپکے گھر کے بارے میں مندرجہ ذیل میں سے کونسے بیانات درست ہیں؟**

	جی ہاں	*نہیں*
اس میں یہاں رہنے والے تمام افراد کی جگہ ہے	☐	☐
سردیوں میں یہ گرم رہتا ہے<br>اس میں بہت شور سنائی نہیں دیتا	☐	☐
یہ کائی، کیڑے مکوڑوں اور چوہوں وغیرہ سے پاک ہے	☐	☐
اس کے اندر کسی دھویں مثلا سگریٹ وغیرہ کی بدبو نہیں ہے	☐	☐
یہ مضبوط بنا ہوا ہے	☐	☐
یہ ایسے محلے میں ہے جو فضائی آلودگی سے پاک ہے	☐	☐
یہ ایک محفوظ علاقے میں ہے (یعنی جرائم وغیرہ سے پاک)	☐	☐

7. **آپکا پوسٹل کوڈ ہمیں آپکے علاقے کے بارے میں مزید معلومات فراہم کرتا ہے ۔ آپکا پوسٹل کوڈ کیا ہے؟**

☐☐☐ ☐☐☐

بچہ پیدا کرنے کے ارادے کے بارے میں ہم آپ سے 4 سوال پوچھنا چاہتے ہیں

8. **جب آپ اس بچے کے ساتھ امید سے ہوئیں تو آپ حاملہ ہونا چاہتی تھیں؟**

☐ جی (سوال نمبر 12 پر جایئں)
☐ نہیں
☐ کچھ کہہ نہیں سکتی

---

9. **اگر آپ یہ بچہ پیدا نہیں کرنا چاہتی تھیں یا ابھی ارادہ نہیں کیا تھا تو کیا آپ نے خاندانی منصوبہ بندی کا کوئی انتظام کیا تھا؟**
(اگر ضرورت ہو تو سوال نمبر 10 سے مثالیں دیکھ لیں)

☐ جی
☐ نہیں (سوال نمبر 11 پر جایئں)

---

10. **اگر ہاں تو آپ نے کیا استعمال کیا ؟**
(ماں کے جوابات کے مطابق نشان لگائیں پھر سوال نمبر 12 پر جائیں)

☐ کونڈم، (غبارہ)
☐ ماں کا دودھ پلانا
☐ دوائی
☐ Depo-Provera کا ٹیکا
☐ رحم کے اندر کائل (IUD)
☐ ماہواری کی تاریخوں کا خیال رکھنا
☐ یہ خیال تھا کہ آپ خود یا آپکا ساتھی بانچھ ہے (بچے بند کرانے کا آپریشن کرایا)
☐ بیرونی انزل
☐ ڈایافرام / رحم کے منہ پر جھلی، خواتین کیلئے غبارہ
☐ بازو کی جلد کے نیچے کچھ رکھوایا (Norplant)
☐ پرہیز
☐ کوئی اور (برائے مہربانی وضاحت کیجئے) ____________________
☐ لاگو نہیں ہوتا N/A

---

11. **اگر آپ نے خاندانی منصوبہ بندی کا کوئی طریقہ استعمال نہیں کیا تو اس کی کیا وجہ تھی؟**
(ماں کے جواب کے مطابق نشان لگائیں)

☐ کسی ماہر صحت تک رسائی نہیں تھی
☐ منفی اثرات
☐ اس کے پیسے نہیں تھے
☐ دینی وجوہات
☐ شوہر یا گھر والوں نے اجازت نہیں دی
☐ کوئی اور (برائے مہربانی وضاحت کیجئے) ____________________
☐ لاگو نہیں ہوتا N/A

آپ کے دانتوں اور مسوڑھوں کی صحت کے بارے میں 5 ہم آپ سے پوچھنا چاہتے ہیں.

12. مجموعی طور پر آپ اپنے دانتوں اور مسوڑھوں کی صحت کو کیسا سمجھتی ہیں؟
(اونچا پڑھ کر سنائیں اور کسی ایک پر نشان لگائیں)

☐ بہترین
☐ بہت اچھی
☐ اچھی
☐ بس ٹھیک
☐ خراب
☐ معلوم نہیں

13. کیا آپ کے خیال میں آپ کو مسوڑھوں کی کوئی بیماری ہے؟

☐ جی ہاں
☐ نہیں
☐ معلوم نہیں

14. آپ نے کبھی اپنے مسوڑھوں کا کوئی علاج کرایا مثلا دانت کی جڑ پر سے سخت چیزکھرچوانا (یعنی سکیلنگ، یا جڑ کا کوئی انتظام جسے تہ تک صفائی بھی کہتے ہیں)

☐ جی ہاں
☐ نہیں
☐ معلوم نہیں

15. آپ کو کبھی کسی دانتوں کے ڈاکٹر نے یہ کہا کہ آپکی دانت کے پاس سے آپ کی ہڈی گھل رہی ہے؟

☐ جی ہاں
☐ نہیں
☐ معلوم نہیں

16. برش سے دانت صاف کرنے کے علاوہ آپ نے پچھلے 7 دنوں میں کتنی دفعہ دھاگے سے فلاس کیا یا دانتوں کے درمیان کسی اور طرح سے صفائی کی؟

__________ دفعہ

☐ معلوم نہیں

بعض ممالک میں بچیوں کی جاے مخصوصہ کو کاٹا جاتا ہے (جیسے ختنے) ہم اس کے بارے میں آپ سے 2 سوال پوچھنا چاہیں گے.

17. کیا آپ کے ساتھ ایسا ہوا؟

☐ جی ہاں
☐ نہیں (سوال نمبر ۱۹ پر جایں)

18. اگر ہاں تو کیا یہ حصّہ سیا گیا تھا؟

☐ جی ہاں
☐ نہیں
☐ معلوم نہیں

نیے ملک میں آ کر رہنے کے بارے میں ۹ سوال ہیں جو ہم آپ سے پوچھنا چاہتے ہیں

19. اپنے حالیہ بچے کی پیدائش سے پہلے آپ نے کب اور کہاں بچے/ بچوں کو جنم دیا ؟

(ملک) ___________ ، (سال) __________
(ملک) ___________ ، (سال) __________
(ملک) ___________ ، (سال) __________
(ملک) ___________ ، (سال) __________

☐ لاگو نہیں ہوتا N/A (اس سے پہلے کوئی اور بچے نہیں تھے)

---

20. جب آپ کینیڈا رہنے کے لیے ییں تو آپ کتنے سال کی تھیں ؟ ______________ (سال)

---

21. کیا آپ کے کینیڈا آنے کے لیے کسی اور نے آپکی امیگریشن کی درخواست بھری کیا تھا جو آپ کی یہاں ذمہ داری اٹھاتا/اٹھاتی ہیں؟ (یعنی آپکو سپانسر کیا)

☐ جی
☐ نہیں (سوال نمبر 23پر جایئں)

---

22. اگر ہاں تو وہ کون ہے؟
(ماں کے جواب کے مطابق کسی ایک پر نشان لگائیں)

☐ شوہر
☐ والدین
☐ بچے
☐ کوئی نجی ادارہ (مثلا دینی ادارہ یا NGO وغیرہ)
☐ گورمنٹ
☐ کوئی اور (برائے مہربانی وضاحت کیجئے)______________________

---

23. اس بچے کے والد کس ملک میں پیدا ہوے؟ _________________ (ملک)

☐ معلوم نہیں

---

24. اس بچے کے والد آپ کے ساتھ رہ رہے ہیں؟

☐ جی ہاں
☐ نہیں

---

25. اس بچے کے والد کا کیا آپ سے کوئی خوں کا رشتہ ہے؟

☐ جی ہاں
☐ نہیں

---

26. اگر بچے کی پیدائش سے پہلے آپکی کوئی آمدنی والی نوکری تھی تو آپ نے کب کام کرنا بند کیا؟ ______________ (ماہ)/ _______ (سال)
(اندازا کونسے ماہ/ سال)

☐ کام نہیں کرتی تھیں
☐ کام کرنا بند نہیں کیا

27. **اگر آپ نے اپنے حمل ، بچے کی پیدائش سے پہلے یا بعد میں کسی طبّی سہولت یا دیکھ بھال کے لیے پیسے دئیے تو کس کے لیے دئیے اور کتنے؟**

☐ ماہر صحت سے اپائنٹمنٹ	$ ________
☐ جسمانی معائنہ	$ ________
☐ خون ٹیسٹ	$ ________
☐ بچہ دانی کے منہ کا معائنہ/ PAP ٹیسٹ	$ ________
☐ کسی پیدائشی بیماری کی نشاندہی (مثلاً DOWN SYNDROME)	$ ________
☐ الٹراساؤنڈ	$ ________
☐ ذہنی صحت کی سہولت	$ ________
☐ حمل/ بچے کی پیدائش کے متعلق کلاسیں	$ ________
☐ دوائیاں	$ ________
☐ پیدائش کے وقت کی سہولیات	$ ________
☐ کوئی اور (براے مہربانی وضاحت کیجئے) ________	$ ________
☐ لاگو نہیں ہوتا N/A	

حمل کے دوران آپکی صحت کے بارے میں 7 سوال آپ سے پوچھیں گے

28. **مندرجہ ذیل میں سے کونسا جملہ آپکے حالیہ حمل کے دوران آپ کی تمباکو / سگریٹ نوشی کی عادت کو بہتر طور پر بیان کرتا ہے؟**

☐ آپ سگریٹ نہیں پیتی ہیں
☐ آپ کبھی کبھار سگریٹ پیتی ہیں
☐ آپ روزانہ سگریٹ/ حقہ پیتی ہیں (براے مہربانی بتائے کہ کتنی دفع) ________

29. **حمل کے مکمّل ہونے پر آپکا وزن کتنا تھا؟**

________کلو ________ گرام / ________ پاؤنڈ ________ اونس

30. **پچھلے ہفتے میں کتنی مرتبہ آپ نے ان میں سے کوئی چیز کھائی یا پی ہے ؟**
(اونچا پڑھ کر سنائیں اور تعداد لکھیں)

دالیں (مثلاً لوبیا وغیرہ)	________
گہرے سبز پتوں والی سبزی (مثلاً پالک وغیرہ)	________
کلیجی	________
مالٹا، کینووغیرہ	________
چھان والی روٹی	________
وٹامن D ملا اورینج جوس	________
گائے کا دودھ	________

31. **حاملہ ہونے سے کم از کم ایک ماہ پہلے کیا آپ نے اپنے حمل کے لیے وٹامن یا فولاد (فولک ایسڈ) کی کوئی دوائی لی؟**

☐ جی ہاں (سوال نمبر33پر جائیے)
☐ نہیں

.32 **اگر نہیں تو کیوں نہیں؟**
(ماں کے جوابات کے مطابق نشان لگائیں)

- ☐ معلوم نہیں تھا کہ یہ کس لیے ہے؟
- ☐ ڈھونڈ نہیں سکی
- ☐ اس کو خریدنے کے پیسے نہیں تھے
- ☐ میسر نہیں تھی
- ☐ اس کی ضرورت نہیں تھی
- ☐ اس کو لینے کا کہا نہیں گیا
- ☐ کوئی اور (براے مہربانی وضاحت کیجئے)________________________
- ☐ لاگو نہیں ہوتا N/A

.33 **اپنے <u>حمل کے دوران</u> کیا آپ نے کوئی حمل کی وٹامن روزانہ لی؟**

- ☐جی ہاں (سوال نمبر 35 پر جائیں)
- ☐ نہیں

.34 **اگر نہیں تو کیوں نہیں؟**
(ماں کے جواب کے مطابق نشان لگائیں)

- ☐ معلوم نہیں تھا کہ یہ کس لیے ہے؟
- ☐ ڈھونڈ نہیں سکی
- ☐ اس کے پیسے نہیں تھے
- ☐ میسر نہیں تھی
- ☐ اس کی ضرورت نہیں تھی
- ☐ اس کو لینے کا کہا نہیں گیا
- ☐ کوئی اور (براے مہربانی وضاحت کیجئے)________________________
- ☐ لاگو نہیں ہوتا N/A

.35 **ہمارا انٹرویو یہاں ختم ہوتا ہے. ہم نے جن موضوعات پر بات کی ہے آپ ان مپر کچھ اور کہنا چاہیں گی؟ یا کسی بات کا اضافہ کرنا چاہیں گی؟**
