# Supplementary material for: Migrant-friendly maternity care in Montreal, Canada: A cross-sectional study on migrant women’s care perspectives
Source: PLoS One. 2025 Aug 21;20(8):e0330830. doi: 10.1371/journal.pone.0330830 (PMC12370051; doi:10.1371/journal.pone.0330830)
Supplement: S17 Appendix — (PDF) [file pone.0330830.s017.pdf]

MFMC Montreal Supplemental Questions - Vietnamese Version	ID của người phụ nữ:	
	Tên của người phỏng vấn:	
	Ngày phỏng vấn:	

Chúng tôi rất biết ơn bạn đã trả lời những câu hỏi của chúng tôi. Chúng tôi có một số câu hỏi thêm về các chủ đề không hoàn toàn được bao gồm trong các câu hỏi trước đây. Những câu hỏi đầu tiên có liên quan đến sức khỏe nói chung của bạn trước khi mang thai.

1. Bạn có bất cứ tình trạng hay bệnh gì hay không *(ví dụ như, bệnh tiểu đường, bệnh tim, hen suyễn, viêm khớp, sốt rét, lao, HIV, viêm gan C, giun sản)?*

☐ Có *(Xin ghi rõ thêm chi tiết)* ___________________________
☐ Không

2. Bạn đã có được điều trị cho tất cả các bệnh này không?

☐ Có, tất cả đã được điều trị
☐ Không, vẫn còn một số hoặc tất cả vẫn chưa được điều trị

3. Bạn đã bao giờ ngưng sự chăm sóc hoặc điều trị cho bất kỳ bệnh nào của bạn hay không

☐ Có *(Xin ghi rõ thêm chi tiết)* ___________________________
☐ Không

4. Bình thường bạn nặng bao nhiêu cân (khi không có thai)?

_________(kg) ________ (g)/ _________(lbs) _______(oz)

5. Chiều cao của bạn là bao nhiêu?

_______ (ft) _______(inches)/ ______ (m) _____ (cm)

6. Những câu đưa ra sau đây có đúng vời sự thật về nhà của bạn hay không?

	Có	*Không*
Nhà của tôi đủ rộng cho mọi người sống trong nhà	☐	☐
Nhà của tôi đủ ấm trong mùa đông	☐	☐
Nhà của tôi đủ yên tĩnh	☐	☐
Nhà của tôi không có nấm mốc và sâu bọ (ví dụ như, côn trùng hay chuột)	☐	☐
Nhà của tôi không có khói trông nhà (bào gồm khói thuốc lá)	☐	☐
Kiến trúc của nhà tôi an toàn (vị dụ như, toà nhà được vững chắc	☐	☐
Nhà của tôi nằm trong khu vực ít hoặc không có ô nhiễm không khí	☐	☐
Nhà tôi nằm trong khu vực an toàn (vị dụ như, không có tội phạm)	☐	☐

7. Mã bưu điện (Postal Code) của bạn sẽ giúp chúng tôi biết thêm về khu vực mà bạn đang ở, mã bưu điện của bạn là gì?

☐☐☐ ☐☐☐

Chúng tôi có 4 câu hỏi về dự định có thai của bạn.

8. Khi bạn có thai em bé này, bạn đã có ý định có thai trong thời điểm đó hay không?

- ☐ Có *(Xin hãy tiêp tục với câu hỏi số 12)*
- ☐ Không
- ☐ Tôi không rõ

9. Nếu bạn không có rõ hoặc không muốn mang thai, bạn đã có sử dụng cách nào để ngăn ngừa mang thai hay không? *(Xin hãy xem những câu trả lời trong câu hỏi số 10 để làm ví dụ nếu cần thiết)*

- ☐ Có
- ☐ Không *(Xin hãy tiêp tục với câu hỏi số 11)*

10. Nếu bạn trả lời CÓ, bạn đã sử dụng các cách ngăn ngừa thai nào?
(Xin hãy cho người phụ nữ trả lời câu hỏi này trước, rồi sau đó đánh dấu tất cả những gì là hợp lý với bạn. Và sau đó hãy tiêp tục với câu hỏi số 12)

- ☐ Cao su [*Condom/ Préservatif*]
- ☐ Cho con bú
- ☐ Thuốc ngăn ngừa có thai
- ☐ Tiêm Depo-Provera
- ☐ Ngăn ngừa trong tử cung (vòng tránh thai)
- ☐ Quan sát chu kỳ kinh nguyên
- ☐ Bạn hay người yêu của bạn không có khả năng mang thai (chẳng hạn vì đã làm phẩu thuật để ngăn ngừa có thai)
- ☐ Rút rời ("kéo ra")
- ☐ Nắp màng/ cổ tử cung (cap)
- ☐ Chèn Norplant dưới da (Norplant là thuốc tránh thai)
- ☐ Kiêng [*Abstinence*]
- ☐ Cách khác *(Xin ghi rõ thêm chi tiết)* _________________________
- ☐ Câu hỏi này không áp dụng đối với tôi

11. Nếu bạn đã không sử dụng cách ngăn ngừa thai, lý do tại sao?
(Xin hãy cho người phụ nữ trả lời câu hỏi này trước, rồi sau đó đánh dấu tất cả những gì là hợp lý với bạn)

- ☐ Không có văn phòng khám bệnh hoặc nhà cung cấp chăm sóc y tế
- ☐ Các phản ứng phụ
- ☐ Không có điều kiện
- ☐ Vì lý do tôn giáo
- ☐ Chồng/ gia đình không cho phép
- ☐ Lý do khác *(Xin ghi rõ thêm chi tiết)* _________________________________
- ☐ Câu hỏi này không áp dụng đối với tôi

Chúng tôi có 5 câu hỏi về sức khoẻ răng lợi của bạn.

12. Nói chung, bạn đánh giá sức khoẻ răng lợi của bạn như thế nào?
(Xin hãy đọc tất cả và đánh dấu một câu trả lời hợp lý nhất với bạn)

- ☐ Tuyệt vời
- ☐ Rất tốt
- ☐ Tốt
- ☐ Khá
- ☐ Không tốt
- ☐ Tôi không biết

13. Bạn có nghĩ rằng bạn có <u>bệnh về lợi</u> hay không?

- ☐ Có
- ☐ Không
- ☐ Tôi không biết

14. Bạn đã từng có điều trị cho các <u>bệnh về lợi</u> như lấy vôi răng và làm láng bề mặt chân răng, hay còn được gồi là "nạo túi" hoặc "làm sạch vôi răng dưới nướu"?

- ☐ Có
- ☐ Không
- ☐ Tôi không biết

15. Chuyên gia nha khoa đã có bao giờ nói với bạn là bạn <u>bị mất xương xung quanh răng của bạn</u> hay không?

- ☐ Có
- ☐ Không
- ☐ Tôi không biết

16. Ngoài trừ đánh răng với bàn chải đánh răng, trong bảy ngày qua, bạn đã sử dụng chỉ nha khoa hoặc bất kỳ thiết bị khác để làm sạch kẽ răng của bạn bao nhiêu lần?

___________(lần)

- ☐ Tôi không biết

Ở một số nước, có một phong tục là một cô gái trẻ có thể cắt một phần nhỏ của vùng kín (âm vật) của mình đi vì lý do truyền thống (hay còn được gọi là cắt bao quy đầu [Circumcision/ circoncision]*). Chúng tôi muốn hỏi bạn 2 câu hỏi về phong tục này.*

17. Phong tục này đã bao giờ được thực hành với bạn không?

- ☐ Có
- ☐ Không *(Xin hãy tiêp tục với câu hỏi số 19)*

18. Nếu bạn trả lời CÓ, khủ vục bị cắt có được khâu lại hay không?

- ☐ Có
- ☐ Không
- ☐ Tôi không biết

Chúng tôi có 9 câu hỏi về sự di chuyển của bạn đến một quốc gia mới.

19. Trước khi sinh em bé mới đây nhất, bạn đã sinh con ở đau và trong khoảng thời gian nào?

___________________________ (quốc gia), __________ (năm)

___________________________ (quốc gia), __________ (năm)

___________________________ (quốc gia), __________ (năm)

___________________________ (quốc gia), __________ (năm)

☐ Câu hỏi này không áp dụng đối với tôi (Không có sinh con trước đây)

20. Bạn đã được bao nhiêu tuổi khi mà bạn định cư tại Canada? ____________(tuổi)

21. Có ai đăng ký cho bạn sang Canada và có trách nhiệm cho bạn ở đây hay không (hay còn gọi là người "tài trợ" [bảo lãnh] cho bạn)?

☐ Có

☐ Không *(Xin hãy tiêp tục với câu hỏi số 23)*

22. Nếu bạn trả lời CÓ, người đó là ai?

(Xin hãy cho người phụ nữ trả lời câu hỏi này trước, rồi sau đó đánh dấu một câu trả lời mà hợp lý với bạn)

☐ Chồng của tôi

☐ Cha mẹ của tôi

☐ Con của tôi

☐ Các tổ chức tư nhân (ví dụ như, nhà thờ, tổ chức phi chỉnh phủ)

☐ Chính phủ

☐ Người khác *(Xin ghi rõ thêm chi tiết)* __________________________

23. Cha của em bé đã sinh ra tại quốc gia nào? __________________________ (quốc gia)

☐ Tôi không biết

24. Cha của em bé có sống với bạn hay không?

☐ Có

☐ Không

25. Cha của em bé có cùng huyết thống với bạn hay không?

☐ Có

☐ Không

26. Nếu bạn đã có một công việc có tiền lương trước khi em bé được sinh ra, bạn đã ngừng làm việc từ khi nào _____(tháng)/______ (năm)

☐ Tôi đã không có công việc

☐ Tôi đã không ngừng làm viêc

27. Nếu bạn là người đã trả tiền chi phí cho việc chăm sóc hoặc dịch vụ y tế của bạn <u>tại CANADA</u> trong khi có thai gần đây nhất, trong khi sinh, hoặc sau khi sinh, bạn đã phải trả những chi phí gì và bao nhiêu tiền? ***(Xin hãy đọc tất cả và đánh dấu tất cả những gì là hợp lý với bạn. Rồi sau đó, xin hãy điền vào ô trống)***

☐ Hẹn với chuyên gia y tế	$ ________
☐ Khám sức khoẻ	$ ________
☐ Xét nghiệm máu	$ ________
☐ Kiểm tra cổ tử cung/ Pap test	$ ________
☐ Kiểm tra tật hay bệnh bẩm sinh của thai nhi (ví dụ như, bệnh Down)	$ ________
☐ Siêu âm	$ ________
☐ Dịch vụ sức khoẻ tâm thần	$ ________
☐ Lớp học về mang thai và sinh con	$ ________
☐ Thuốc	$ ________
☐ Dịch vụ liên quan tới sinh đẻ	$ ________
☐ Những chi phí khác *(Xin ghi rõ thêm chi tiết)* - ________________	$ ________
☐ Câu hỏi này không áp dụng đối với tôi	

Chúng tôi có 7 câu hỏi về sức khoẻ của bạn khi mang thai.

28. Câu trả lời nào sau đây phù hợp nhất để mô tả thói quen hút thuốc lá của bạn trong thời gian mang thai gần đây nhất?

☐ Tôi không hút thuốc lá
☐ Tôi chỉ thỉnh thoảng mới hút thuốc lá
☐ Tôi hút thuốc lá mỗi ngày *(Xin hãy nghi rõ là bạn hút thuốc là bao nhiêu lần trong một ngày)* _________

29. Bạn nặng bao nhiêu cân trong cuối kỳ mang thai của bạn (trước khi sinh em bé)?

_________(kg) ________ (g)/ __________(lbs) _______(oz)

30. Trong tuần vừa rồi, bạn đã ăn hay uống những món đồ sau đây bao nhiêu lần?
(Xin hãy đọc tất cả và ghi số lần vào ô trống)

Đậu lăng và đỗ	_______
Các loại rau lá với mày xanh đậm (ví dụ như, hoa lơ xanh)	_______
Gan	_______
Trái cây gia đình nhà cam như bưởi, quýt, chanh, v.v.	_______
Bánh mì hạt ngũ cốc nguyên chất	_______
Nước cam với chất bổ sung Vitamin D	_______
Sữa bò	_______

31. <u>Ít nhất một tháng trước khi mang thai</u>, bạn đã có sử dụng các vitamin hoặc axit folic hàng ngày để bổ sung sức khoẻ trong khi mang thai hay không?

☐ Có *(Xin hãy tiêp tục với câu hỏi số 33)*
☐ Không

32. Nếu bạn trả KHÔNG, lý do tại sao?
(Xin hãy cho người phụ nữ trả lời câu hỏi này trước, rồi sau đó đánh dấu tất cả những gì là hợp lý với bạn)

- ☐ Không biết những thứ đó dùng để làm gì
- ☐ Không thể tìm thấy nó
- ☐ Không có điều kiện để mua
- ☐ Những thứ đó không có sẵn
- ☐ Tôi không cần thiết phải sử dụng
- ☐ Đã không ai nói tôi nên sử dụng những thứ đó
- ☐ Lý do khác *(Xin ghi rõ thêm chi tiết)* ______________________
- ☐ Câu hỏi này không áp dụng đối với tôi

---

33. <u>Trong khi mang thai</u>, bạn đã có sử dụng các vitamin hàng ngày để bổ sung sức khoẻ trong khi mang thai hay không?

- ☐ Có *(Xin hãy tiêp tục với câu hỏi số 35)*
- ☐ Không

---

34. Nếu bạn trả KHÔNG, lý do tại sao?
((Xin hãy cho người phụ nữ trả lời câu hỏi này trước, rồi sau đó đánh dấu tất cả những gì là hợp lý với bạn)

- ☐ Không biết những thứ đó dùng để làm gì
- ☐ Không thể tìm thấy nó
- ☐ Không có điều kiện để mua
- ☐ Những thứ đó không có sẵn
- ☐ Tôi không cần thiết phải sử dụng
- ☐ Đã không ai nói tôi nên sử dụng những thứ đó
- ☐ Lý do khác *(Xin ghi rõ thêm chi tiết)* ______________________
- ☐ Câu hỏi này không áp dụng đối với tôi

---

35. Cuộc phỏng vấn của chúng ta đã kết thúc. Bạn có muôn nói thêm về bất cứ điều gì khác về chủ để chúng ta đã nói về hay không? Hay bạn có điều gì khác bạn muốn bổ sung thêm hay không?
