# Supplementary material for: Migrant-friendly maternity care in Montreal, Canada: A cross-sectional study on migrant women’s care perspectives
Source: PLoS One. 2025 Aug 21;20(8):e0330830. doi: 10.1371/journal.pone.0330830 (PMC12370051; doi:10.1371/journal.pone.0330830)
Supplement: S18 Appendix — (PDF) [file pone.0330830.s018.pdf]

MFMC-MTL Medical Record Review

Study ID: ☐☐-☐☐☐☐

NAME of individual who completed MRR: ___________________
DATE MRR completed: _____/_______/_______ **(day/month/year)**
TIME TO COMPLETE MRR: _____________**(mins)**

MATERNAL MEDICAL HISTORY

1. **Maternal Birth date**: ______ / _____ ; Month / Year

2. **Health Insurance** (check all that apply):
   - ☐ Medicare (RAMQ or other provincial health card)
   - ☐ IFHP
   - ☐ Private
   - ☐ None

3. **Prenatal Care**: Gestational age at first prenatal visit (earliest noted prenatal visit in medical record):

   ______weeks________ days or ☐ Info. not in chart ; ☐ 1st trimester (0- 12 weeks) or ☐2nd trimester (13-28 weeks)

   or ☐ 3rd (29-40 weeks) trimester or ☐ Info not in chart

4. **Maternal infectious diseases** (if **yes**, note date of diagnosis/test)

		**YES** *DD/MM/YYYY*	**NO**	**Info. not in chart**
a)	HIV/AIDS	☐ Specify date of diagnosis/test: ________________ or ☐ Date unknown	☐	☐
b)	Hepatitis B (HbSAg)	☐ Specify date of diagnosis/test: ________________ or ☐ Date unknown	☐	☐
c)	Genital Herpes	☐ Specify date of diagnosis/test: ________________ or ☐ Date unknown	☐	☐
d)	VDRL (Syphilis)	☐ Specify date of diagnosis/test: ________________ or ☐ Date unknown	☐	☐
e)	Chlamydia	☐ Specify date of diagnosis/test: ________________ or ☐ Date unknown	☐	☐
f)	Gonorrhea	☐ Specify date of diagnosis/test: ________________ or ☐ Date unknown	☐	☐
g)	HPV	☐ Specify date of diagnosis/test: ________________ or ☐ Date unknown	☐	☐
h)	Bacterial vaginosis	☐ Specify date of diagnosis/test: ________________ or ☐ Date unknown	☐	☐
i)	TB	☐ Specify date of diagnosis/test: ________________ or ☐ Date unknown	☐	☐
j)	Malaria	☐ Specify date of diagnosis/test: ________________ or ☐ Date unknown	☐	☐
k)	Hepatitis C	☐ Specify date of diagnosis/test: ________________ or ☐ Date unknown	☐	☐
l)	Toxoplasmosis	☐ Specify date of diagnosis/test: ________________ or ☐ Date unknown	☐	☐
m)	Schistosomiasis	☐ Specify date of diagnosis/test: ________________ or ☐ Date unknown	☐	☐
n)	Strongyloidiasis	☐ Specify date of diagnosis/test: ________________ or ☐ Date unknown	☐	☐
o)	Erythema infectiosum (fifth disease)	☐ Specify date of diagnosis/test: ________________ or ☐ Date unknown	☐	☐
p)	Varicella (chicken pox)	☐ Specify date of diagnosis/test: ________________ or ☐ Date unknown	☐	☐

MATERNAL MEDICAL HISTORY

5. **Group Beta Strep (GBS)**:
   - ☐ Reactive/Positive
   - ☐ Negative
   - ☐ Recorded as unknown
   - ☐ Info. not in chart

6. **Rubella immune**:
   - ☐ Yes
   - ☐ No
   - ☐ Recorded as unknown
   - ☐ Info. not in chart

7. **Maternal chronic diseases**:

	YES	NO	Info. not in chart
a) Cancer	☐ Specify:	☐	☐
b) Cardiovascular (heart) disease	☐ Specify:	☐	☐
c) Hypertension	☐ Specify:	☐	☐
d) Respiratory disease (asthma, COPD)	☐ Specify:	☐	☐
e) Diabetes	☐ Specify:	☐	☐
f) Mental illness (e.g., depression, bipolar disorder, schizophrenia, anxiety disorder)	☐ Specify:	☐	☐
g) Neurological conditions (e.g., paralysis, seizures, pain, altered cognition):	☐ Specify:	☐	☐
h) Arthritis	☐ Specify:	☐	☐
i) Osteoporosis	☐ Specify:	☐	☐

8. **Complications during pregnancy:**

	YES	NO	Info. not in chart
a) Gestational diabetes (GDM)	☐	☐	☐
b) Gestational hypertension	☐	☐	☐
c) Deep vein thrombosis	☐	☐	☐
d) Anaemia	☐	☐	☐
e) Hyperemesis gravidarum	☐	☐	☐
f) Urinary Tract Infections (UTI)	☐	☐	☐
g) Chorioamnionitis	☐	☐	☐
h) Maternal fever	☐	☐	☐
i) Pre-eclampsia or eclampsia	☐	☐	☐
j) Oligohydraminos	☐	☐	☐
k) Placenta Previa	☐	☐	☐
l) Placenta abruption	☐	☐	☐
m) Placenta accrete	☐	☐	☐
n) Premature rupture of membranes (PROM)	☐	☐	☐
o) Intrauterine growth retardation (IUGR)	☐	☐	☐
p) Congenital anomaly (e.g., neural tube defect), specify:________________	☐	☐	☐

9. **Admitted to hospital** for any reason during pregnancy:

YES	NO	Info. not in chart
☐ Specify reason: ______________________________________<br>or ☐ Info. not in chart	☐	☐

MATERNAL MEDICAL HISTORY

10. **Tests/Screens** during pregnancy:

a) **Ultrasound(s)**: ☐ Yes ☐ No or ☐ Info not in chart

If **yes** (check all that apply), ☐ 1st trimester (0-12 weeks) ☐2nd trimester (13-28 weeks) ☐ 3rd trimester (29-40weeks) or ☐ Info not in chart

If **yes** (check all that apply), ☐ Dating ultrasound (10-13 weeks) ☐Screening ultrasound (18-20 weeks) or ☐ Info not in chart

b) **Genetic testing**:

	YES	NO	Info not in chart
▪ Cystic fibrosis	☐	☐	☐
▪ Sickle cell disease	☐	☐	☐
▪ Thalassemia	☐	☐	☐
▪ Tay Sachs	☐	☐	☐
▪ T21	☐	☐	☐

c) **Chorionic Villus Sampling** ☐ Yes ☐ No; or ☐ Info. not in chart

d) **Amniocenthesis** ☐ Yes ☐ No; or ☐ Info. not in chart

11. **Social issues** during pregnancy

		**YES**	**NO**	**Info. not in chart**
a)	Smoking	☐	☐	☐
b)	Drug use	☐	☐	☐
c)	Alcohol use	☐	☐	☐
d)	Violence (conjugal or other)	☐	☐	☐
e)	Pregnancy the result of rape	☐	☐	☐
f)	Food insecurity	☐	☐	☐

If **yes**, to any of the above describe: ______________________________

____

____

____

12. **Assisted Reproduction** (ART):

**YES**	**NO**	**Info. not in chart**
☐ If yes, indicate type:<br>☐ In vitro fertilization,<br>☐ Artificial insemination,<br>☐ Other, specify______________________<br>☐ Info. not in chart	☐	☐

13. **Pre-pregnancy weight**: ________ (lbs) or_________ (Kg) or ☐ Info. not in chart

Height: _________ (cm) or __________ (ft & inches) or ☐ Info. not in chart

Maternal Weight at birth: _______ (lbs) or________ (Kg) or ☐ Info. not in chart

Study ID: ☐☐-☐☐☐☐

MATERNAL MEDICAL HISTORY

14. **Female circumcision** (FGM, genital cutting):

**YES**	**NO**	**Info. not in chart**
☐ If yes, indicate degree (circle one) : 1° 2° 3° 4°	☐	☐

☐ Info. not in chart or ☐ N/A

15. **Gravida:** ______

Parity: _______ (live_____, stillbirths_____)

Miscarriages ______ (Spontaneous Abortion) Therapeutic Abortion______ (Induced Abortion) or Abortions total (therapeutic or miscarriage) ________

**Multiples count as one pregnancy/birth. Include current birth in parity.*

PREVIOUS PREGNANCIES

16. If applicable and , for each **previous birth** indicate date, mode of delivery and country:

☐ **N/A** (no previous births)

a) ______ (month), _____ (year) or ☐ Info. not in chart; ☐ Vaginal or ☐ C/B or ☐ Info. not in chart;

_________________________ (country) or ☐ Info. not in chart

b) ______ (month), _____ (year) or ☐ Info. not in chart ; ☐ Vaginal or ☐ C/B or ☐ Info. not in chart;

_________________________ (country) or ☐ Info. not in chart

c) ______ (month), _____ (year) or ☐ Info. not in chart; ☐ Vaginal or ☐ C/B or ☐ Info. not in chart;

_________________________ (country) or ☐ Info. not in chart

d) ______ (month), _____ (year) or ☐ Info. not in chart; ☐Vaginal or ☐ C/B or ☐ Info. not in chart ;

_________________________ (country) or ☐ Info. not in chart

LABOUR AND DELIVERY

17. a) **Date and time of admission** to labour unit/birthing centre: ____ / _____ / ______ __ __: __ __
Day / Month / Year (24-hour clock)

Day of the week admitted: ☐ Mo ☐ Tu ☐ We ☐ Th ☐ F ☐ Sa ☐ Su

b) Upon admission:

	**YES**	**NO**	**Info. not in chart**
**Contracting**:	☐	☐	☐

Cervical dilation: _____ cm or ☐ Info. not in chart;

	**YES**	**NO**	**Info. not in chart**
**Membranes spontaneously ruptured**:	☐	☐	☐

	**YES**	**NO**	**Info. not in chart**
c) **Transferred** from another hospital for delivery:	☐	☐	☐

Study ID: ☐☐-☐☐☐☐

MATERNAL MEDICAL HISTORY

18. **Induction/Augmentation:** ☐ Yes ☐ No

a) If **yes,** Gestational age: _______ (weeks) ______ (days) Or ☐ N/A;
Gestational age based on: ☐ LMP , ☐ early ultrasound (11-14 weeks gestation)
☐ other, specify ________________ or ☐ not indicated; Or ☐ N/A

b) If **yes**, specify reason(s) for induction: ☐ PROM, ☐ Fetal growth restriction, ☐ Chorioamnionitis,
☐ Suspected macrosomia, ☐ Hypertensive disorder, ☐ Post-datism, ☐ Elective,
☐ Reason not indicated, ☐ other, specify ___________________ or ☐ N/A

c) If **yes**, specify type(s) of induction/augmentation:

	**Yes or No or N/A**	**Day / Month / Year or Info. not in chart or N/A**	**24-hour-clock or Info. not in chart ☐r N/A**	**Dilation (cm) or Info. not in chart or N/A**	**Effacement (%) or Info. not in chart or N/A**	**Station (-3…+2) or Info. not in chart or N/A**
Pitocin/Syntocinon (synthetic oxytocin)						
Prostaglandin gels [Cervidil/Prepidil/ ProstinE2 (Dinoprostone)]						
Balloon catheter						
Other, specify________________						

19. **Amniotomy** (Artificial Rupture of Membrane – AROM):

YES ☐ Specify: ____/______/_____ __ __: __ __
Day / Month / Year (24-hour-clock)

NO ☐ **Info not in chart** ☐

If **yes,** Dilation at time of ARM: ________cm
or ☐ Info not in chart or ☐ N/A

20. **Sweeping (stripping) of membranes**:

YES ☐ Specify: ____/______/_____ __ __: __ __
Day / Month / Year (24-hour-clock)

NO ☐ **Info not in chart** ☐

If **yes,** Dilation at time of sweeping: _______cm
or ☐ Info not in chart or ☐ N/A

21. Total number of **vaginal exams** performed: _______ or ☐ Info. not in chart

How many individuals performed a vaginal exam: ______ or ☐ Info. not in chart

22. **Fetal heart monitoring** (FHR):

**Type**	**Yes or No**
Intermittent auscultation	
Intermittent electronic fetal monitoring	
Continuous electronic fetal monitoring	

MATERNAL MEDICAL HISTORY

		YES	NO	Info not in chart
23.	**Epidural:**	☐ If **yes**, Date/Time of epidural ____/______/_____ __ __: __ __<br>Day / Month / Year (24-hour-clock)	☐	☐

If **yes,** dilation at time of administration of epidural _________cm
or ☐ Info not in chart or ☐ N/A

24. Other **medical pain management** during labour and delivery:

	YES	NO	Info. not in chart
a) Narcotic (Demerol, Sufentanil, Fentanyl, morphine, etc.)	☐	☐	☐
b) Local Anaesthetic (pudendal block)	☐	☐	☐
c) Nitrous oxide	☐	☐	☐
d) Other, specify:	☐	☐	☐

25. **Nurse care during labour**:

Number of nurses responsible for care: ____________________ or ☐ Info. not in chart

Number of times responsibility 'changed hands': ____________ or ☐ Info. not in chart

26. **Physician/midwifery care during labour and delivery**:

a) During **labour** (check all that apply): ☐ **N/A** (no labour)

☐ **MD(s)**	☐ Info. not in chart
☐ **Resident(s)**	☐ Info. not in chart
☐ **Midwife(s)**	☐ Info. not in chart

Total Number of HCPs that provided physician/midwifery care during labour (including residents/fellows and medical students if documented): ___________ or ☐ Info. not in chart
or ☐ N/A (no labour)

b) During **delivery** (check all that apply):

☐ **MD(s)**	☐ Info. not in chart
☐ **Resident(s)**	☐ Info. not in chart
☐ **Midwife(s)**	☐ Info. not in chart

Was the **primary HCP** who attended the delivery the same as the HCP who provided prenatal care:
☐ Yes
☐ No
☐ Info. not in chart

27. Documentation of **Strategies used to facilitate communication** during labour & birth:
☐ Yes ☐ No or ☐ N/A
If **yes**, specify what was recorded by the HCP(s), including what was written in their notes:

____

____

Study ID: ☐☐-☐☐☐☐

MATERNAL MEDICAL HISTORY

28. **Number of Infants**: ☐ Single ☐ Twins ☐ Multiple

29. **Type of Birth**:
☐ Vaginal (spontaneous vaginal delivery – SVD)
☐ Vaginal with forceps
☐ Vaginal with vacuum extraction
☐ Planned Caesarean birth (C/B)
☐ Emergency C/B (i.e., woman had a labour)

CAESAREAN BIRTH

30. If **C/B**, second opinion was obtained for decision to perform C/B: ☐ Yes ☐ No or
☐ Info. not in chart or ☐ N/A

If **C/B**, Stage of labour: ☐ 1st (not fully dilated) ☐ 2nd (10 cm dilated) or ☐ Info. not in chart or ☐ N/A (no C/B or no labour)

If **C/B,** Specify reason or ☐ Info. not in chart or ☐ N/A

Dystocia/Failure to progress due to:<br>☐ Dystocia/FTP<br>☐ Shoulder dystocia<br>☐ Cephalo-pelvic disproportion (CPD)<br>☐ Failed forceps<br>☐ Failed induction<br>Fetal Distress as measured by:<br>☐ abnormal pH<br>☐ (FHR) Fetal heart rate, decels,<br>☐ Meconium staining<br>☐ Prolapsed cord<br>☐ Fetal distress (reason not specified)<br>Fetal Growth:<br>☐ Intrauterine growth retardation<br>☐ Macrosomia (> 4,000 g infant)<br>☐ Oligohydraminos<br>☐ Congenital malformation, specify________________________<br>Placenta:<br>☐ Placenta previa<br>☐ Placenta abruptio<br>☐ Placenta accrete	Malpresentation or lie:<br>☐ Breech<br>☐ Footling<br>☐ Transverse Lie<br>☐ Malpresentation (type not specified)<br>Maternal infection:<br>☐ HIV/AIDS<br>☐ Genital Herpes<br>☐ Maternal fever/Infection (not specified)<br>Maternal condition:<br>☐ Pre-eclampsia or eclampsia<br>☐ GDM<br>☐ Premature rupture of membranes<br>☐ Previous C/B, classical incision ☐<br>☐ Uterine scar<br>☐ Hemorrhage<br>☐ Multiple gestation<br>☐ Ovarian cysts, tumors in pelvic cavity<br>☐ Pelvic abnormality<br>☐ Other condition, specify ___________

- If **CPD**, justification recorded for this indication: ________________________________Or ☐ Info. not in chart or ☐ N/A
- If **Dystocia/FTP**:

Last recorded dilation: _____ cm or ☐ Info. not in chart or ☐ N/A ;
Effacement: _____ % or ☐ Info. not in chart or ☐ N/A;
Day dilation/effacement recorded: ____/______/_____ or ☐ Info. not in chart or ☐ N/A;
Day / Month / Year
Time dilation/effacement recorded:__ __: __ __ or ☐ Info. not in chart or ☐ N/A;
(24-hour-clock)

Study ID: ☐☐-☐☐☐☐

BIRTH SUMMARY

31. **Meconium** present: ☐ Yes
☐ No
☐ Info. not in chart

32. **Fetal scalp pH** level (last measurement): ☐ < 7.20 (low O_2) ☐ ≥ 7.20 or ☐ not measured

33. **Episiotomy**: ☐ Yes
☐ No

34. **Perineal Tear/Laceration**: ☐ Yes If **yes**, specify degree of laceration (circle one): 1° 2° 3° 4°
Or ☐ Info. not in chart or
☐ No ☐ N/A

35. **Oxytocin** administered after delivery: ☐ Yes
☐ No
☐ Info. not in chart

36. **Maternal complications** during **labour, birth& immediately after birth** (check all that apply):

☐ Blood transfusion	☐ Cardiac arrest	☐ Hysterectomy
☐ Hemorrhage	☐ Pulmonary edema	☐ Puerperal sepsis
☐ Uterine rupture	☐ Cardiomyopathy	☐ Repair of bladder, urethra or intestine
☐CVA	☐ Obstetric shock	☐ Pre-Eclampsia/eclampsia
☐ Hepatic failure	☐ Obstetric embolism	☐ Anesthesia complications
☐ Acute psychosis	☐ Seizure	☐ Sickle cell anemia crisis
☐ MI	☐ Respiratory distress	☐ Acute renal failure

Or ☐ **No complications**

37. **Maternal complications postpartum** (check all that apply):

☐Infection, if **yes** specify: ☐ Perineum ☐ Uterine ☐ Breast/mastitis
☐ Bladder/UTI ☐ C/B incision site ☐ Sepsis
☐ Other, specify _______________ ☐ Not specified
☐ N/A

☐ Hemorrhoids
☐ Constipation
☐ Incontinence (urinary or fecal)
☐ Excessive lochia (“heavy menstrual flow”) or clots
☐ Fundus is not firm and/or above the umbilicus
☐ DVT
☐ Mental health (anxiety, depression)
☐ Breastfeeding difficulties (pain, difficulty latching)

Or ☐ **No complications**

Study ID: ☐☐-☐☐☐☐

INFANT 1

Infant 1

38. a) Date and time of Birth : ____ / _____ / ______ __ __: __ __ ;
Day / Month / Year (24-hour clock)

Day of week: ☐ Mo ☐ Tu ☐ We ☐ Th ☐ F ☐ Sa ☐ Su

39. a) Sex of infant: ☐ Female ☐ Male

40. a) Presentation at birth: ☐ vertex ☐ face ☐ brow ☐ breech ☐ other, specify ____________

41. a) Lie: ☐ Longitudinal ☐ Transverse

42. a) Birth weight: ________ grams

43. a) Gestational age: ____ weeks ___ days;
Based on: ☐ LMP or ☐ early ultrasound (11-14 weeks gestation)
or ☐ other, specify ________________ or ☐ not indicated.

44. a) Apgar (Total): ____ 1 min ____ 5 mins

45. a) Admission to neonatal intensive care unit (NICU) ☐ Yes ☐ No
If **yes**, specify indication for admission _____________________ or ☐ N/A

46. a) Was infant transferred to another hospital? ☐ Yes ☐ No
If **yes**, specify indication for transfer _____________________ or ☐ N/A

47. a) **Infant complications** (check all that apply): or ☐ No complications

☐ Prematurity	☐ Jaundice	☐ Respiratory distress	☐ Lung disorder
☐ LBW	☐ Nuchal cord	☐ Pneumonia	☐ GI disorder
☐ SGA	☐ Birth injury	☐ Ventilatory support	☐ Blood disorder
☐ Postmature	☐ Hypoglycemia	☐ Resuscitation	☐ Heart disorder
☐ Macrosomia	☐ Intravenous fluids	☐ Vision/hearing disorder	☐ Brain/NS disorder
☐ Clavicle fracture	☐ Infection	☐ Seizure	☐ Cerebral infarction
☐ Birth defect	☐ Sepsis	☐ Substance exposure	☐ Hydrops fetalis
☐ Transfusion	☐ Surgery	☐ Central venous/ arterial catheter	
☐ Intraventricular hemorrhage		☐ Hypoxic ischemic encephalopathy	
☐ Birth trauma (intracranial hemorrhage paralysis)			

Study ID: ☐☐-☐☐☐☐

INFANT 2

Infant 2 ☐ Not Applicable (Single Infant)

38. b) Date and time of Birth : ____ / _____ / ______ __ __: __ __ ;
Day / Month / Year (24-hour clock)

Day of week: ☐ Mo ☐ Tu ☐ We ☐ Th ☐ F ☐ Sa ☐ Su

39. b) Sex of infant: ☐ Female ☐ Male

40. b) Presentation at birth: ☐ vertex ☐ face ☐ brow ☐ breech ☐ other, specify ____________

41. b) Lie: ☐ Longitudinal ☐ Transverse

42. b) Birth weight: ________ grams

43. b) Gestational age: ____ weeks ___ days;
Based on: ☐ LMP or ☐ early ultrasound (11-14 weeks gestation)
or ☐ other, specify ________________ or ☐ not indicated.

44. b) Apgar (Total): ____ 1 min ____ 5 mins

45. b) Admission to neonatal intensive care unit (NICU) ☐ Yes ☐ No
If **yes**, specify indication for admission _____________________ or ☐ N/A

46. b) Was infant transferred to another hospital? ☐ Yes ☐ No
If **yes**, specify indication for transfer _____________________ or ☐ N/A

47. b) **Infant complications** (check all that apply): or ☐ No complications

☐ Prematurity	☐ Jaundice	☐ Respiratory distress	☐ Lung disorder
☐ LBW	☐ Nuchal cord	☐ Pneumonia	☐ GI disorder
☐ SGA	☐ Birth injury	☐ Ventilatory support	☐ Blood disorder
☐ Postmature	☐ Hypoglycemia	☐ Resuscitation	☐ Heart disorder
☐ Macrosomia	☐ Intravenous fluids	☐ Vision/hearing disorder	☐ Brain/NS disorder
☐ Clavicle fracture	☐ Infection	☐ Seizure	☐ Cerebral infarction
☐ Birth defect	☐ Sepsis	☐ Substance exposure	☐ Hydrops fetalis
☐ Transfusion	☐ Surgery	☐ Central venous/ arterial catheter	
☐ Intraventricular hemorrhage		☐ Hypoxic ischemic encephalopathy	
☐ Birth trauma (intracranial hemorrhage paralysis)			

Study ID: ☐☐-☐☐☐☐

INFANT 3

Infant 3 ☐ Not Applicable (Single/twins)

38. c) Date and time of Birth : ____ / _____ / ______ __ __: __ __ ;
Day / Month / Year (24-hour clock)

Day of week: ☐ Mo ☐ Tu ☐ We ☐ Th ☐ F ☐ Sa ☐ Su

39. c) Sex of infant: ☐ Female ☐ Male

40. c) Presentation at birth: ☐ vertex ☐ face ☐ brow ☐ breech ☐ other, specify ____________

41. c) Lie: ☐ Longitudinal ☐ Transverse

42. c) Birth weight: ________ grams

43. c) Gestational age: ____ weeks ___ days;
Based on: ☐ LMP or ☐ early ultrasound (11-14 weeks gestation)
or ☐ other, specify ________________ or ☐ not indicated.

44. c) Apgar (Total): ____ 1 min ____ 5 mins

45. c) Admission to neonatal intensive care unit (NICU) ☐ Yes ☐ No
If **yes**, specify indication for admission _____________________ or ☐ N/A

46. c) Was infant transferred to another hospital? ☐ Yes ☐ No
If **yes**, specify indication for transfer _____________________ or ☐ N/A

47. c) **Infant complications** (check all that apply): or ☐ No complications

☐ Prematurity	☐ Jaundice	☐ Respiratory distress	☐ Lung disorder
☐ LBW	☐ Nuchal cord	☐ Pneumonia	☐ GI disorder
☐ SGA	☐ Birth injury	☐ Ventilatory support	☐ Blood disorder
☐ Postmature	☐ Hypoglycemia	☐ Resuscitation	☐ Heart disorder
☐ Macrosomia	☐ Intravenous fluids	☐ Vision/hearing disorder	☐ Brain/NS disorder
☐ Clavicle fracture	☐ Infection	☐ Seizure	☐ Cerebral infarction
☐ Birth defect	☐ Sepsis	☐ Substance exposure	☐ Hydrops fetalis
☐ Transfusion	☐ Surgery	☐ Central venous/ arterial catheter	
☐ Intraventricular hemorrhage		☐ Hypoxic ischemic encephalopathy	
☐ Birth trauma (intracranial hemorrhage paralysis)			

Study ID: __ __ - __ __ __ __

ADDITIONAL MRR NOTES
