# Supplementary material for: Migrant-friendly maternity care in Montreal, Canada: A cross-sectional study on migrant women’s care perspectives
Source: PLoS One. 2025 Aug 21;20(8):e0330830. doi: 10.1371/journal.pone.0330830 (PMC12370051; doi:10.1371/journal.pone.0330830)
Supplement: Attachment — Submitted filename: MFMC_Response to reviewers_June 2025.docx [file pone.0330830.s021.docx]

We would like to thank the reviewers for their positive feedback. We have reviewed and responded to their comments below and have made changes accordingly in the manuscript (highlighted in yellow). We look forward to your response.

**Reviewer #1**:

1- This is a perfectly publishable study. The specific restrictions on data availability are justified in the manuscript. The strengths and limitations section is considered and fair. *I would question how the service landscape/quality may have changed since data collection as this was 10 years ago*. Other than that I have no hesitation in recommending this article for publication.

In recent years, there was a change to improve prenatal care access for asylum seekers in Québec. We added the following sentence to the discussion section to include this information:

“Since 2022, to improve access to maternity care for asylum seekers, PRAIDA- the Regional program for the settlement and integration of Asylum Seekers in Québec, implemented a triage system in which pregnant women are screened and directly referred to a hospital for follow-up care. However, no additional policies to improve access for other migrant groups, have been introduced.”

To further address the issue of older data and changes in the healthcare system and migration context, we added the following sentence to the limitations section:

“Moreover, since the data were collected 10 years ago, it’s possible that the population and migrants’ maternity experiences have changed and the results do not capture the current situation. However, there has been limited policy change in recent years regarding migrants and maternity healthcare in Québec, so the issues related to MFMC identified in this study are likely still relevant. Furthermore, the healthcare system has faced increasing strain, largely due to the pandemic, and migrants’ vulnerabilities have intensified as a result of ongoing sociopolitical and economic challenges, suggesting that these issues may have worsened over time.”

**Reviewer #2**:

1- Although the study is well designed, described and discussed after the findings, there are a few places where adding some explanations or wording could improve the understanding for the readers. Please see them in the attached file. Thanks.

We incorporated all of the small changes that were suggested. Responses to comments and other changes made in the manuscript, are summarised below.

2- Can you explain why the sample was taken from 10 years ago, given that things may have changed a lot since such period (especially now with more migration and political situations)?

The study was conducted in 2014-2015 and there was a delay in publishing the results. As mentioned in our response above to Reviewer 1, to acknowledge this issue, we added a sentence to the limitations section.

3- Important to describe as much as possible how the study requirements reduced this figure tremendously to become only 22% of the total number! … Probably the majority of excluded ones were from USA? This would be good for the reader to know… After looking at the Figure 1, it is clear that the vast majority were Born in Canada or from a Western country, >= 8 years in Canada = 8,081 so it's important to mention such number then, for the reader to understand the context.

To clarify the description of the sample in the methods section, we added the following sentence: “…2962 women were confirmed to meet the eligibility criteria and 2636 of these women (89%) consented and participated in the study (Fig. 1).”

4- Important consideration (mothers with stillborn/neonatal deaths were excluded to avoid burdening the mother) to be put as part of the weaknesses of the study, because you may have found different or more critical associations if included...

We added the following sentence to the limitations section: “Since we excluded some women due their vulnerability (e.g., those who had a stillbirth), we were not able to determine the eligibility of all women who gave birth during the study period, and we removed participants with missing information from our regression analyses, it is possible that the results are not fully representative.”

5- It is always good to add the relative weakness of being a cross-sectional sampling study, where one is not certain of any biases (e.g., as mentioned women with lower education, who may go somewhere else...), plus the fact that all connections with "results" are only associations, not predictions.

To acknowledge the limitations of the cross-sectional design, we added the following sentence in the limitations section: “Due to the cross-sectional design, we cannot explain the relationships between the socioeconomic and migration variables and the MFMC outcomes, and the results only reflect one point in time.”

6- Any mention about whether care has been improved by policies in recent years? (considering that the sample was from several years ago...)

There is one policy that was recently implemented to improve prenatal care access for asylum seekers in Québec. We added this to the discussion section. Otherwise, there has been no real change in policies, and the overall situation in terms of healthcare and the conditions for migrants, has become more difficult. This is now explained in the limitations section. See response to Reviewer 1.
